# A universal approach to determine footfall timings from kinematics of a single foot marker in hoofed animals

**DOI:** 10.7717/peerj.783

**Published:** 2015-03-26

**Authors:** Sandra D. Starke, Hilary M. Clayton

**Affiliations:** 1 School of Electronic, Electrical and Systems Engineering, University of Birmingham, Edgbaston, Birmingham, West Midlands, UK; 2 Sport Horse Science, LC, Mason, MI, USA

**Keywords:** Kinematics, Footfall timings, Horse, Stride segmentation, Biomechanics, Footfall events, Motion capture, Stride cutting, Event detection, Contact

## Abstract

The study of animal movement commonly requires the segmentation of continuous data streams into individual strides. The use of forceplates and foot-mounted accelerometers readily allows the detection of the foot-on and foot-off events that define a stride. However, when relying on optical methods such as motion capture, there is lack of validated robust, universally applicable stride event detection methods. To date, no method has been validated for movement on a circle, while algorithms are commonly specific to front/hind limbs or gait. In this study, we aimed to develop and validate kinematic stride segmentation methods applicable to movement on straight line and circle at walk and trot, which exclusively rely on a single, dorsal hoof marker. The advantage of such marker placement is the robustness to marker loss and occlusion. Eight horses walked and trotted on a straight line and in a circle over an array of multiple forceplates. Kinetic events were detected based on the vertical force profile and used as the reference values. Kinematic events were detected based on displacement, velocity or acceleration signals of the dorsal hoof marker depending on the algorithm using (i) defined thresholds associated with derived movement signals and (ii) specific events in the derived movement signals. Method comparison was performed by calculating limits of agreement, accuracy, between-horse precision and within-horse precision based on differences between kinetic and kinematic event. In addition, we examined the effect of force thresholds ranging from 50 to 150 N on the timings of kinetic events. The two approaches resulted in very good and comparable performance: of the 3,074 processed footfall events, 95% of individual foot on and foot off events differed by no more than 26 ms from the kinetic event, with average accuracy between −11 and 10 ms and average within- and between horse precision ≤8 ms. While the event-based method may be less likely to suffer from scaling effects, on soft ground the threshold-based method may prove more valuable. While we found that use of velocity thresholds for foot on detection results in biased event estimates for the foot on the inside of the circle at trot, adjusting thresholds for this condition negated the effect. For the final four algorithms, we found no noteworthy bias between conditions or between front- and hind-foot timings. Different force thresholds in the range of 50 to 150 N had the greatest systematic effect on foot-off estimates in the hind limbs (up to on average 16 ms per condition), being greater than the effect on foot-on estimates or foot-off estimates in the forelimbs (up to on average ±7 ms per condition).

## Introduction

The analysis of animal movement has held a long fascination for scientists. Since Eadweard Muybridge presented highspeed photographs of the flight phase in galloping horses in the late 19th century (Muybridge, 2000), there has been an escalating interest in animal locomotion through the study of kinematics, kinetics and neuro-muscular control (Dickinson et al., 2000; Alexander, 2003; Biewener, 2003). These studies commonly rely on the determination of stride events, specifically ‘foot on’ and ‘foot off’ timings, to define movement cycles. Stride events allow comparison of locomotor parameters within and across animals and species, calculation of parameters such as stance time, swing time and duty factor (Biewener, 1983) and even approximation of kinetic features (Witte, Knill & Wilson, 2004). Footfall timings can be established in different ways, with force plates generally considered the gold standard (Merkens & Schamhardt, 1994; Witte, Knill & Wilson, 2004): a measurable force means that the foot is weight-bearing and hence in stance. If the force falls to zero, the foot has left the ground and is hence in swing.

Despite the advantages of footfall event detection using forceplates, this technology is not always suitable in the wider context of a study: force plates may not be used due to venue restrictions such as during competitions (Deuel & Park, 1991; Clayton, Colborne & Burns, 1995; Clayton, 1997; Hodson, Clayton & Lanovaz, 1999) or due to unfavourable environments such as during hydrotherapy (Hunt, 2001; Mooij et al., 2013). Further, the limited capture area of force plates would prohibit research utilising large numbers of consecutive strides on racetracks (Witte, Hirst & Wilson, 2006; Parsons, Pfau & Wilson, 2008b; Pfau et al., 2009) or on turns and circles (Clayton & Sha, 2006; Hobbs, Licka & Polman, 2011; Starke et al., 2012a). Force plates may also simply not be part of a laboratory’s inventory, or synchronisation and processing of multiple data streams can prove difficult to accomplish. Further, despite the conceptually clear definition of footfall events by means of forceplate data, the exact timings of these events still depend on the set threshold at which one considers a force sufficient to indicate weight bearing. These thresholds tend to vary in the literature—for example, in human studies researchers have used a vertical force threshold of 20 N (Hobbs et al., 2010) or 100 N (Chang & Kram, 2007) to detect touch down and foot off, while in substantially heavier species such as horses thresholds of 50 N (Peham, Scheidl & Licka, 1999; Witte, Knill & Wilson, 2004; Boye et al., 2014) have been employed. However, due to the sharp rise in force during impact (Merkens & Schamhardt, 1994), the effect of different thresholds may be small for hoofed animals. In humans, a force threshold of 20 N instead of 10 N for example may cause event detection to differ by only 5 ms (Leitch et al., 2011).

As an alternative to force plates, movement characteristics of the limbs or body are used to approximate foot on and foot off events. In the past, hoof kinematics have been utilised qualitatively to segment strides in many studies. Hoof-mounted accelerometers have been used as an alternative to optical motion capture or video (Schamhardt & Merkens, 1994; Witte, Knill & Wilson, 2004; Parsons & Wilson, 2006; Witte, Hirst & Wilson, 2006; Parsons et al., 2008a; Parsons, Pfau & Wilson, 2008b). The explicit validation of kinematics-based stride event detection against force plate data has been performed in only a few studies on the straight line: for horses, these included the use of accelerometers attached to the distal limb (Schamhardt & Merkens, 1994; Witte, Knill & Wilson, 2004) or optical motion capture data of the distal limb (Peham, Scheidl & Licka, 1999; Galisteo et al., 2010; Hobbs et al., 2010; Olsen, Haubro Andersen & Pfau, 2012; Boye et al., 2014). None of these algorithms has yet been tested during locomotion on the circle. Foot on events of the hind limbs have also been successfully approximated from pelvic movement in walking and trotting horses on straight lines and circles (Starke et al., 2012b). Some of these studies took inspiration from gait event detection in the human literature, which—albeit the different mechanics of the human foot compared to hoofed animals—provides a multitude of interesting algorithms (Hreljac & Marshall, 2000; Mickelborough et al., 2000; Hansen, Childress & Meier, 2002; Ghoussayni et al., 2004; O’Connor et al., 2007; Zeni Jr, Richards & Higginson, 2008; Miller, 2009; Kiss, 2010; Leitch et al., 2011). Kinematic footfall event detection has also been studied in cats (Pantall, Gregor & Prilutsky, 2012).

Both accelerometers and optical motion capture have proven valuable in determining footfall events. Accelerometer-based stride event detection proved very reliable: features of signals recorded using an accelerometer mounted to the dorsal hoof wall resulted in excellent accuracy and precision for foot on and foot off detection, accuracy ranging from 1.8 to 5.0 ms across walk, trot and canter (Witte, Knill & Wilson, 2004). However, this method may not translate easily to motion capture-based data acquisition, as it requires a hoof-based reference system (an accelerometer can be mounted in the desired orientation relative to the hoof) and double-differentiation of displacement data introduces noise (an accelerometer measures accelerations directly). Instead, optical motion capture-based stride event detection has focussed mainly on displacement and velocity characteristics of hoof-mounted markers, often with good results: horizontal velocity features of the toe have successfully been employed for foot on and foot off detection in horses (Peham, Scheidl & Licka, 1999; Boye et al., 2014) and several alternative algorithms have been proposed and tested (Peham, Scheidl & Licka, 1999; Galisteo et al., 2010; Boye et al., 2014). A pilot study investigating foot on detection from kinematics during jump landing on soft ground also found kinematic features likely suitable for reliable event detection (Hobbs et al., 2010). However, a recent comparison of multiple algorithms concluded that optimal algorithms vary between fore- and hind limbs and for walk and trot (Boye et al., 2014). This introduces analytical complexity and often requires a toe-mounted marker, which is vulnerable to damage or accidental removal.

While foot on events are typically characterised by a high collision, foot off events tend to be a continuous process. In hoofed animals, the structural properties of the hoof capsule prevent notable deformation during foot contact and foot off: the hoof wall, especially the proximal part, has a high Young’s Modulus/stiffness (Landeau, Barrett & Batterman, 1983; Leach & Zoerb, 1983; Douglas et al., 1996), while flaring of lateral and medial hoof walls (Douglas et al., 1996) and expansion of the heel region (Dyhre-Poulsen et al., 1994; Burn & Brockington, 2001) function as shock-absorbers. When moving on a hard surface, footfall events can therefore be approximated as rigid body collisions. For foot on, the simplest scenario is a hoof colliding non-elastically with the ground and the resultant velocity falling to zero to indicate stance. To detect this event, previous studies used the most frequently occurring horizontal velocity in a test set (Peham, Scheidl & Licka, 1999; Boye et al., 2014). Uncertainty about the exact foot on timing is however introduced by the brief period between initial contact when only part of the hoof touches the ground and full foot plant (Balch, Butler & Collier, 1997; Van Heel et al., 2004; Thomason & Peterson, 2008), hoof slip (Pardoe et al., 2001) or the deformation of soft ground on impact (Burn & Usmar, 2005; Chateau et al., 2010). In late stance, the hoof rolls forward over the toe during breakover (Back et al., 1995b; Back et al., 1995c; Witte, Knill & Wilson, 2004) as the point of force application moves forward towards the toe (Wilson et al., 1998). Breakover is the time between heel-off and toe-off and in the forelimb occupies approximately 20% of stance during trotting (Chateau, Degueurce & Denoix, 2006). During breakover, rotational movement of the hoof causes translation of those foot markers that are mounted away from the center of rotation. Therefore, a non-zero velocity of locations on the hoof does not necessarily correspond to foot off, confounding foot off detection not only in horses (Weyand et al., 2001). Motion capture markers placed close to the distal tip of dorsal hoof wall, respectively the toe tip (Boye et al., 2014) have the advantage of being in close proximity to the center of rotation at which zero velocity can be expected. Such distally placed markers have the disadvantages of being easily obscured or lost during movement on soft surfaces or being struck- or rubbed off in horses that over-track or toe-drag.

In this study, our overall aims and objectives were to develop a universal method across gaits and movement directions for the detection of foot on and foot off events using a single marker mounted on the proximal hoof wall. Specifically, we aimed to (1) validate, using method comparison metrics, two methods for foot on detection and two methods for foot off detection against kinetic gait events as well as (2) quantifying the robustness of kinetic events to different force thresholds ranging from 50 to 150 N. For foot on detection, we developed one threshold-based algorithm using resultant velocity and one event-based algorithm using distinct events in the acceleration signal. For foot off detection, we developed one threshold-based algorithm using rigid body trigonometry to calculate the threshold beyond which horizontal translation cannot be attributed to rotation and one event-based algorithm using distinct events in the vertical velocity signal.

## Materials and Methods

All procedures were performed under the approval of the Michigan State University Institutional Animal Care and Use Committee, protocol #06/11-112-00. All data analysis was performed in Matlab (The Mathsworks) using custom-written scripts created by Dr. Starke unless indicated otherwise. Scripts for automatic stride segmentation are available at https://uk.mathworks.com/matlabcentral/fileexchange/50093-segmentstrides.

### Data collection

**Figure 1:**
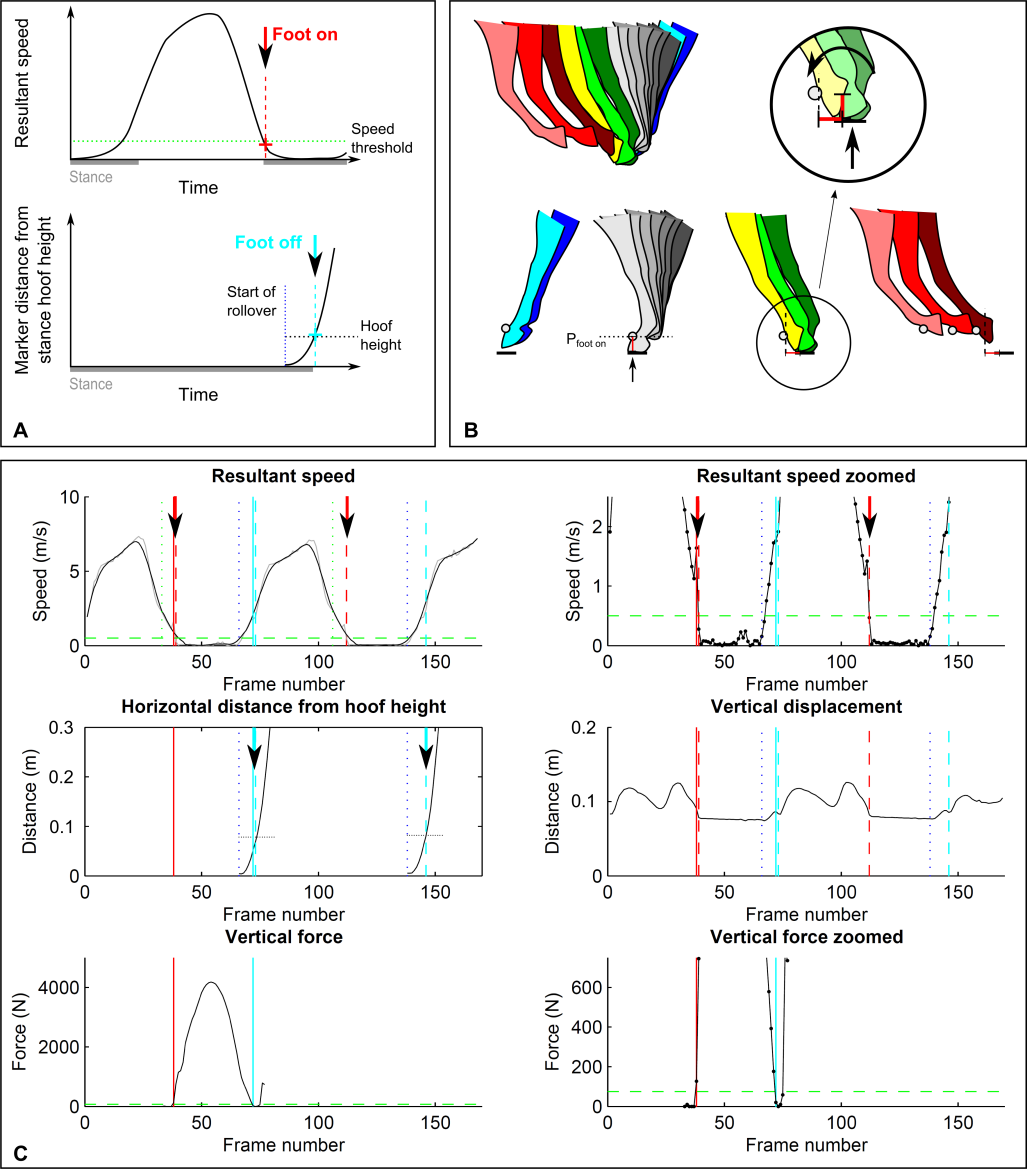
Threshold-based footfall detection method. (A) Principle for foot on (top, red) and foot off (bottom, cyan) detection based on a threshold in resultant speed for foot on and a threshold in distance travelled for foot off. (B) Detailed illustration of the foot-off detection approach: hoof height is extracted during stance; foot off then corresponds to the first frame at which the horizontal distance travelled by the marker exceeds the marker height in stance. (C) Kinematic (dashed lines) and kinetic (solid lines) event detection for foot on (red) and foot off (cyan) illustrated for a single stride of a forelimb during trot on a circle. Top row: resultant speed shown at two magnifications; middle row: distance measurements of horizontal distance travelled relative to the marker hoof height position during stance (left) as well as vertical displacement (right); bottom row: vertical ground reaction force shown at two magnifications. Arrows are shown in those data fields that are used to determine kinematic events. Grey: raw data (unfiltered) for resultant speed. Vertical displacement is given as a reference. Vertical blue dotted line: approximate end of stance. Horizontal green dashed line: 75 N force threshold.

Eight unshod Arabian horses (mean ± SD weight: 448 ± 19 kg; height at the withers: 149.6 ± 2.6 cm, height at the hip: 150.4 ± 2.8 cm) were equipped with retro-reflective markers attached to the proximal aspect of the dorsal hoof wall on each of the four feet (Fig. 1). Horses were visually assessed for lameness by Dr. Clayton and passed as moving within the margins of what is perceived ‘normal.’ Marker movement in 3D space was recorded at 100 Hz using an optical motion capture system (Motion Analysis Corporation, Santa Rosa, California, USA). The error in a linear measurement of 1,000 mm was <0.8 mm. Horses repeatedly walked and trotted in hand on a straight line and on the lunge on a 3 m radius circle, moving both clockwise (‘right rein’) and anti-clockwise (‘left rein’). This radius was chosen to correspond with the smallest diameter circle (volte) performed in dressage competitions as specified by the International Equestrian Federation. ^1^1FEI Dressage Rules 25th edition, 2015, pp 17; http://d2ig246cioy4di.cloudfront.net/cdn/farfuture/I2CO6xxmKgIeYOwyGCRxRZPkM6cf86PYiDyJOa5dnrA/mtime:1418911964/sites/default/files/DRE-Rules_2015_GA%20approved_black.pdf. For all conditions the ground was flat and hard with non-slip coating. On the straight line, horses crossed four force plates arranged linearly with their long axes parallel to the runway. The first and last plates measured 60 × 120 cm (FP61290; Bertec Corporation, Columbus, Ohio, USA) and the plates between them measured 60 × 90 cm (FP6090; Bertec Corporation, Columbus, Ohio, USA). On the circle, horses crossed the same force plate array and two laterally placed force plates (FP6090; Bertec Corporation, Columbus, Ohio, USA). The circle was drawn on the floor with chalk, and the handler ensured that the horse maintained the pre-defined radius across all trials. All force plates were mounted flush with the ground and recorded 3D force components at 1,000 Hz.

### Processing of kinematic data

3D displacement trajectories of all four feet were tracked semi-automatically in Cortex (version 1.1.4.368, Motion Analysis Corporation, Santa Rosa, California USA) and exported as .csv files for further processing in Matlab (The MathWorks, Natick, Massachusetts, USA). Trials were pre-selected in which horses moved steadily without marker dropouts for at least one stride. In Matlab, the three orthogonal displacement components [*x*, *y*, *z*] of the marker in a global reference system (based on the calibrated motion capture volume) were extracted for each trial and each foot for subsequent footfall event detection. Data for each trial were automatically pre-segmented into individual strides based on the frames at which the resultant velocity dropped below 2.5 m s^−1^ (see Figs. 1A and 1C); for this purpose, the resultant velocity was filtered with a moving average filter (‘smooth’ function in Matlab, window width = 10) to remove noise.

In the following, four algorithms for footfall detection are described. The ord er of reporting is based on the ‘functional’ approach to event detection: In Sections A and B, foot on and foot off are determined based on set thresholds, whereas in Sections C and D, foot on and foot off are determined based on distinct events in the data. This order remains the same throughout the manuscript. Foot on refers to touch-down of a foot, concurrent with the onset of limb loading. Foot off refers to lift-off of a foot, concurrent with the termination of the foot exerting a force on the ground. Both definitions are with reference to the ground reaction force exceeding 75 N (see kinetics section).

#### A. Foot on detection based on velocity thresholds

For the detection of ‘foot on’ events, displacement along each of the three coordinate system axes was differentiated to arrive at unfiltered velocity components. For each sample, the resultant velocity was then calculated as ${v}_{\mathrm{res}}=\sqrt{{v}_{x}^{2}+{v}_{y}^{2}+{v}_{z}^{2}}$. Foot on was identified as the first frame of this raw signal that fell below a specified velocity threshold (Figs. 1A and 1C). For each stride, this foot on event was automatically identified within a window (Fig. 1C) that spanned the pre-defined beginning of the stride (see above) plus 20 frames (0.2 s). The threshold was varied between 0.2 and 1.4 m s^−1^ to investigate sensitivity of method differences to thresholding (see ‘Results’ section). The final velocity threshold was selected as 0.5 m s^−1^ from the examined range based on lowest within—and across horse variation in method differences. To compensate for a bias introduced to the accuracy of event detection for limbs on the inside of the circle at trot (see ‘Results’ section), the threshold was adjusted for the inside forelimb on the circle at trot to 1.0 m s^−1^ and for the inside hind limb on the circle at trot to 1.2 m s^−1^.

#### B. Foot off detection based on trigonometry thresholds

For the detection of ‘foot off’ events, we approximated the breakover process using rigid body assumptions to determine the point at which movement is no longer explicable by rotation. We hypothesised that foot off can be approximated based on the limitations imposed on hoof translation while the toe maintains contact with the ground: horizontal translation of locations on the hoof is limited by hoof size, and there is a threshold beyond which horizontal translation cannot be attributed to rotation (Fig. 1B). To test this approach, we estimated foot off events using the following workflow: first, the 3D position of the hoof at the frame identified as foot on was extracted from the motion capture data (point *P*_foot on_) to gain hoof height; second, the smoothed resultant velocity signal (as above, window width = 10) was used to determine the beginning of breakover using the end of the plateau of near-zero velocity following foot-on events (corresponding to stance, Figs. 1A and 1B); third, the magnitude of horizontal foot displacement from this point onwards was calculated as the Euclidian distance (Figs. 1B and 1C) between *P*_foot on_[*x*, *z*] and the [*x*, *z*] marker position in each frame within the possible lift-off window of 15 frames (0.15 s). Foot off was then identified as the last frame at which the distance travelled in the horizontal plane away from the location of the hoof at *P*_foot on_ was smaller than the height of the hoof marker at point *P*_foot on_ (Figs. 1A and 1B). If a trial started during stance without the initial foot on event, the 3D position of the hoof at the determined end-of-stance point was extracted for an instantaneous hoof height value.

#### C. Foot on detection based on acceleration events

**Figure 2:**
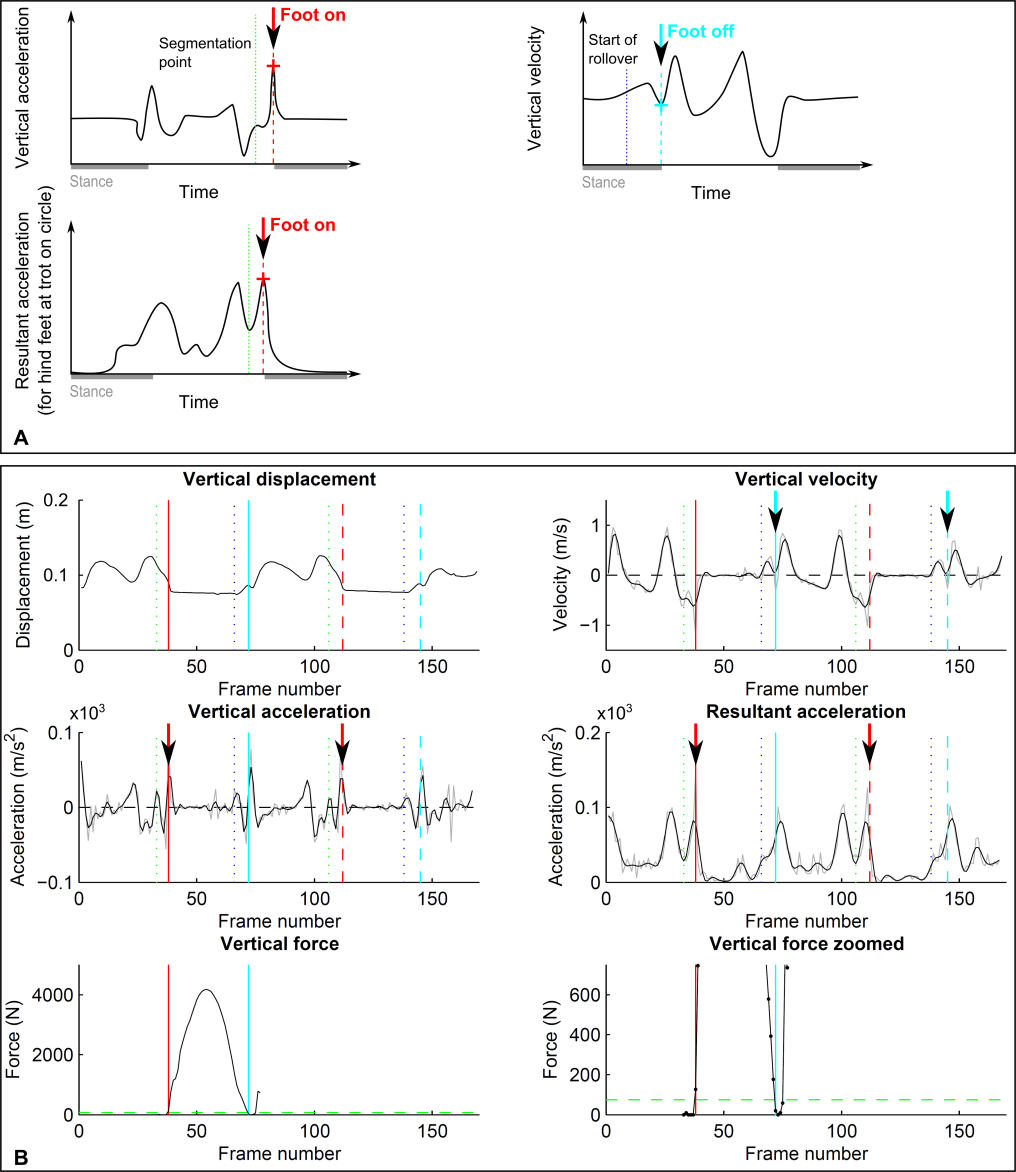
Event-based footfall detection method. (A) Principle for foot on (left, red) and foot off (right, cyan) detection based on distinct events in the acceleration signal for foot on and the vertical velocity signal for foot off. For foot on, generally vertical acceleration was used. Only for the hind limbs during trot on the circle did we use resultant acceleration, as for individual horses there was no distinct event in vertical acceleration. (B) Kinematic (dashed lines) and kinetic (solid lines) event detection for foot on (red) and foot off (cyan) illustrated for a single stride of a forelimb during trot on a circle, the same stride as shown in Fig. 1. Top row: vertical displacement (left) and velocity (right); middle row: vertical acceleration (left) and resultant acceleration (right); bottom row: vertical ground reaction force shown at two magnifications. For further details, refer to Fig. 1.

This foot on detection method was examined to test whether differentiation of displacement data would allow detection of footfall events similar to hoof-mounted accelerometers (Witte, Knill & Wilson, 2004) despite the introduced noise and a different co-ordinate reference system. For the detection of ‘foot on’ events, displacement along each of the three coordinate system axes was double-differentiated to arrive at unfiltered acceleration components. We then tested a variety of low-pass and raw event detection approaches using both vertical acceleration and the resultant acceleration to determine the best settings for impact detection. These tests showed best accuracy and precision (both between and within horses) for events based on vertical acceleration (compare Fig. 2), except for the hind feet during trot on the circle. Here, impact accelerations were often not detectable; however, resultant acceleration proved reliable. The final algorithm we tested is hence a composite: for all conditions except the hind feet during trot on the circle, vertical acceleration was low-pass filtered (4th order, zero-lag Butterworth filter, cut-off frequency 25 Hz for trot and 20 Hz for walk). Foot on (Figs. 2A and 2C) was then identified as the maximum between the pre-segmentation point (see above) and a further 20 frames (0.2 s). For the hind feet during trot on the circle, the same procedure was performed, but in this case the time of foot on was based on the resultant acceleration which was low-pass filtered with a 4th order, zero-lag Butterworth filter with a cut-off frequency of 15 Hz (Figs. 2A and 2C).

#### D. Foot off detection based on velocity events

After initial tests we chose to work with the velocity signal to detect foot-off events based on the assumption that at the end of breakover vertical velocity would change direction from downwards to upwards as the foot is lifted to achieve ground clearance and to avoid toe-dragging (Figs. 2B and 2C). Vertical velocity was hence derived by differentiating the vertical displacement signal followed by low-pass filtering (4th order, zero-lag Butterworth filter, cut-off frequency 15 Hz for walk and trot). Foot off (Figs. 2B and 2C) was then identified as the first minimum following the approximated beginning of breakover (compare Section B). This minimum was identified by first finding a preliminary minimum in the filtered signal and then finding the exact minimum in the raw velocity signal within the frames surrounding the preliminary minimum.

### Processing of kinetic data

Forces were pre-processed to determine eligible footfall events as part of a different study: a virtual hoof imprint was specified using four markers rigidly attached to the hoof as the hoof approached the ground. If the complete virtual imprint of a hoof fell within the plate area, the point of force application was calculated from the 3D forceplate data and compared to the area covered by the virtual hoof imprint. If the point of force application fell within the virtual foot imprint, the stride was retained for further processing if kinematic data were available.

To determine the kinetic footfall events for all retained strides, raw force data (unfiltered) were processed: force data associated with each file used for kinematic analysis were extracted from the .c3d files using the b-tk toolkit for Matlab (Barre & Armand, 2014). In brief, data for the analogue channels of the data acquisition corresponding to forces in [*x*, *y*, *z*] direction were extracted and offset and scaling factors associated with the acquisition as well as gravity applied. For each trial, each force component for each plate was then offset-corrected by subtracting the average force calculated over a 200 frame window (0.2 s at 1,000 Hz) at the beginning of the trial or the end of the trial in case the beginning of the trial held actual loading data. Data were then downsampled to 100 Hz by retaining every 10th frame of the datastream. Vertical force was used to determine footfall contacts, as calculating the resultant force raised the noise floor. For the method comparison of kinematic stride events, foot on was identified as the first frame in which the vertical force exceeded 75 N. Foot off was identified as the first frame in which the vertical force dropped below 75 N.

### Method comparison

To compare between kinematic and kinetic stride event detection, method differences were calculated by subtracting the kinematics based value (*p*_mocap_) from the kinetics based value (*p*_force plates_) as method difference =*p*_force plates_ − *p*_mocap_. Four strides were quarantined from analysis due to unexplained inconsistencies such as a ‘blip’ in the data (see Supplemental Information 1, part 1) which resulted in substantial outliers.

Limits of Agreement (LoA) were calculated across all pooled strides for each condition as the mean ± 2 SD of all individual method differences, covering 95% of events across all individual strides.

Accuracy and precision (Bland & Altman, 1986) were calculated from horse-based mean values of the method differences as follows: for each horse, method difference mean and SD were calculated across limb pairs (fore or hind) for each condition and each of the four event types (fore on, hind on, fore off, hind off). For locomotion on the circle, these pairs consisted of limbs in identical loading conditions; for example, data for the outside limb were calculated across the left limb on the right rein (clockwise) and the right limb on the left rein (anti-clockwise). Across these horse-based values, accuracy (overall mean across horse-based mean values), precision across horses (SD across horse-based mean values) as well as precision within horses (average horse-based SD) were calculated. These three metrics were chosen to quantify bias (average difference from kinetic event) as well as the precision (variability) of kinematic event detection both across and within horses.

All statistical tests were carried out in IBM SPSS Statistics 20, unless indicated otherwise.

To determine the effect of the six conditions on accuracy estimates for fore- and hind limbs, the eight horse-based mean values for each condition were tested for normality using the Shapiro–Wilk test and then compared using a repeated measures design. If datasets did not deviate significantly from normal distribution, a repeated measures ANOVA was performed. In case of significance, a Sidak-corrected test for the main effect was executed post hoc. If datasets did deviate significantly from normal distribution, a Friedman test was performed. In case of significance, pairwise Wilcoxon tests were performed post hoc, with the significance level corrected for multiple testing as *α* divided by the number of tests (*α* = 0.05/15 = 0.0033).

To determine whether accuracy estimates varied between fore- and hind limbs within each individual condition, pairwise tests were performed as follows: if the two contrasted datasets did not deviate significantly from normal distribution, a pairwise *t*-test was performed, corrected for multiple testing as described above (*α* = 0.05/6 = 0.0083). If the two contrasted datasets did deviate significantly from normal distribution, a pairwise Wilcoxon test was performed, corrected to the same significance level.

### Introduced time shifts in stance and swing

To examine the impact of the proposed kinematic event detection method on the accuracy of derived stance and swing durations, the time shift between kinematic foot on and foot off events was calculated. For this purpose, the difference between accuracy for foot on and foot off detection was calculated for the forequarters and hindquarters of each horse and each condition by subtracting the mean accuracy for foot off from the mean accuracy for foot on: a value of zero would indicate that derived stance and swing times would on average be unbiased, respectively identical to those calculated from kinetics, despite a possible offset between kinematic and kinetic events. The offset was calculated both in ms and in percentage stride by dividing the offset by the average stride duration of each horse in each condition. Stride duration was calculated from successive foot on events of the left hind (LH) foot determined from kinematic event detection. In addition, stance times were calculated from the kinetic dataset for fore- and hind feet separately. All results were then averaged across horses.

### Effect of different force thresholds

To test the effect of threshold values on kinetic footfall timings, the force threshold was varied between 50 N and 150 N in 10 N increments and the above identification of foot on and foot off repeated (see ‘Processing of kinetic data’). Changes in timings were expressed as the difference to timings based on a 50 N threshold. Across all pooled strides for each condition, the average time shift introduced by different force thresholds were calculated and histograms created.

## Results

A total of 3,074 footfall events (1,559 foot on events, 1,515 foot off events) were processed across horses, gaits and movement directions. Between 5 and 35 strides were retained for each horse per condition for each of the four event types (fore on, hind on, fore off, hind off), the average number of strides ranging from 11 to 25 per horse. This resulted in between 90 and 200 strides for each condition and event type. An overview of basic kinematic parameters is given in Table 1.

**Table 1:** General information on the six conditions. Mean (SD) values for stride duration (based on kinematics) and stance duration (based on kinetic events) for all six conditions. Stride duration for inside and outside limb on the circle for the same gait is calculated across all strides on the circle and hence identical.

Direction	Gait	Stride duration (in ms)	Stance duration (in ms)	
			Fore	Hind
Straight	Walk	1,153 (55)	734 (41)	722 (39)
Straight	Trot	722 (31)	328 (24)	283 (11)
Circle, I	Walk	1,189 (64)	772 (48)	769 (41)
Circle, O	Walk	1,189 (64)	764 (59)	742 (45)
Circle, I	Trot	783 (26)	394 (25)	345 (15)
Circle, O	Trot	783 (26)	373 (25)	310 (13)

### Method comparison

#### A. Foot on detection based on velocity thresholds

**Figure 3:**
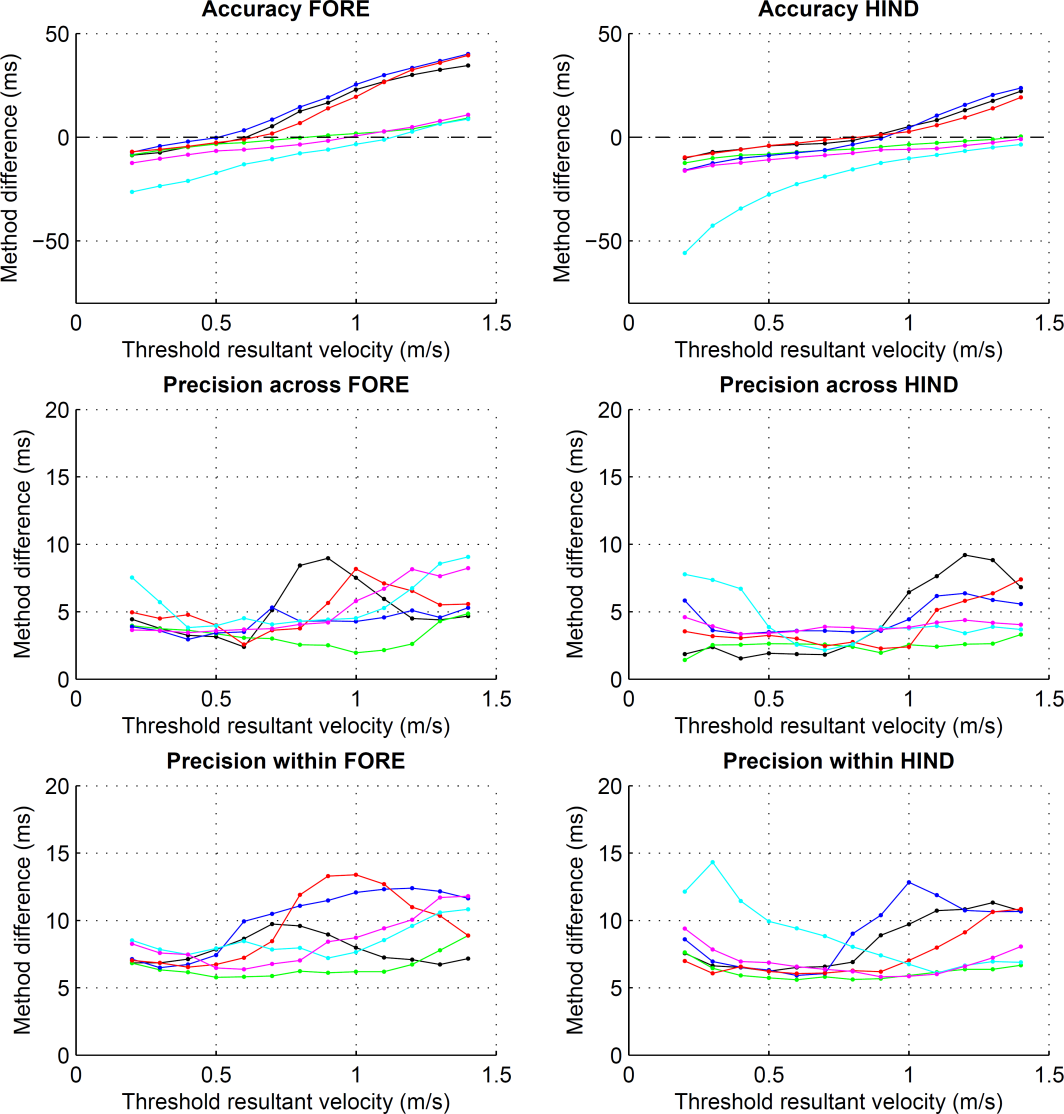
Preliminary assessment of the effect of different resultant velocity thresholds for the detection of foot on. Accuracy and precision, both within and across horses, for foot on detection using resultant velocity thresholds ranging from 0.2 to 1.4 m s^−1^. Values are calculated across each of the eight horse-based means for the six conditions: straight walk (black), straight trot (green), walk on the circle with the foot on the inside (blue) and outside (red), trot on the circle with the foot on the inside (cyan) and outside (magenta). Note the different scales for accuracy and precision.

Different velocity thresholds affected both accuracy and precision estimates (Fig. 3). There was a bias in both accuracy and precision estimates for fore- and hind limbs on the inside of the circle at trot (see Fig. 3, cyan), accuracy differing from the remaining conditions by up to 20 ms (fore) and up to 40 ms (hind). By setting the final velocity threshold to 1.0 m s^−1^ (fore) and 1.2 m s^−1^ (hind) for these conditions as described in the methods section, accuracy and precision estimates approached values of the remaining conditions.

**Figure 4:**
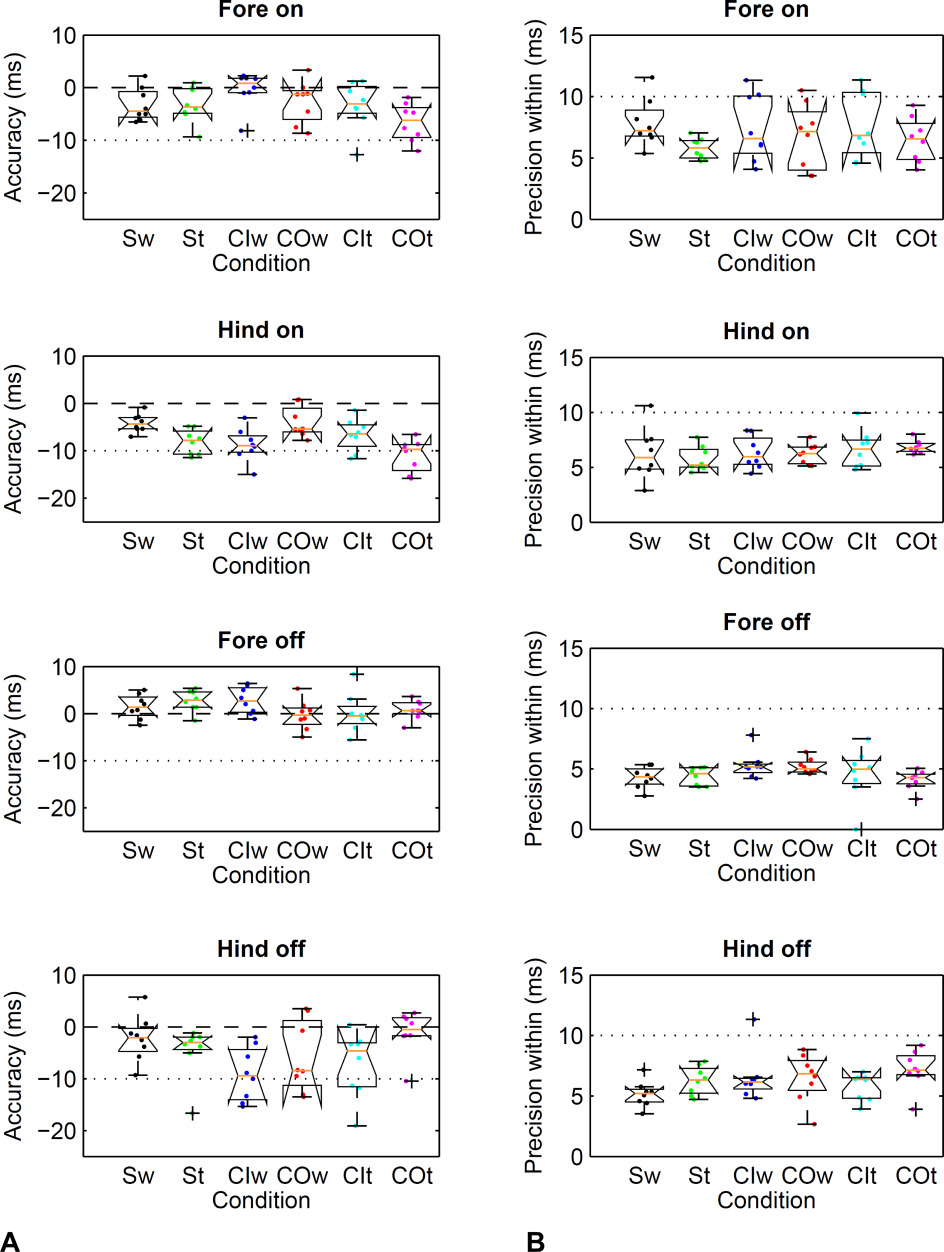
Method comparison using the threshold-based kinematic footfall event detection methods (Sections A and B) compared to kinetic footfall events. Boxplots show accuracy (A) and within-horse precision (B) across the eight horse-based mean values (dots). Dashed line (- - -): zero difference between kinematic and kinetic method. Dotted line (…  …): a single frame of the kinematic datastream, equivalent to 10 ms at the 100 Hz sampling rate. Condition codes: Sw, straight walk (black); St, straight trot (green); CIw, walk on the circle with the foot on the inside (blue); COw, walk on the circle with the foot on the outside (red); CIt, trot on the circle with the foot on the inside (cyan); COt, trot on the circle with the foot on the outside (magenta).

Limits of agreement ranged from −26 to 17 ms (Table 2) depending on limb, gait and direction. Accordingly, accuracy showed a bias of −11 to 0 ms depending on limb, gait and direction (Table 3). Precision across and within horses ranged from 2 to 8 ms (Table 3). Horse-based means for accuracy and within-horse precision are shown in Fig. 4.

**Table 2:** Limits of agreement: threshold-based method. Limits of agreement (lower/upper bound) were calculated across all pooled 97–200 strides per condition as the mean ± 2 SD. Foot on events were detected by the resultant hoof velocity dropping below 0.5 m s^−1^, except for the inside forelimb on the circle at trot (1.0 m s^−1^) and for the inside hind limb on the circle at trot (1.2 m s^−1^) to compensate for method bias. Foot off events were detected by horizontal marker translation moving further than the hoof height during stance and hence further than explainable by hoof rotation. Circle, I—limb on the inside of the circle; Circle, O—limb on the outside of the circle.

Direction	Gait	Foot on (in ms)		Foot off (in ms)	
		Fore	Hind	Fore	Hind
Straight	Walk	−20/12 (*N* = 90)	−17/8 (*N* = 99)	−8/10 (*N* = 93)	−16/10 (*N* = 98)
Straight	Trot	−15/10 (*N* = 152)	−20/4 (*N* = 200)	−7/13 (*N* = 156)	−20/11 (*N* = 185)
Circle, I	Walk	−17/17 (*N* = 104)	−22/6 (*N* = 103)	−10/14 (*N* = 105)	−26/6 (*N* = 96)
Circle, O	Walk	−20/15 (*N* = 114)	−18/9 (*N* = 132)	−11/12 (*N* = 116)	−25/13 (*N* = 128)
Circle, I	Trot	−20/15 (*N* = 107)	−20/8 (*N* = 134)	−12/11 (*N* = 118)	−23/10 (*N* = 112)
Circle, O	Trot	−21/8 (*N* = 157)	−26/4 (*N* = 167)	−8/9 (*N* = 164)	−18/15 (*N* = 144)

**Table 3:** Accuracy and precision of gait event detection: threshold-based method. Accuracy (mean of differences) and precision across horses (SD of differences) were calculated as the average across the eight horse-specific mean values. Precision within horses was calculated as the average across the eight horse-specific SD values. For details on kinematic event detection and abbreviations, please refer to Table 2. a, b, c—Conditions with the same superscript letter are significantly different from each other.

Direction	Gait	Accuracy (in ms)				Precision across horse means (in ms)				Average precision within horses (in ms)			
		Foot on		Foot off		Foot on		Foot off		Foot on		Foot off	
		Fore	Hind	Fore	Hind	Fore	Hind	Fore	Hind	Fore	Hind	Fore	Hind
Straight	Walk	−3	−4^a^	1	−2	3	2	3	4	8	6	4	5
Straight	Trot	−3	−8	3	−5	3	3	2	5	6	6	4	6
Circle, I	Walk	0	−9	3	−9	3	4	3	5	7	6	5	7
Circle, O	Walk	−3	−4^b^	0	−6	4	3	3	7	7	6	5	7
Circle, I	Trot	−3	−7^c^	0	−7	5	3	4	6	8	7	5	6
Circle, O	Trot	−7	−11 ^a,b,c^	1	−1	4	3	2	4	7	7	4	7

The movement condition had no significant effect on accuracy estimates for the forefeet (Friedman Test, *P* ≥ 0.093, *α* = 0.05). For the hind feet, a repeated measures ANOVA revealed a significant effect of the condition on accuracy (*P* < 0.001, *α* = 0.05). Significant pairwise differences were detected between selected conditions (Table 3), differences between means ranging from 7 to 4 ms. Paired tests between fore- and hind limbs within each condition revealed significant differences in accuracy for trot on the straight line (*P* < 0.001, *α* = 0.0083; mean (SD) difference 5 (2) ms) but not for the other conditions (*P* ≥ 0.012, *α* = 0.0083).

#### B. Foot off detection based on trigonometry thresholds

Limits of agreement ranged from −26 to 15 ms (Table 2) depending on limb, gait and direction. Accordingly, accuracy showed a bias of −9 to 3 ms depending on limb, gait and direction (Table 3). Precision across and within horses ranged from 2 to 7 ms (Table 3). Horse-based means for accuracy and within-horse precision are shown in Fig. 4.

The movement condition had no significant effect on accuracy estimates for the forefeet (repeated measures ANOVA, *P* ≥ 0.067, *α* = 0.05). For the hind feet, a Friedman test revealed a significant effect of the condition on accuracy (*P* = 0.003, *α* = 0.05). However, post-hoc Wilcoxon matched-pairs signed-ranks tests were not able to detect significant differences after correction for multiple testing (*P* ≥ 0.012, *α* = 0.0033). Paired tests between fore- and hind limbs within each condition revealed significant differences in accuracy for walk on the circle with the limb on the inside (*P* < 0.001, *α* = 0.0083; mean (SD) difference 12 (6) ms) but not the other conditions (*P* ≥ 0.011, *α* = 0.0083).

#### C. Foot on detection based on acceleration events

**Figure 5:**
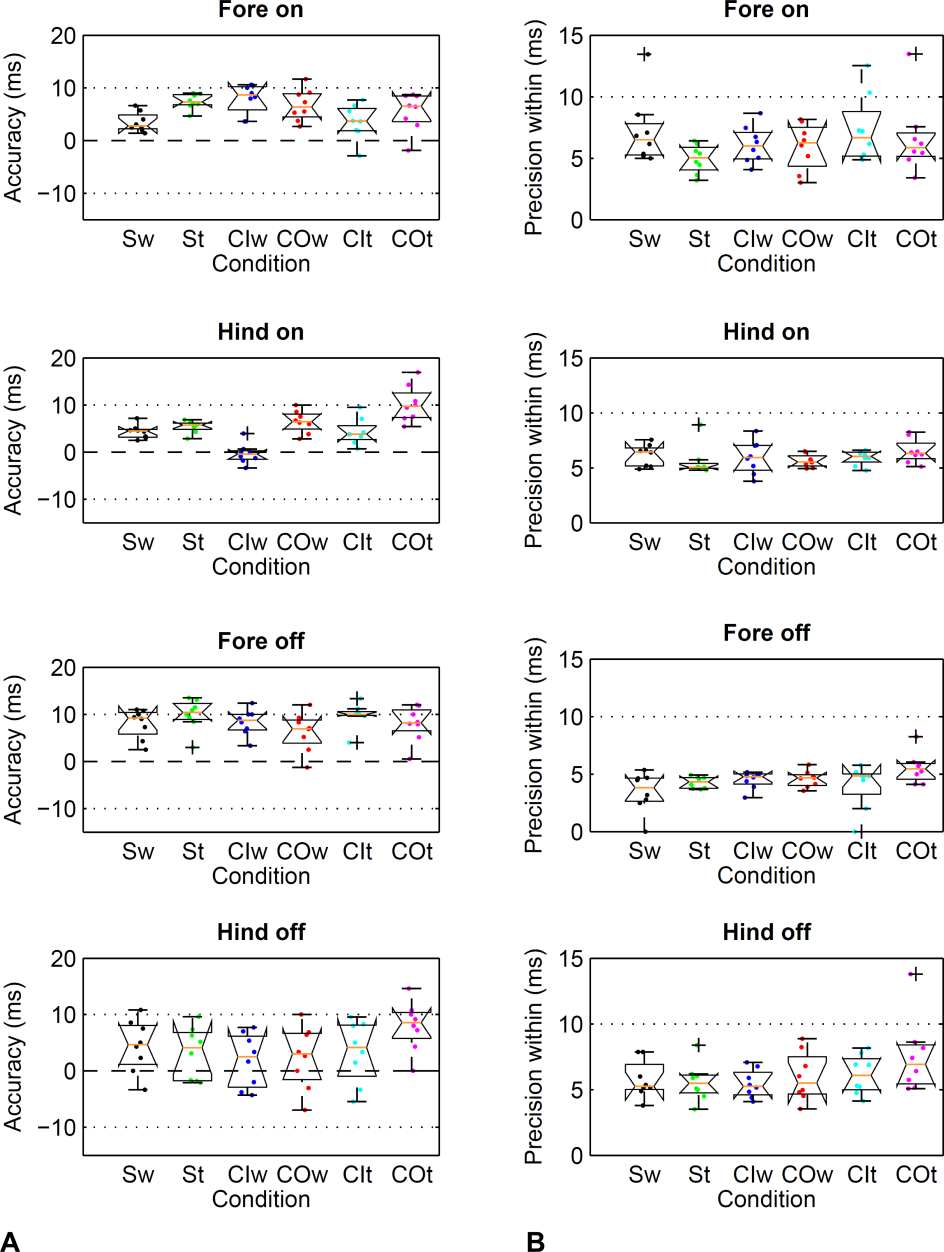
Method comparison using the event-based kinematic footfall event detection methods (Sections C and D) compared to kinetic footfall events. Boxplots show accuracy (A) and within-horse precision (B) across the eight horse-based mean values (dots). For details, please refer to Fig. 4.

Limits of agreement ranged from −12 to 25 ms (Table 4) depending on limb, gait and direction. Accordingly, accuracy showed a bias of 0 to 10 ms depending on limb, gait and direction (Table 5). Precision across and within horses ranged from 1 to 7 ms (Table 5). Horse-based means for accuracy and within-horse precision are shown in Fig. 5.

**Table 4:** Limits of agreement: event-based method. Limits of agreement (lower/upper bound) calculated as described in Table 2. Foot on events were detected by distinct events in the vertical acceleration trajectory, except for the hind limbs on the circle at trot where resultant acceleration was used. Foot off events were detected by distinct events in the vertical velocity trajectory. For details on abbreviations, please refer to Table 2.

Direction	Gait	Foot on (in ms)		Foot off (in ms)	
		Fore	Hind	Fore	Hind
Straight	Walk	−11/18 (*N* = 90)	−8/16 (*N* = 99)	0/18 (*N* = 93)	−10/19 (*N* = 98)
Straight	Trot	−2/18 (*N* = 152)	−6/16 (*N* = 200)	−1/21 (*N* = 156)	−10/18 (*N* = 185)
Circle, I	Walk	−5/22 (*N* = 104)	−12/13 (*N* = 103)	−1/19 (*N* = 105)	−13/16 (*N* = 96)
Circle, O	Walk	−7/21 (*N* = 114)	−6/18 (*N* = 132)	−5/18 (*N* = 116)	−15/19 (*N* = 128)
Circle, I	Trot	−11/19 (*N* = 107)	−8/17 (*N* = 134)	1/20 (*N* = 118)	−12/20 (*N* = 112)
Circle, O	Trot	−10/21 (*N* = 157)	−5/25 (*N* = 167)	−5/21 (*N* = 164)	−11/26 (*N* = 144)

**Table 5:** Accuracy and precision of gait event detection: event-based method. Accuracy and precision calculated as described in Table 4. For details on kinematic event detection and abbreviations, please refer to Tables 2 and 3. a, b, c, d, e—Conditions with the same superscript letter are significantly different from each other.

Direction	Gait	Accuracy (in ms)				Precision across horse means (in ms)				Average precision within horses (in ms)			
		Foot on		Foot off		Foot on		Foot off		Foot on		Foot off	
		Fore	Hind	Fore	Hind	Fore	Hind	Fore	Hind	Fore	Hind	Fore	Hind
Straight	Walk	4	4^a^	8	4	2	1	3	5	7	6	3	6
Straight	Trot	7	5^b^	10	3^a^	1	1	3	5	5	6	4	6
Circle, I	Walk	8	0^a,b,c,d^	8	2	3	2	3	5	6	6	5	5
Circle, O	Walk	7	7^c^	6	2	3	2	4	6	6	6	5	6
Circle, I	Trot	4	4^e^	10	3	3	3	3	6	7	6	4	6
Circle, O	Trot	6	10^d,e^	8	8^a^	4	4	4	4	7	7	6	8

The movement condition had an effect on accuracy estimates for fore- and hind feet. For the fore feet, a Friedman test revealed a significant effect of the condition on accuracy (*P* = 0.021, *α* = 0.05). However, post-hoc Wilcoxon matched-pairs signed-ranks tests were not able to detect significant differences after correction for multiple testing (*P* ≥ 0.017, *α* = 0.0033). For the hind feet, a repeated measures ANOVA revealed a significant effect of the condition on accuracy (*P* < 0.001, *α* = 0.05). Significant pairwise differences were detected between selected conditions (Table 5), differences between means ranging from 4 to 7 ms. Paired tests between fore- and hind limbs within each condition revealed no significant differences in accuracy (*P* ≥ 0.011, *α* = 0.0083).

#### D. Foot off detection based on velocity events

Limits of agreement ranged from −15 to 26 ms (Table 4) depending on limb, gait and direction. Accordingly, accuracy showed a bias of 2 to 10 ms depending on limb, gait and direction (Table 5). Precision across and within horses ranged from 3 to 6 ms (Table 5). Horse-based means for accuracy and within-horse precision are shown in Fig. 5.

The movement condition had an effect on accuracy estimates for fore- and hind feet. For the fore feet, a Friedman test revealed a significant effect of the condition on accuracy (*P* = 0.004, *α* = 0.05). However, post-hoc Wilcoxon matched-pairs signed-ranks tests were not able to detect significant differences after correction for multiple testing (*P* ≥ 0.012, *α* = 0.0033). For the hind feet, a repeated measures ANOVA revealed a significant effect of the condition on accuracy (*P* = 0.016, *α* = 0.05). Significant pairwise differences were detected between two conditions only (Table 5) with a difference between means of 5 ms. Paired tests between fore- and hind limbs within each condition revealed significant differences in accuracy for trot on the straight and walk on the circle with the limb on the inside (*P* < 0.008, *α* = 0.0083; mean (SD) difference up to 7 (4) ms) but not the other conditions (*P* ≥ 0.018, *α* = 0.0083).

### Introduced time shifts

For the threshold-based method (Sections A and B), the inaccuracy of event detection resulted in an average temporal shift between foot on and off events by 2 to 7 ms for forelimb event detection and −2 to 10 ms for hind limb event detection across gaits and directions (Table 6). This corresponded to on average 0.2 to 1.0% of stride duration for forelimb event detection and −0.1 to 1.3% of stride duration for hind limb event detection.

**Table 6:** Introduced shift between time points. The shift between average kinematic foot on and foot off event introduced by the threshold based (left) and event-based (right) approach, both compared to the kinetic method.

Direction	Gait	Threshold-based method				Event-based method			
		Offset (in ms)		Offset (in % stride)		Offset (in ms)		Offset (in % stride)	
		Fore	Hind	Fore	Hind	Fore	Hind	Fore	Hind
Straight	Walk	5 (3)	2 (5)	0.4 (0.3)	0.2 (0.4)	5 (4)	0 (5)	0.4 (0.4)	0.0 (0.4)
Straight	Trot	6 (4)	4 (7)	0.8 (0.6)	0.5 (0.9)	3 (4)	−2 (5)	0.4 (0.5)	−0.3 (0.7)
Circle, I	Walk	3 (6)	0 (7)	0.3 (0.5)	0.0 (0.6)	0 (3)	2 (6)	0.0 (0.3)	0.2 (0.5)
Circle, O	Walk	2 (4)	−2 (8)	0.2 (0.4)	−0.1 (0.7)	−1 (6)	−4 (6)	0.0 (0.5)	−0.3 (0.5)
Circle, I	Trot	4 (7)	−1 (8)	0.5 (0.9)	−0.1 (1.1)	6 (4)	−1 (5)	0.8 (0.5)	−0.1 (0.7)
Circle, O	Trot	7 (5)	10 (6)	1.0 (0.6)	1.3 (0.7)	2 (4)	−2 (4)	0.3 (0.6)	−0.3 (0.5)

For the event-based method (Sections C and D), the inaccuracy of event detection resulted in an average temporal shift between foot on and off events by −1 to 6 ms for forelimb event detection and −4 to 2 ms for hind limb event detection across gaits and directions (Table 6). Onaverage this corresponded to 0.0 to 0.8% of stride duration for forelimb event detection and −0.3 to 0.2% of stride duration for hind limb event detection.

### Effect of different force thresholds

**Figure 6:**
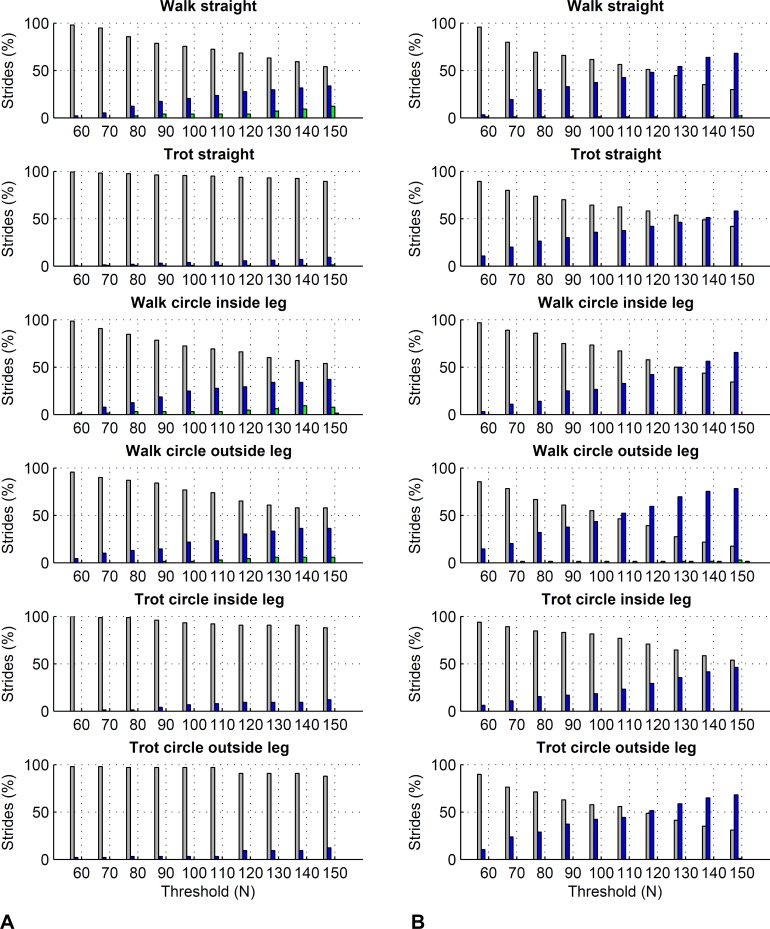
Effect of different force thresholds on kinetic event timings: forelimbs. Histograms across all pooled strides for the difference compared to events based on a 50 N threshold depending on vertical force thresholds ranging from 60 to 150 N. Grey: no difference in timing, blue: 10 ms (1 frame) difference in timing, green: 20 ms (2 frames) difference, red: 30 ms (3 frames) difference, black: 40 ms (4 frames) difference. (A) foot on detection as the first frame exceeding the threshold. The greater the difference, the later the kinetic event occurs compared to the foot on event based on a 50 N threshold. (B) foot off detection as the first frame falling below the threshold. The greater the difference, the earlier the kinetic event occurs compared to the foot off event based on a 50 N threshold.

**Figure 7:**
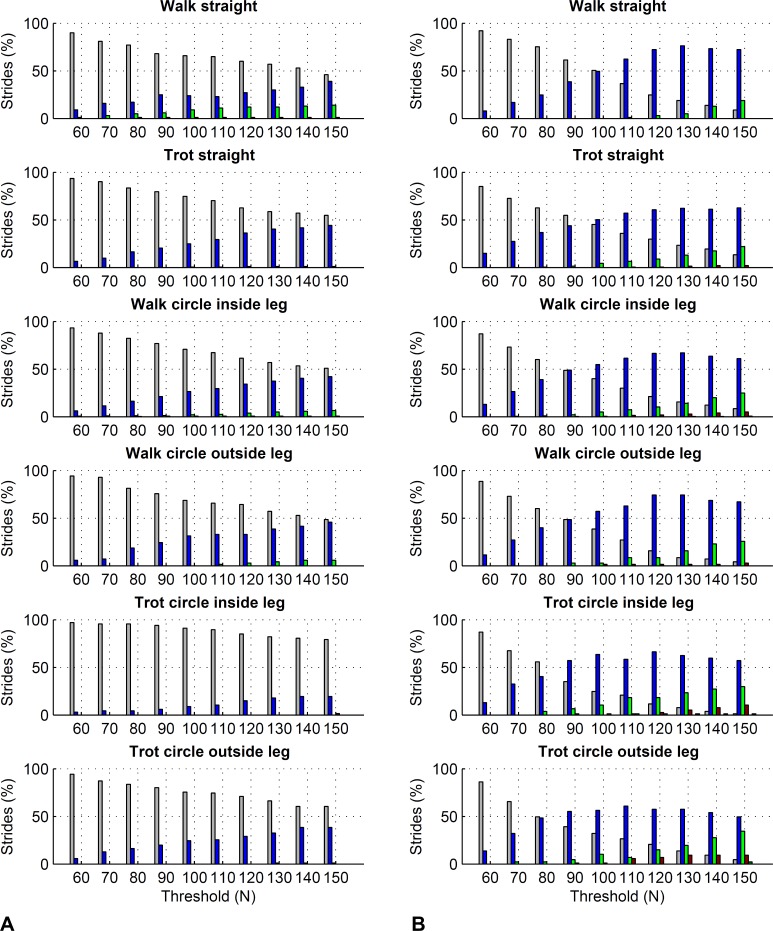
Effect of different force thresholds on kinetic event timings: hind limbs. Histograms across all pooled strides for the difference compared to events based on a 50 N threshold depending on vertical force thresholds ranging from 60 to 150 N. For details, please refer to Fig. 6.

For the forelimbs, varying the force threshold between 50 and 150 N resulted in a maximum average difference in kinetic foot timing of 6 ms for foot on detection and −7 ms for foot off detection (Table 7). For the hind limbs, these differences were 7 ms for foot on detection and −16 ms for foot off detection (Table 8). Maximum deviations for individual strides ranged from 0 to 30 ms for foot on detection and 0 to 50 ms for foot off detection. Histograms are shown in Figs. 6 and 7.

**Table 7:** Effect of force threshold on kinetic stride timings: forelimbs. Average difference compared to event based on a 50 N force threshold calculated across all pooled strides. Negative values indicate that the event was detected earlier than the event based on the 50 N threshold. Force thresholds have a greater impact on foot off timings than foot on timings. Condition codes: Sw, straight walk; St, straight trot; CIw, walk on the circle with the foot on the inside; COw, walk on the circle with the foot on the outside; CIt, trot on the circle with the foot on the inside; COt, trot on the circle with the foot on the outside.

Threshold	Average difference in foot on timing compared to 50 N threshold (in ms)						Average difference in foot off timing compared to 50 N threshold (in ms)					
	Sw	St	CIw	COw	CIt	COt	Sw	St	CIw	COw	CIt	COt
60 N	0	0	0	0	0	0	−1	−1	0	−1	−1	−1
70 N	1	0	1	1	0	0	−2	−2	−1	−3	−1	−2
80 N	2	0	2	1	0	0	−3	−3	−1	−4	−2	−3
90 N	3	0	2	2	0	0	−4	−3	−3	−4	−2	−4
100 N	3	1	3	2	1	0	−4	−4	−3	−5	−2	−4
110 N	3	1	3	3	1	0	−4	−4	−3	−6	−2	−4
120 N	4	1	4	4	1	1	−5	−4	−4	−7	−3	−5
130 N	4	1	5	4	1	1	−6	−5	−5	−8	−4	−6
140 N	5	1	5	5	1	1	−7	−5	−6	−8	−4	−6
150 N	6	1	6	5	1	1	−7	−6	−7	−9	−5	−7

**Table 8:** Effect of force threshold on kinetic stride timings: hind limbs. Average difference compared to event based on a 50 N force threshold calculated across all pooled strides. For details, please refer to Table 7.

Threshold	Average difference in foot on timing compared to 50 N threshold (in ms)						Average difference in foot off timing compared to 50 N threshold (in ms)					
	Sw	St	CIw	COw	CIt	COt	Sw	St	CIw	COw	CIt	COt
60 N	1	1	1	1	0	1	−1	−1	−1	−1	−1	−1
70 N	2	1	1	1	0	1	−2	−3	−3	−3	−3	−4
80 N	3	2	2	2	0	2	−2	−4	−4	−4	−5	−5
90 N	4	2	3	2	1	2	−4	−5	−5	−5	−7	−7
100 N	5	3	3	3	1	2	−5	−6	−7	−7	−9	−8
110 N	5	3	4	4	1	3	−6	−7	−8	−8	−10	−9
120 N	5	4	4	4	1	3	−8	−8	−9	−10	−12	−11
130 N	6	4	5	5	2	3	−9	−9	−11	−11	−13	−12
140 N	6	4	5	5	2	4	−10	−10	−12	−12	−14	−14
150 N	7	5	6	6	2	4	−11	−11	−13	−13	−15	−16

## Discussion

### Summary

This study investigated two universal approaches for foot on and foot off detection derived from optical motion capture data of a marker on the proximal hoof, one based on thresholds and the other based on events. The two approaches resulted in similarly good performance with no notable effects of conditions on accuracy or precision estimates (compare Figs. 4 and 5 and Tables 4 and 5). Limits of agreement suggest that 95% of detected foot on and foot off events will differ by no more than 26 ms from the kinetic event, with average bias values being close to zero. Both average accuracy and precision fell within approximately one frame of the kinetic event at a sampling rate of 100 Hz. We do not expect substantial deviations from the values reported here if gaits are performed at slower or higher speed: previous work showed that method differences are robust to such changes for trot both on the straight line and circle (Peham, Scheidl & Licka, 1999; Starke et al., 2012b). While we were not able to test the method at canter or gallop due to health and safety concerns, we anticipate the algorithm to work equally well, as the principles of impact and lift-off mechanics are unlikely to change substantially. One encouraging finding in this respect comes from a study that observed no significant difference between foot on detection for humans performing running and stepping down tasks with very different impact kinetics (Hobbs et al., 2010). Based on the conditions under which the study was performed, the event-based method may be less likely to suffer from scaling effects when examining horses of different sizes and breeds and did not show a potential bias for the limb on the inside of the circle at trot. However, when testing the algorithms on soft ground in future it may be that events are less pronounced and the threshold-based method may prove more valuable. We recommend a small pilot study before extending findings to soft ground, where velocity thresholds for foot on may have to be adjusted; for the purpose of qualitatively checking new data, we include sample trajectories from all horses and conditions in Supplemental Information 3.

There are two major benefits to the proposed approaches. Firstly, they require only one marker placed high on the hoof where it is more likely than a distally placed marker to stay clean, is less susceptible to accidental loss or damage and allows movement tracking on a soft surface where the hooves sink into the ground. Secondly, only a single algorithm is required to process data irrespective of movement condition with comparable accuracy and precision throughout. Hence, computational processing does not have to be customised to limb and gait (except for hard-coding condition-specific thresholds (method A) and hard-coding condition-specific acceleration components (method C)). The algorithms can be applied uniformly without introducing noteworthy bias between conditions or between front- and hind-foot timings. Our hope is that the method will find application in a wide range of research contexts: while we used the horse as a model, we assume that the method will lend itself to stride segmentation in a wide variety of hoofed animals, assisting movement analysis in the wild.

Further to the main study goals, we showed that different force thresholds in the range of 50–150 N had the greatest systematic effect on foot-off estimates in the hind limbs (up to on average 16 ms per condition), being greater than the effect on foot-on estimates or foot-off estimates of the forelimbs (up to on average ±7 ms per condition).

### Foot on detection algorithms

In Section A, we used a velocity threshold-based approach for foot on estimation. For movement on the straight line, our accuracy of −3 to −8 ms compares well with previous studies that reported 4.5 ms (Peham, Scheidl & Licka, 1999) or −5 to 25 ms (Boye et al., 2014). In the present study we refrained from using only the horizontal velocity component, which has been used by other authors (Peham, Scheidl & Licka, 1999; Boye et al., 2014), since it becomes unreliable in situations where the hoof is slipping during contact such as on soft ground (Riemersma et al., 1996; Johnston & Back, 2006; Setterbo et al., 2009) or on surfaces with different coefficients of friction (Pardoe et al., 2001; Mc Clinchey, Thomason & Runciman, 2004; Vos & Riemersma, 2006). We found that velocity-based foot on estimates have a notable bias (up to around 20 ms for the fore feet and 40 ms for the hind feet) for the inside limb on the circle during trot (see Fig. 3 and Supplemental Information 1, part 2). This has not been reported previously, because algorithms were validated only on the straight line (Peham, Scheidl & Licka, 1999; Galisteo et al., 2010; Olsen, Haubro Andersen & Pfau, 2012; Boye et al., 2014). Hence, if uncorrected, velocity-based foot on detection results in biased estimates for swing time, stance time and duty factor on the circle. One reason for this may be misalignment between vertical velocity and the horse’s limb on the circle, although lean angles of the metacarpus only range from 6 to 8° at walk and 15 to 21° at trot while lean angles of the metatarsus range from 6 to 7° at walk and 14 to 24° at trot (Hobbs, Licka & Polman, 2011) and hence should not explain the large bias. On the other hand, at trot lean angles for the inside distal limb are greater than for the outside limb (Hobbs, Licka & Polman, 2011), which may substantially alter the landing characteristics of the hoof and hence influence the velocity drop-off. From visual inspection of video and motion capture data it appears most likely that the foot on the inside of the circle adopts different impact mechanics which are causing the bias in velocity-threshold based foot-on detection. This would be in line with mounting evidence for altered loading regimes of inside and outside limb in horses during circling (Chateau et al., 2012; Chateau et al., 2013; Clayton, Starke & Merritt, 2014; Merritt, Starke & Clayton, 2014). We recommend using our event-based method for foot on estimation on the circle, and implementing the velocity thresholds which we identified as optimal for the susceptible conditions when using the threshold-based method.

In Section C, foot on was detected as a peak in vertical acceleration except for the inside foot during trot on the circle, where resultant velocity was used. The reason for adapting this approach was variability in the signal shape for the inside foot during trot on the circle, where for at least one horse the signal did not hold the impact peak information. Since resultant acceleration proved both accurate and precise for foot on detection during trot on the circle (although it did not perform as well for other conditions, compare Supplemental Information 1, part 3), we hence merged the two approaches for optimal algorithm performance across horses. For movement on the straight line, we found an accuracy of 4 to 7 ms, while previous accelerometer-based studies reported a mean error of 2.4 ms at walk and 1.8 ms at trot (Witte, Knill & Wilson, 2004). To calculate peaks in acceleration, we double-differentiated the optical motion capture data. This process introduces a time-shift of 1 frame in the datastream (see Supplemental Information 1, part 4). In this study, we did not correct for this shift, since the low-pass filter of the acceleration signal slightly shifted peak locations in the opposite direction to the differentiation shift, overall negating the effect of double-differentiation. However, it is an effect to be aware of when modifying the proposed method.

### Foot off detection algorithms

In Section B, the proposed foot-off detection algorithm based on distance travelled by the hoof performed well: average accuracy across horses ranged from −9 to 3 ms, precision across horses ranged from 2 to 7 ms and average precision within horses ranged from 4 to 7 ms. On the straight line, our values ranged from −5 to 3 ms while values reported previously ranged from −35 to 39 ms depending on limb, gait and algorithm (Boye et al., 2014). In the past, horizontal velocity of a single marker attached to the toe tip gave amongst the most accurate results for foot off detection for movement on a straight line: an average bias of −6.3 ms (Peham, Scheidl & Licka, 1999) or −3.8 to −13.6 ms (Boye et al., 2014) was reported. Our values for accuracy matched these findings but the associated precision was substantially better: on the straight line, our precision estimate ranged from 2 to 5 ms (across horse means) or 4 to 6 ms (average precision within horses), compared with previously reported precision of 20–43 ms across horses (Boye et al., 2014). This means that our algorithm introduces less unsystematic variation to a dataset and allows for the detection of smaller effect sizes. Alternative algorithms for foot off detection either show almost 25 ms difference in accuracy between fore- and hind limbs, or fall within the range of values reported here (Boye et al., 2014). The delayed detection of foot off compared to kinetic data when using trigonometric thresholds results from inaccuracy of the ‘allowable’ horizontal movement during breakover before attributing it to swing. We based this distance on the vertical height of the hoof marker during stance, assuming an approximately square shape of the simplified rigid body. In future, this distance can be optimised, trading computational simplicity for increased accuracy of the foot off estimate, especially in horses with a more acute hoof angle.

In Section D, the proposed foot-off detection algorithm based on the vertical velocity profile of the hoof marker performed well: across gaits and movement directions, average accuracy across horses ranged from 2 to 10 ms, precision across horses ranged from 3 to 6 ms and average precision within horses ranged from 3 to 8 ms. For movement on the straight line, we found an accuracy of 3 to 5 ms, while previous accelerometer-based studies reported a mean error of 3.6 ms at walk and 2.4 ms at trot (Witte, Knill & Wilson, 2004). We selected the local minimum in vertical velocity as the event corresponding to lift-off, assuming that it correlates with limb activation/lift following breakover to prevent dragging of the toe during protraction. We are not aware of previous studies having defined the exact event corresponding to lift-off and have not been able to test event validity in conditions other than those described here. Especially using the algorithm on soft ground might result in an altered velocity profile, which may require the selection of an alternative event for foot off detection.

### Differences in accuracy between fore- and hind limbs and reliability of relative stride timings

The present study highlighted small differences between accuracy estimates of fore- and hind limbs within the same condition. This was reflected by differences in the time-shift between foot on and foot off events of fore- and hind limbs (Table 6), with a systematic bias of up to 10 ms across both threshold- and event based methods and ranging from −0.3 to 1.3% stride duration (see Table 6). These findings are in range or better than previously reported findings: in one study the estimated stance duration increased by 11 ms due to artifacts related to kinematic event detection (Peham, Scheidl & Licka, 1999). In a study comparing multiple algorithms, estimated stance duration differed from the kinetic gold standard by −60 to 69 ms between different algorithms (Boye et al., 2014). The reason for these differences between event detection for fore- and hind limbs may be differences in hoof shape (Balch, White & Butler, 1991) which can significantly affect breakover characteristics (Clayton, Sigafoos & Curle, 1991; Keegan et al., 2005): Clayton 1991 reported the “normal” angulation of front hooves as 53.7°(range: 48°–55°) and hind hooves as 55.7°(range: 52°–60°). Further, differences between fore- and hind limb kinematics (Back et al., 1995a; Back et al., 1995b; Back et al., 1995c) may affect the foot off estimates. As above, this finding highlights a potential small bias in the calculation of stance and swing timings between limbs and across conditions. The highlighted uncertainty about the exact duration of stance and swing should be considered in studies employing kinematic event detection and discussed in case of detected main effects (regarding e.g., stance time, swing time or duty factor) that range within effects attributable to the stride segmentation approach.

### Effect of force thresholds on kinetic stride events

In this study we showed that kinetic event detection is systematically affected by the chosen force threshold, although the introduced bias was small. For foot on detection, changing the threshold from 50 N to 150 N resulted in average delay of the event of up to 6 ms across all pooled strides within each condition, meaning that the event occurred slightly later with the higher threshold value. This small effect is explicable by the sharp rise in the vertical force at impact: in the present study, vertical force of the first frame exceeding the threshold was typically around 400–500 N. The effect of a specific force threshold was due to those trials where by chance the sampled force had just started rising and was hence in range of the threshold. Since in Method C we are using the impact acceleration peak as a proxy for foot on, it is in fact these trials where foot on was ‘caught’ relatively early that may have introduced small variation to the method comparison, as peak acceleration at impact should correspond to a local peak in the force trajectory (Merkens & Schamhardt, 1994). For foot off detection, changing the threshold from 50 N to 150 N resulted in an average delay of the event of up to −16 ms across all pooled strides within each condition, meaning that the event occurred slightly earlier with the higher threshold value. Similar to foot on detection, this is a consequence of the rapid fall in force towards the end of the stride. Especially for the hind feet, the force trajectories often showed a more gradual ‘tailing-off’ in late stance, which makes them more sensitive to set force thresholds than trajectories that show a large gradient/rapid change. In this study we used a force threshold of 75 N; the rationale behind this was to accommodate instruments that have a slightly higher noise plateau, as we have seen noise levels around 50 N in the past. Based on the above results, the difference between our 75 N threshold and a 50 N threshold used in other validation studies is marginal. Based on our findings, we must of course highlight that potential differences in bias between studies in the order of a few milliseconds may originate from threshold selection and should not be considered evidence to select one method over another.

### Study limitations

Hoof shape, pathology or shoeing may influence roll-over characteristics as well as general impact characteristics and hence potentially have an influence on footfall timing estimates. This remains open to future investigation and robustness testing. In this study, the comparatively small sample size (*N* = 8) and correction for multiple testing meant that potential pairwise differences between conditions could not be detected by a non-parametric test, while a general repeated measures test sometimes showed a significant effect between conditions. However, for a larger sample size we expect a potentially significant effect to be small: the largest difference between accuracy estimates across conditions was 10 ms. Considering that the stride duration for walk is around 1,100 ms and for trot around 700 ms, a potential bias of 10 ms can be considered negligible. At our sample rate of 100 Hz we had a resolution of 10 ms to detect events. While this leads to slight over- or underestimates compared to the true event on an individual stride basis, these average out over multiple strides and estimated mean values remain accurate at the level of reporting here. Other studies which used higher sampling rates (for example 240 Hz in Boye et al., 2014 and Peham, Scheidl & Licka, 1999) reported values in the same order as found in the present work. Some of the strides used to calculate mean values for a horse may have come from the same trial and their independence could hence be discussable. Since we compare the difference between kinematic and kinetic events, we do not consider this to be an issue as even for a dependent pair of strides the impact characteristics may change. Since in our study we retained the maximum number of strides per horse, N was not equal between horses and conditions. This may have influenced the accuracy of the horse-based mean and SD estimation. However, reducing the number of strides to the smallest common N would have had a more detrimental effect, as this would result in an inaccurate estimate for each horse. Method comparison may also be corrected for repeated measures (Bland & Altman, 2007; Carstensen, Simpson & Gurrin, 2008).

## Supplemental Information

10.7717/peerj.783/supp-1 Supplemental Information 1 Supplementary information as referenced in text

10.7717/peerj.783/supp-2 Supplemental Information 2 Example data to illustrate accuracy of sub-resolution means

10.7717/peerj.783/supp-3 Supplemental Information 3 Supplementary graphs of individual strides in zipped folder

## References

[ref-1] Alexander RM (2003). Principles of animal locomotion.

[ref-2] Back W, Schamhardt H, Hartman W, Barneveld A (1995a). Kinematic differences between the distal portions of the forelimbs and hind limbs of horses at the trot. American Journal of Veterinary Research.

[ref-3] Back W, Schamhardt HC, Savelberg HHCM, Van Den Bogert AJ, Bruin G, Hartman W, Barneveld A (1995b). How the horse moves: 1. Significance of graphical representations of equine forelimb kinematics. Equine Veterinary Journal.

[ref-4] Back W, Schamhardt HC, Savelberg HHCM, Van Den Bogert AJ, Bruin G, Hartman W, Barneveld A (1995c). How the horse moves: 2. Significance of graphical representations of equine hind limb kinematics. Equine Veterinary Journal.

[ref-5] Balch OK, Butler D, Collier MA (1997). Balancing the normal foot: hoof preparation, shoe fit and shoe modification in the performance horse. Equine Veterinary Education.

[ref-6] Balch O, White K, Butler D (1991). Factors involved in the balancing of equine hooves. Journal of the American Veterinary Medical Association.

[ref-7] Barre A, Armand S (2014). Biomechanical ToolKit: open-source framework to visualize and process biomechanical data. Computer Methods and Programs in Biomedicine.

[ref-8] Biewener AA (1983). Allometry of quadrupedal locomotion: the scaling of duty factor, bone curvature and limb orientation to body size. Journal of Experimental Biology.

[ref-9] Biewener AA (2003). Animal locomotion.

[ref-10] Bland JM, Altman DG (1986). Statistical methods for assessing agreement between two methods of clinical measurement. The Lancet.

[ref-11] Bland JM, Altman DG (2007). Agreement between methods of measurement with multiple observations per individual. Journal of Biopharmaceutical Statistics.

[ref-12] Boye JK, Thomsen MH, Pfau T, Olsen E (2014). Accuracy and precision of gait events derived from motion capture in horses during walk and trot. Journal of Biomechanics.

[ref-13] Burn JF, Brockington C (2001). Quantification of hoof deformation using optical motion capture. Equine Veterinary Journal.

[ref-14] Burn JF, Usmar SJ (2005). Hoof landing velocity is related to track surface properties in trotting horses. Equine and Comparative Exercise Physiology.

[ref-15] Carstensen B, Simpson J, Gurrin LC (2008). Statistical models for assessing agreement in method comparison studies with replicate measurements. The International Journal of Biostatistics.

[ref-16] Chang Y-H, Kram R (2007). Limitations to maximum running speed on flat curves. Journal of Experimental Biology.

[ref-17] Chateau H, Camus M, Holden L, Falala S, Ravary B, Vergari C, Denoix JM, Pourcelot P, Crevier-Denoix N (2012). Ground reaction force and moments around the hoof axes during circling on different ground surfaces at the trot.

[ref-18] Chateau H, Camus M, Holden-Douilly L, Falala S, Ravary B, Vergari C, Lepley J, Denoix J-M, Pourcelot P, Crevier-Denoix N (2013). Kinetics of the forelimb in horses circling on different ground surfaces at the trot. The Veterinary Journal.

[ref-19] Chateau H, Degueurce C, Denoix JM (2006). Three-dimensional kinematics of the distal forelimb in horses trotting on a treadmill and effects of elevation of heel and toe. Equine Veterinary Journal.

[ref-20] Chateau H, Holden L, Robin D, Falala S, Pourcelot P, Estoup P, Denoix JM, Crevier-Denoix N (2010). Biomechanical analysis of hoof landing and stride parameters in harness trotter horses running on different tracks of a sand beach (from wet to dry) and on an asphalt road. Equine Veterinary Journal.

[ref-21] Clayton HM (1997). Classification of collected trot, passage and piaffe based on temporal variables. Equine Veterinary Journal.

[ref-22] Clayton HM, Colborne GR, Burns TE (1995). Kinematic analysis of successful and unsuccessful attempts to clear a water jump. Equine Veterinary Journal.

[ref-23] Clayton HM, Sha DH (2006). Head and body centre of mass movement in horses trotting on a circular path. Equine Veterinary Journal.

[ref-24] Clayton HM, Sigafoos R, Curle RD (1991). Effect of three shoe types on the duration of breakover in sound trotting horses. Journal of Equine Veterinary Science.

[ref-25] Clayton HM, Starke SD, Merritt JS (2014). Individual limb contributions to centripetal force generation during circular trot. Equine Veterinary Journal.

[ref-26] Deuel N, Park J (1991). Kinematic analysis of jumping sequences of Olympic show jumping horses. Equine Exercise Physiology.

[ref-27] Dickinson MH, Farley CT, Full RJ, Koehl MAR, Kram R, Lehman S (2000). How animals move: an integrative view. Science.

[ref-28] Douglas JE, Mittal C, Thomason JJ, Jofriet JC (1996). The modulus of elasticity of equine hoof wall: implications for the mechanical function of the hoof. The Journal of Experimental Biology.

[ref-29] Dyhre-Poulsen P, Smedegaard HH, Roed J, Korsgaard E (1994). Equine hoof function investigated by pressure transducers inside the hoof and accelerometers mounted on the first phalanx. Equine Veterinary Journal.

[ref-30] Galisteo AM, Garrido-Castro JL, Miró F, Plaza C, Medina-Carnicer R (2010). Assessment of a method to determine the stride phases in trotting horses from video sequences under field conditions. Wiener Tierarztliche Monatsschrift.

[ref-31] Ghoussayni S, Stevens C, Durham S, Ewins D (2004). Assessment and validation of a simple automated method for the detection of gait events and intervals. Gait & Posture.

[ref-32] Hansen AH, Childress DS, Meier MR (2002). A simple method for determination of gait events. Journal of Biomechanics.

[ref-33] Hobbs SJ, Licka T, Polman R (2011). The difference in kinematics of horses walking, trotting and cantering on a flat and banked 10 m circle. Equine Veterinary Journal.

[ref-34] Hobbs S, Orlande O, Edmundson C, Northrop A, Martin J (2010). Development of a method to identify foot strike on an arena surface: application to jump landing. Comparative Exercise Physiology.

[ref-35] Hodson EF, Clayton HM, Lanovaz JL (1999). Temporal analysis of walk movements in the Grand Prix dressage test at the 1996 Olympic Games. Applied Animal Behaviour Science.

[ref-36] Hreljac A, Marshall RN (2000). Algorithms to determine event timing during normal walking using kinematic data. Journal of Biomechanics.

[ref-37] Hunt ER (2001). Response of twenty-seven horses with lower leg injuries to cold spa bath hydrotherapy. Journal of Equine Veterinary Science.

[ref-38] Johnston C, Back W (2006). Hoof ground interaction: when biomechanical stimuli challenge the tissues of the distal limb. Equine Veterinary Journal.

[ref-39] Keegan KG, Satterley JM, Skubic M, Yonezawa Y, Cooley JM, Wilson DA, Kramer J (2005). Use of gyroscopic sensors for objective evaluation of trimming and shoeing to alter time between heel and toe lift-off at end of the stance phase in horses walking and trotting on a treadmill. American Journal of Veterinary Research.

[ref-40] Kiss RM (2010). Comparison between kinematic and ground reaction force techniques for determining gait events during treadmill walking at different walking speeds. Medical Engineering & Physics.

[ref-41] Landeau LJ, Barrett DJ, Batterman SC (1983). Mechanical properties of equine hooves. American Journal of Veterinary Research.

[ref-42] Leach DH, Zoerb GC (1983). Mechanical properties of equine hoof wall tissue. American Journal of Veterinary Research.

[ref-43] Leitch J, Stebbins J, Paolini G, Zavatsky AB (2011). Identifying gait events without a force plate during running: A comparison of methods. Gait & Posture.

[ref-44] Mc Clinchey HL, Thomason JJ, Runciman RJ (2004). Grip and slippage of the horse’s hoof on solid substrates measured ex vivo. Biosystems Engineering.

[ref-45] Merkens HW, Schamhardt HC (1994). Relationships between ground reaction force patterns and kinematics in the walking and trotting horse. Equine Veterinary Journal.

[ref-46] Merritt JS, Starke SD, Clayton HM (2014). The point of application of the ground reaction force moves in circling horses. Equine Veterinary Journal.

[ref-47] Mickelborough J, Van Der Linden ML, Richards J, Ennos AR (2000). Validity and reliability of a kinematic protocol for determining foot contact events. Gait & Posture.

[ref-48] Miller A (2009). Gait event detection using a multilayer neural network. Gait & Posture.

[ref-49] Mooij MJW, Jans W, Den Heijer GJL, De Pater M, Back W (2013). Biomechanical responses of the back of riding horses to water treadmill exercise. The Veterinary Journal.

[ref-50] Muybridge E (2000). Animals in motion.

[ref-51] O’Connor CM, Thorpe SK, O’Malley MJ, Vaughan CL (2007). Automatic detection of gait events using kinematic data. Gait & Posture.

[ref-52] Olsen E, Haubro Andersen P, Pfau T (2012). Accuracy and precision of equine gait event detection during walking with limb and trunk mounted inertial sensors. Sensors.

[ref-53] Pantall A, Gregor RJ, Prilutsky BI (2012). Stance and swing phase detection during level and slope walking in the cat: effects of slope, injury, subject and kinematic detection method. Journal of Biomechanics.

[ref-54] Pardoe CH, Mcguigan MP, Rogers KM, Rowe LL, Wilson AM (2001). The effect of shoe material on the kinetics and kinematics of foot slip at impact on concrete. Equine Veterinary Journal.

[ref-55] Parsons KJ, Pfau T, Ferrari M, Wilson AM (2008a). High-speed gallop locomotion in the Thoroughbred racehorse. II. The effect of incline on centre of mass movement and mechanical energy fluctuation. Journal of Experimental Biology.

[ref-56] Parsons KJ, Pfau T, Wilson AM (2008b). High-speed gallop locomotion in the Thoroughbred racehorse. I. The effect of incline on stride parameters. Journal of Experimental Biology.

[ref-57] Parsons KJ, Wilson AM (2006). The use of MP3 recorders to log data from equine hoof mounted accelerometers. Equine Veterinary Journal.

[ref-58] Peham C, Scheidl M, Licka T (1999). Limb locomotion–speed distribution analysis as a new method for stance phase detection. Journal of Biomechanics.

[ref-59] Pfau T, Spence A, Starke S, Ferrari M, Wilson A (2009). Modern riding style improves horse racing times. Science.

[ref-60] Riemersma DJ, Van Den Bogert AJ, Jansen MO, Schamhardt HC (1996). Tendon strain in the forelimbs as a function of gait and ground characteristics and in vitro limb loading in ponies. Equine Veterinary Journal.

[ref-61] Schamhardt HC, Merkens HW (1994). Objective determination of ground contact of equine limbs at the walk and trot: comparison between ground reaction forces, accelerometer data and kinematics. Equine Veterinary Journal.

[ref-62] Setterbo JJ, Garcia TC, Campbell IP, Reese JL, Morgan JM, Kim SY, Hubbard M, Stover SM (2009). Hoof accelerations and ground reaction forces of Thoroughbred racehorses measured on dirt, synthetic, and turf track surfaces. American Journal of Veterinary Research.

[ref-63] Starke SD, Willems E, May SA, Pfau T (2012a). Vertical head and trunk movement adaptations of sound horses trotting in a circle on a hard surface. The Veterinary Journal.

[ref-64] Starke SD, Witte TH, May SA, Pfau T (2012b). Accuracy and precision of hind limb foot contact timings of horses determined using a pelvis-mounted inertial measurement unit. Journal of Biomechanics.

[ref-65] Thomason JJ, Peterson ML (2008). Biomechanical and mechanical investigations of the hoof-track interface in racing horses. Veterinary Clinics of North America: Equine Practice.

[ref-66] Van Heel MCV, Barneveld A, Van Weeren PR, Back W (2004). Dynamic pressure measurements for the detailed study of hoof balance: the effect of trimming. Equine Veterinary Journal.

[ref-67] Vos NJ, Riemersma DJ (2006). Determination of coefficient of friction between the equine foot and different ground surfaces: an *in vitro* study. Equine and Comparative Exercise Physiology.

[ref-68] Weyand PG, Kelly M, Blackadar T, Darley JC, Oliver SR, Ohlenbusch NE, Joffe SW, Hoyt RW (2001). Ambulatory estimates of maximal aerobic power from foot -ground contact times and heart rates in running humans. Journal of Applied Physiology.

[ref-69] Wilson AM, Seelig TJ, Shield RA, Silverman BW (1998). The effect of foot imbalance on point of force application in the horse. Equine Veterinary Journal.

[ref-70] Witte TH, Hirst CV, Wilson AM (2006). Effect of speed on stride parameters in racehorses at gallop in field conditions. Journal of Experimental Biology.

[ref-71] Witte TH, Knill K, Wilson AM (2004). Determination of peak vertical ground reaction force from duty factor in the horse (Equus caballus). Journal of Experimental Biology.

[ref-72] Zeni JA, Richards JG, Higginson JS (2008). Two simple methods for determining gait events during treadmill and overground walking using kinematic data. Gait & Posture.

