# Supplementary material for: A universal approach to determine footfall timings from kinematics of a single foot marker in hoofed animals
Source: PeerJ. 2015 Mar 26;3:e783. doi: 10.7717/peerj.783 (PMC4493675; doi:10.7717/peerj.783)
Supplement: Supplemental Information 1 [file peerj-03-783-s001.pdf]

SUPPLEMETARY INFORMATION:

A universal approach to determine footfall timings from kinematics of a single foot marker in hoofed animals.

Sandra D. Starke and Hilary M. Clayton

Part 1: Four quarantined strides

The figure below indicate the four strides (out of thousands) that had to be quarantined and excluded from further analysis due to erroneous features.

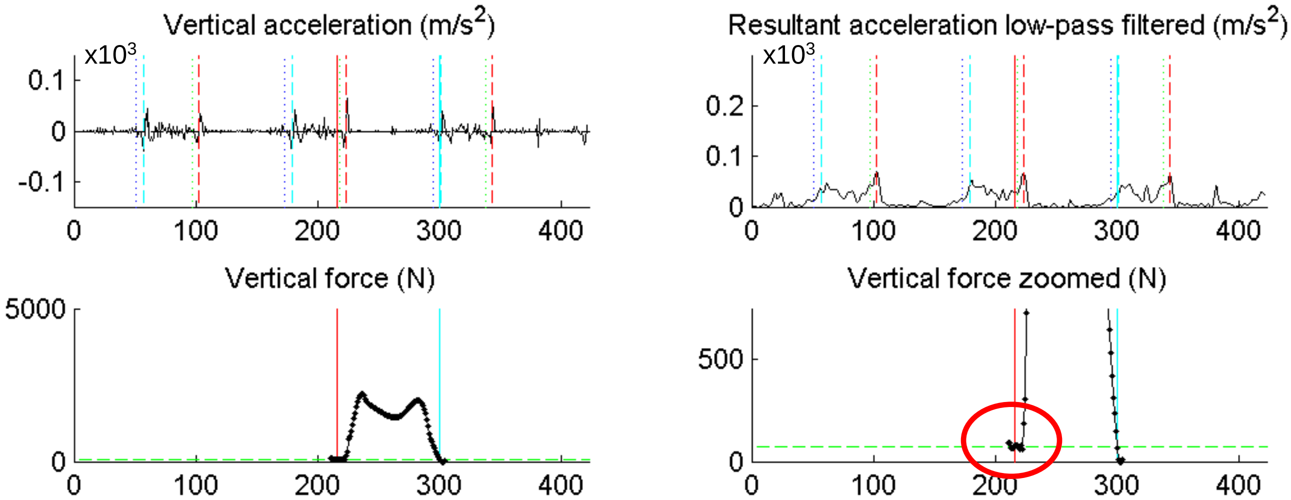

Supplementary Figure 1.1. Stride of RH during walk on the straight line, quarantined due to blip in force trace prior to foot contact.

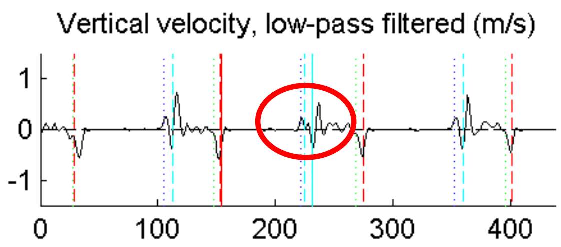

Supplementary Figure 1.2. Stride of LH during walk on the circle with the limb on the inside, quarantined due to noise in filtered lift-off signal.

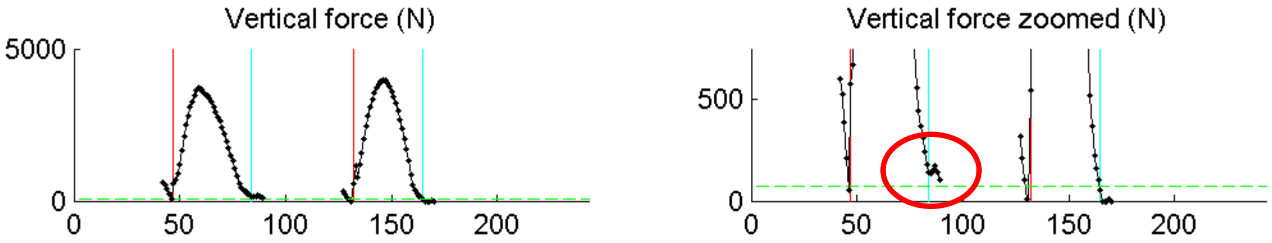

Supplementary Figure 1.3. Stride of LH during trot on the circle with the limb on the outside, quarantined due to unexplained noise in the force at lift off.

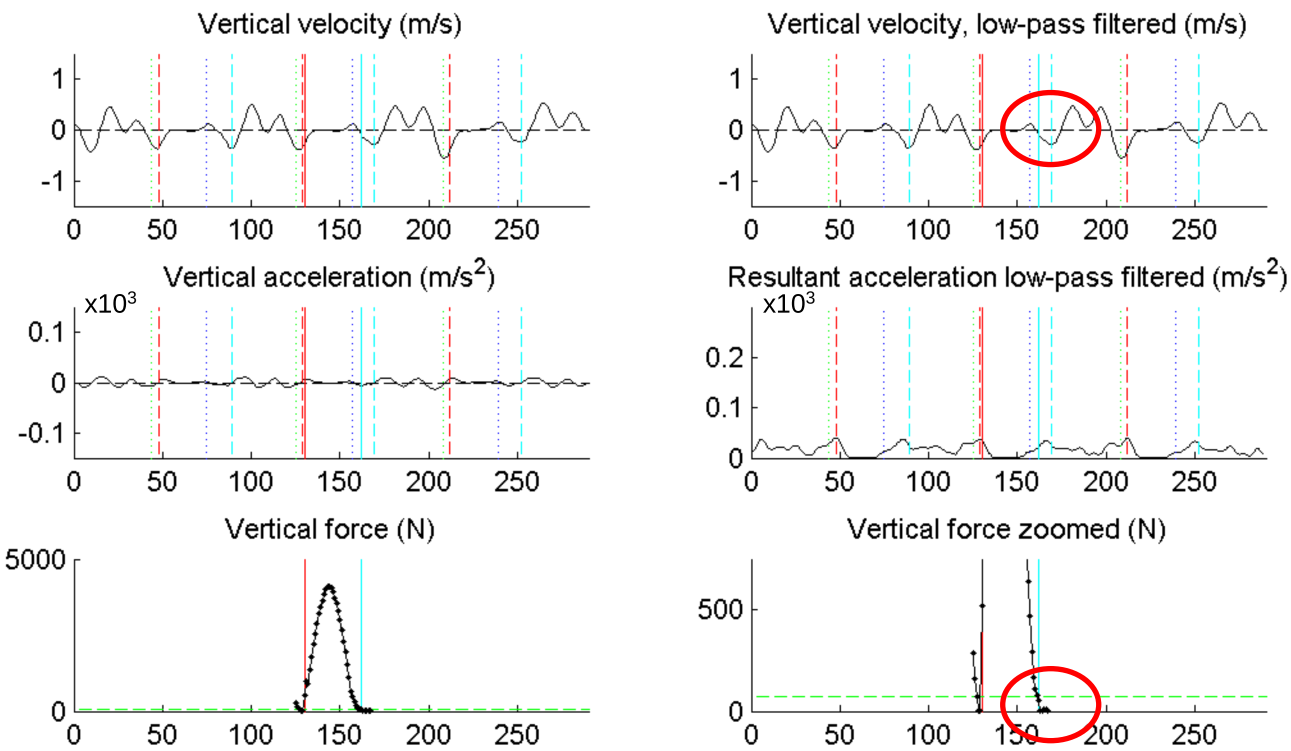

Supplementary Figure 1.4. Stride of RH during trot on the circle with the limb on the inside, quarantined due to unexplained mismatch in events between force and kinematic data resulting in a outlier.

Part 2: Results for different set velocity thresholds

The figures below illustrate example results for accuracy of the velocity-threshold based foot-on detection method for selected fixed thresholds 0.5 m s^{-1} and 0.7 m s^{-1}. Thresholds for inside fore- and hind limbs during trot on the circle had to be adjusted in order to negate the bias.

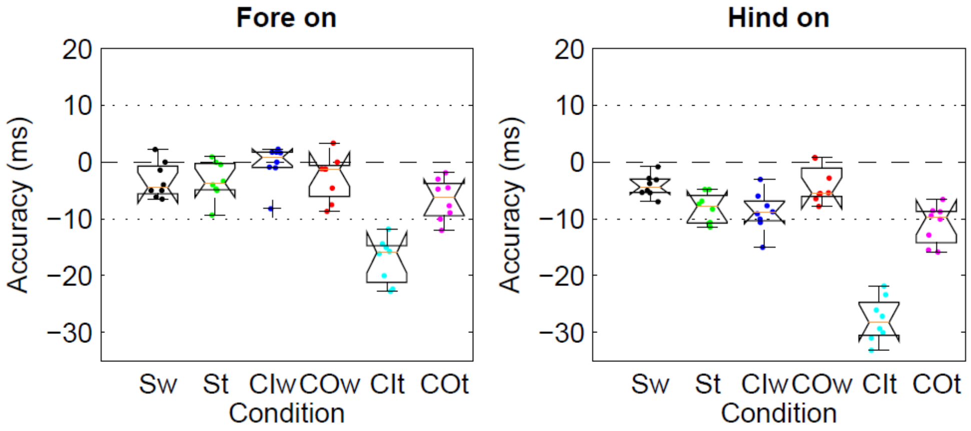

Supplementary Figure 2.1. Accuracy for foot-on events of fore- and hind limbs based on a fixed velocity threshold of 0.5 m s^{-1}.

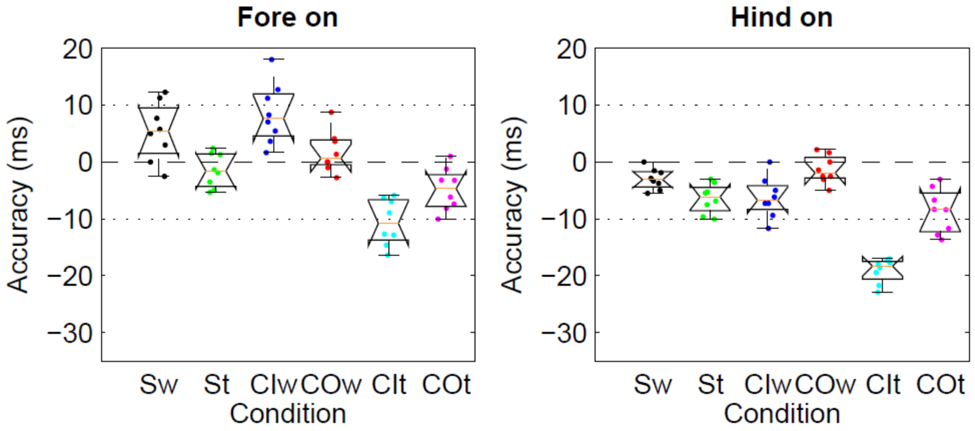

Supplementary Figure 2.2. Accuracy for foot-on events of fore- and hind limbs based on a fixed velocity threshold of 0.7 m s^{-1}.

Part 3: Results for vertical and resultant acceleration-based foot-on detection across all conditions

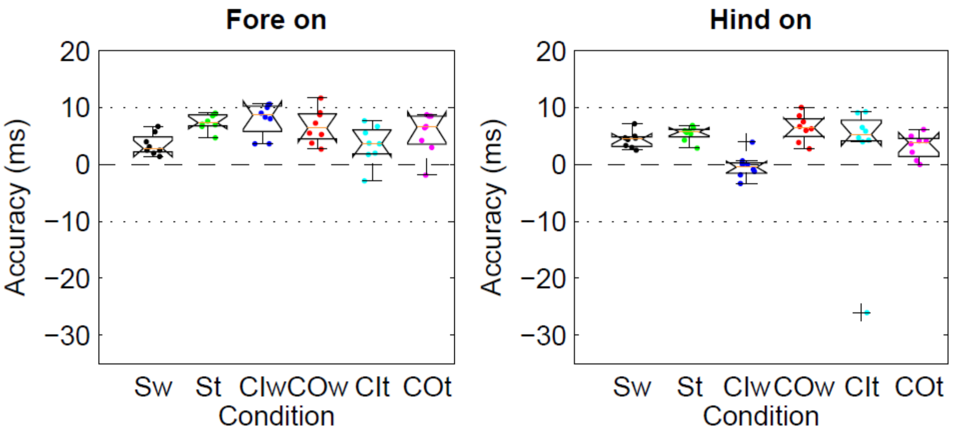

Supplementary Figure 3.1. Accuracy for foot-on events of fore- and hind limbs based on a peak in vertical acceleration. The outlier for the hind limb, CIt (circle with limb on the inside of the circle, trot) is a result of an absent distinct signal in the data for this horse.

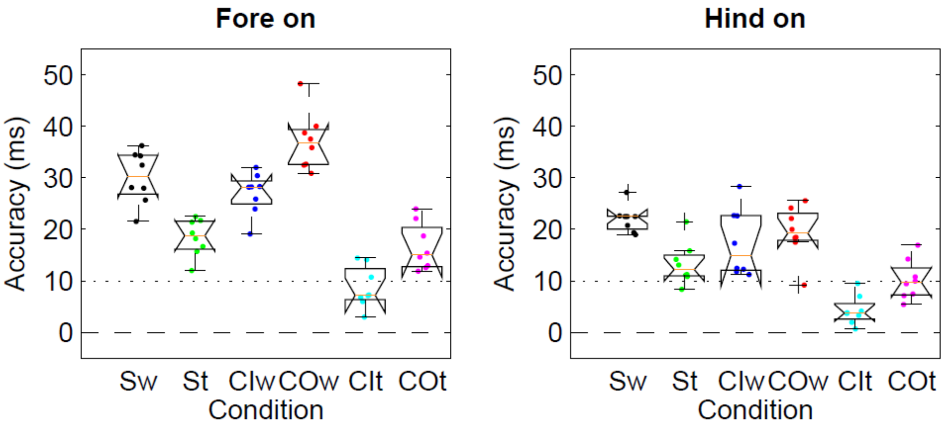

Supplementary Figure 3.2. Accuracy for foot-on events of fore- and hind limbs based on resultant acceleration. While this method works well for trot on the circle, it introduces inacceptable bias to other conditions due to variability in signal shape, especially for the forelimbs.

Part 4: Time shift due to double-differentiation

The figures below illustrates that double-differentiation usually shifts the data by one frame. In the present paper we did not correct for this shift, as it was offset by the effect of the low-pass filter.

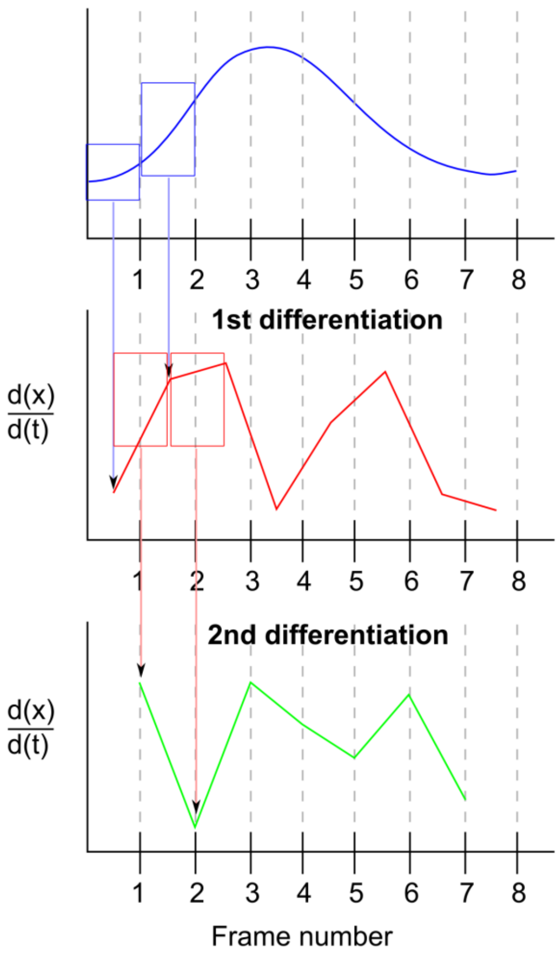

Supplementary Figure 4.1. The effect of double-differentiation on the time code of data. The differentiated signals are illustrated as absolute values. Simple differentiation calculates a change in a signal within a specified time window. This change is hence an average value and corresponds to the time point in between the two points framing the window. Differentiating this derived signal once more results in the same effect, hence shifting the whole signal one frame to the right.
