# Supplementary figures and images for: A universal approach to determine footfall timings from kinematics of a single foot marker in hoofed animals (part 1 of 2)
Source: PeerJ. 2015 Mar 26;3:e783. doi: 10.7717/peerj.783 (PMC4493675; doi:10.7717/peerj.783)

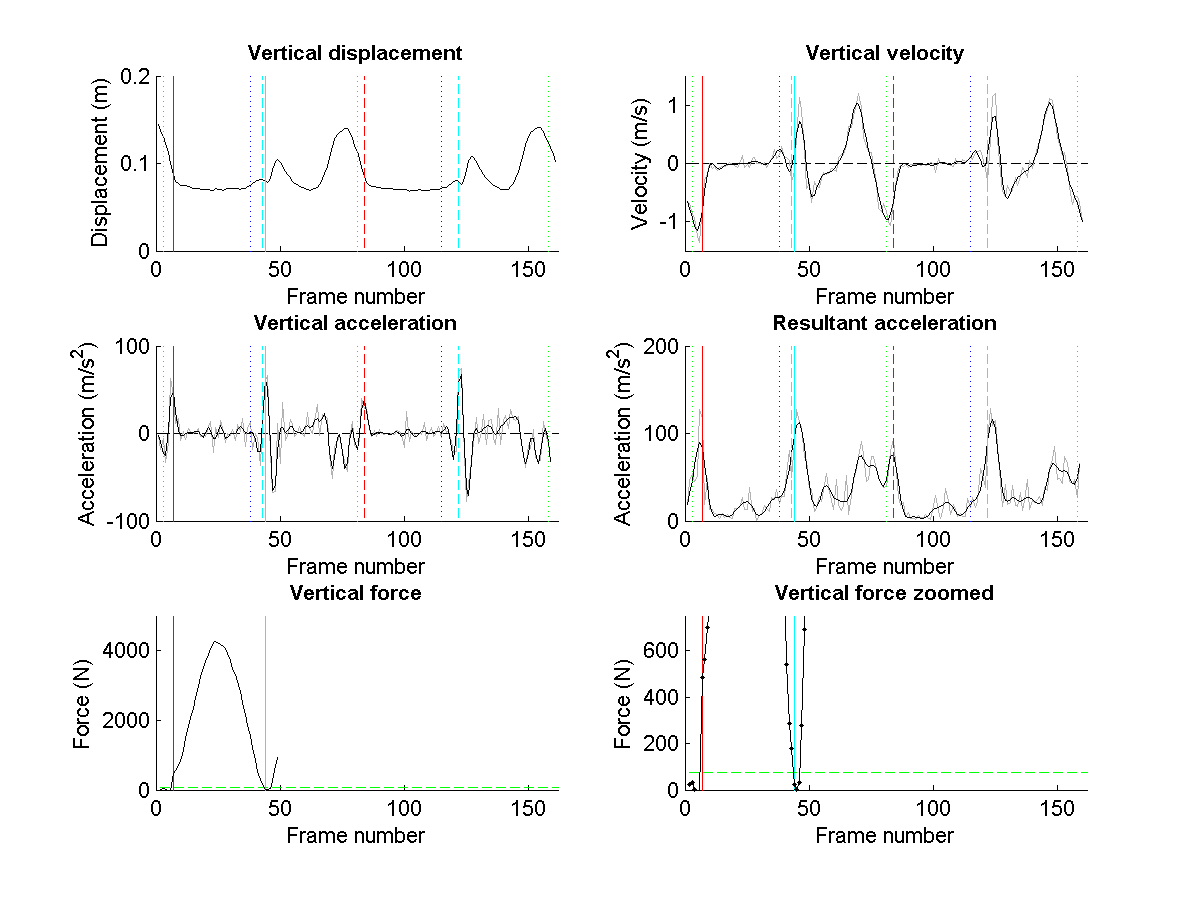

Supplement: Supplemental Information 3 [file peerj-03-783-s003.zip › Suppl figures/Event-based/Event_plot-LF_Horse10_circle_left_trot_03.png]

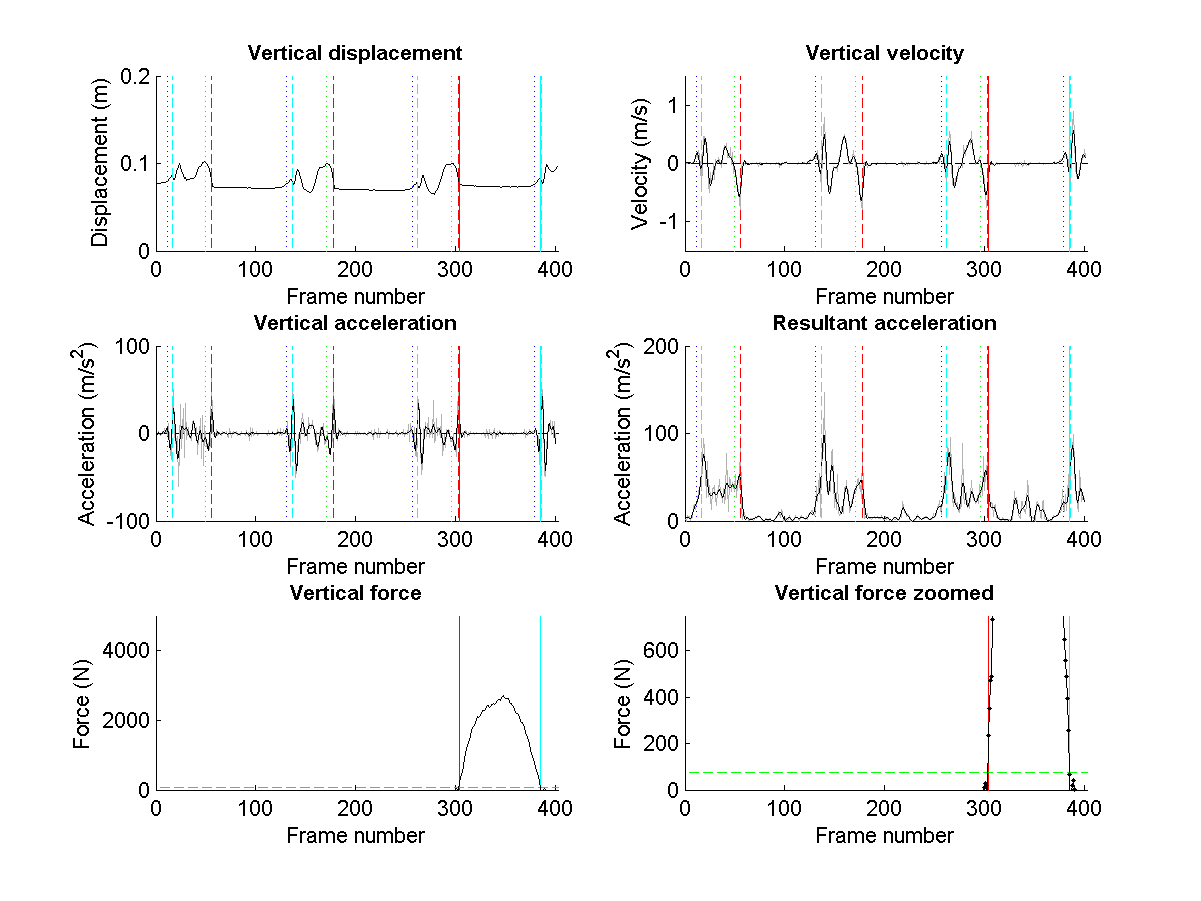

Supplement: Supplemental Information 3 [file peerj-03-783-s003.zip › Suppl figures/Event-based/Event_plot-LF_Horse10_circle_left_walk_02.png]

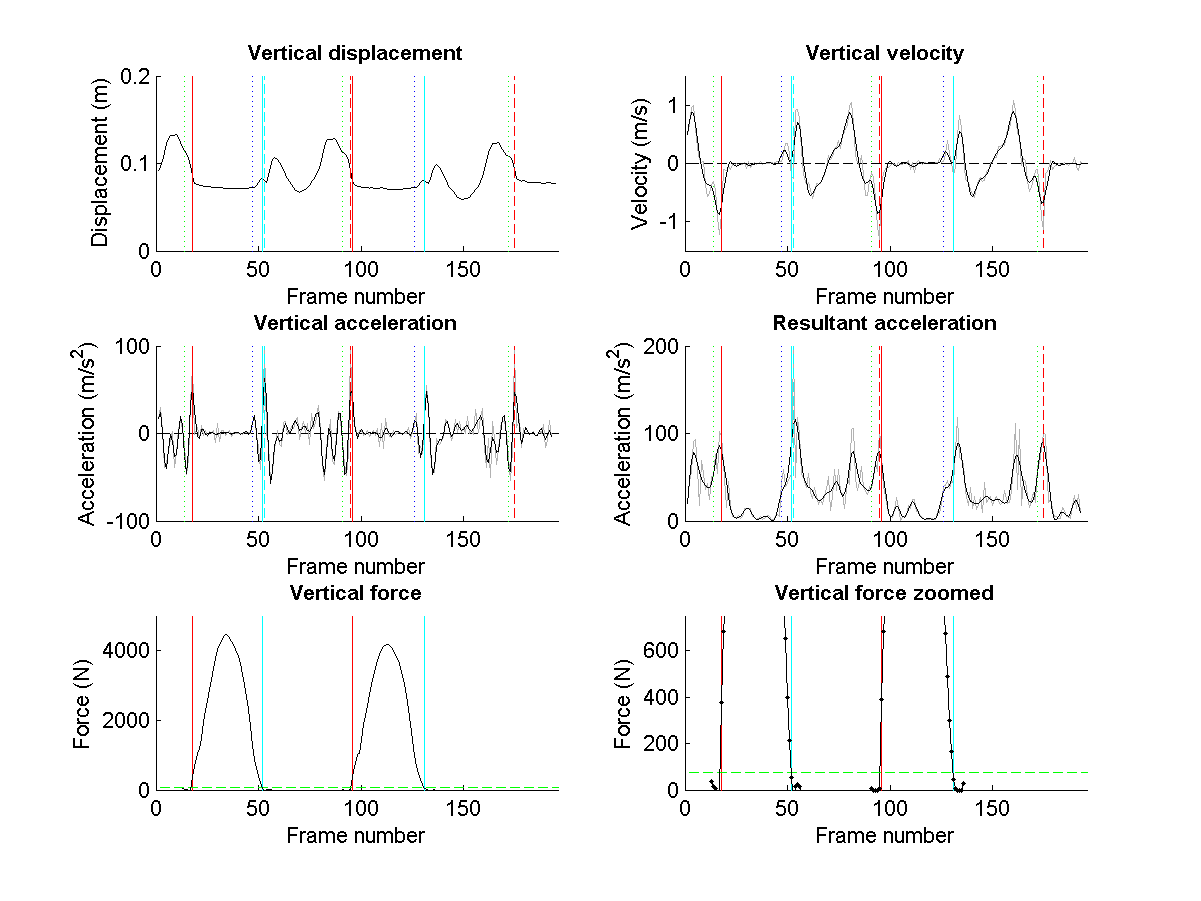

Supplement: Supplemental Information 3 [file peerj-03-783-s003.zip › Suppl figures/Event-based/Event_plot-LF_Horse10_circle_right_trot_03.png]

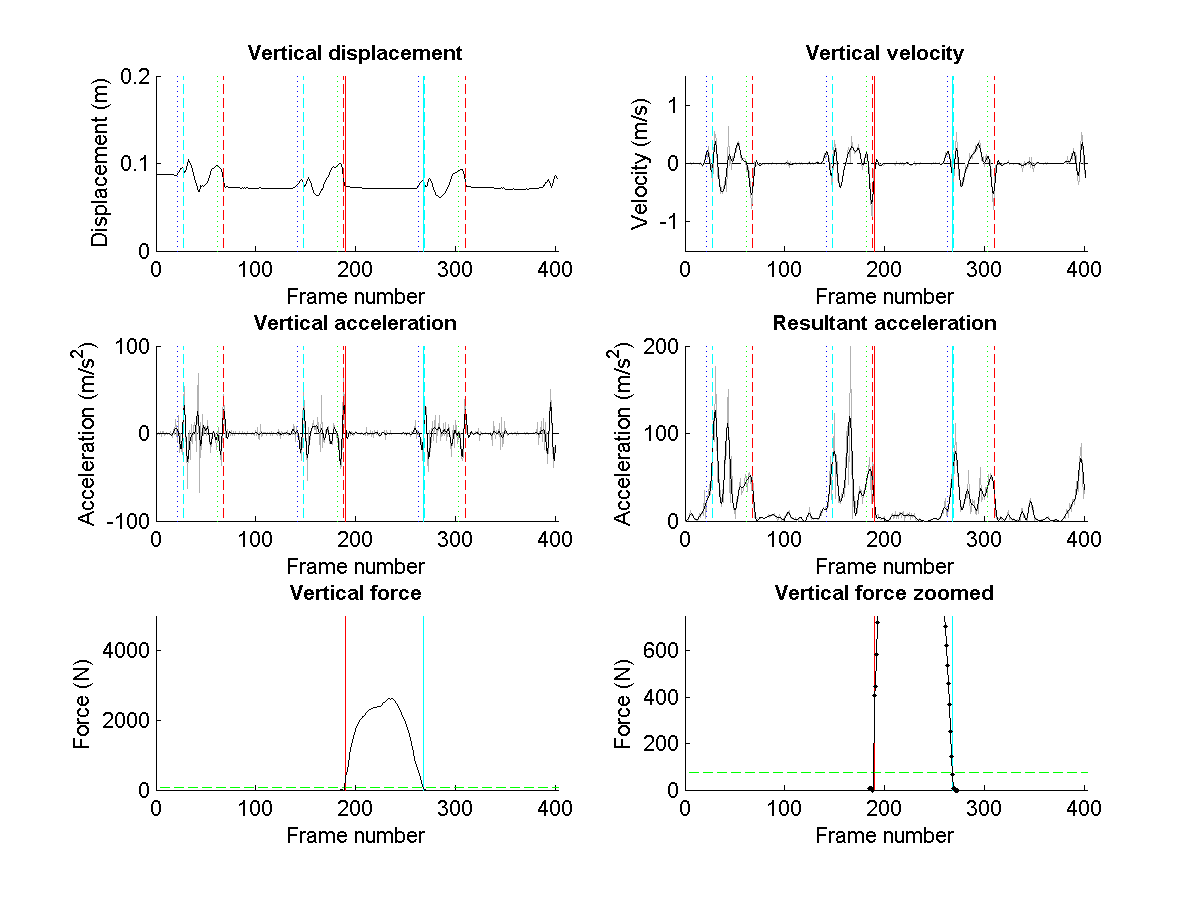

Supplement: Supplemental Information 3 [file peerj-03-783-s003.zip › Suppl figures/Event-based/Event_plot-LF_Horse10_circle_right_walk_08.png]

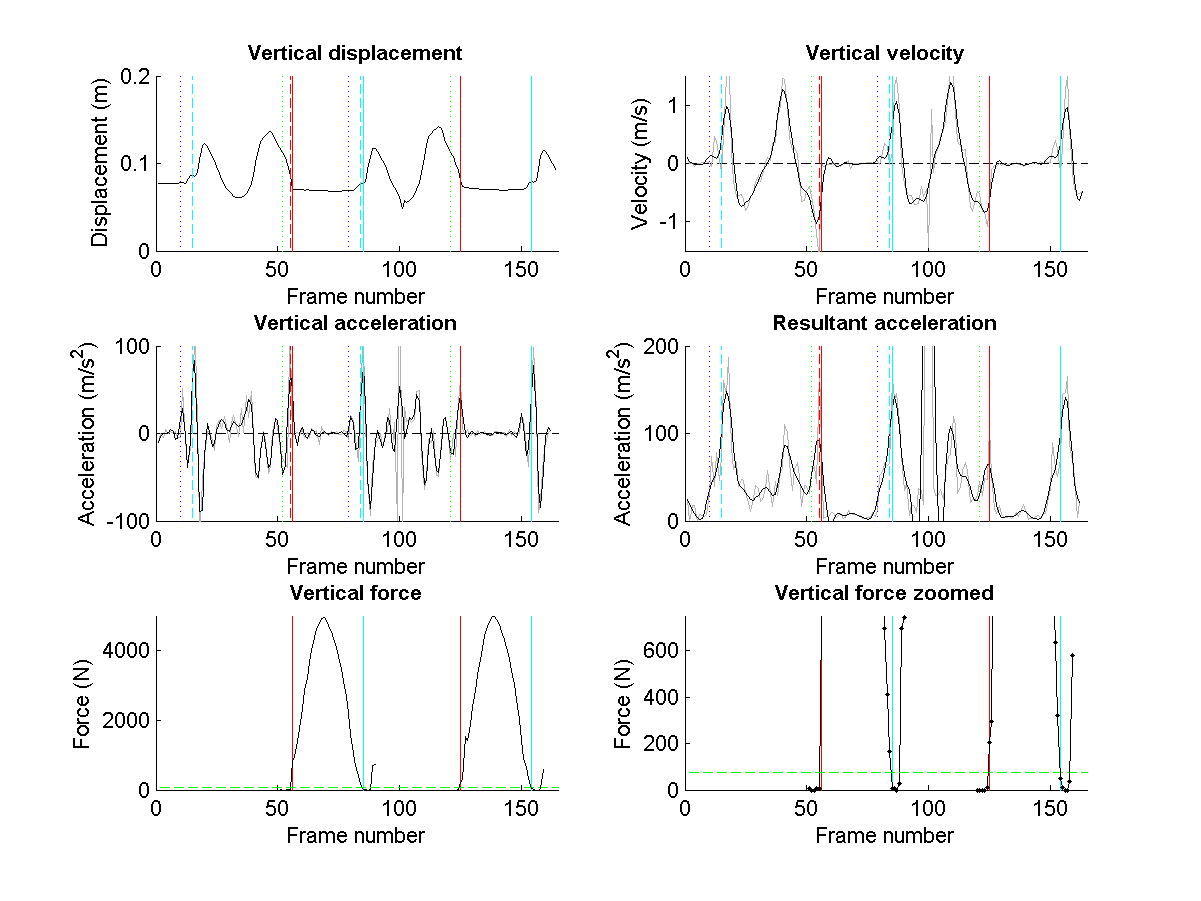

Supplement: Supplemental Information 3 [file peerj-03-783-s003.zip › Suppl figures/Event-based/Event_plot-LF_Horse10_trot02.png]

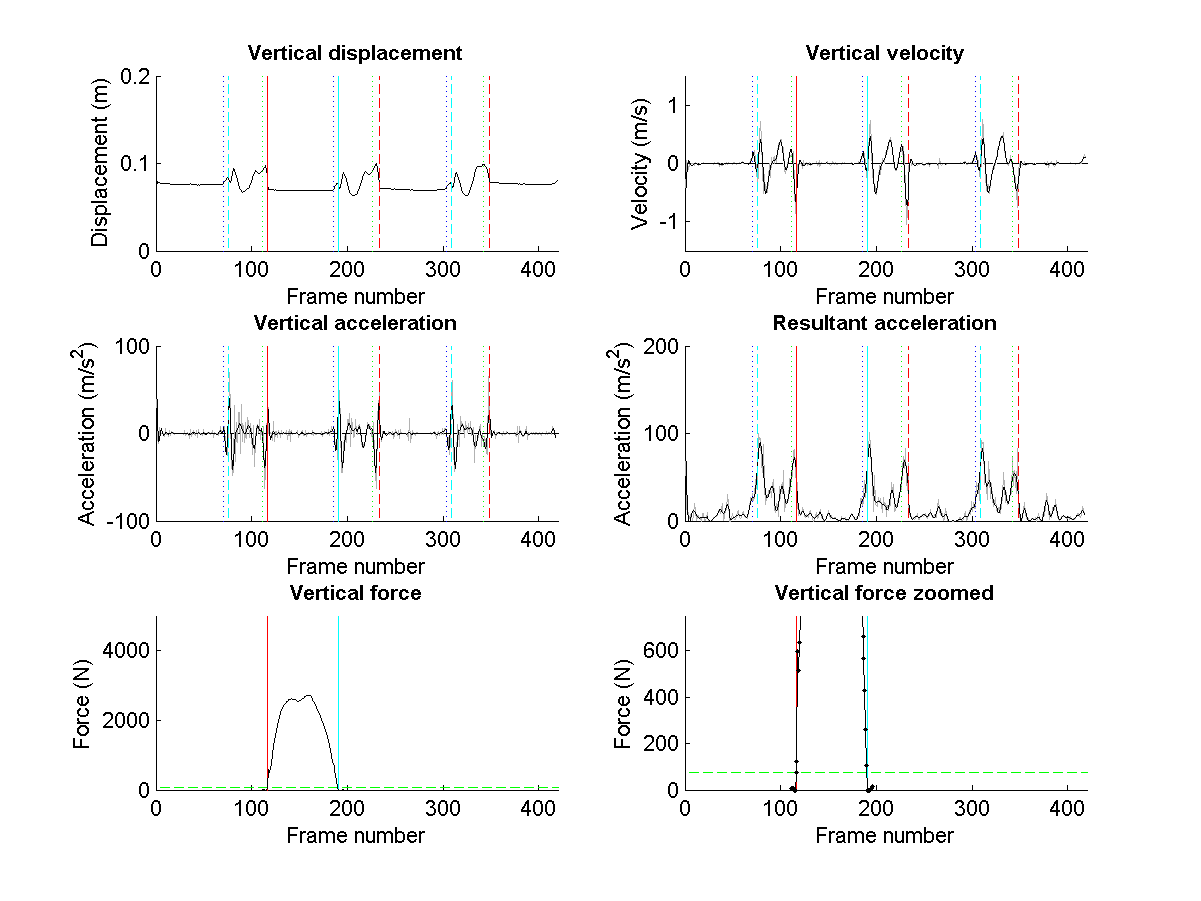

Supplement: Supplemental Information 3 [file peerj-03-783-s003.zip › Suppl figures/Event-based/Event_plot-LF_Horse10_walk11.png]

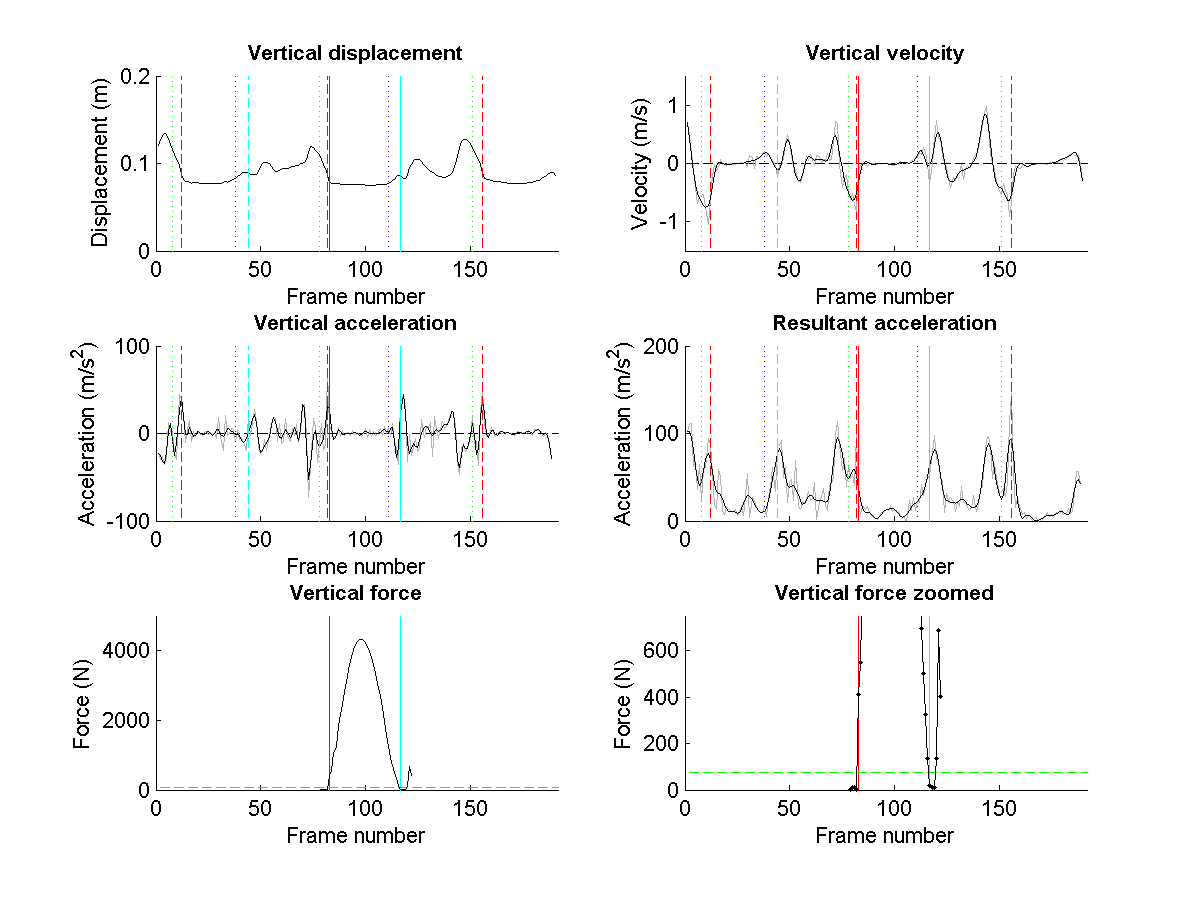

Supplement: Supplemental Information 3 [file peerj-03-783-s003.zip › Suppl figures/Event-based/Event_plot-LF_Horse3_circle_left_trot_03.png]

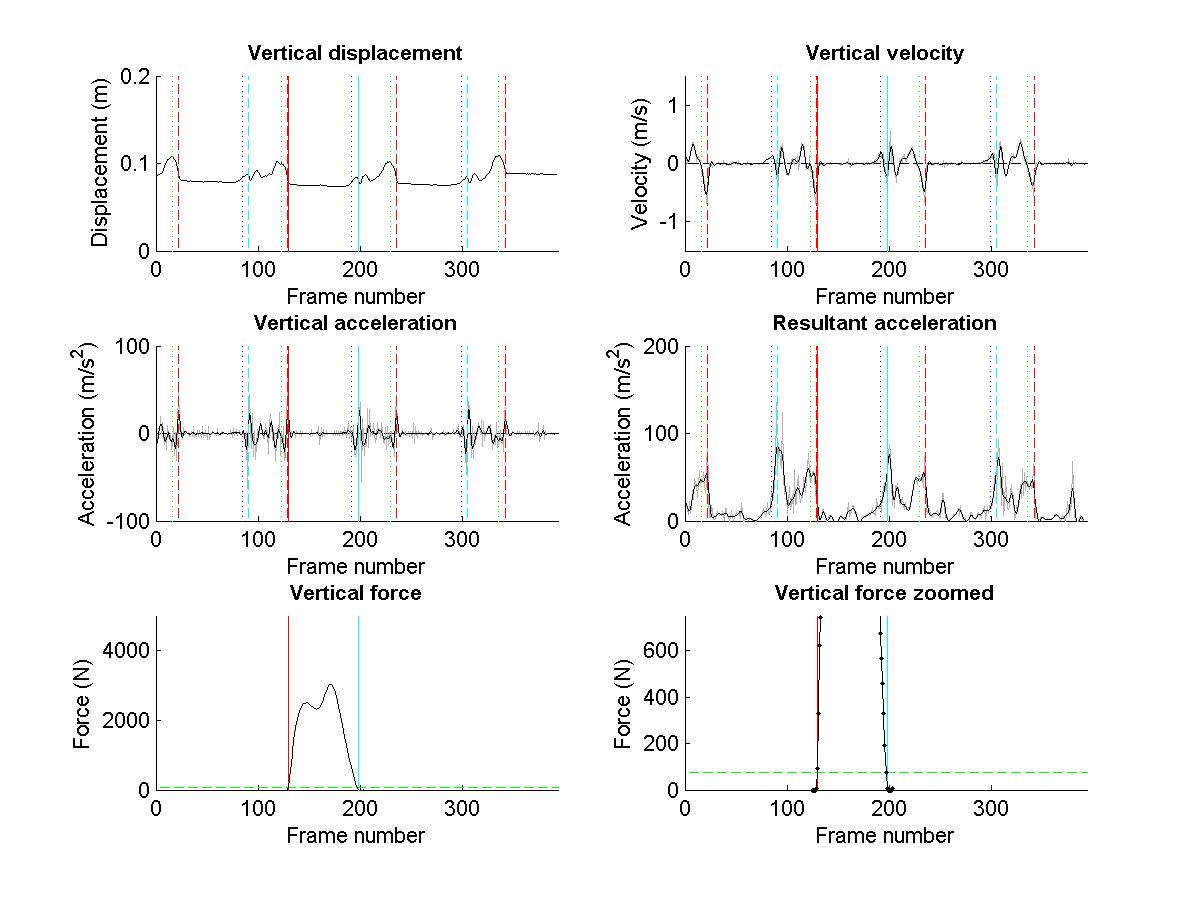

Supplement: Supplemental Information 3 [file peerj-03-783-s003.zip › Suppl figures/Event-based/Event_plot-LF_Horse3_circle_left_walk_04.png]

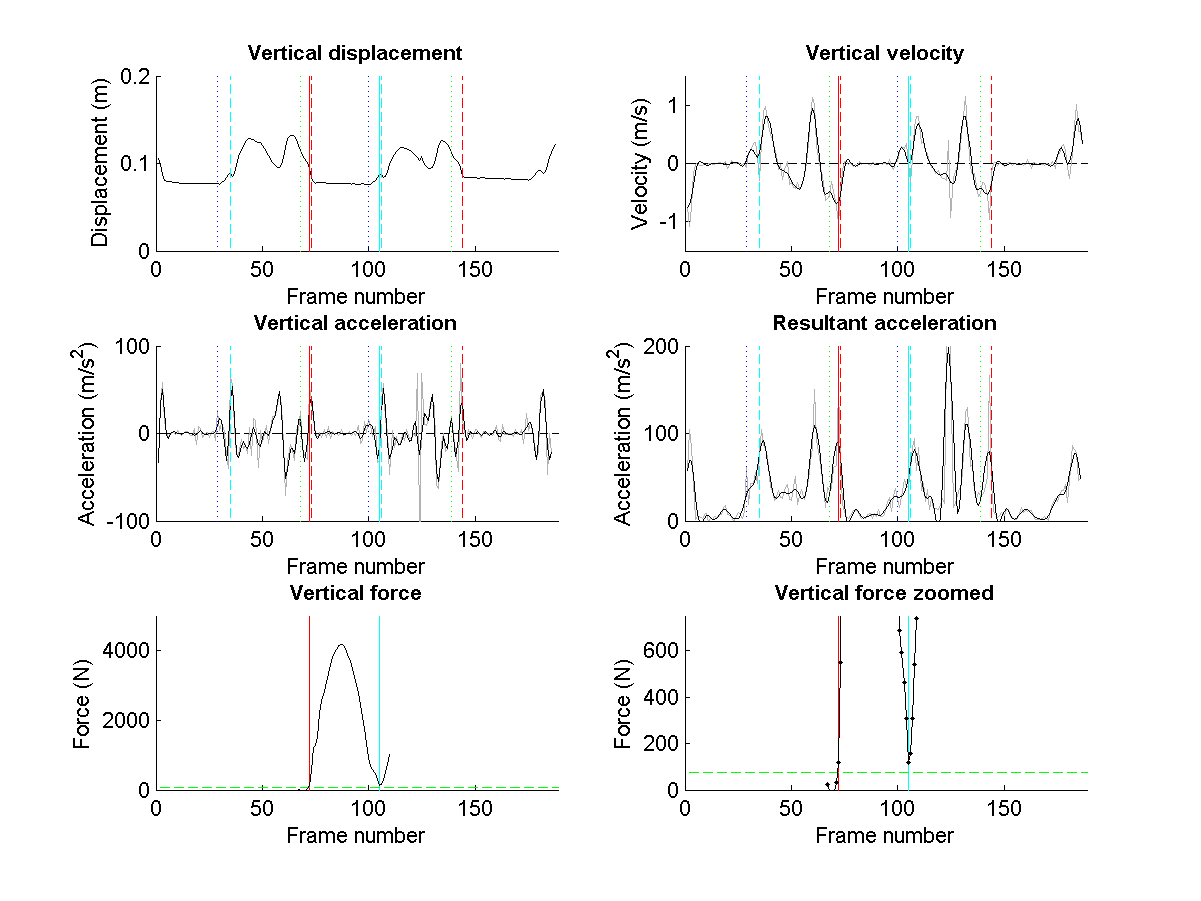

Supplement: Supplemental Information 3 [file peerj-03-783-s003.zip › Suppl figures/Event-based/Event_plot-LF_Horse3_circle_right_trot_02.png]

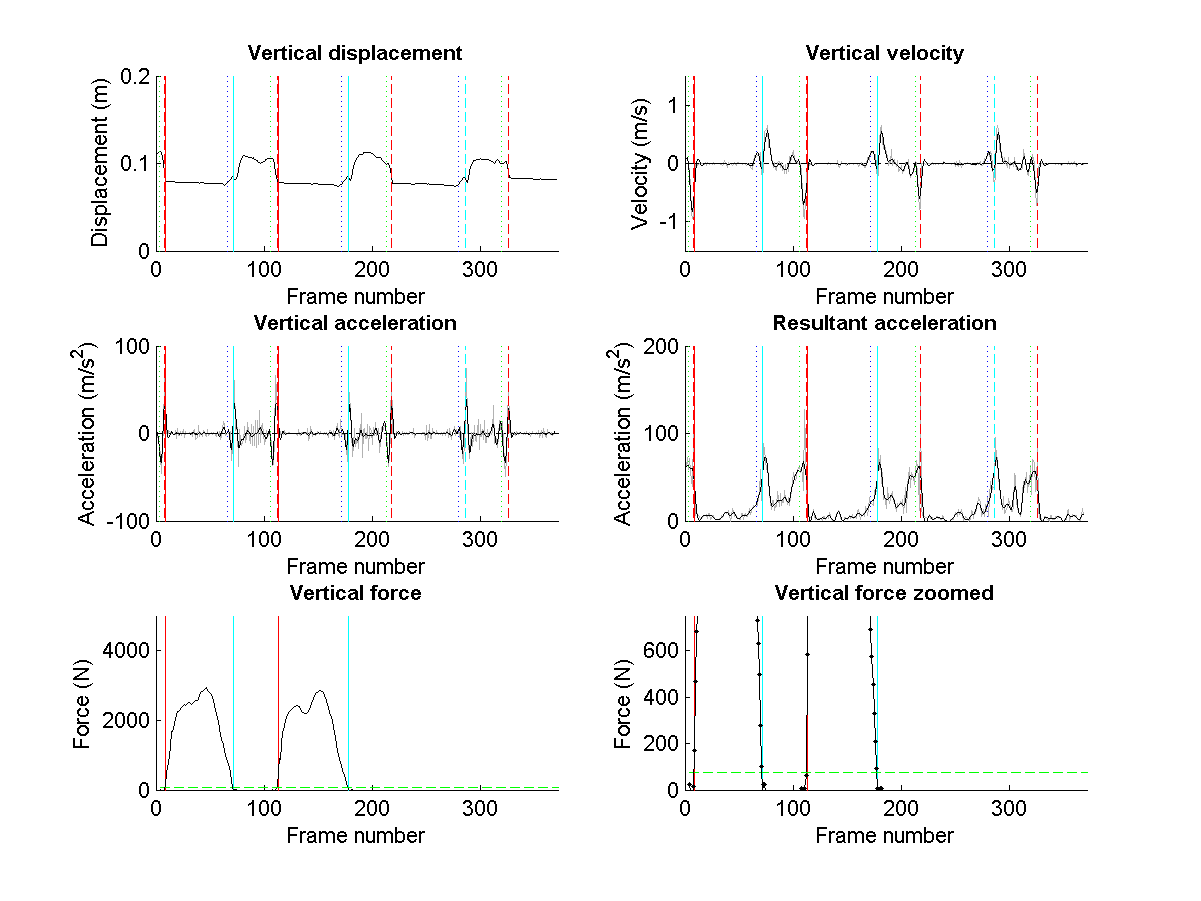

Supplement: Supplemental Information 3 [file peerj-03-783-s003.zip › Suppl figures/Event-based/Event_plot-LF_Horse3_circle_right_walk_08.png]

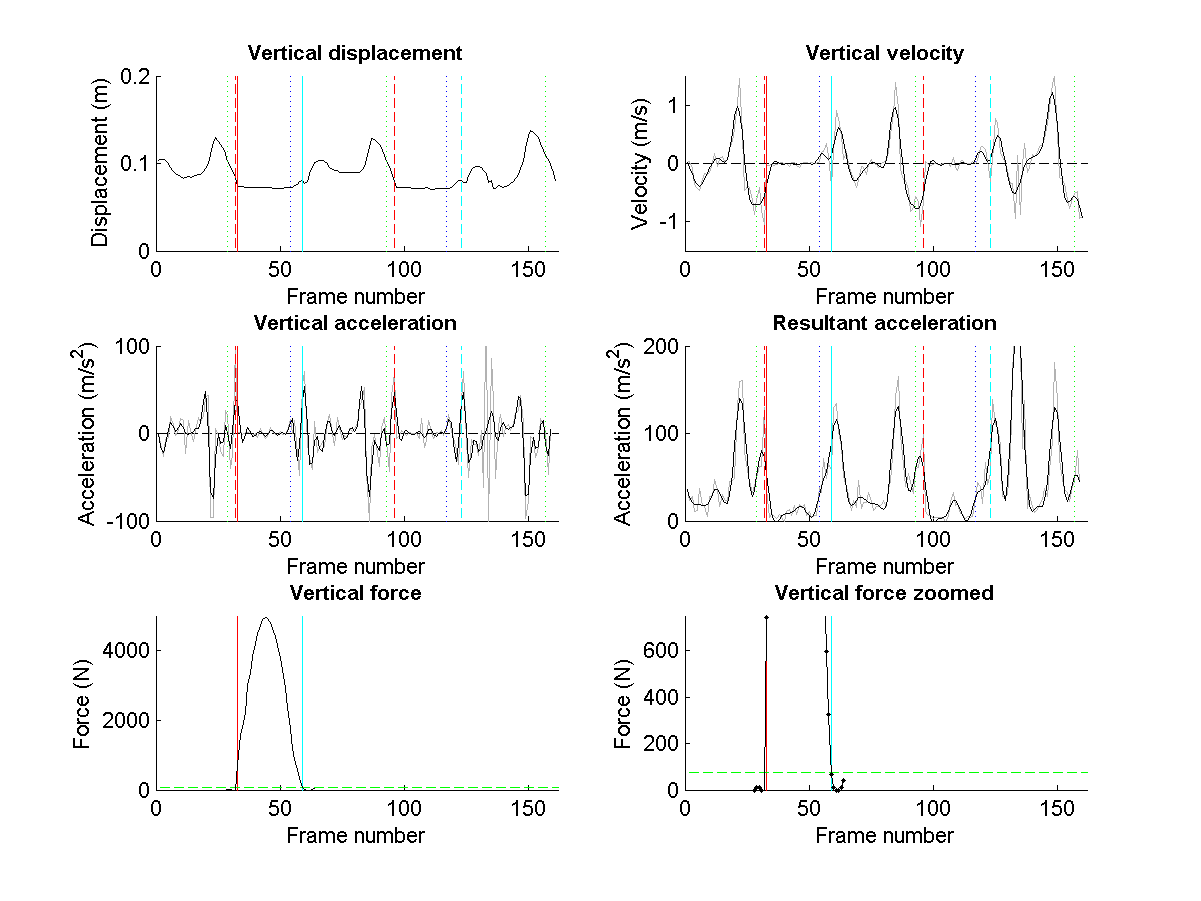

Supplement: Supplemental Information 3 [file peerj-03-783-s003.zip › Suppl figures/Event-based/Event_plot-LF_Horse3_trot_12.png]

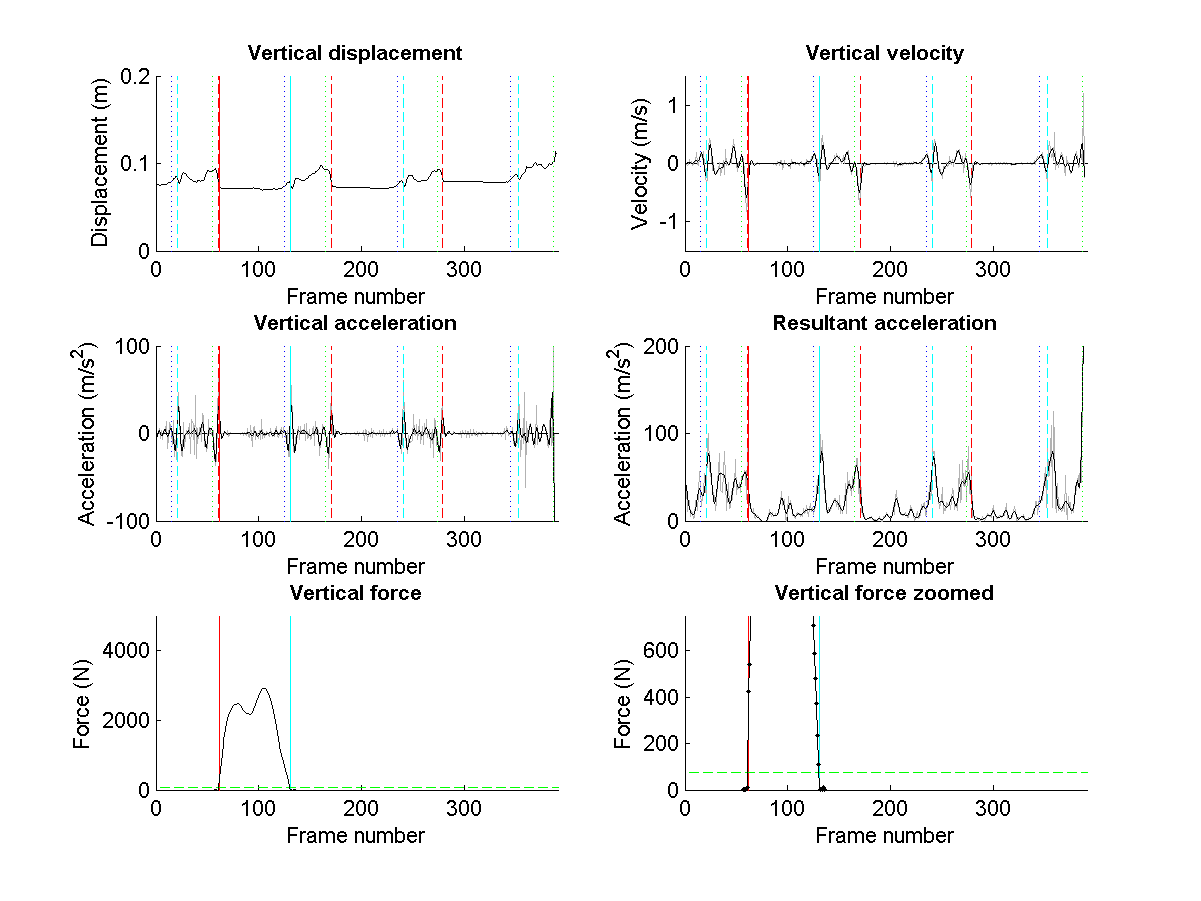

Supplement: Supplemental Information 3 [file peerj-03-783-s003.zip › Suppl figures/Event-based/Event_plot-LF_Horse3_walk_03.png]

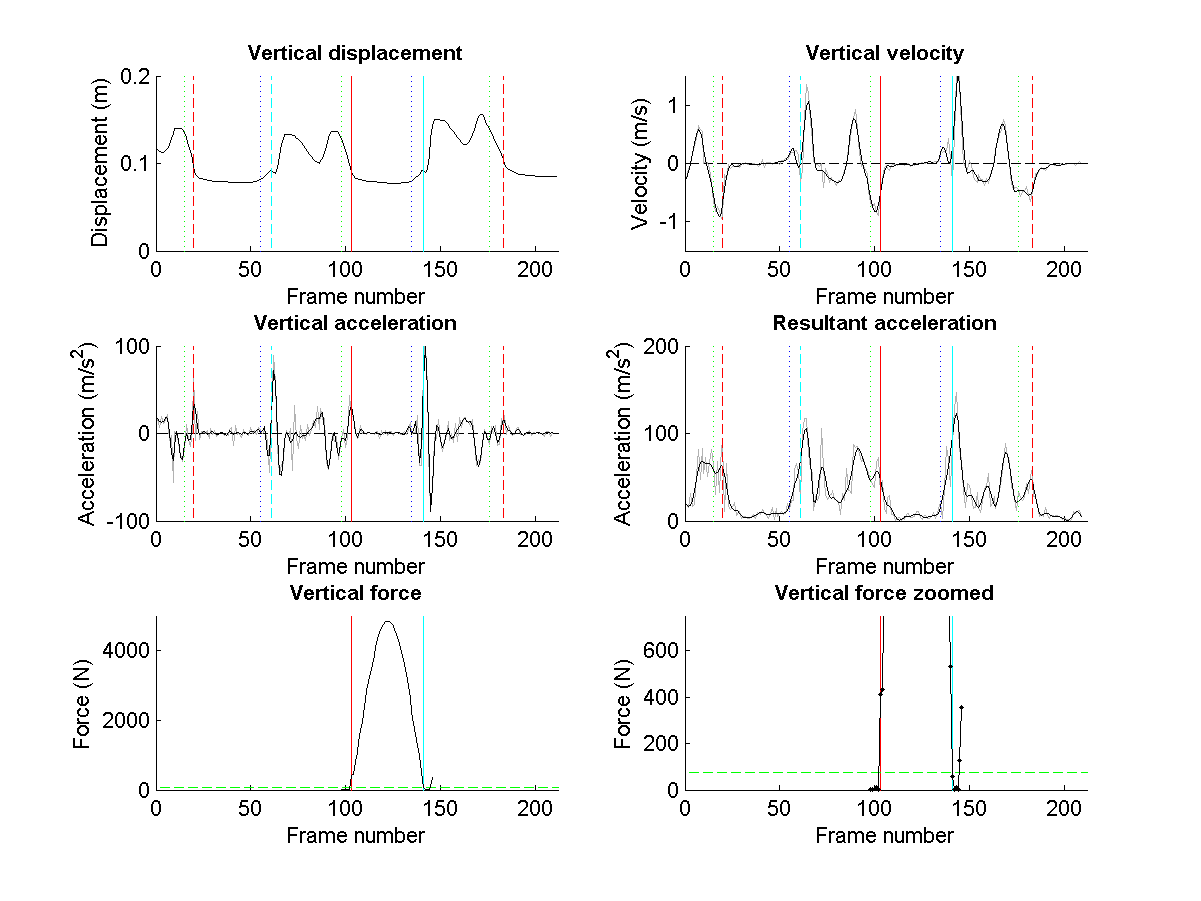

Supplement: Supplemental Information 3 [file peerj-03-783-s003.zip › Suppl figures/Event-based/Event_plot-LF_Horse5_circle_left_trot_03.png]

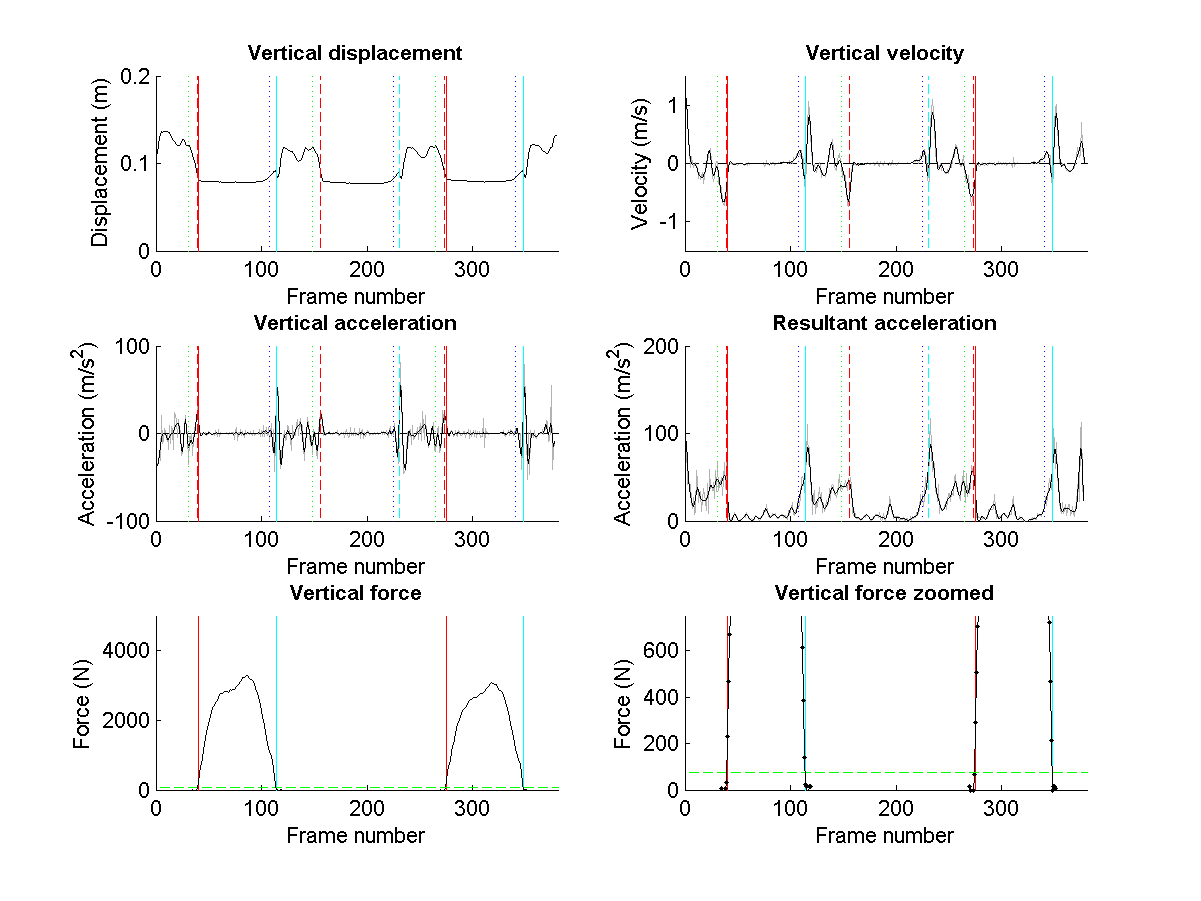

Supplement: Supplemental Information 3 [file peerj-03-783-s003.zip › Suppl figures/Event-based/Event_plot-LF_Horse5_circle_left_walk_03.png]

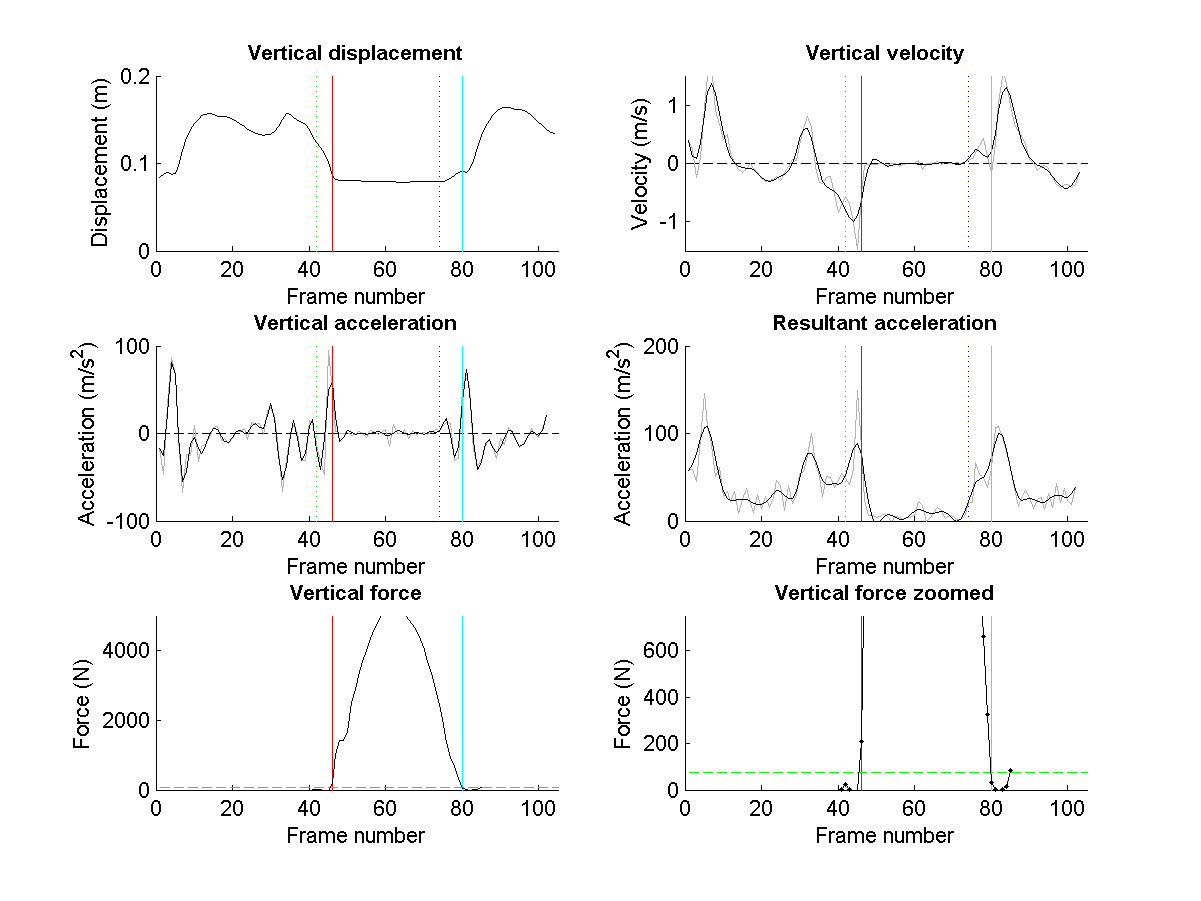

Supplement: Supplemental Information 3 [file peerj-03-783-s003.zip › Suppl figures/Event-based/Event_plot-LF_Horse5_circle_right_trot_02.png]

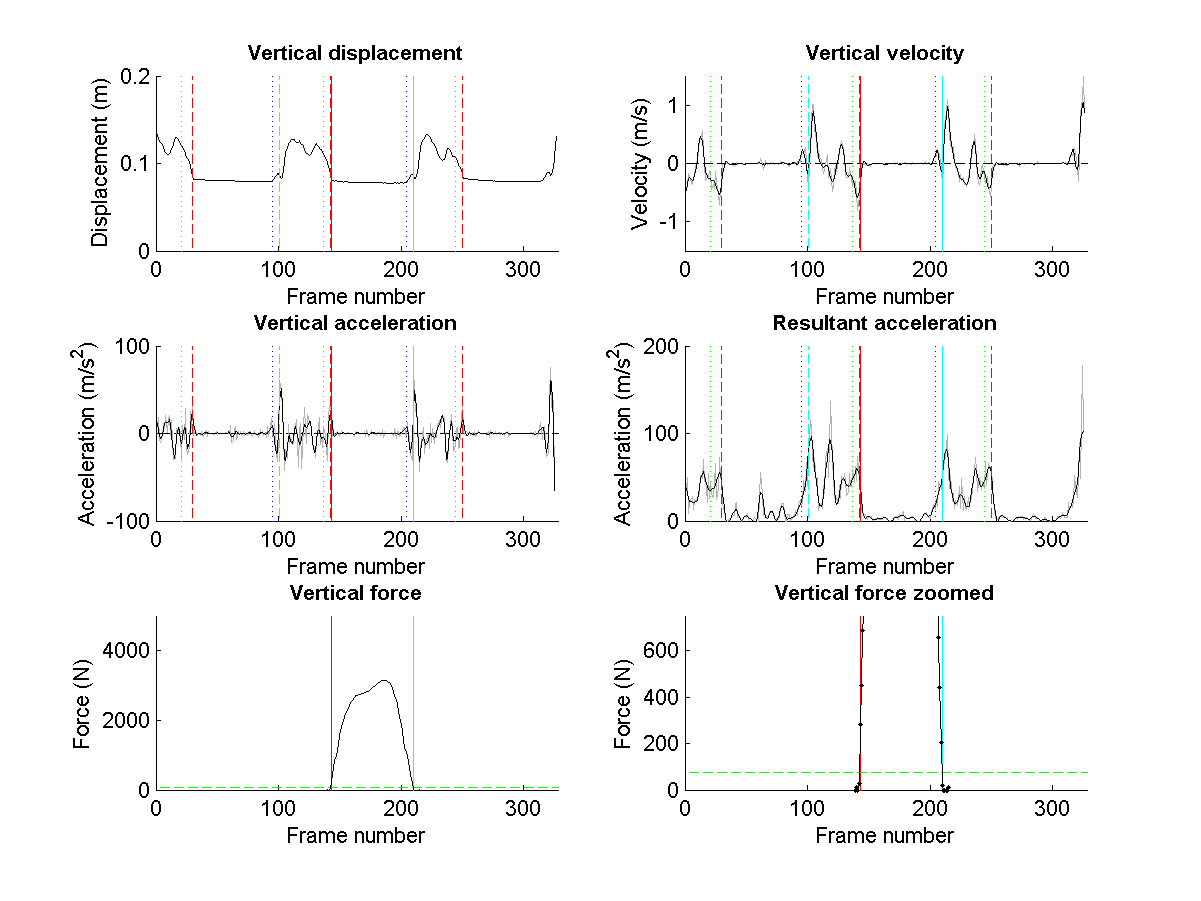

Supplement: Supplemental Information 3 [file peerj-03-783-s003.zip › Suppl figures/Event-based/Event_plot-LF_Horse5_circle_right_walk_02.png]

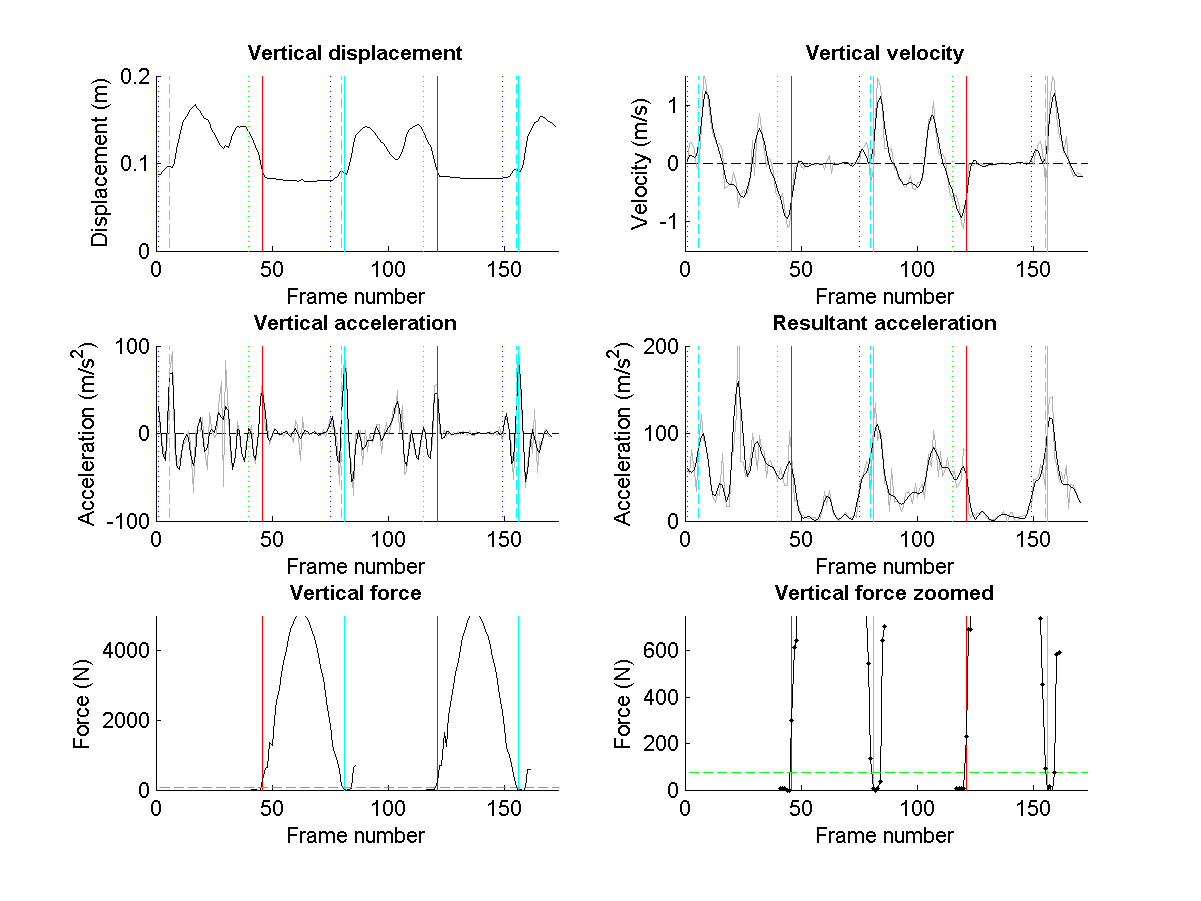

Supplement: Supplemental Information 3 [file peerj-03-783-s003.zip › Suppl figures/Event-based/Event_plot-LF_Horse5_trot_06.png]

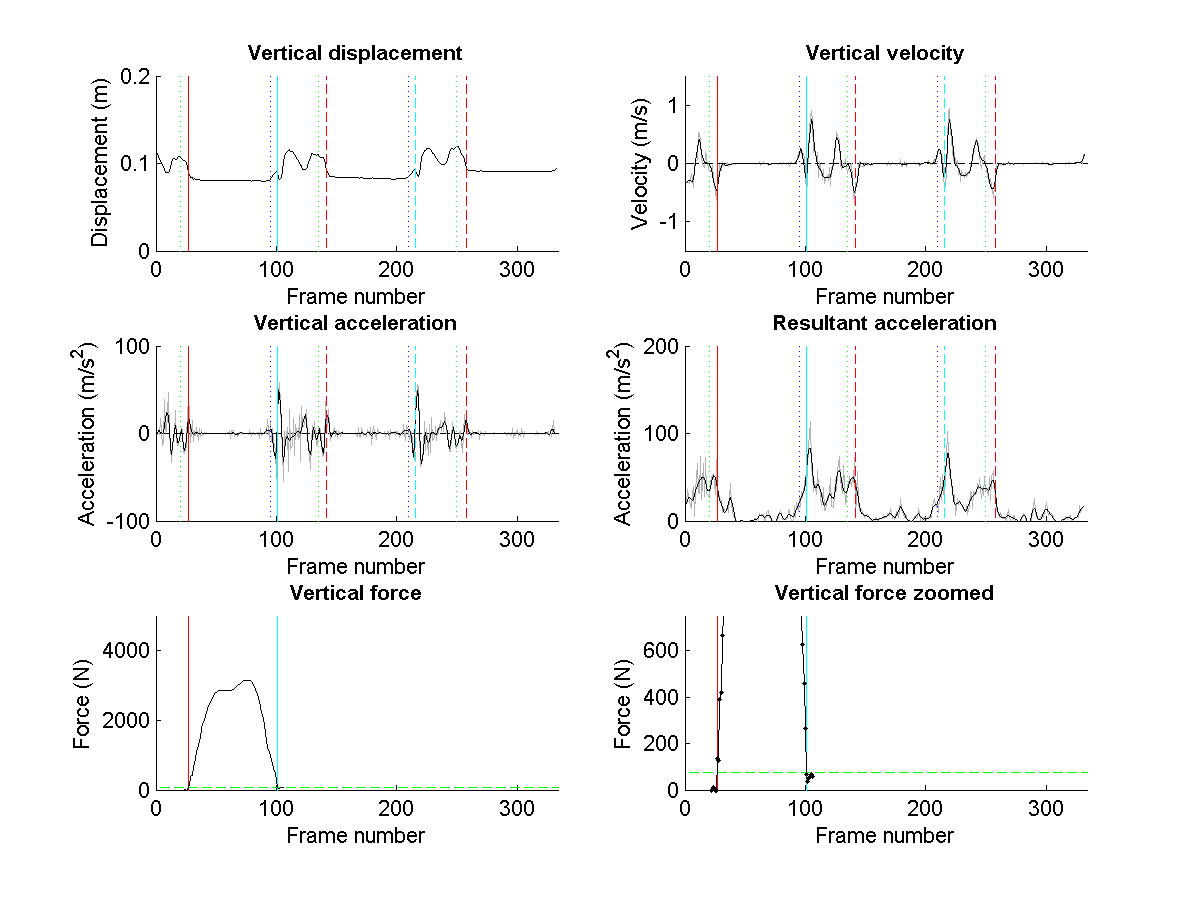

Supplement: Supplemental Information 3 [file peerj-03-783-s003.zip › Suppl figures/Event-based/Event_plot-LF_Horse5_walk_10.png]

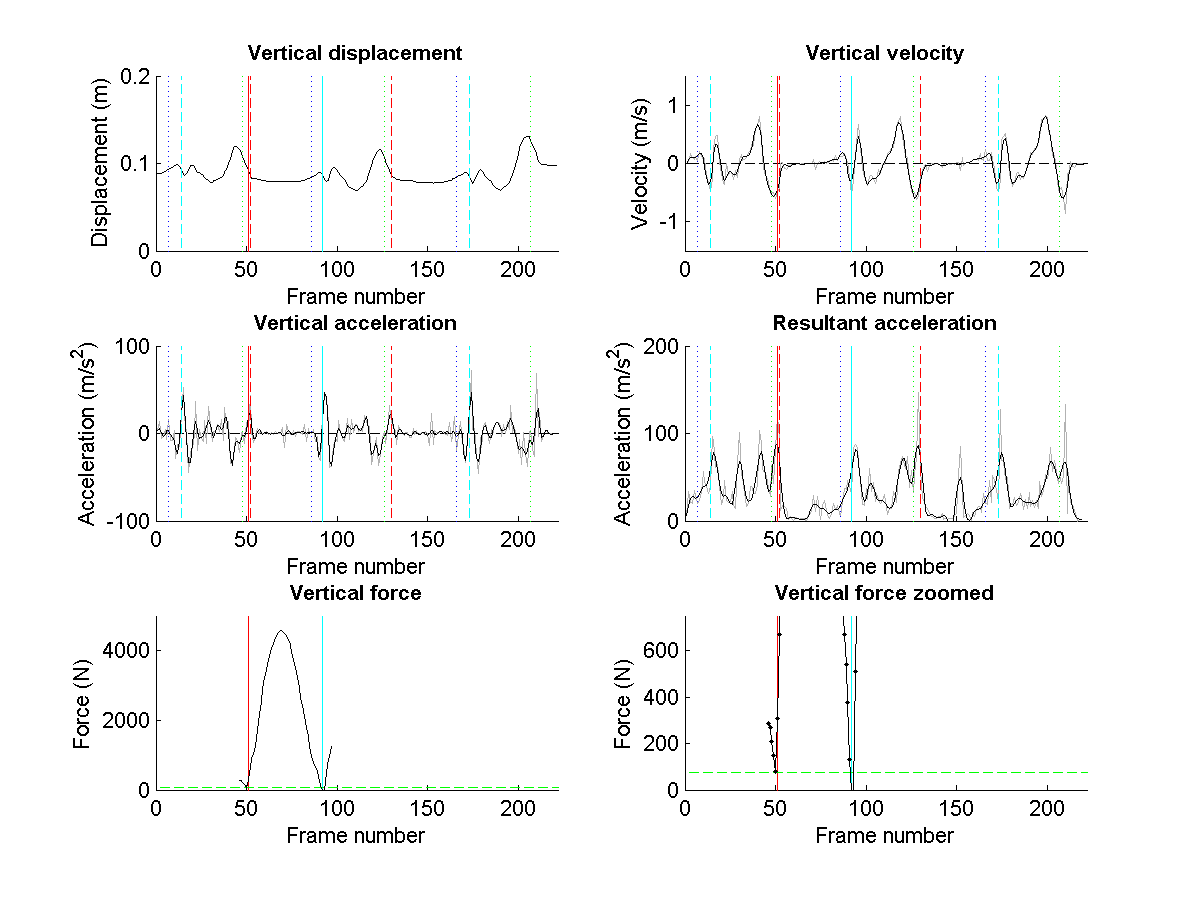

Supplement: Supplemental Information 3 [file peerj-03-783-s003.zip › Suppl figures/Event-based/Event_plot-LF_Horse8_circle_left_trot_11.png]

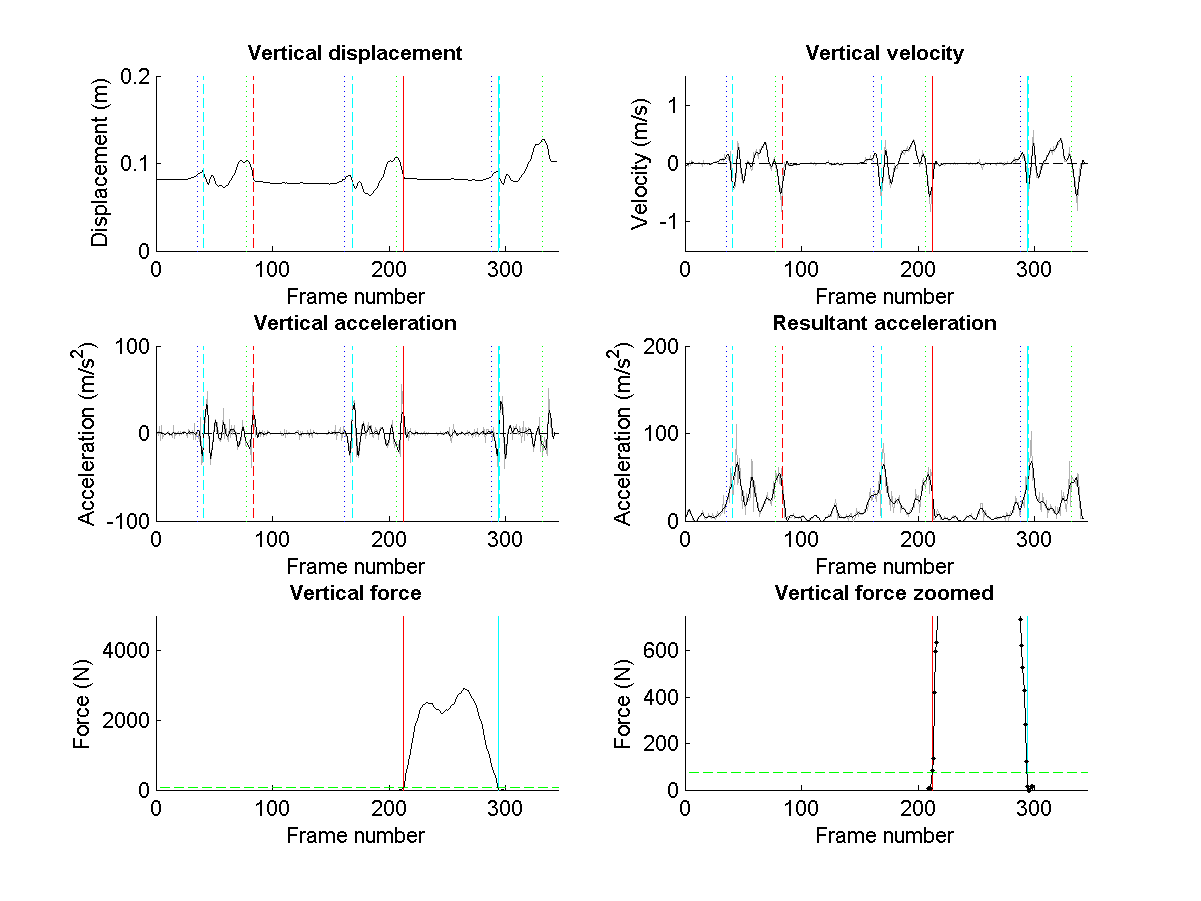

Supplement: Supplemental Information 3 [file peerj-03-783-s003.zip › Suppl figures/Event-based/Event_plot-LF_Horse8_circle_left_walk_01.png]

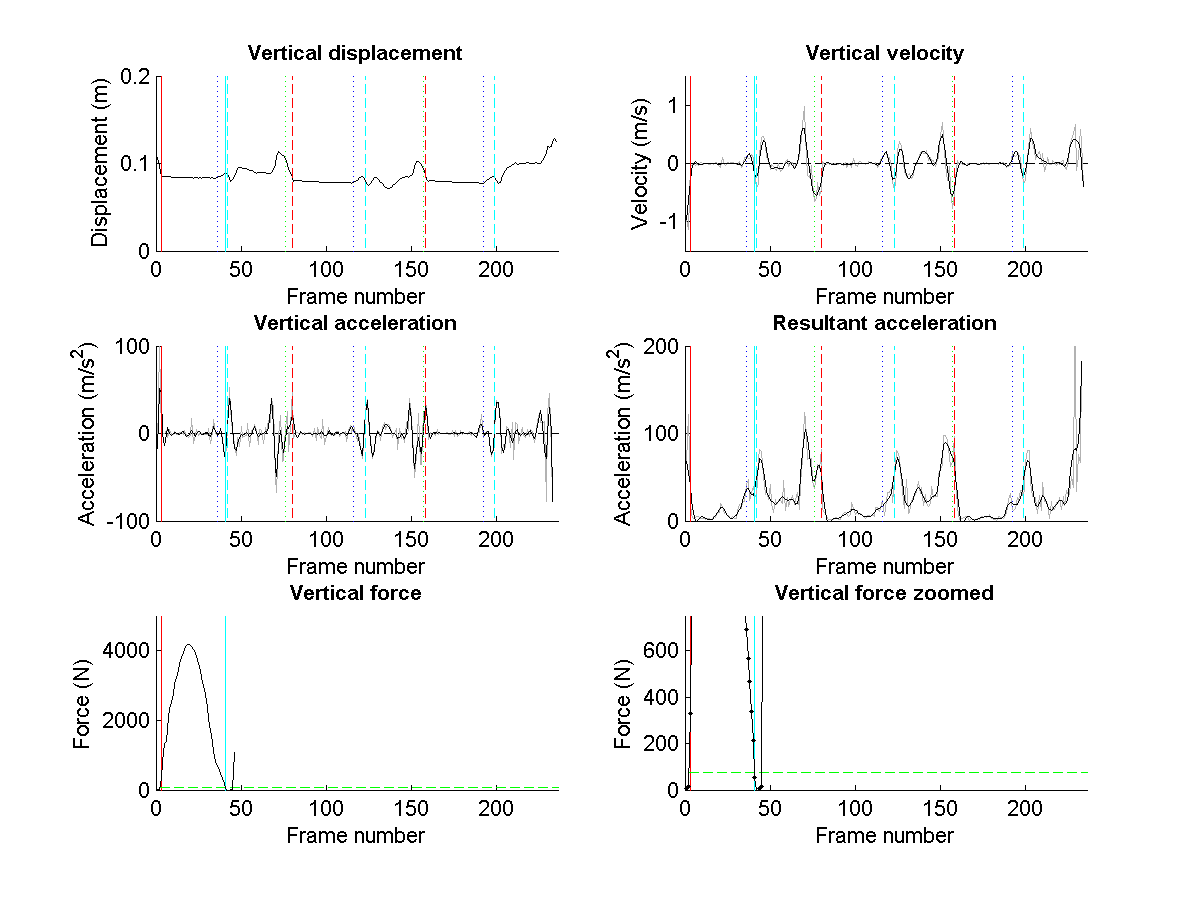

Supplement: Supplemental Information 3 [file peerj-03-783-s003.zip › Suppl figures/Event-based/Event_plot-LF_Horse8_circle_right_trot03.png]

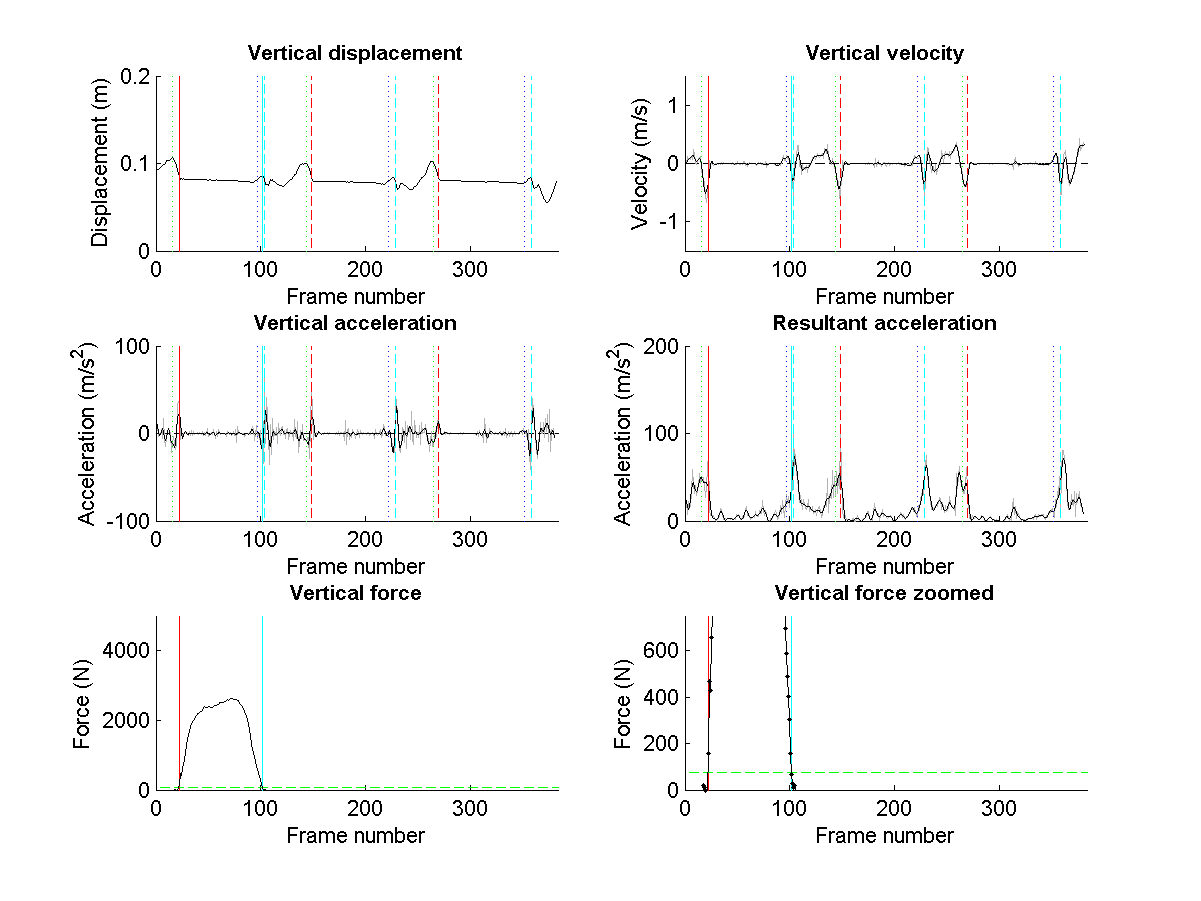

Supplement: Supplemental Information 3 [file peerj-03-783-s003.zip › Suppl figures/Event-based/Event_plot-LF_Horse8_circle_right_walk_02.png]

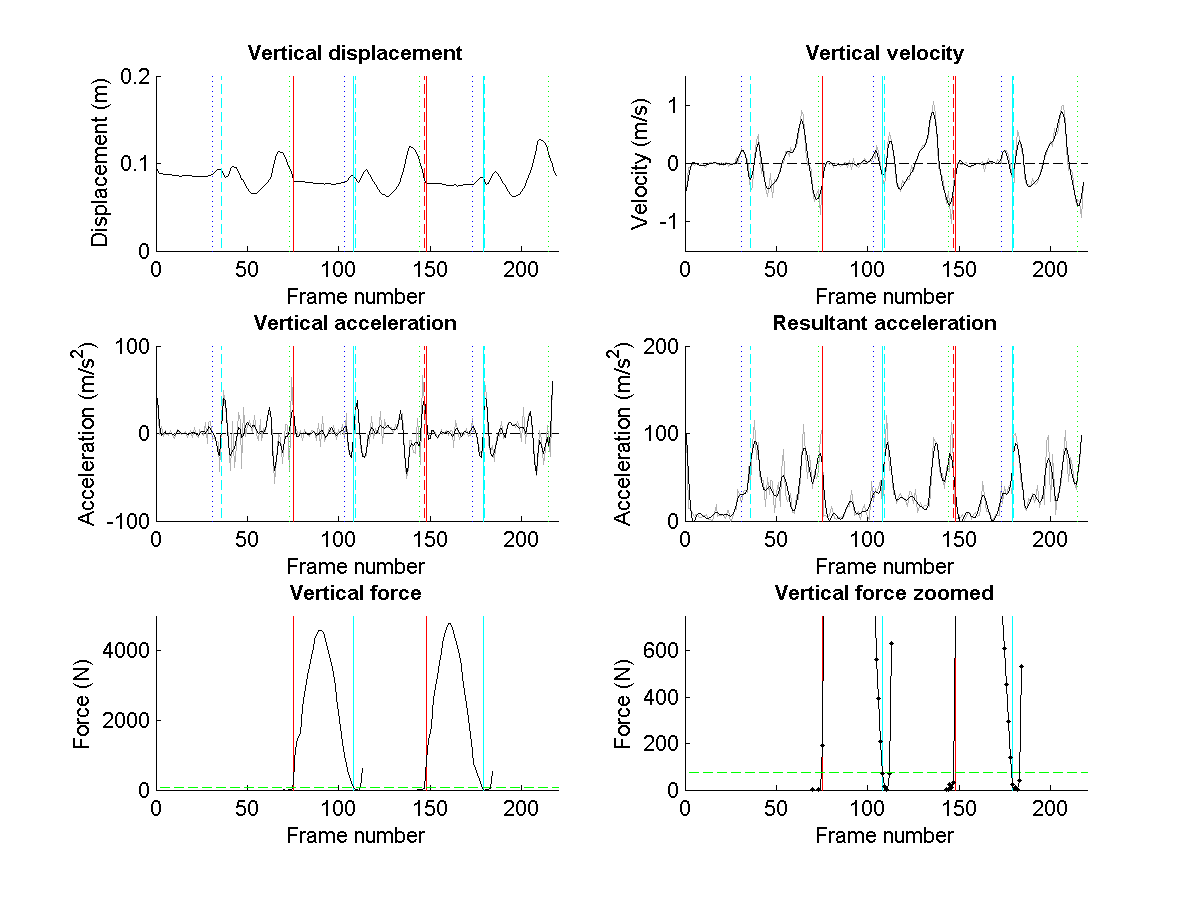

Supplement: Supplemental Information 3 [file peerj-03-783-s003.zip › Suppl figures/Event-based/Event_plot-LF_Horse8_trot_10.png]

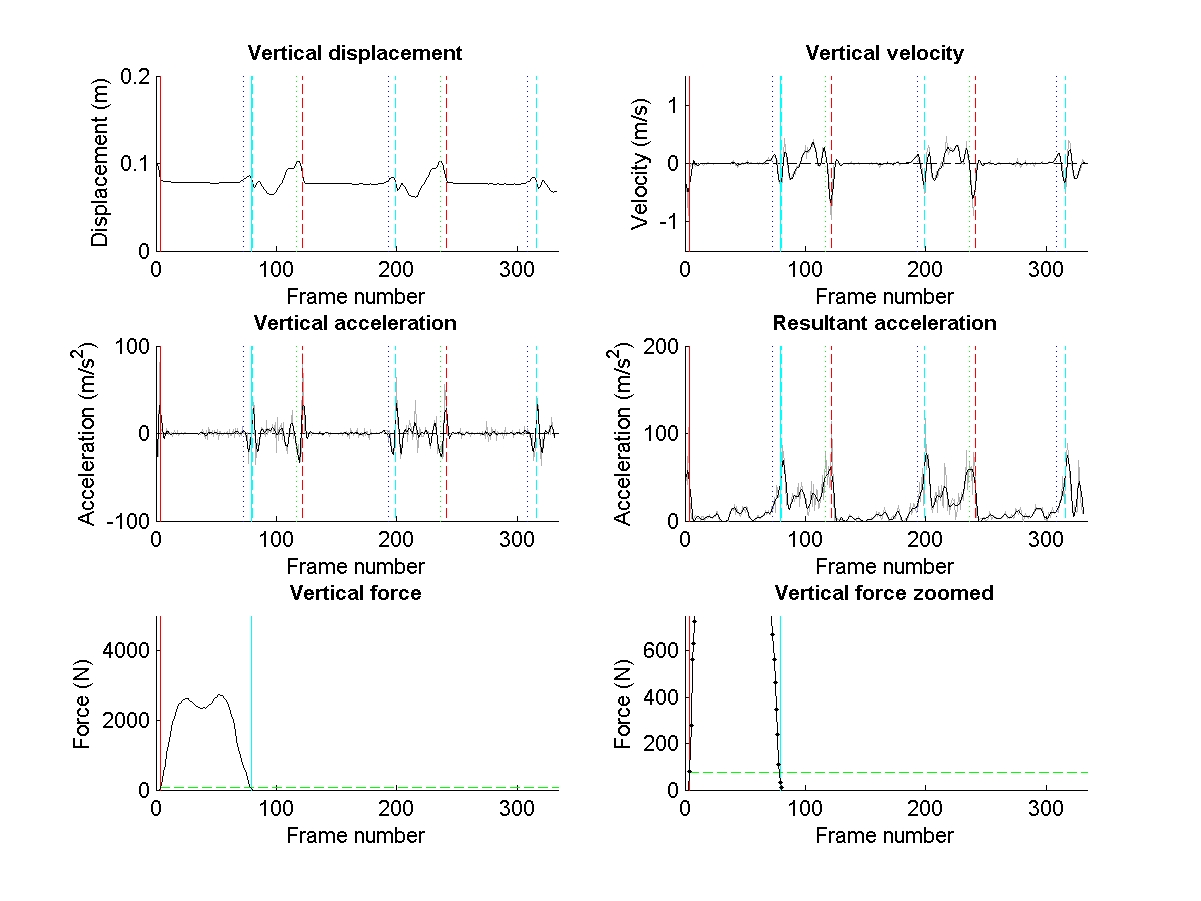

Supplement: Supplemental Information 3 [file peerj-03-783-s003.zip › Suppl figures/Event-based/Event_plot-LF_Horse8_walk_01.png]

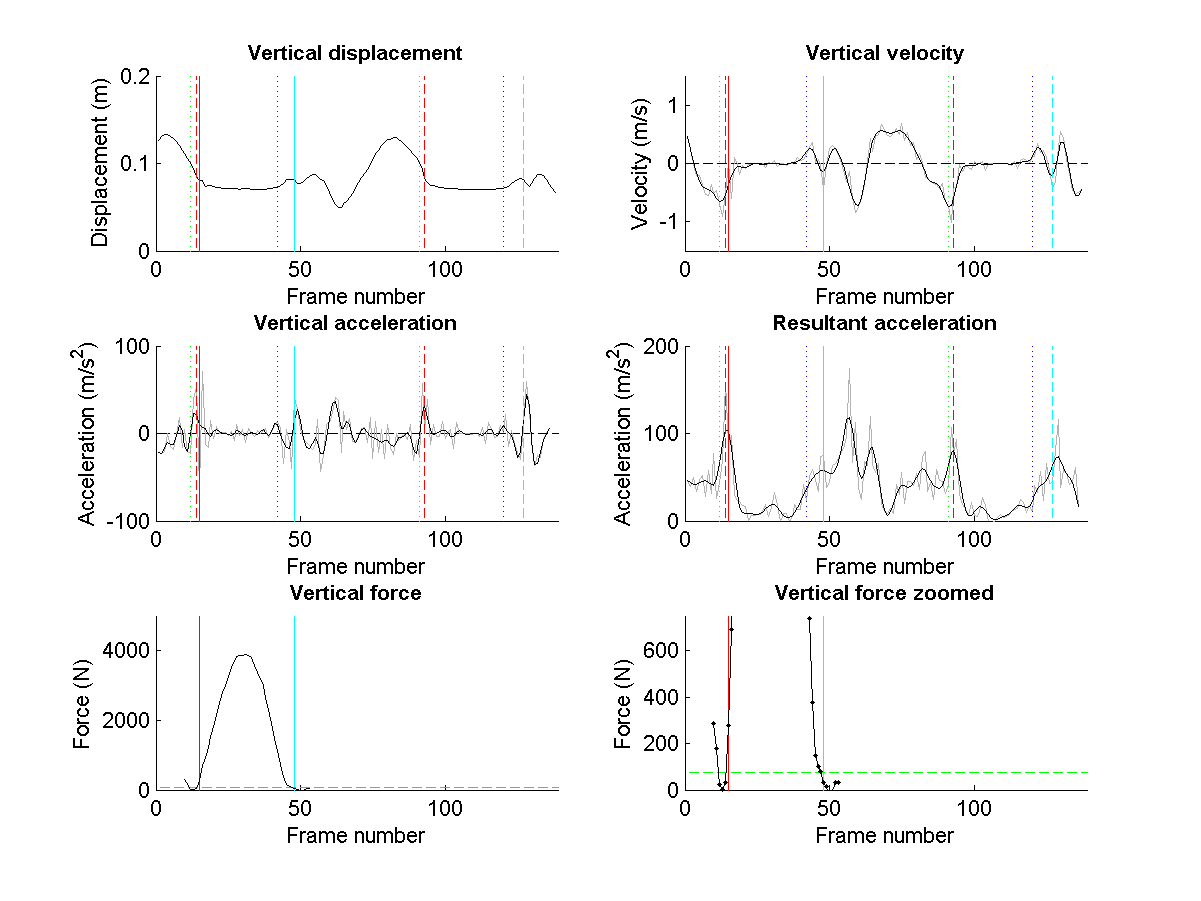

Supplement: Supplemental Information 3 [file peerj-03-783-s003.zip › Suppl figures/Event-based/Event_plot-LH_Horse10_circle_left_trot_03.png]

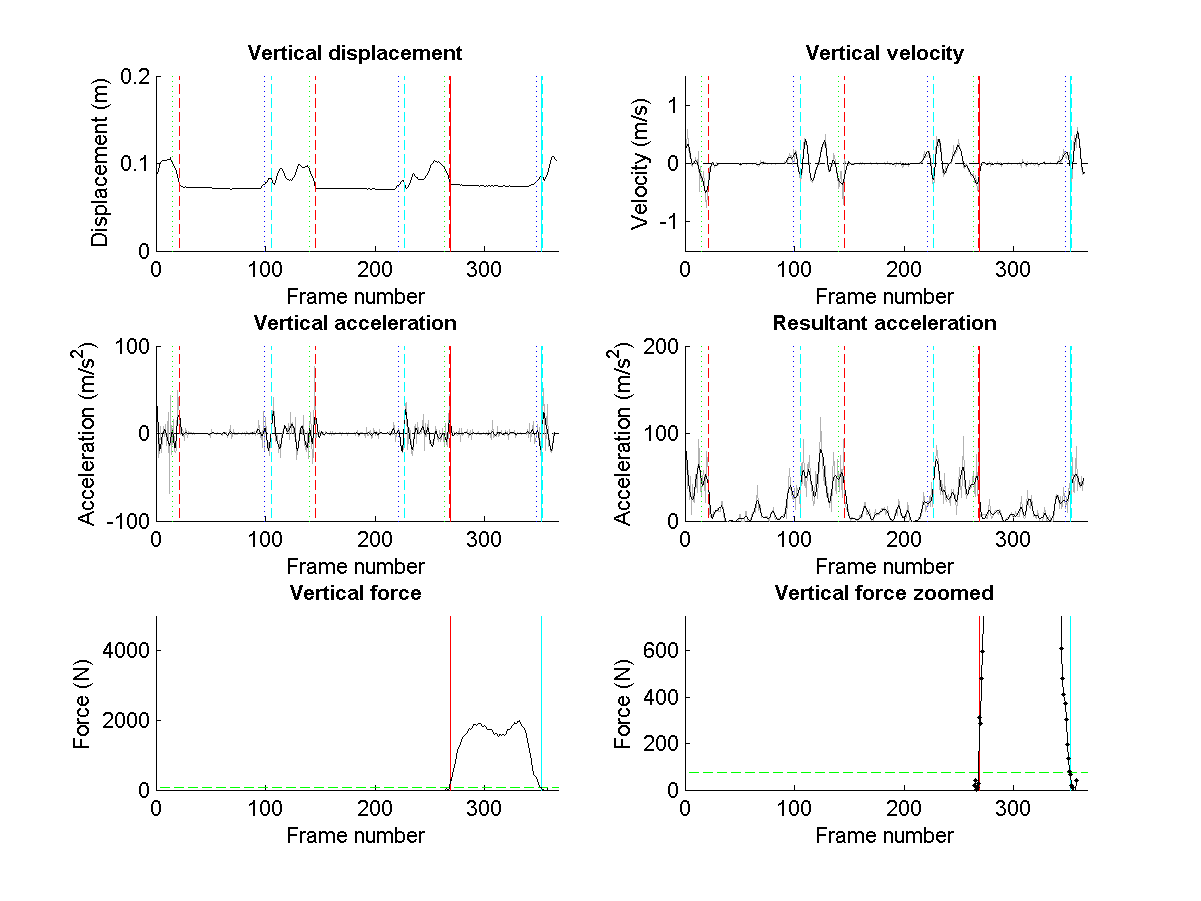

Supplement: Supplemental Information 3 [file peerj-03-783-s003.zip › Suppl figures/Event-based/Event_plot-LH_Horse10_circle_left_walk_02.png]

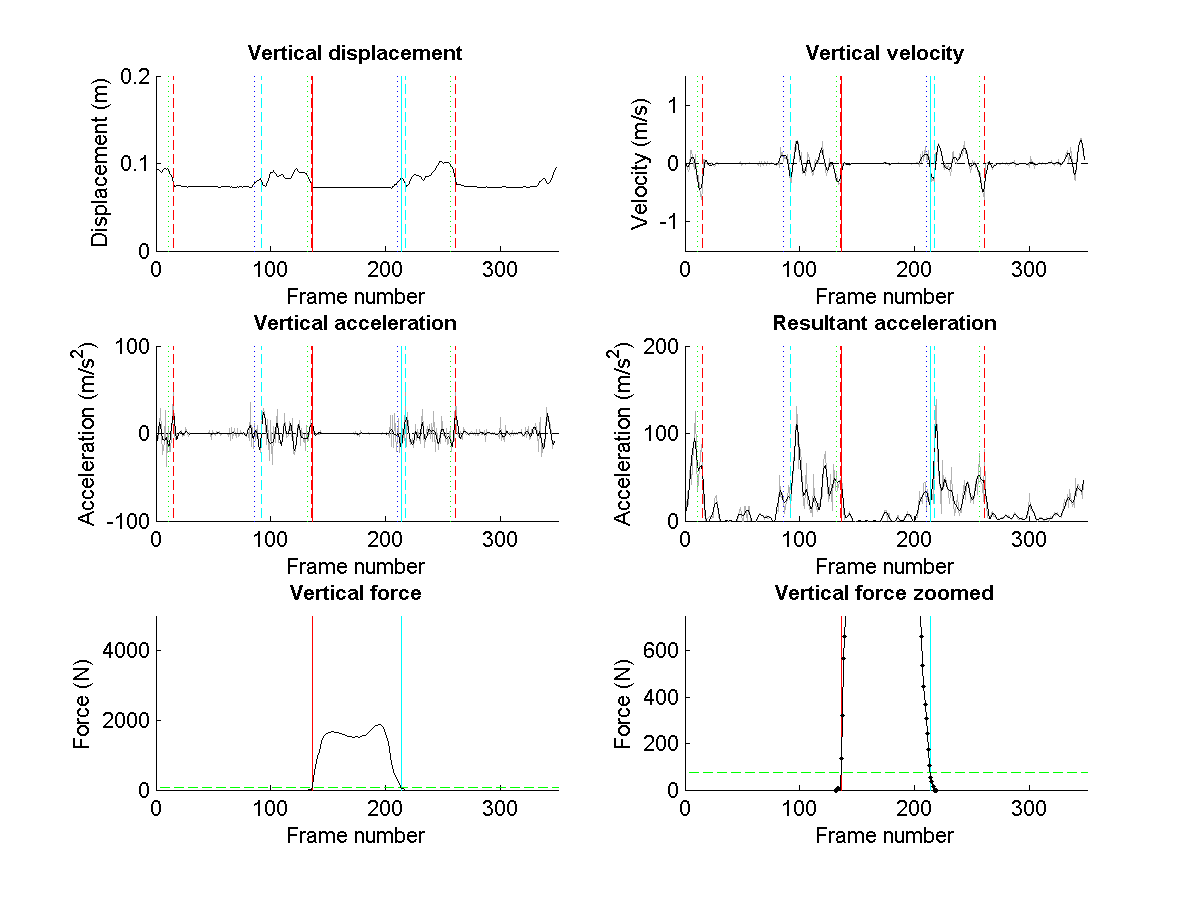

Supplement: Supplemental Information 3 [file peerj-03-783-s003.zip › Suppl figures/Event-based/Event_plot-LH_Horse10_circle_right_walk_08.png]

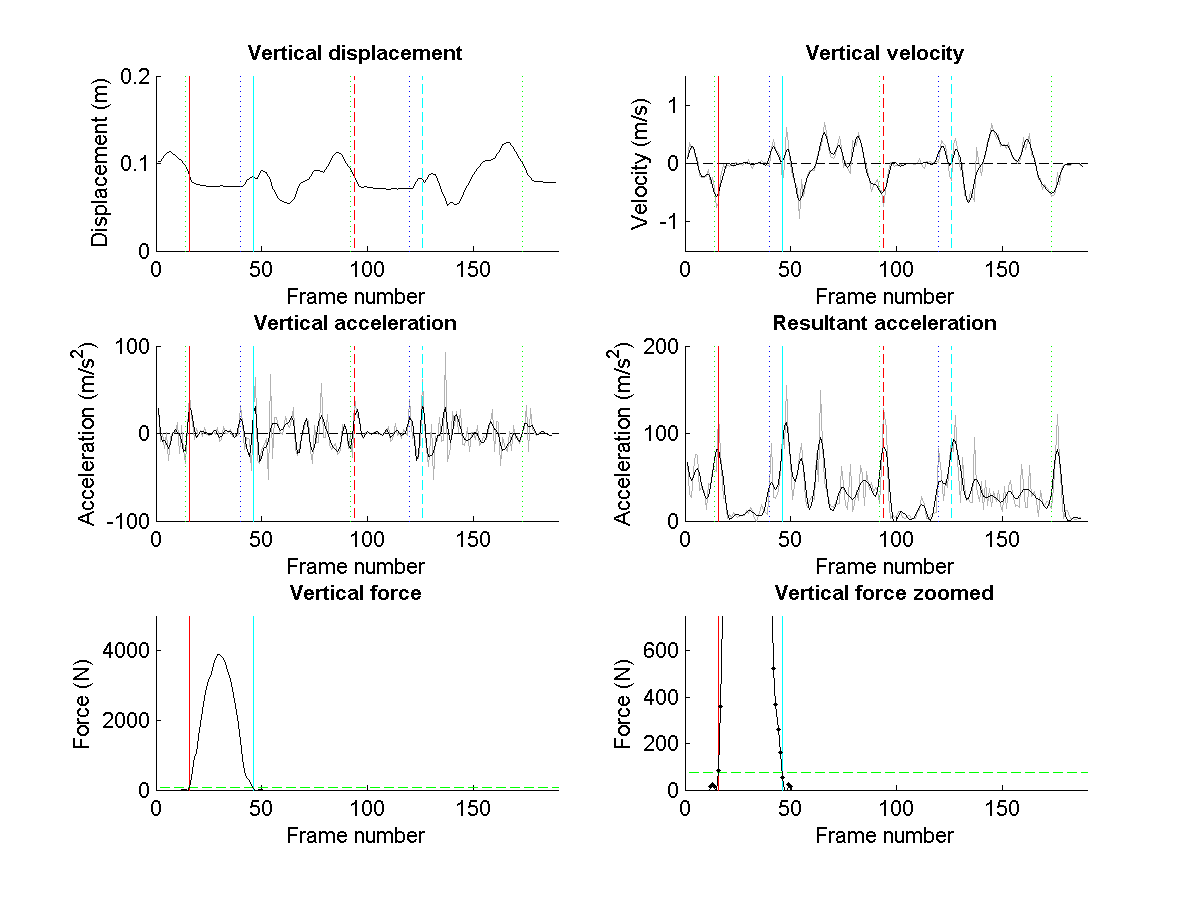

Supplement: Supplemental Information 3 [file peerj-03-783-s003.zip › Suppl figures/Event-based/Event_plot-LH_Horse10_circle_rightt_trot_03.png]

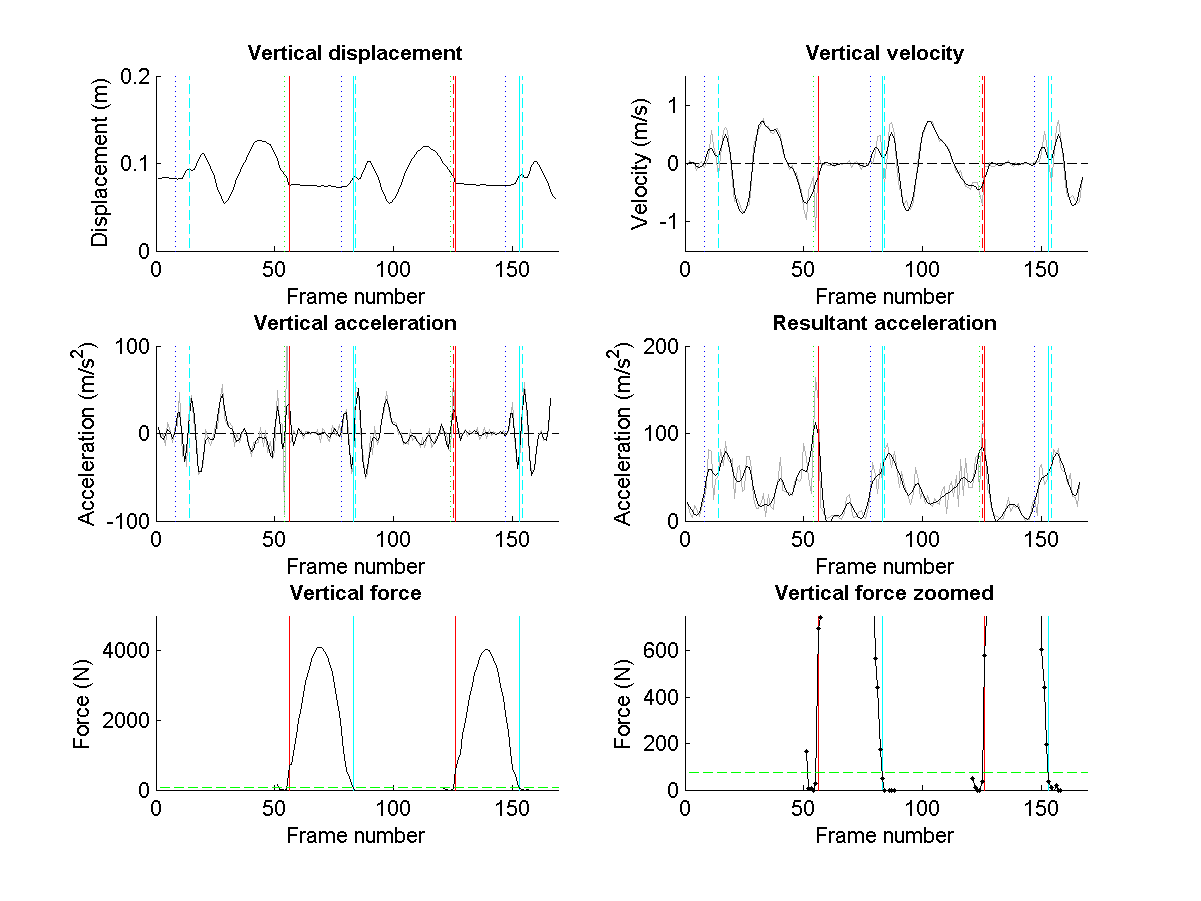

Supplement: Supplemental Information 3 [file peerj-03-783-s003.zip › Suppl figures/Event-based/Event_plot-LH_Horse10_trot02.png]

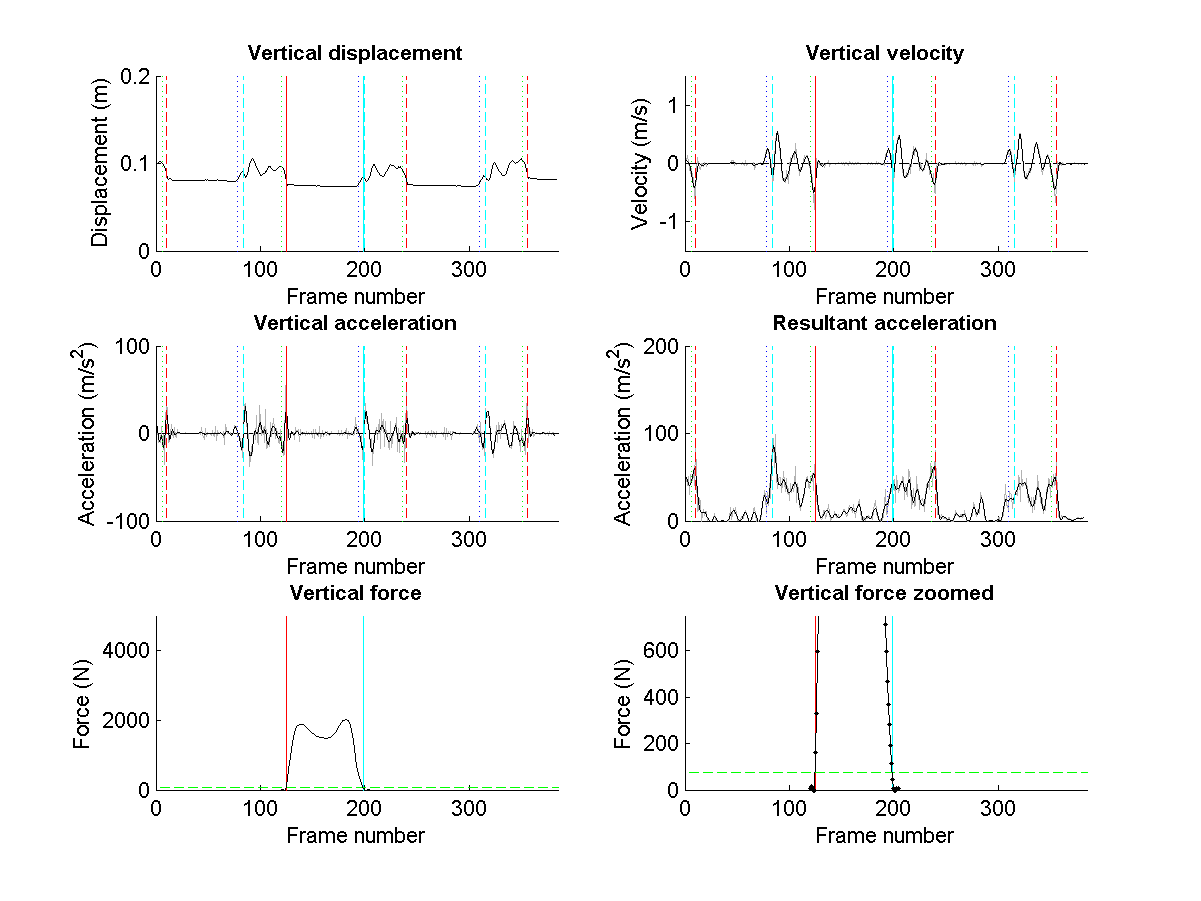

Supplement: Supplemental Information 3 [file peerj-03-783-s003.zip › Suppl figures/Event-based/Event_plot-LH_Horse10_walk11.png]

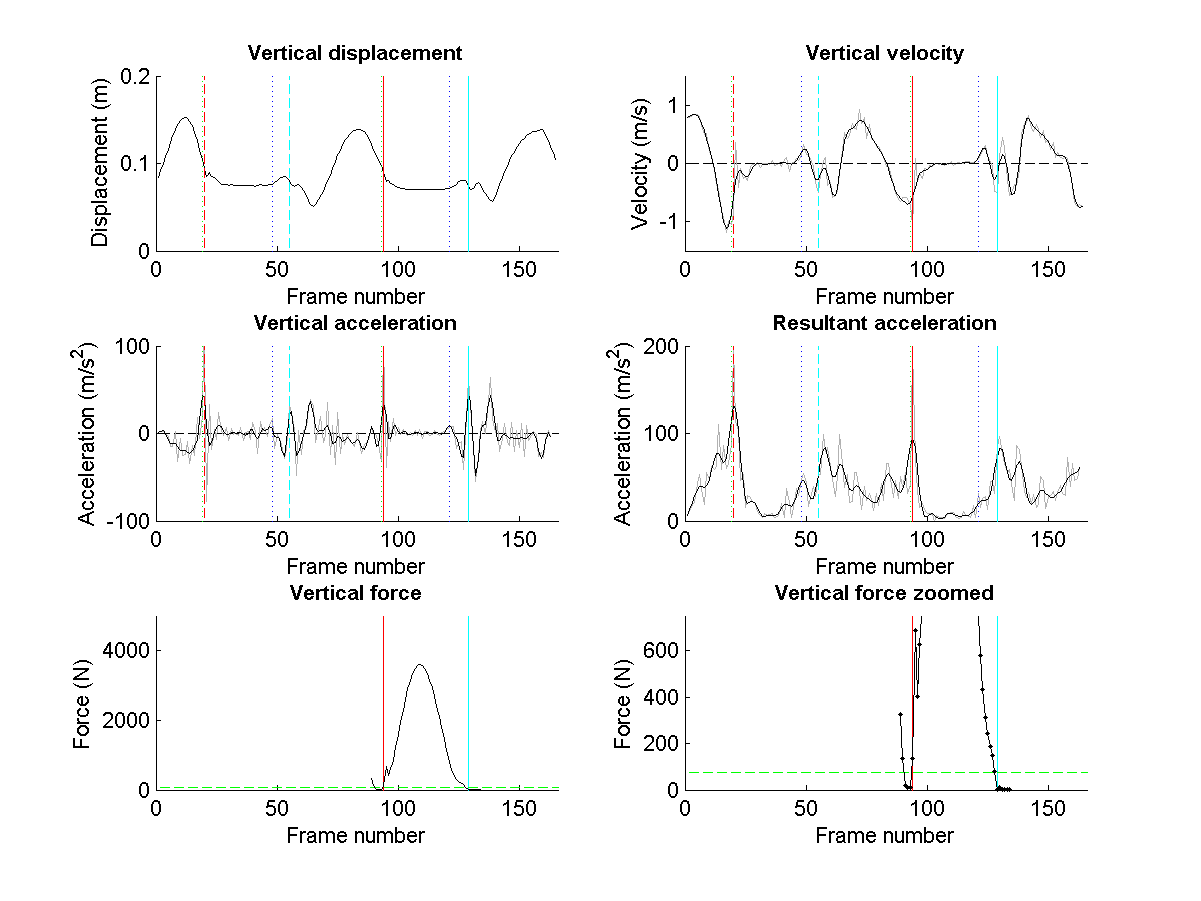

Supplement: Supplemental Information 3 [file peerj-03-783-s003.zip › Suppl figures/Event-based/Event_plot-LH_Horse3_circle_left_trot_3.png]

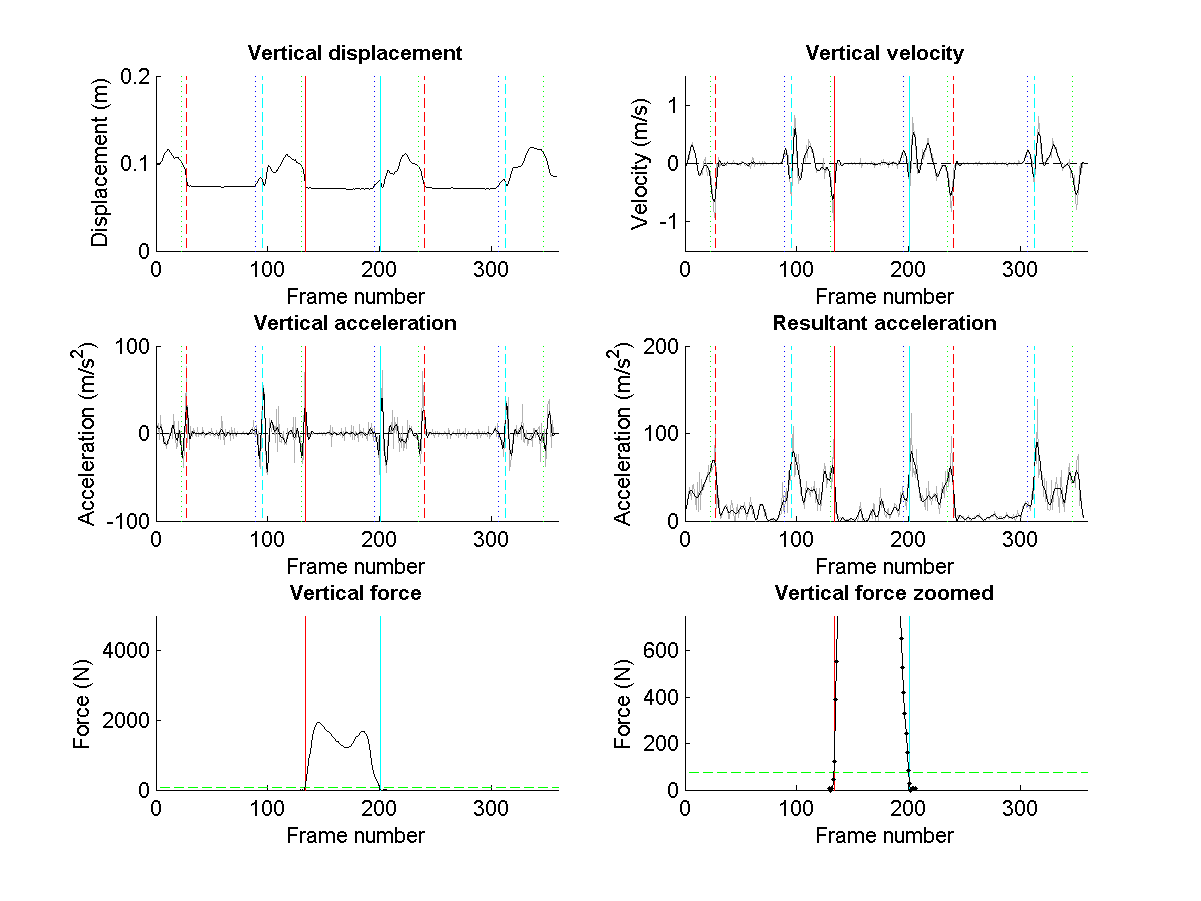

Supplement: Supplemental Information 3 [file peerj-03-783-s003.zip › Suppl figures/Event-based/Event_plot-LH_Horse3_circle_left_walk_04.png]

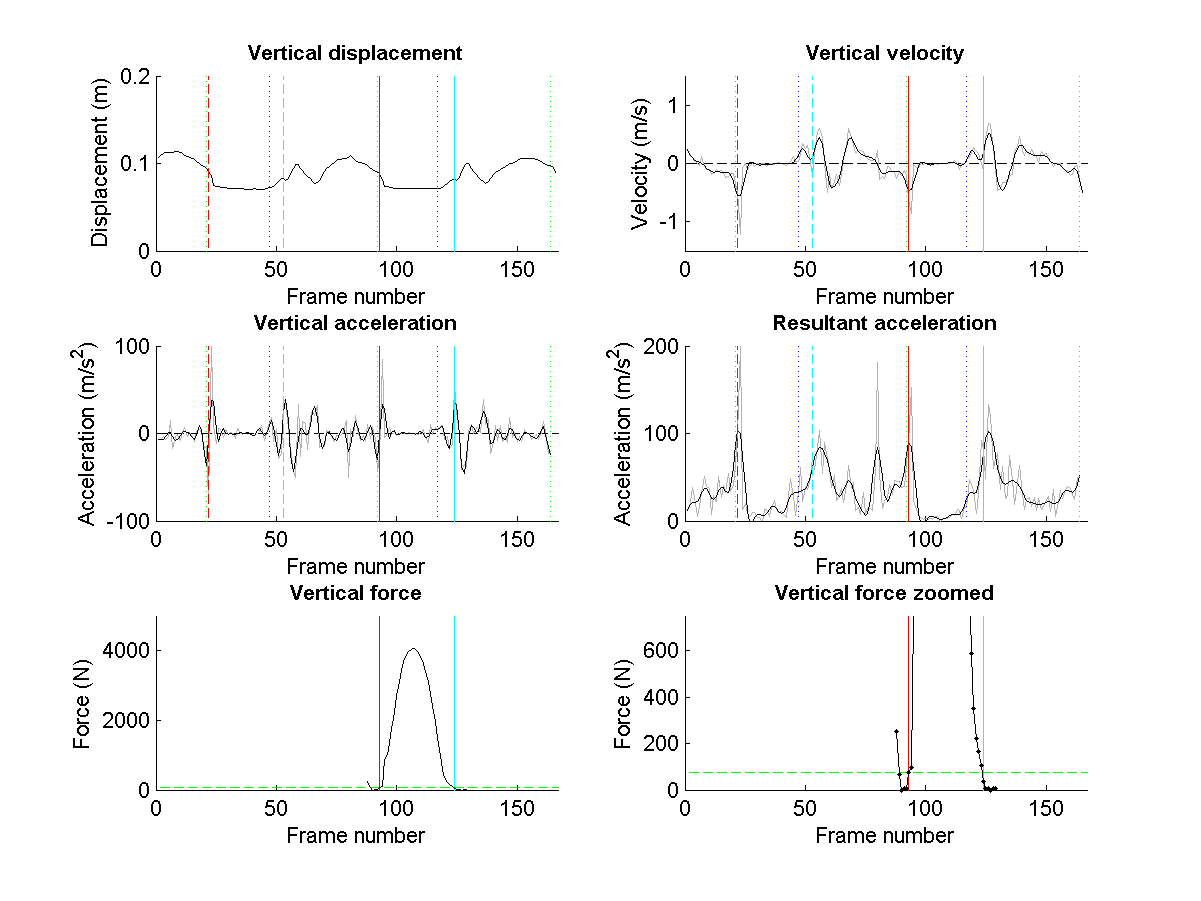

Supplement: Supplemental Information 3 [file peerj-03-783-s003.zip › Suppl figures/Event-based/Event_plot-LH_Horse3_circle_right_trot_07.png]

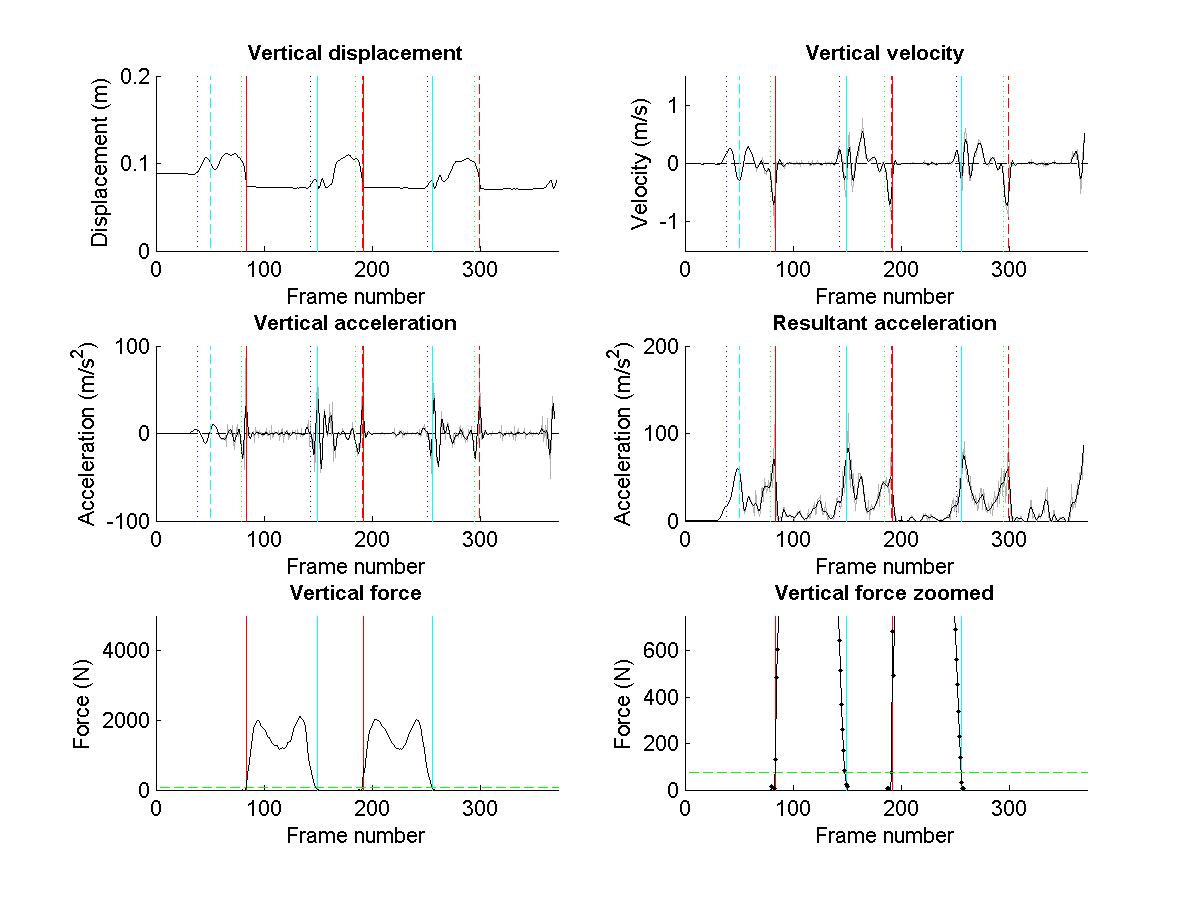

Supplement: Supplemental Information 3 [file peerj-03-783-s003.zip › Suppl figures/Event-based/Event_plot-LH_Horse3_circle_right_walk_08.png]

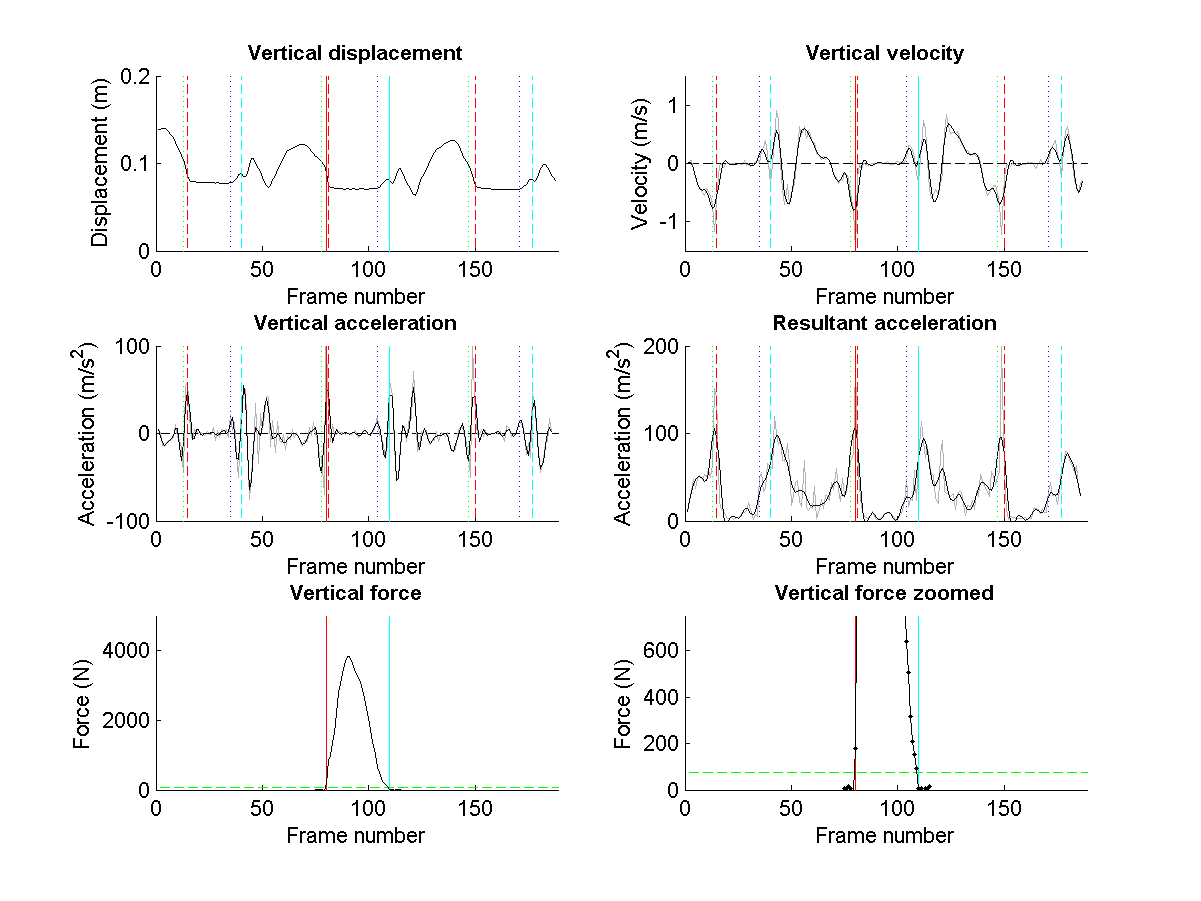

Supplement: Supplemental Information 3 [file peerj-03-783-s003.zip › Suppl figures/Event-based/Event_plot-LH_Horse3_trot_1.png]

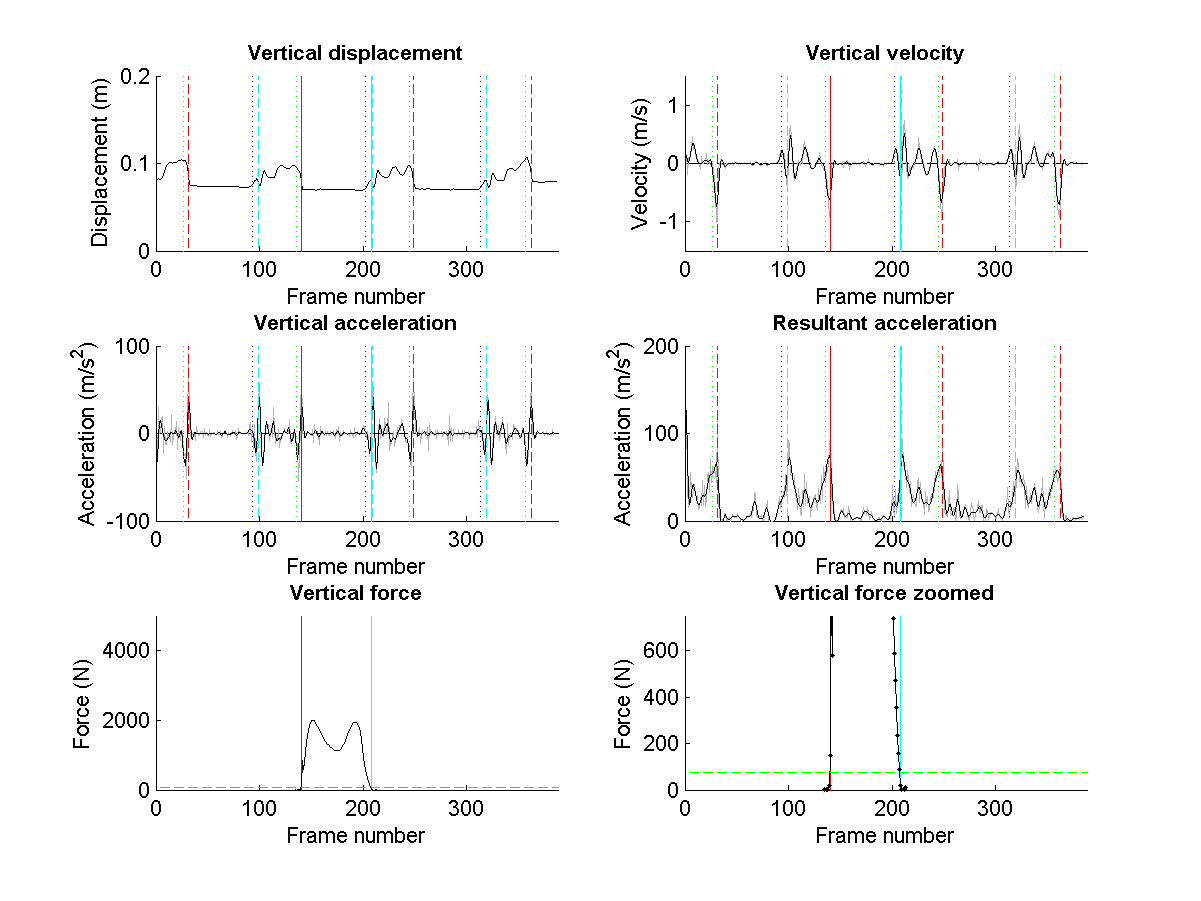

Supplement: Supplemental Information 3 [file peerj-03-783-s003.zip › Suppl figures/Event-based/Event_plot-LH_Horse3_walk_03.png]

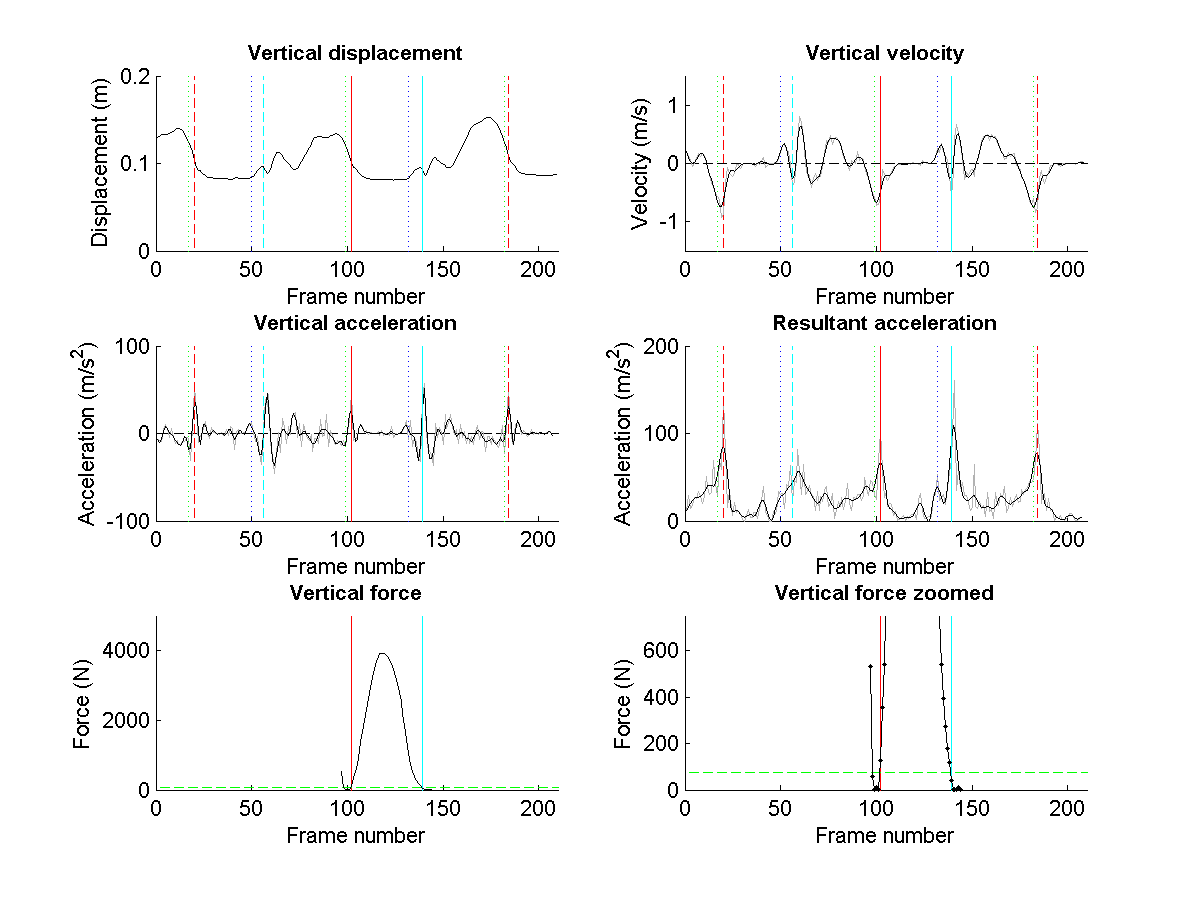

Supplement: Supplemental Information 3 [file peerj-03-783-s003.zip › Suppl figures/Event-based/Event_plot-LH_Horse5_circle_left_trot_03.png]

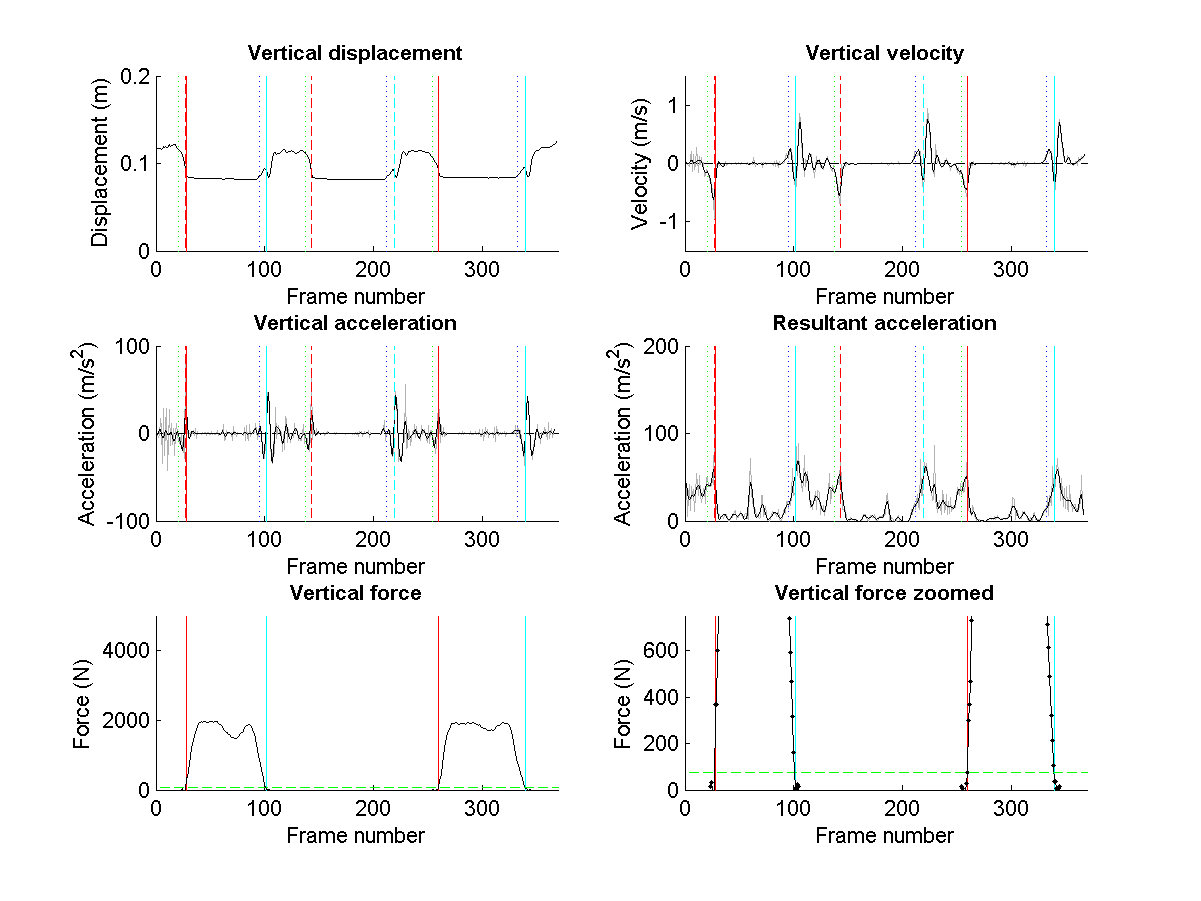

Supplement: Supplemental Information 3 [file peerj-03-783-s003.zip › Suppl figures/Event-based/Event_plot-LH_Horse5_circle_left_walk_03.png]

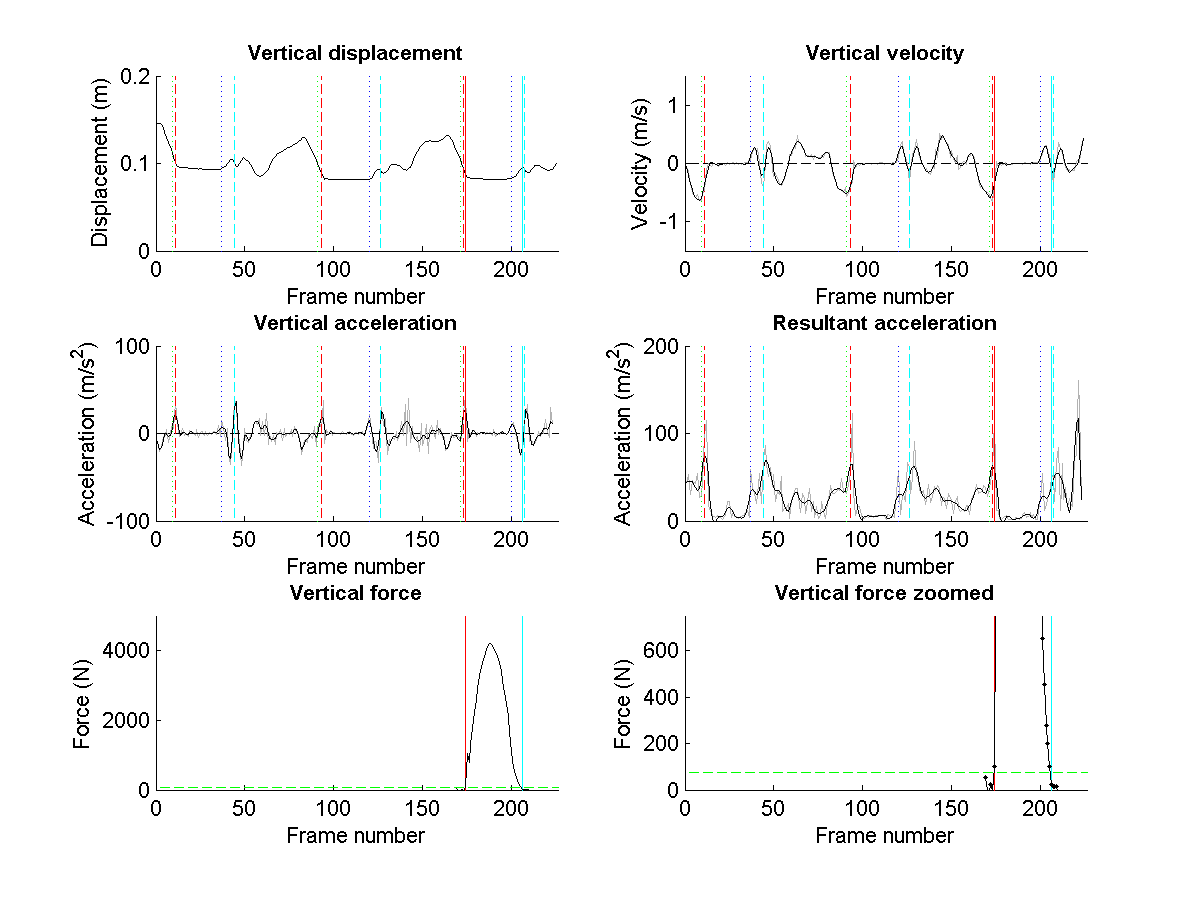

Supplement: Supplemental Information 3 [file peerj-03-783-s003.zip › Suppl figures/Event-based/Event_plot-LH_Horse5_circle_right_trot_09.png]

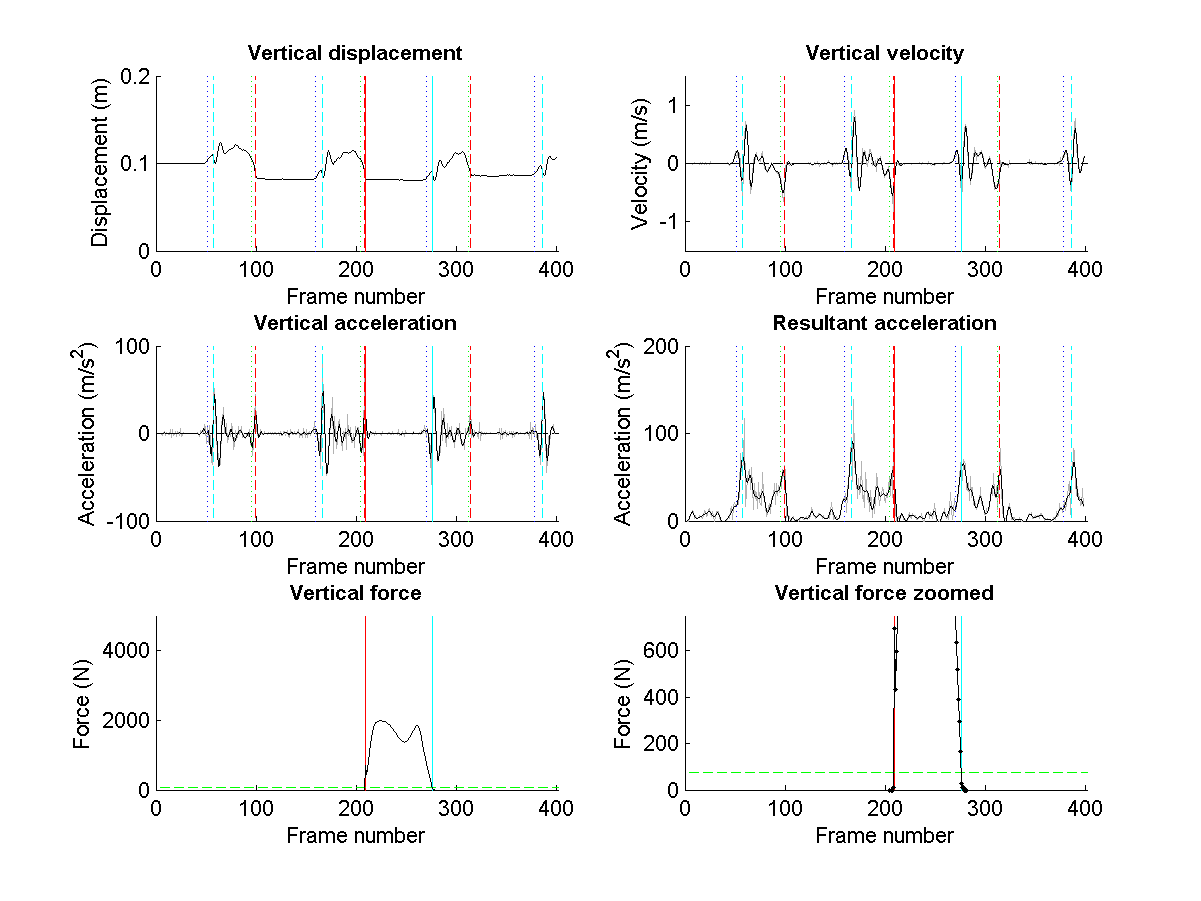

Supplement: Supplemental Information 3 [file peerj-03-783-s003.zip › Suppl figures/Event-based/Event_plot-LH_Horse5_circle_right_walk_02.png]

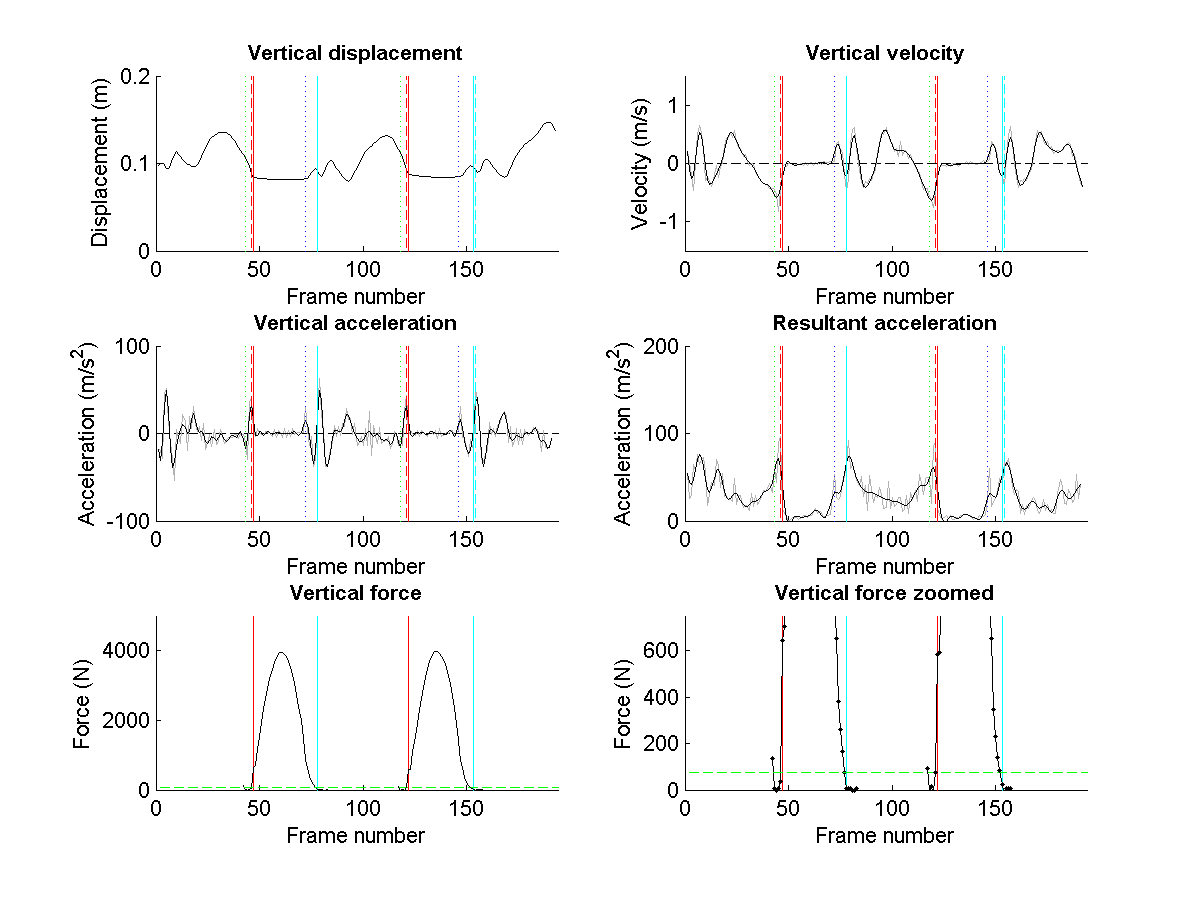

Supplement: Supplemental Information 3 [file peerj-03-783-s003.zip › Suppl figures/Event-based/Event_plot-LH_Horse5_trot_06.png]

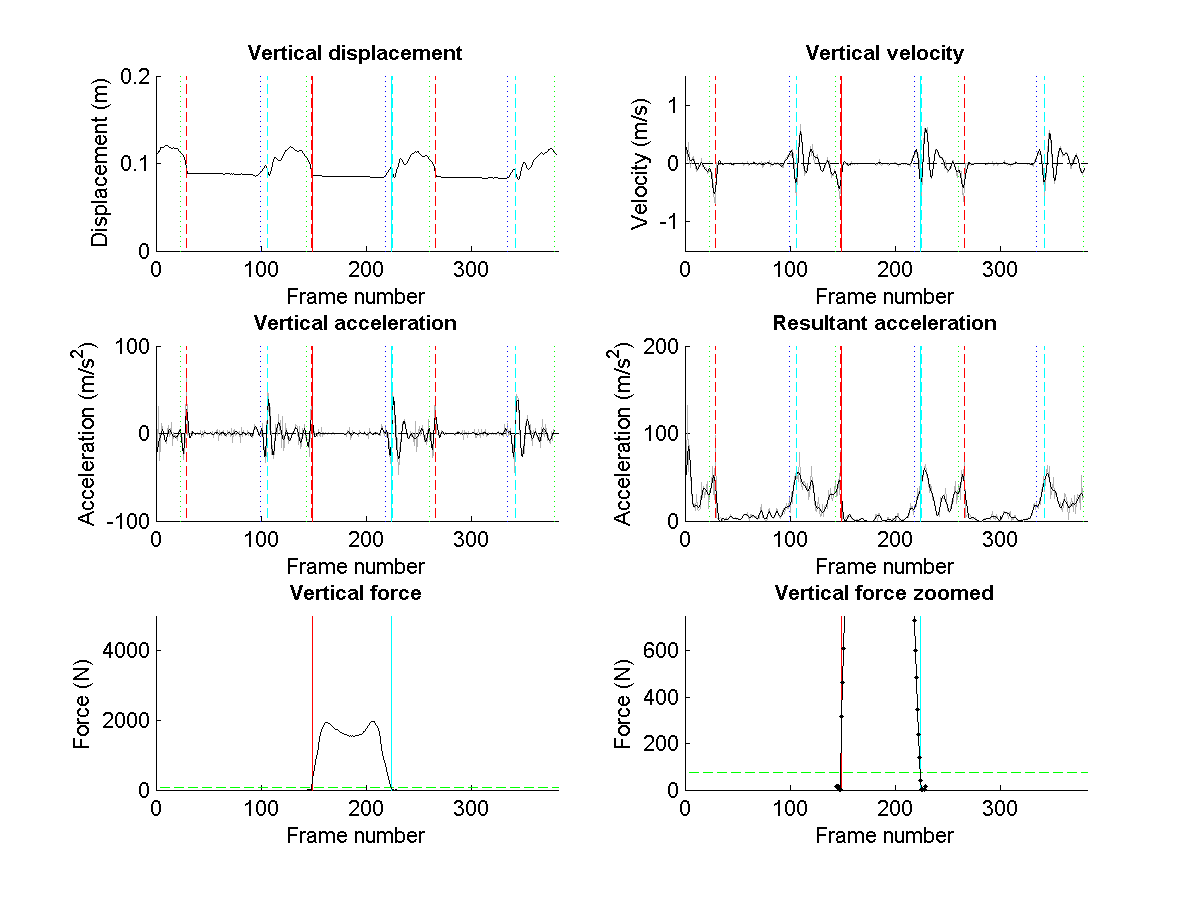

Supplement: Supplemental Information 3 [file peerj-03-783-s003.zip › Suppl figures/Event-based/Event_plot-LH_Horse5_walk_09.png]

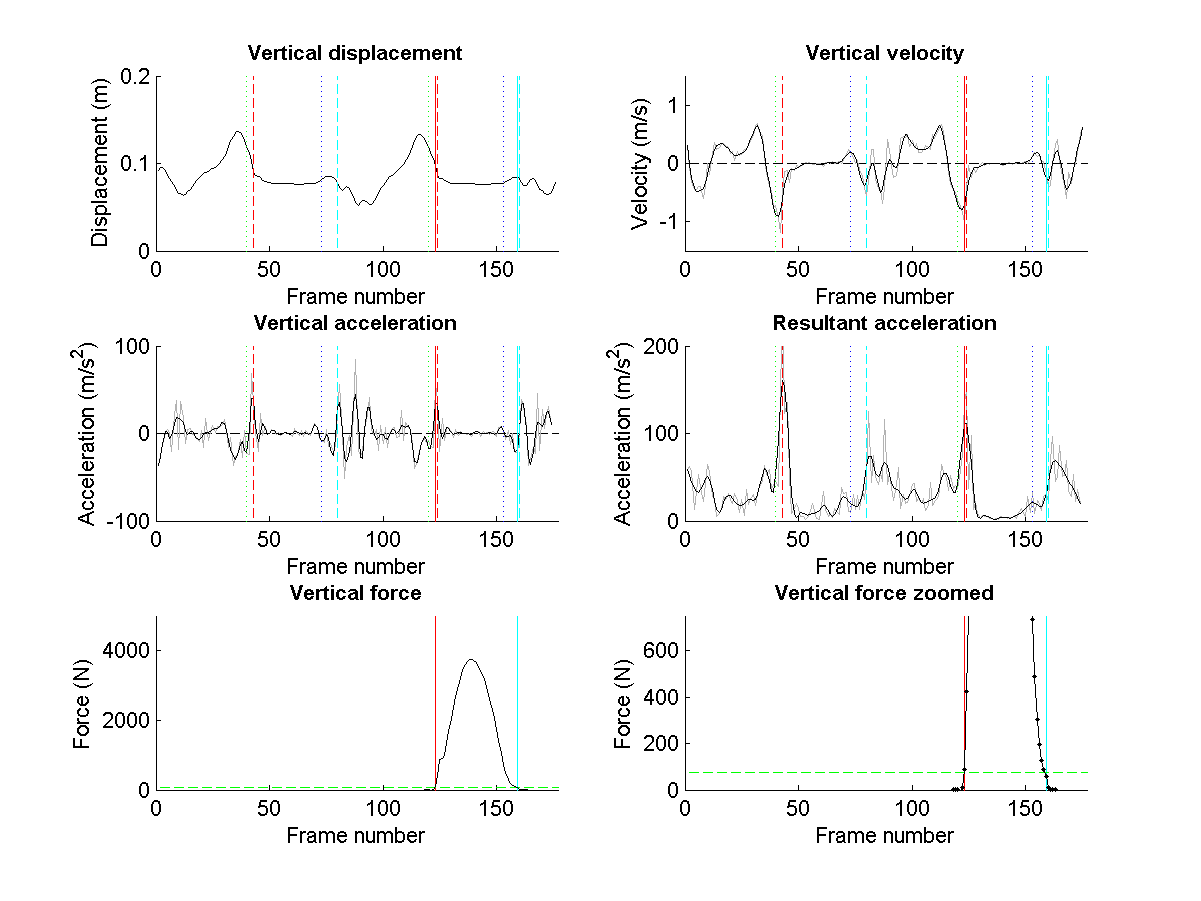

Supplement: Supplemental Information 3 [file peerj-03-783-s003.zip › Suppl figures/Event-based/Event_plot-LH_Horse8_circle_left_trot_06.png]

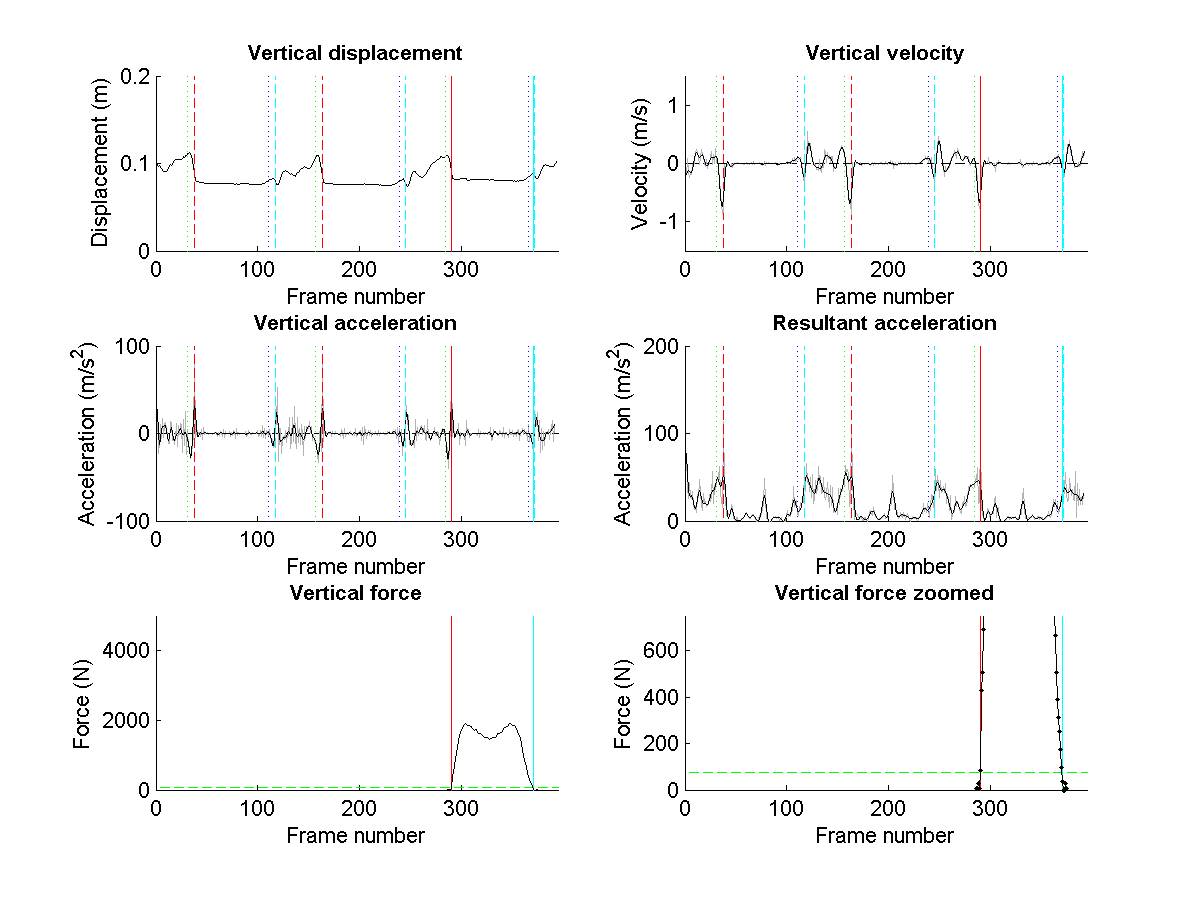

Supplement: Supplemental Information 3 [file peerj-03-783-s003.zip › Suppl figures/Event-based/Event_plot-LH_Horse8_circle_left_walk_01.png]

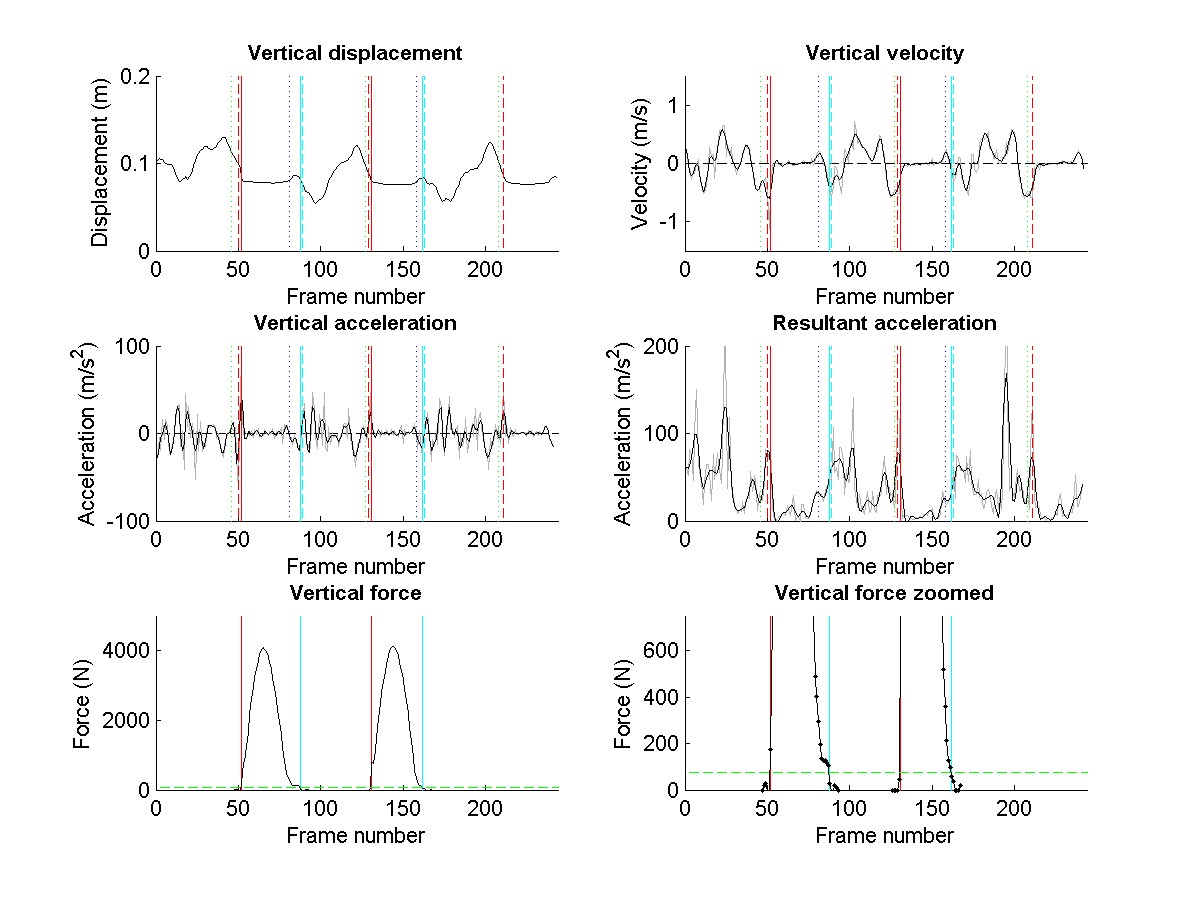

Supplement: Supplemental Information 3 [file peerj-03-783-s003.zip › Suppl figures/Event-based/Event_plot-LH_Horse8_circle_right_trot01.png]

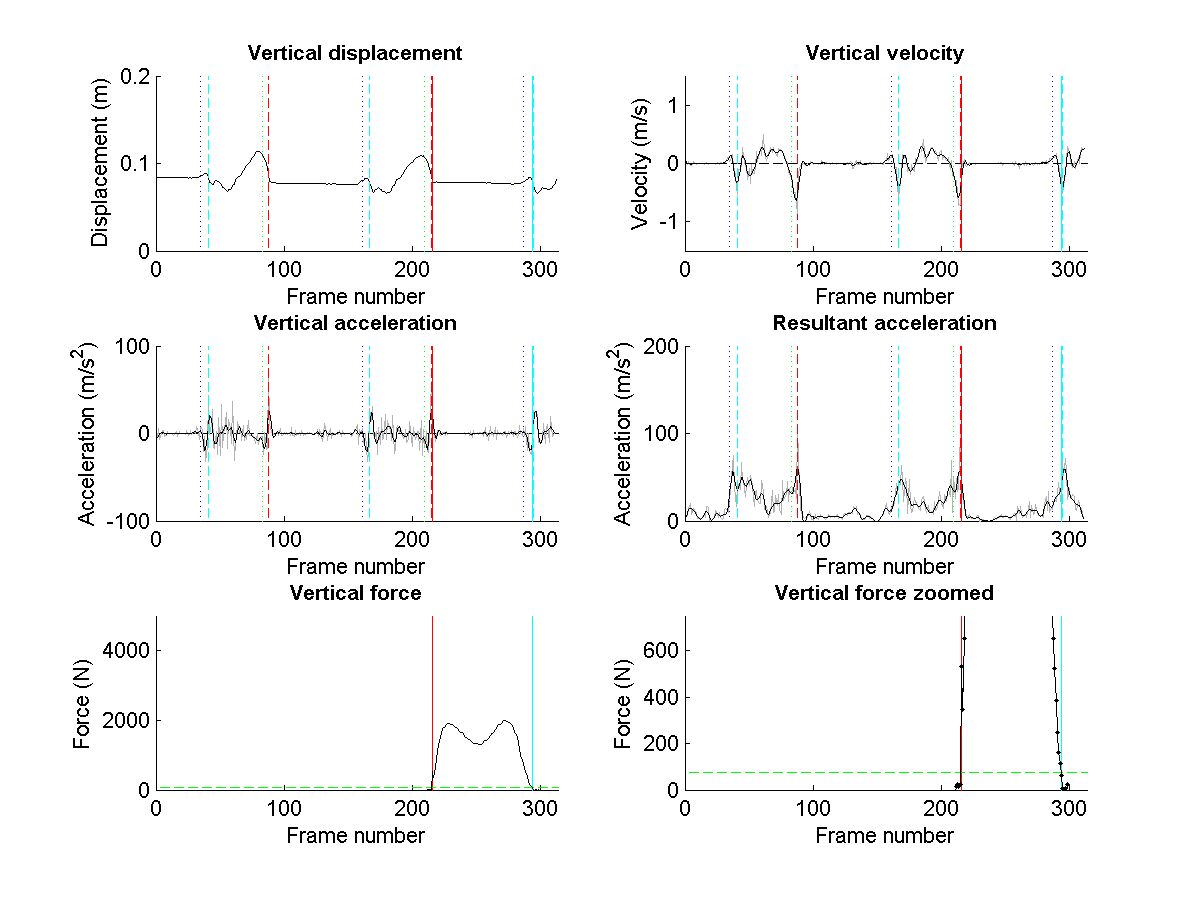

Supplement: Supplemental Information 3 [file peerj-03-783-s003.zip › Suppl figures/Event-based/Event_plot-LH_Horse8_circle_right_walk_05.png]

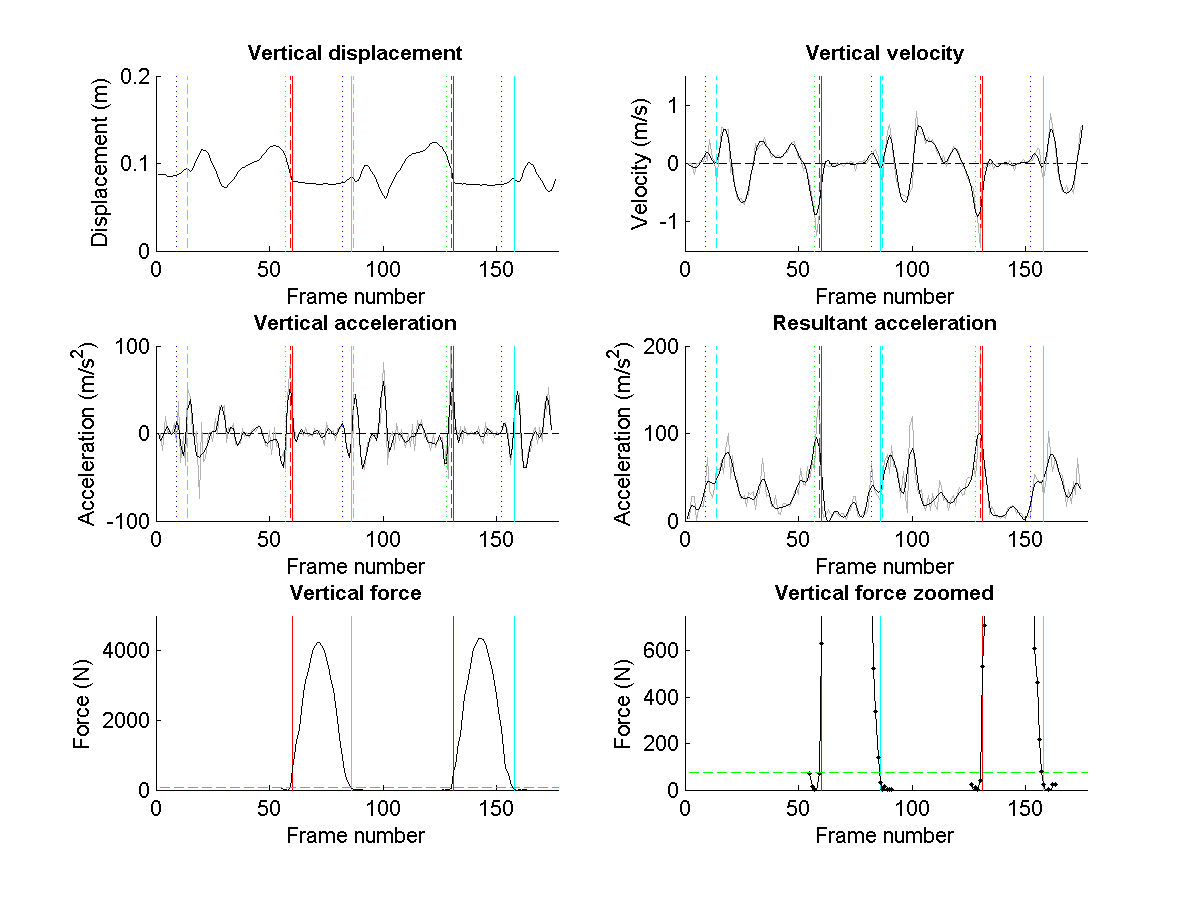

Supplement: Supplemental Information 3 [file peerj-03-783-s003.zip › Suppl figures/Event-based/Event_plot-LH_Horse8_trot_10.png]

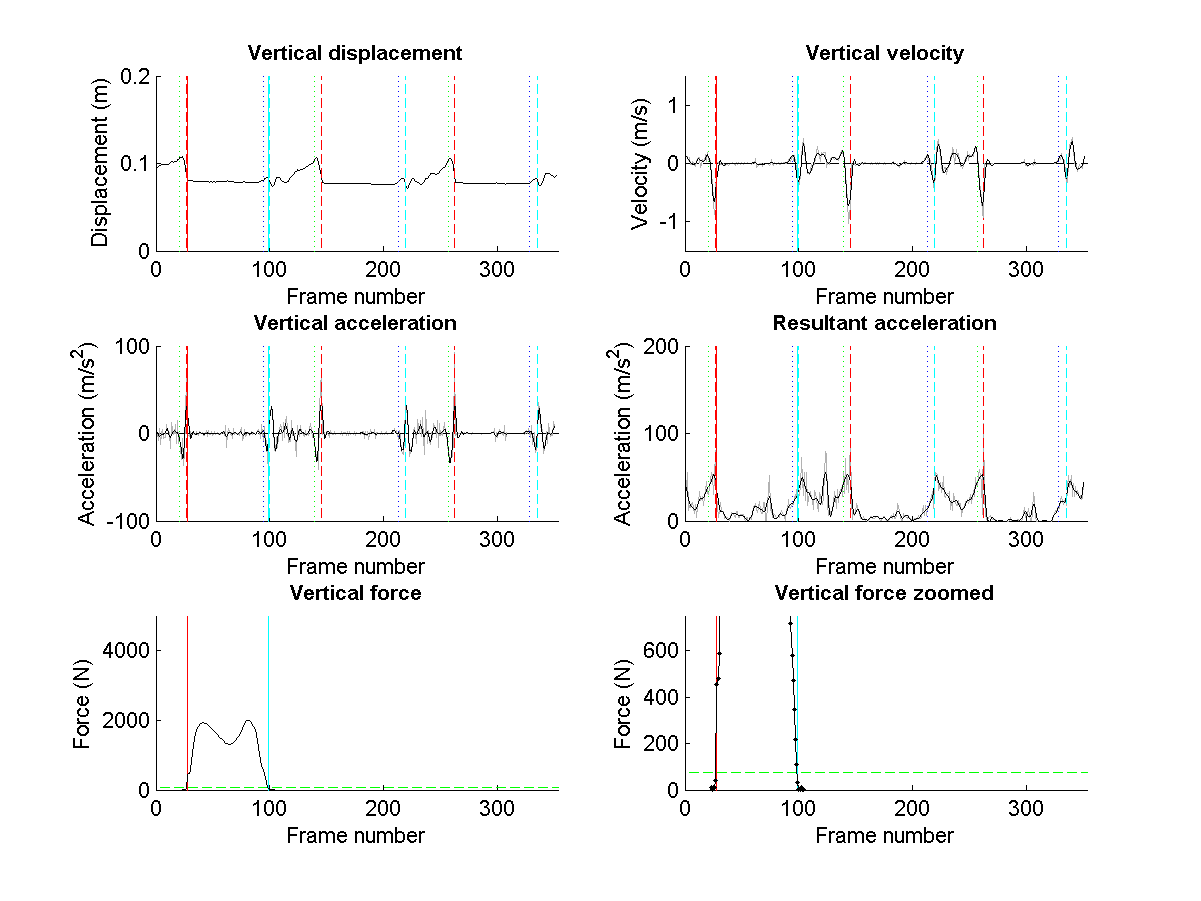

Supplement: Supplemental Information 3 [file peerj-03-783-s003.zip › Suppl figures/Event-based/Event_plot-LH_Horse8_walk_01.png]

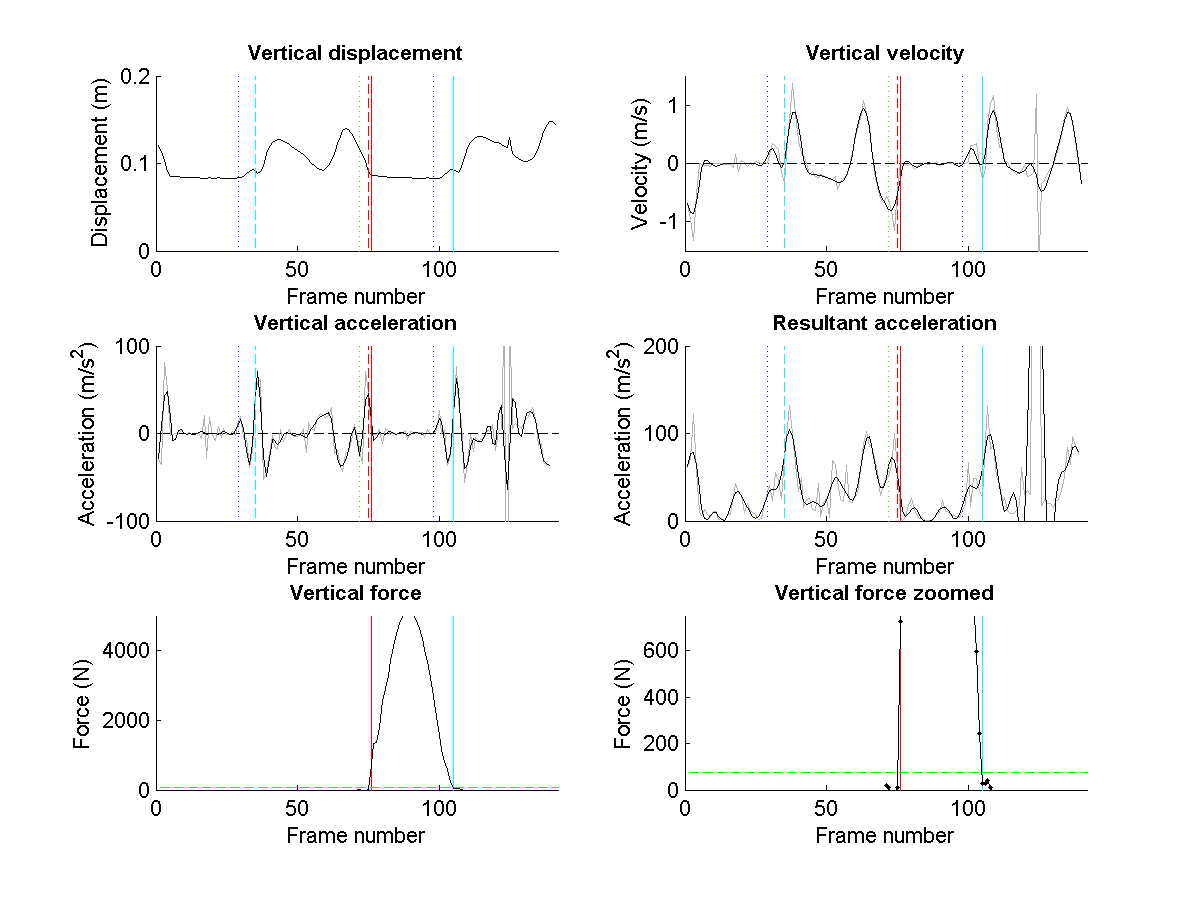

Supplement: Supplemental Information 3 [file peerj-03-783-s003.zip › Suppl figures/Event-based/Event_plot-RF_Horse11_trot_04.png]

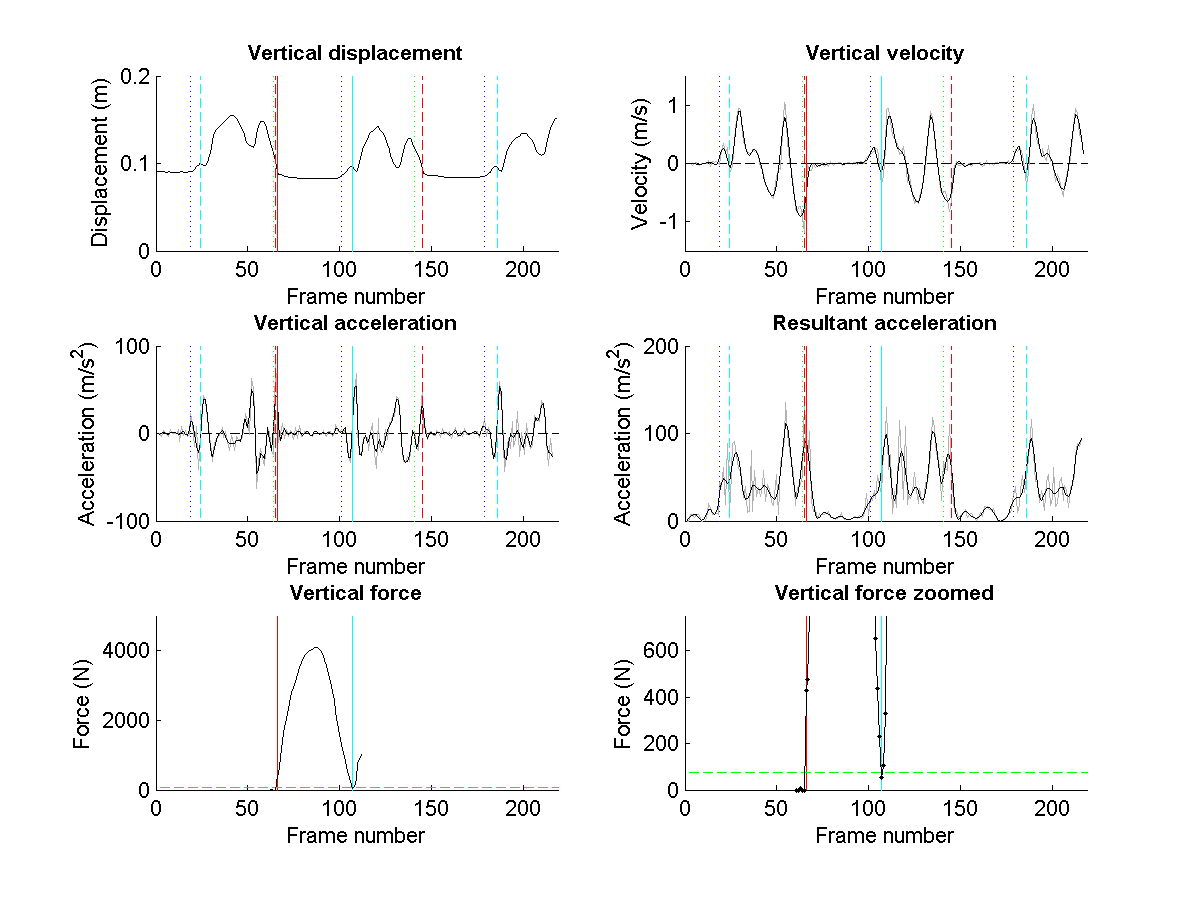

Supplement: Supplemental Information 3 [file peerj-03-783-s003.zip › Suppl figures/Event-based/Event_plot-RF_Horse11_trot_circle_left_05.png]

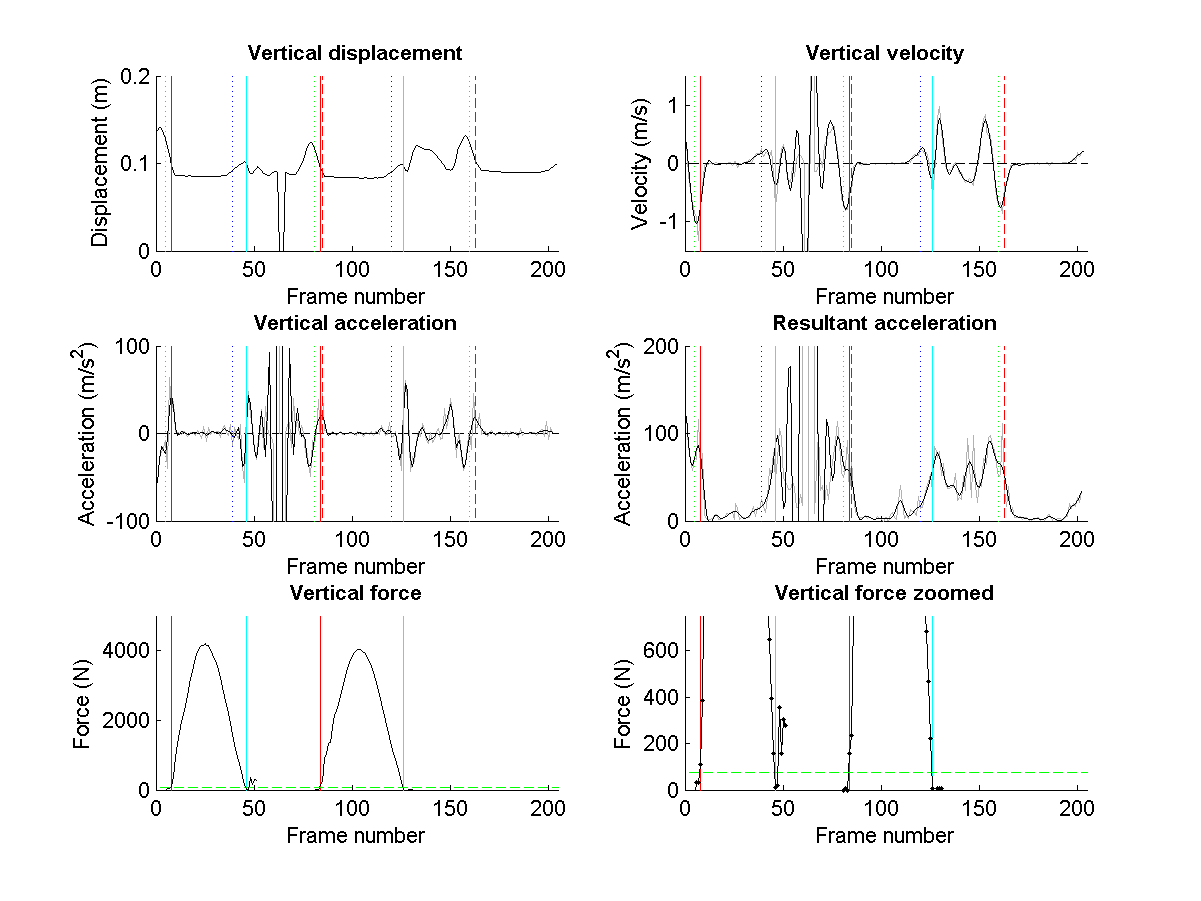

Supplement: Supplemental Information 3 [file peerj-03-783-s003.zip › Suppl figures/Event-based/Event_plot-RF_Horse11_trot_circle_right_07.png]

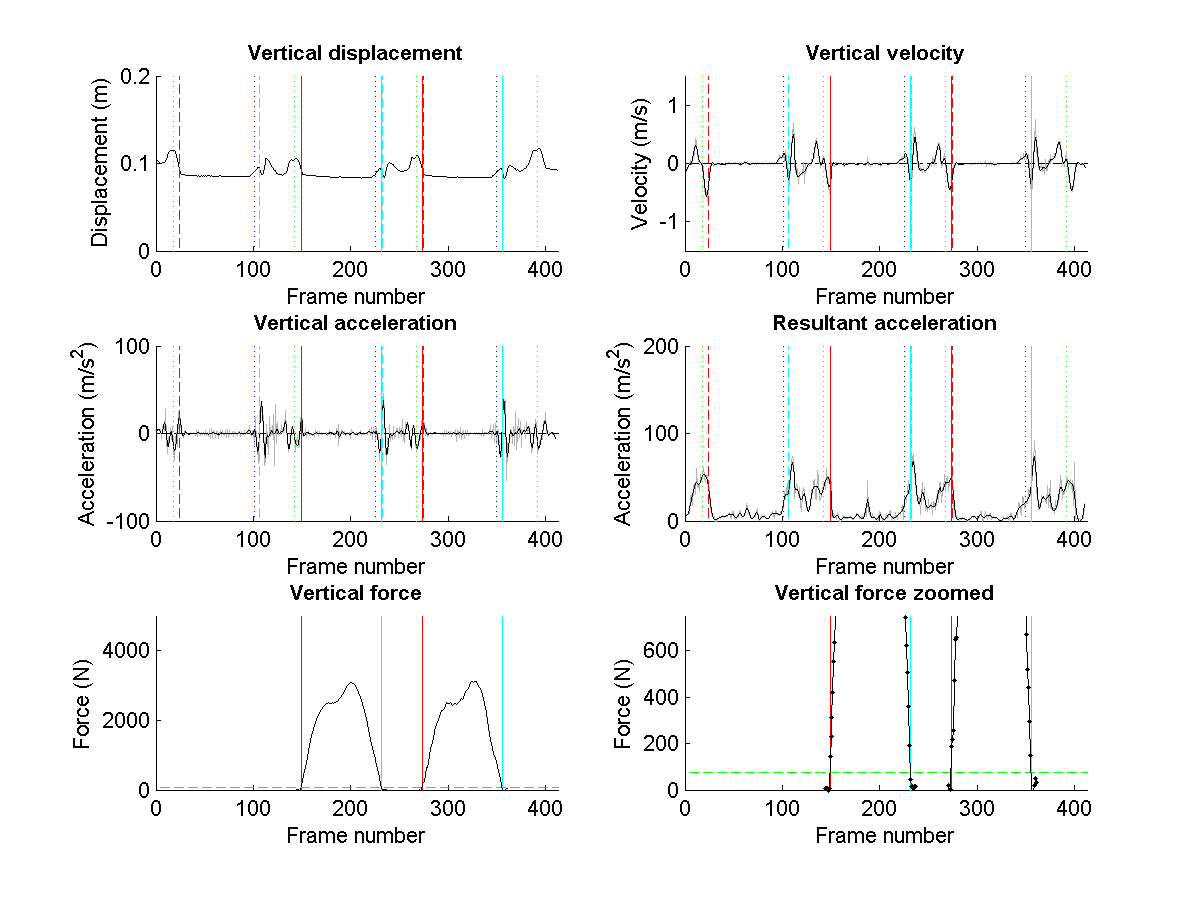

Supplement: Supplemental Information 3 [file peerj-03-783-s003.zip › Suppl figures/Event-based/Event_plot-RF_Horse11_walk_03.png]

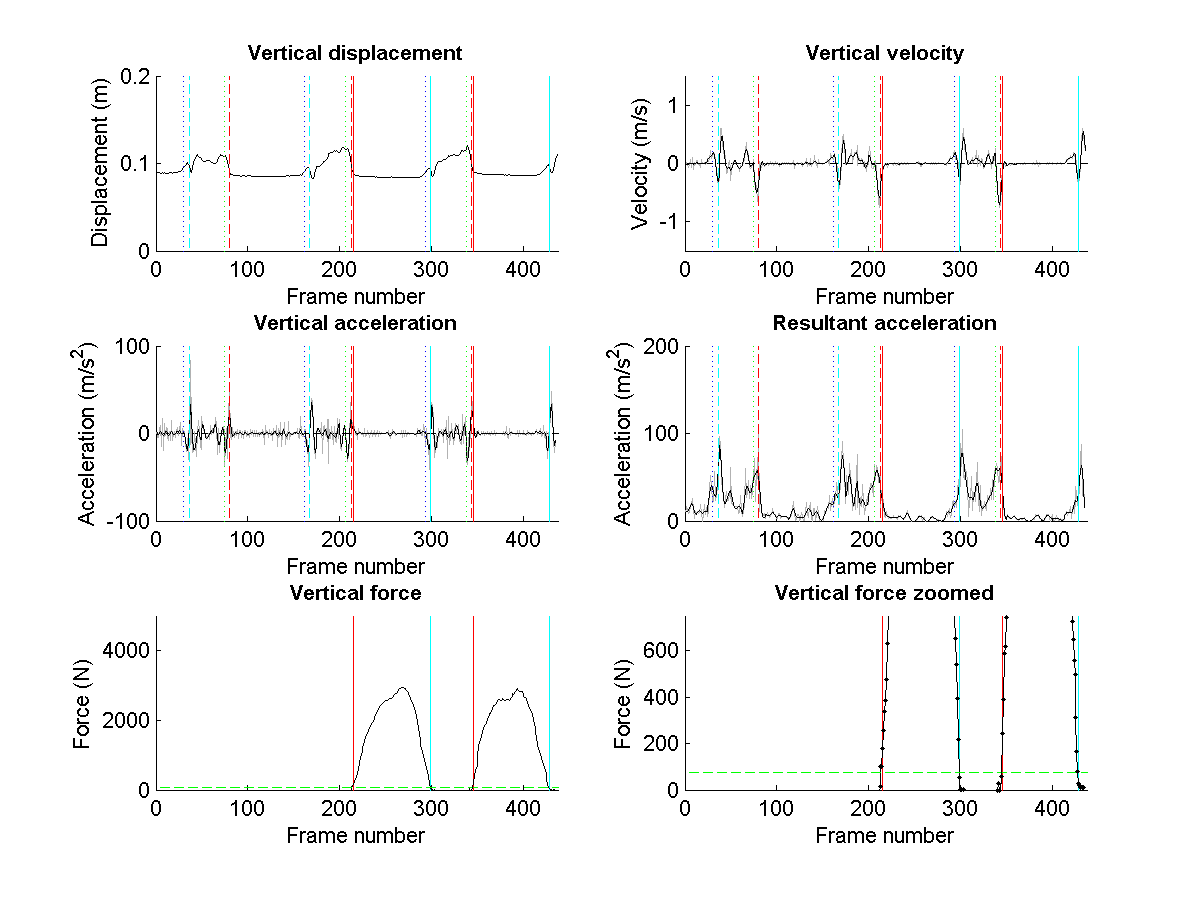

Supplement: Supplemental Information 3 [file peerj-03-783-s003.zip › Suppl figures/Event-based/Event_plot-RF_Horse11_walk_circle_left_06.png]

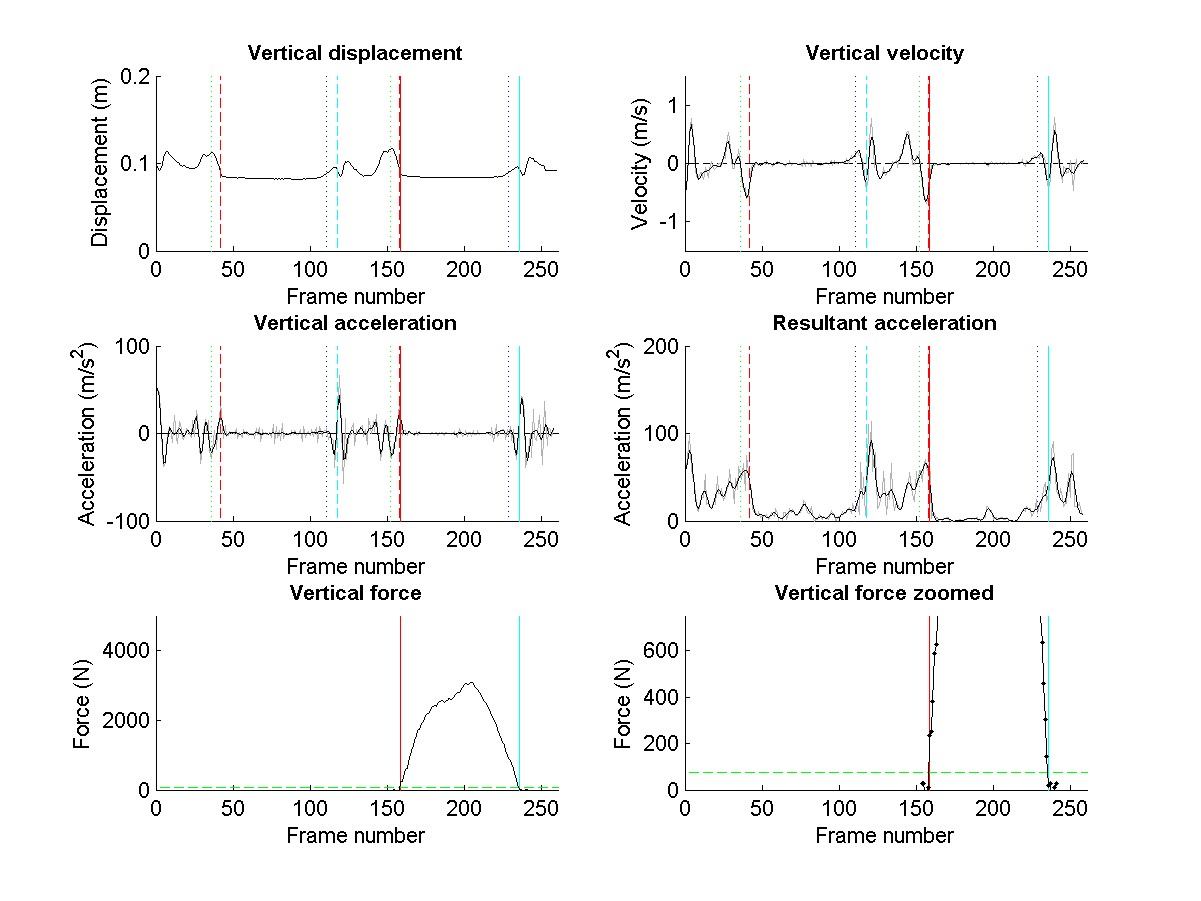

Supplement: Supplemental Information 3 [file peerj-03-783-s003.zip › Suppl figures/Event-based/Event_plot-RF_Horse11_walk_circle_right_06.png]

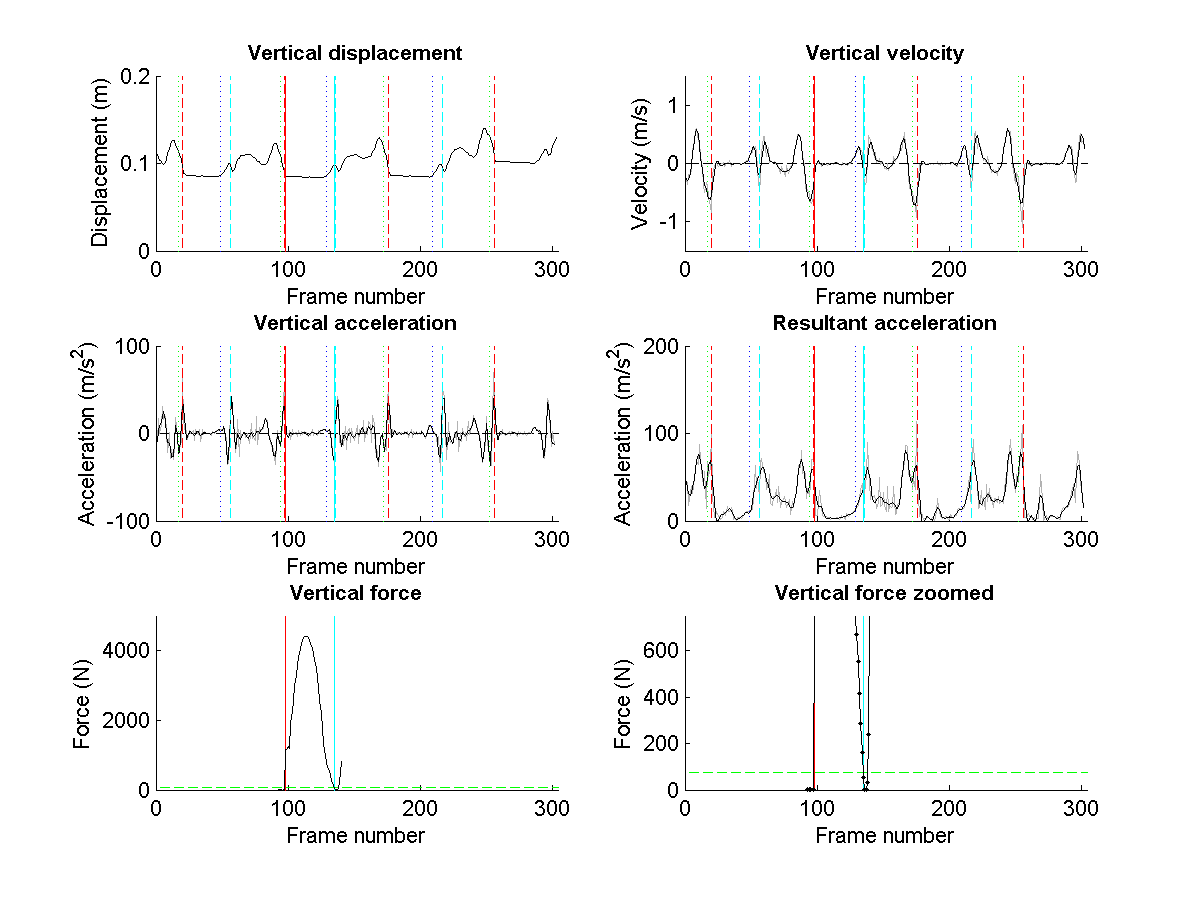

Supplement: Supplemental Information 3 [file peerj-03-783-s003.zip › Suppl figures/Event-based/Event_plot-RF_Horse4_circle_left_trot_07.png]

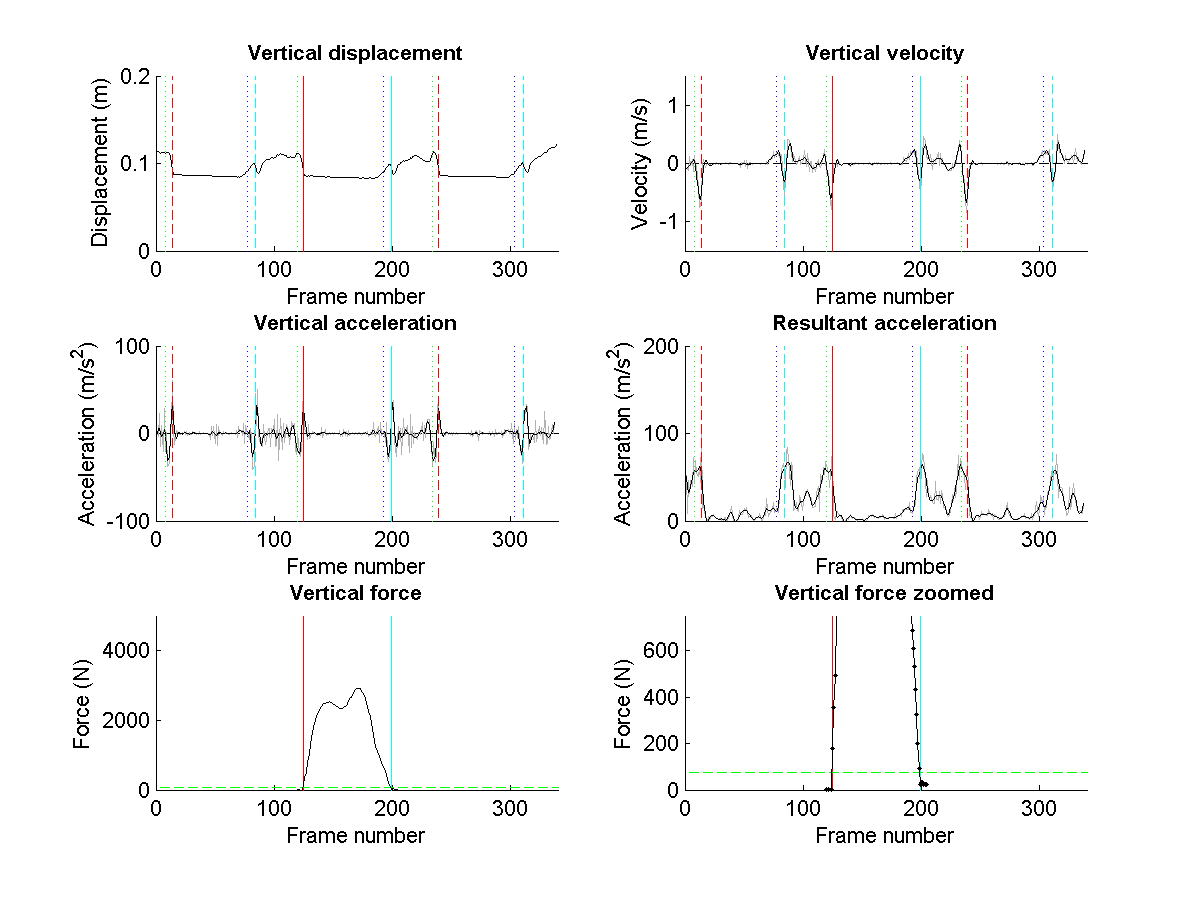

Supplement: Supplemental Information 3 [file peerj-03-783-s003.zip › Suppl figures/Event-based/Event_plot-RF_Horse4_circle_left_walk04.png]

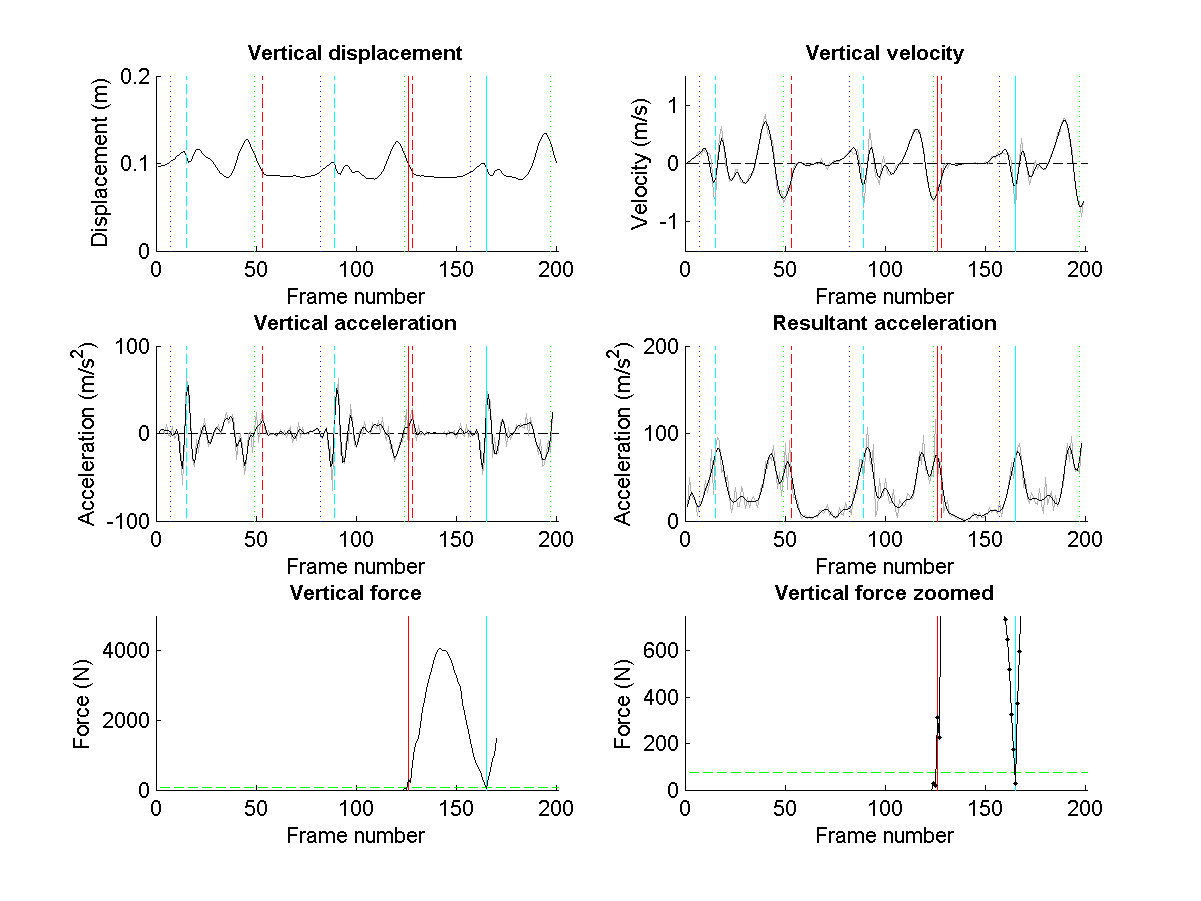

Supplement: Supplemental Information 3 [file peerj-03-783-s003.zip › Suppl figures/Event-based/Event_plot-RF_Horse4_circle_right_trot22.png]

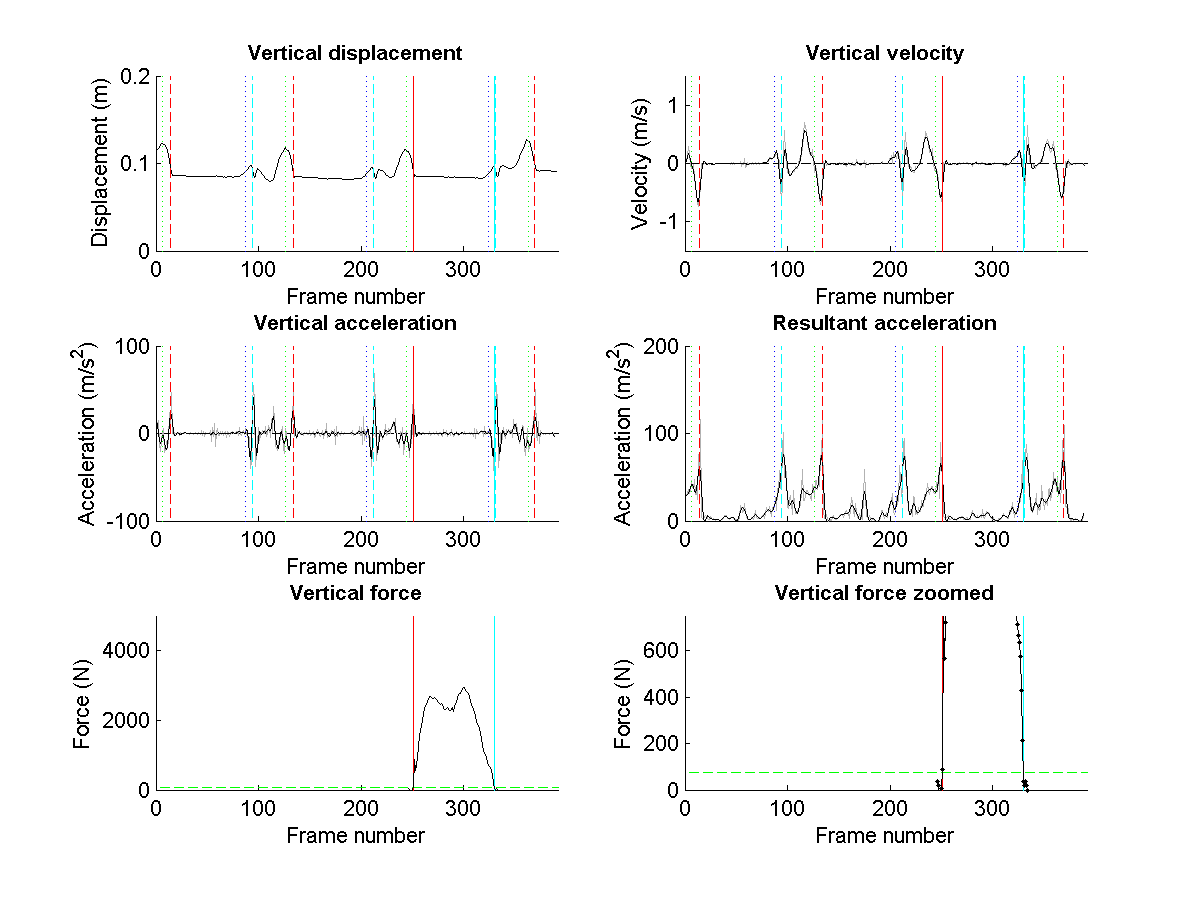

Supplement: Supplemental Information 3 [file peerj-03-783-s003.zip › Suppl figures/Event-based/Event_plot-RF_Horse4_circle_right_walk_01.png]

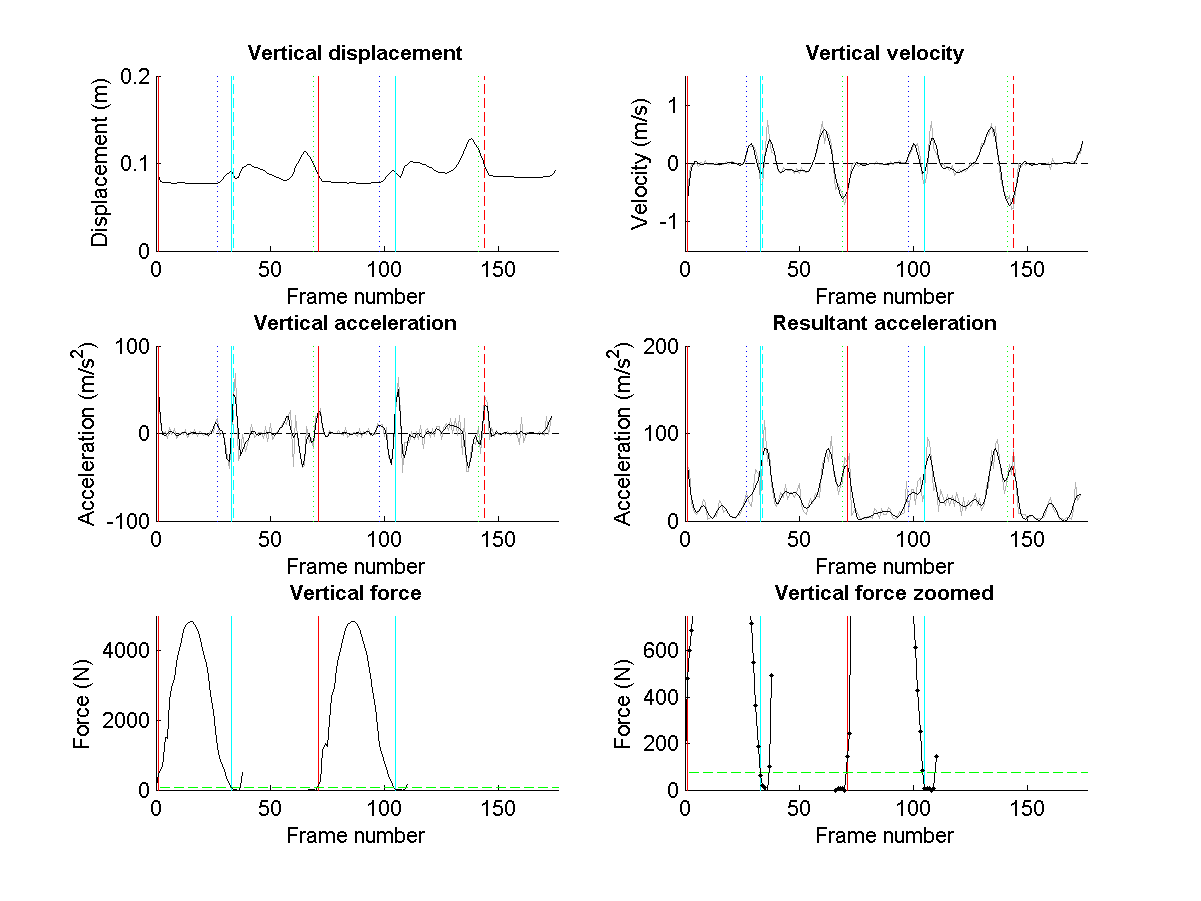

Supplement: Supplemental Information 3 [file peerj-03-783-s003.zip › Suppl figures/Event-based/Event_plot-RF_Horse4_trot_01.png]

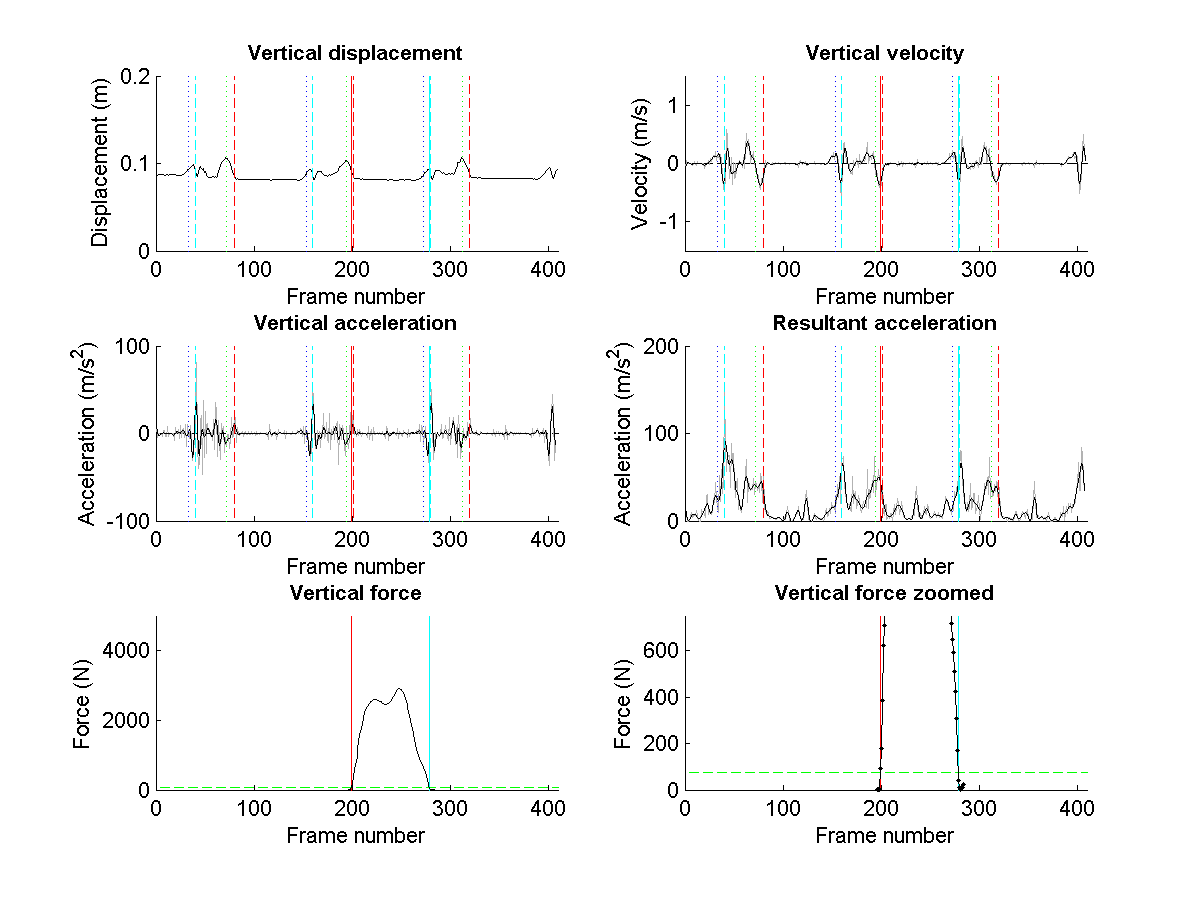

Supplement: Supplemental Information 3 [file peerj-03-783-s003.zip › Suppl figures/Event-based/Event_plot-RF_Horse4_walk_07.png]

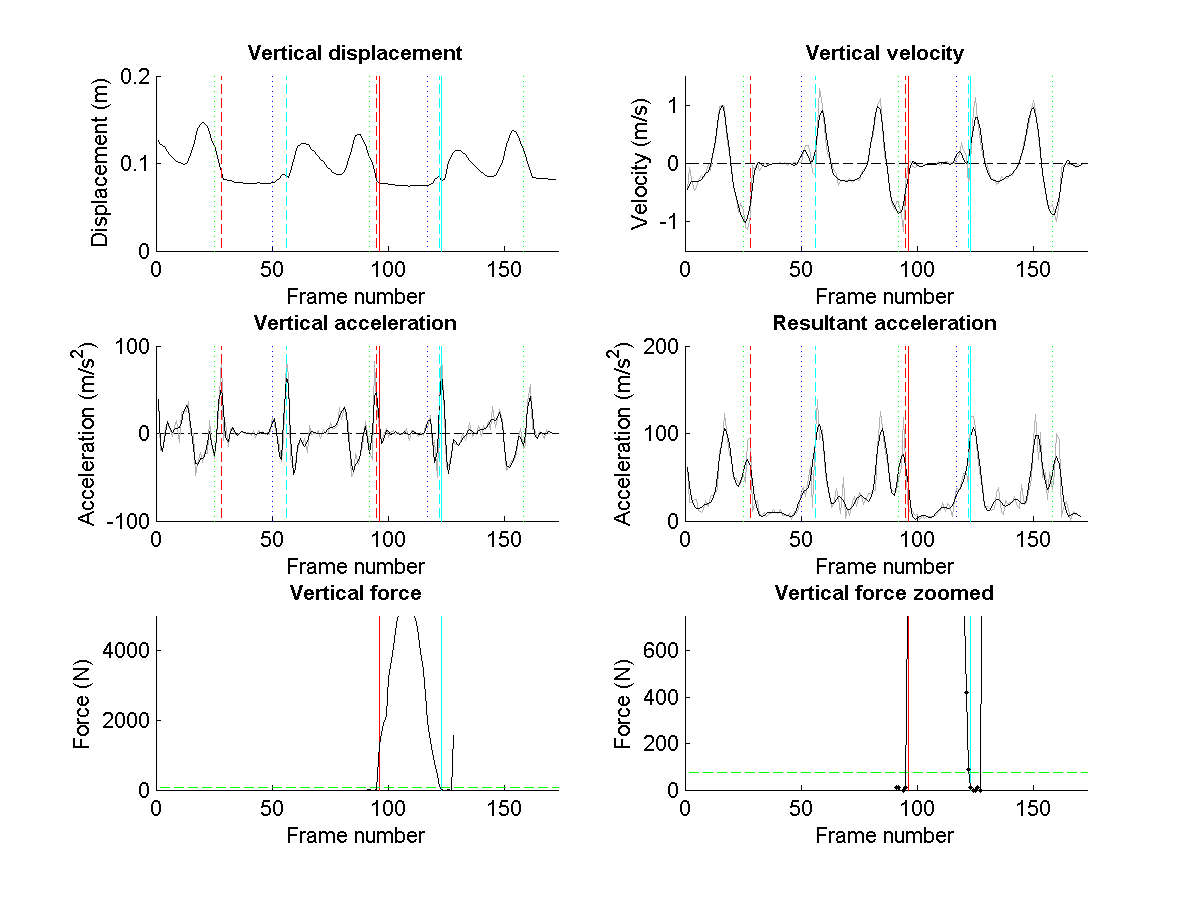

Supplement: Supplemental Information 3 [file peerj-03-783-s003.zip › Suppl figures/Event-based/Event_plot-RF_Horse6_trot_01.png]

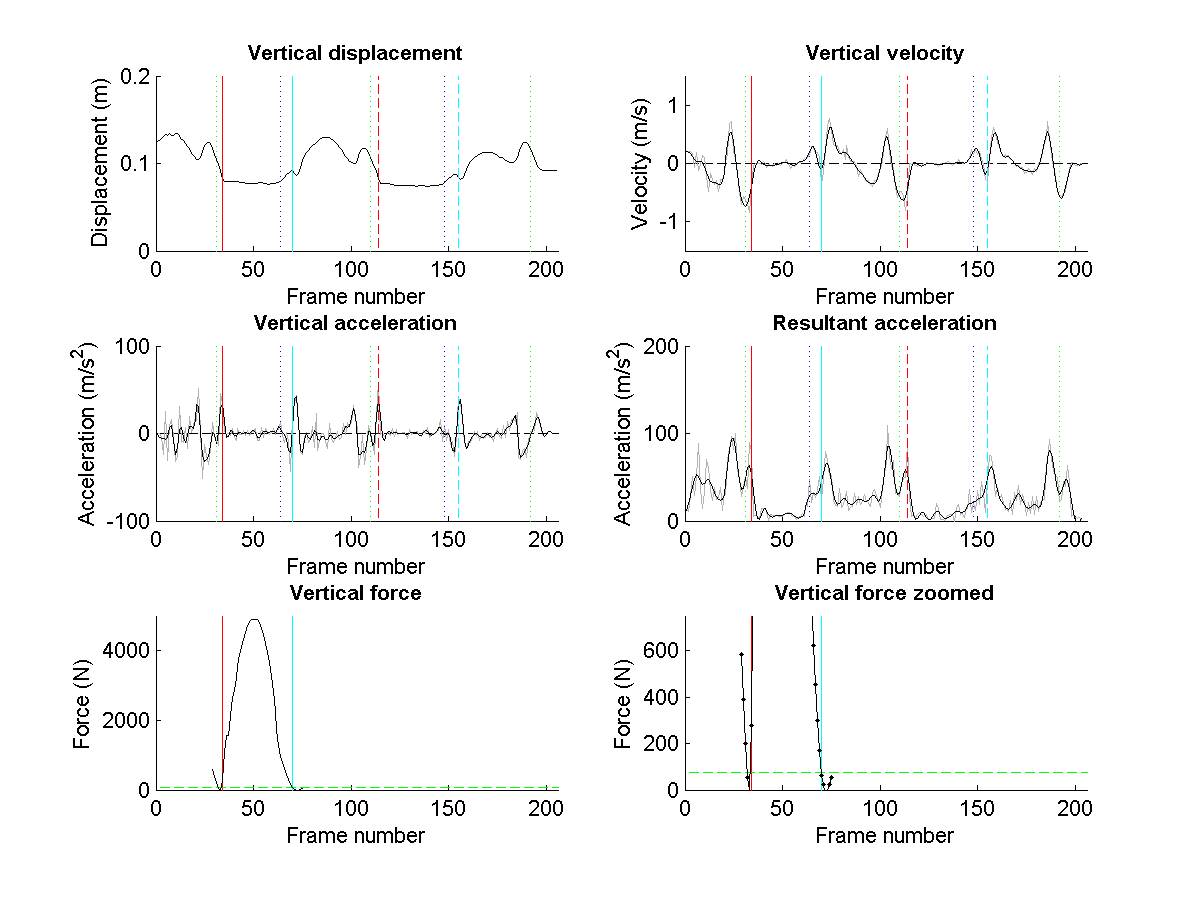

Supplement: Supplemental Information 3 [file peerj-03-783-s003.zip › Suppl figures/Event-based/Event_plot-RF_Horse6_trot_circle_left_04.png]

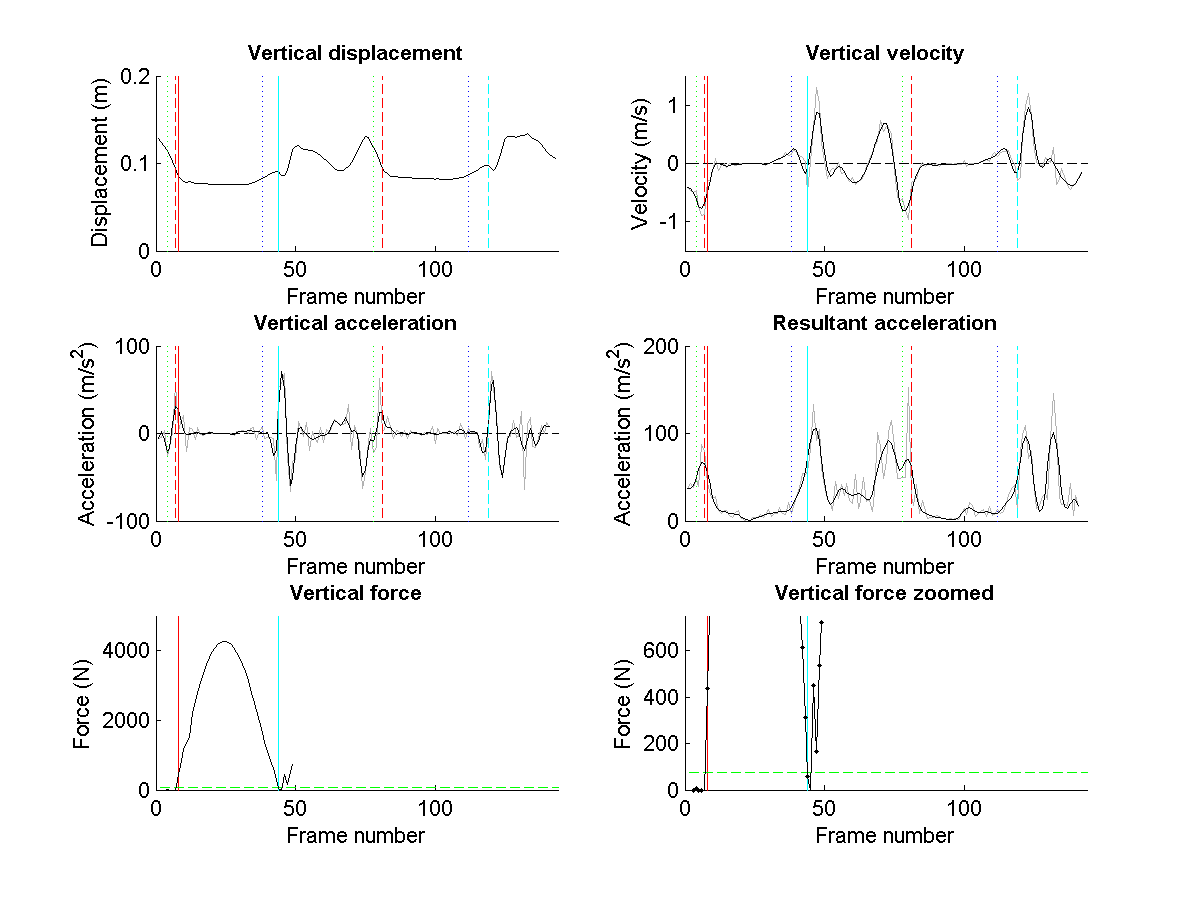

Supplement: Supplemental Information 3 [file peerj-03-783-s003.zip › Suppl figures/Event-based/Event_plot-RF_Horse6_trot_circle_right_02.png]

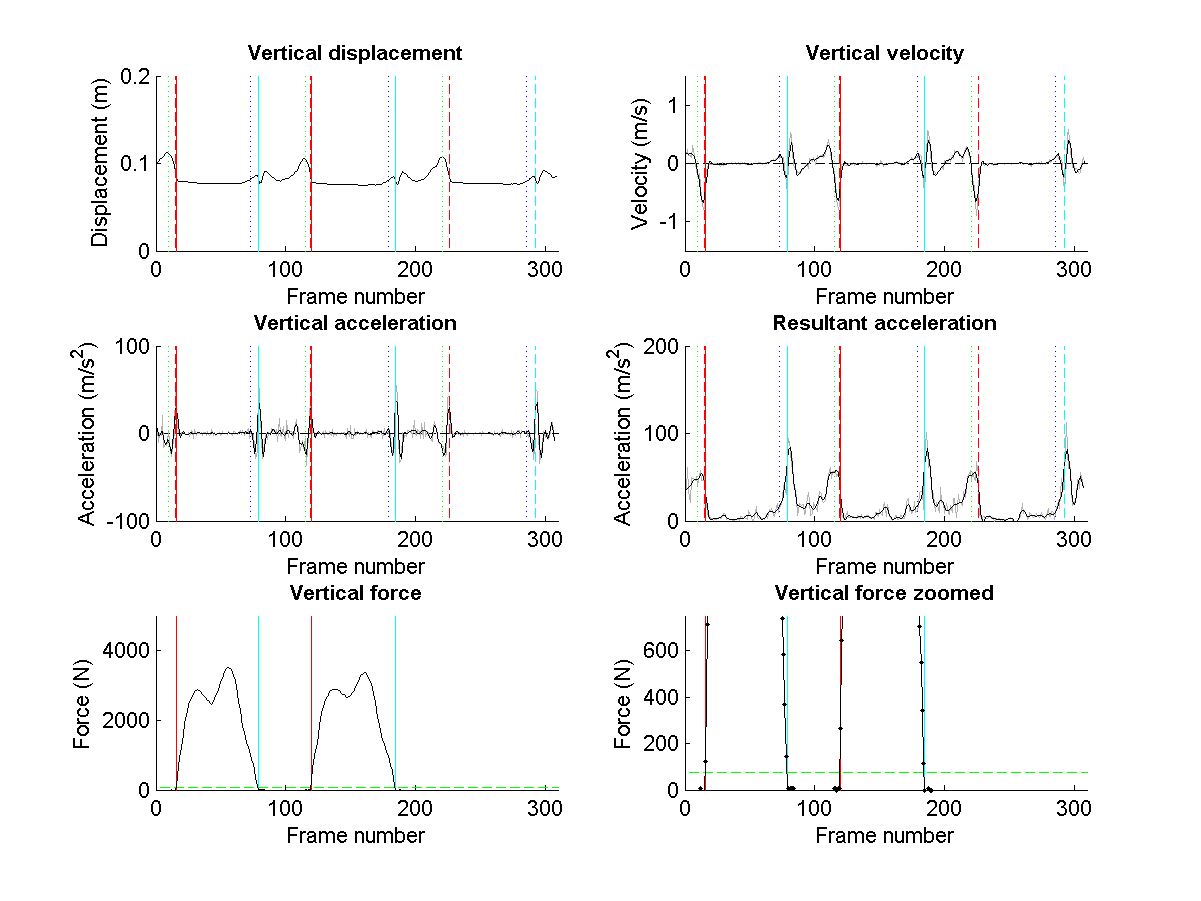

Supplement: Supplemental Information 3 [file peerj-03-783-s003.zip › Suppl figures/Event-based/Event_plot-RF_Horse6_walk_05.png]

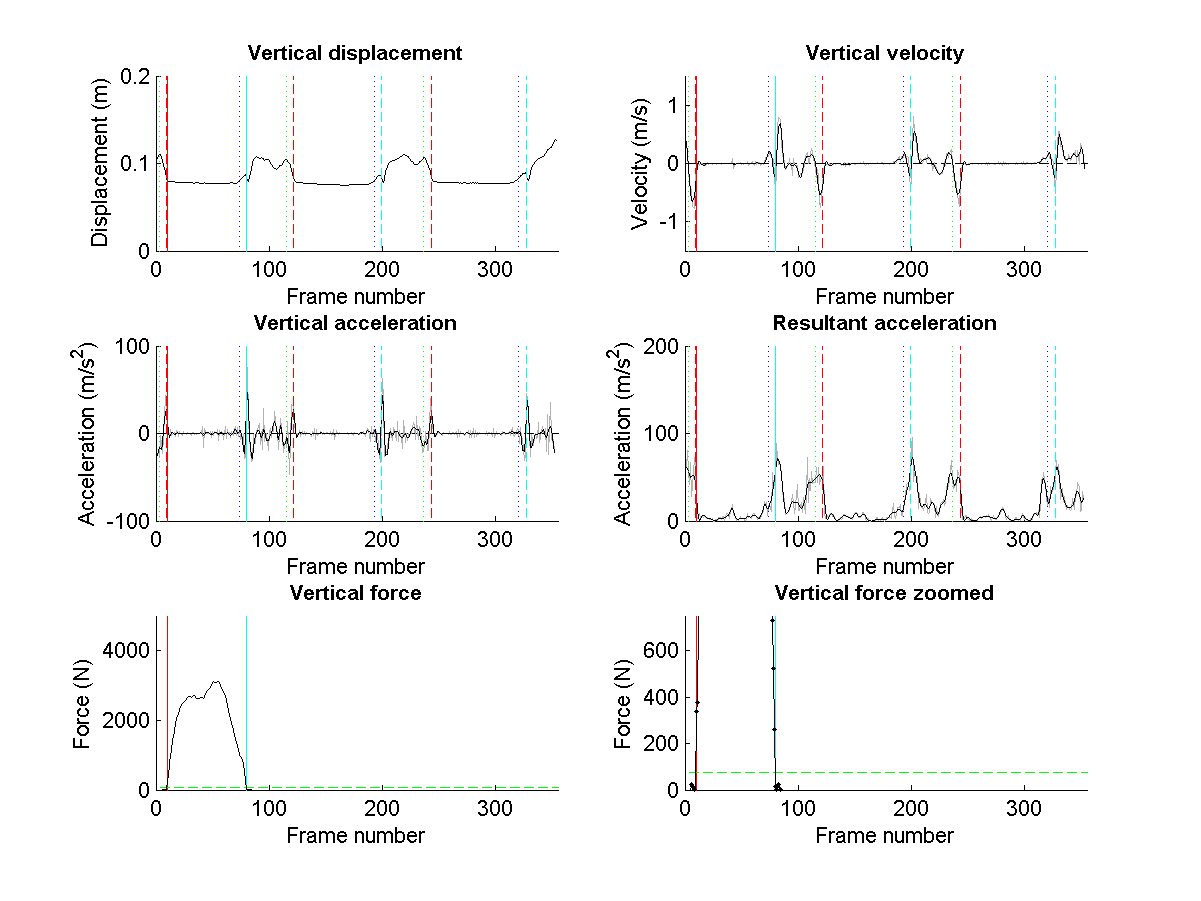

Supplement: Supplemental Information 3 [file peerj-03-783-s003.zip › Suppl figures/Event-based/Event_plot-RF_Horse6_walk_circle_left_05.png]

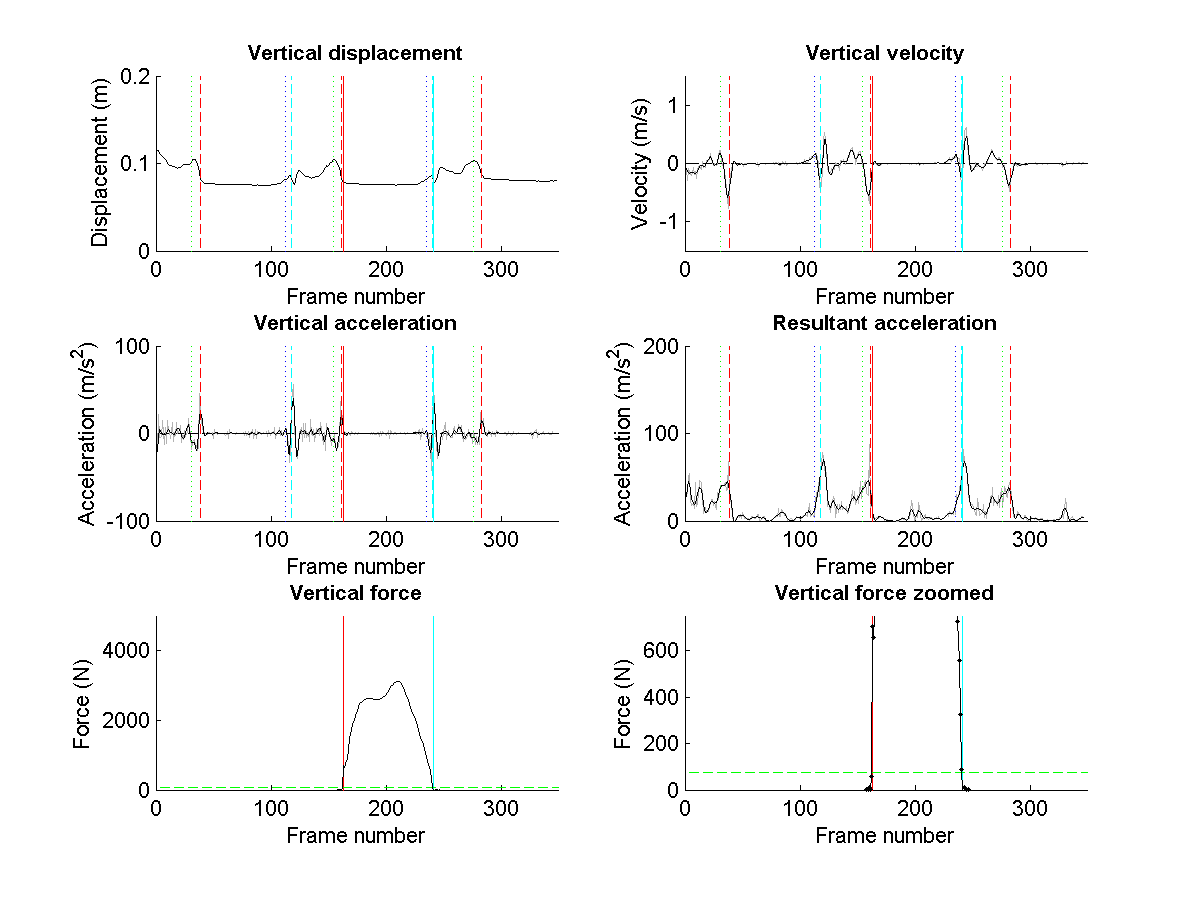

Supplement: Supplemental Information 3 [file peerj-03-783-s003.zip › Suppl figures/Event-based/Event_plot-RF_Horse6_walk_circle_right_14.png]

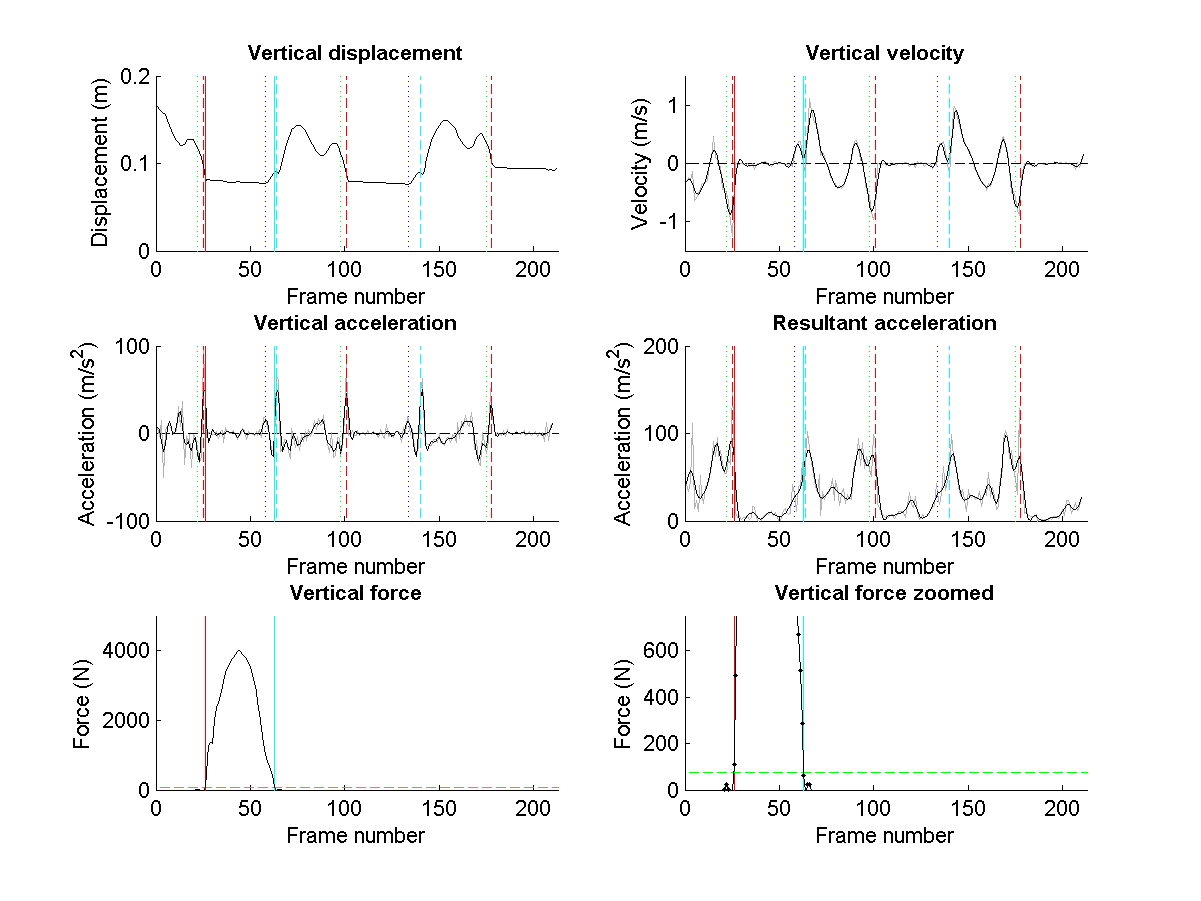

Supplement: Supplemental Information 3 [file peerj-03-783-s003.zip › Suppl figures/Event-based/Event_plot-RF_Horse9_circle_left_trot_06.png]

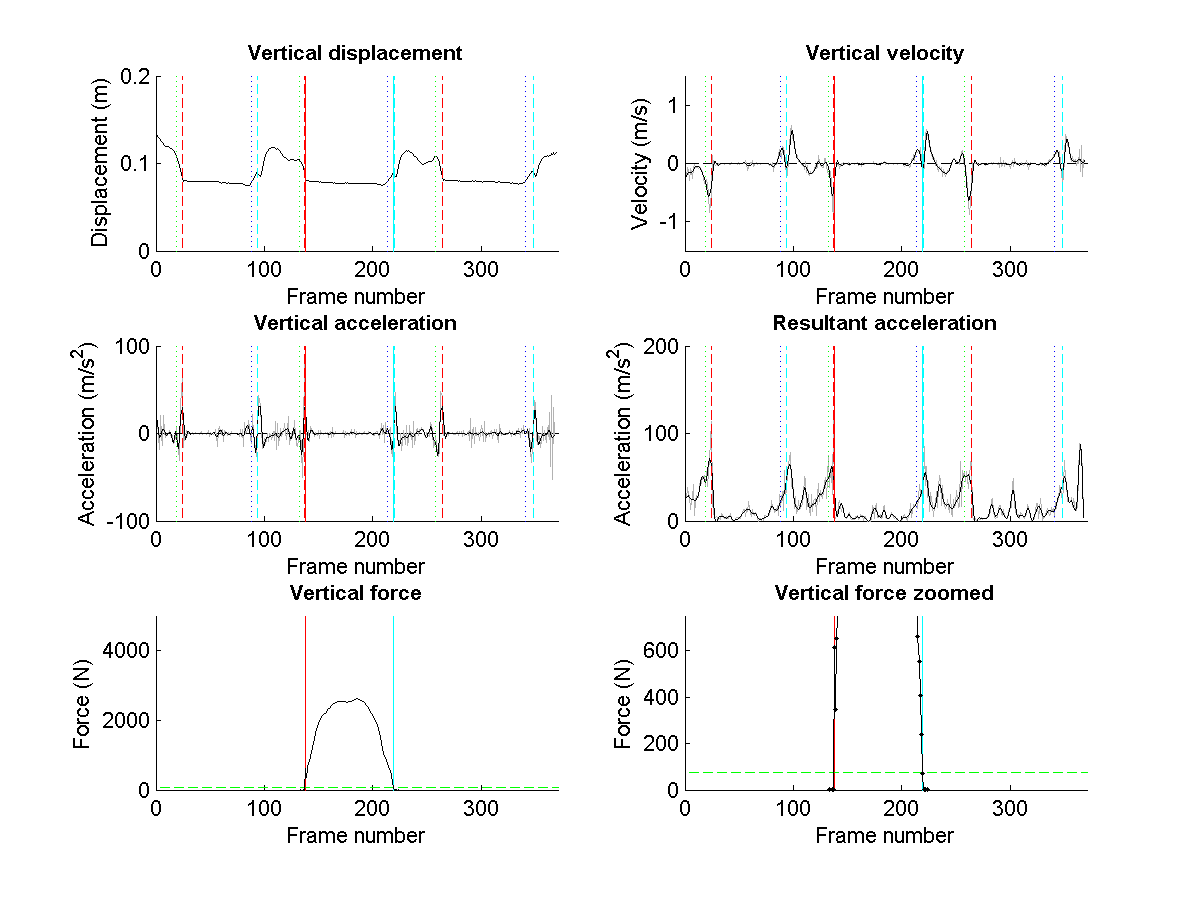

Supplement: Supplemental Information 3 [file peerj-03-783-s003.zip › Suppl figures/Event-based/Event_plot-RF_Horse9_circle_left_walk_11.png]

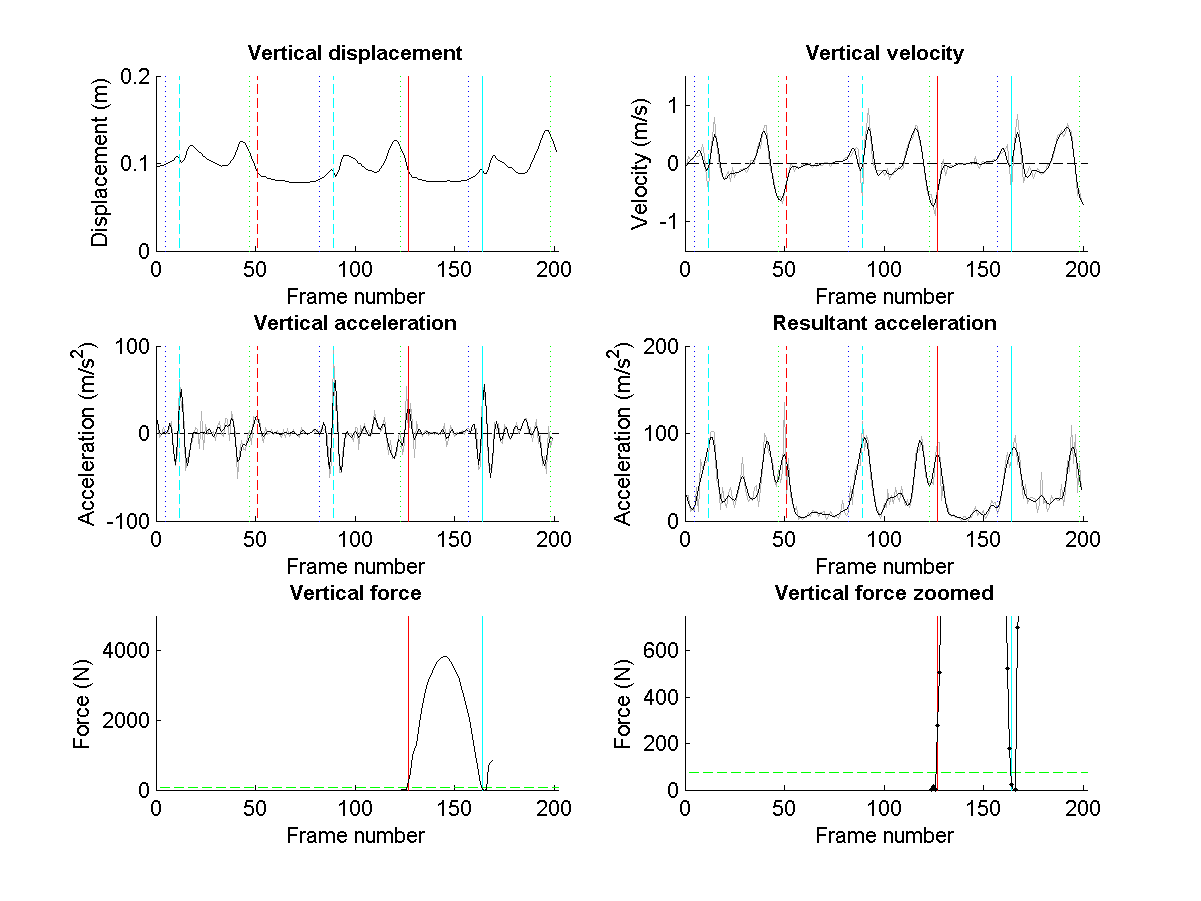

Supplement: Supplemental Information 3 [file peerj-03-783-s003.zip › Suppl figures/Event-based/Event_plot-RF_Horse9_circle_right_trot_04.png]

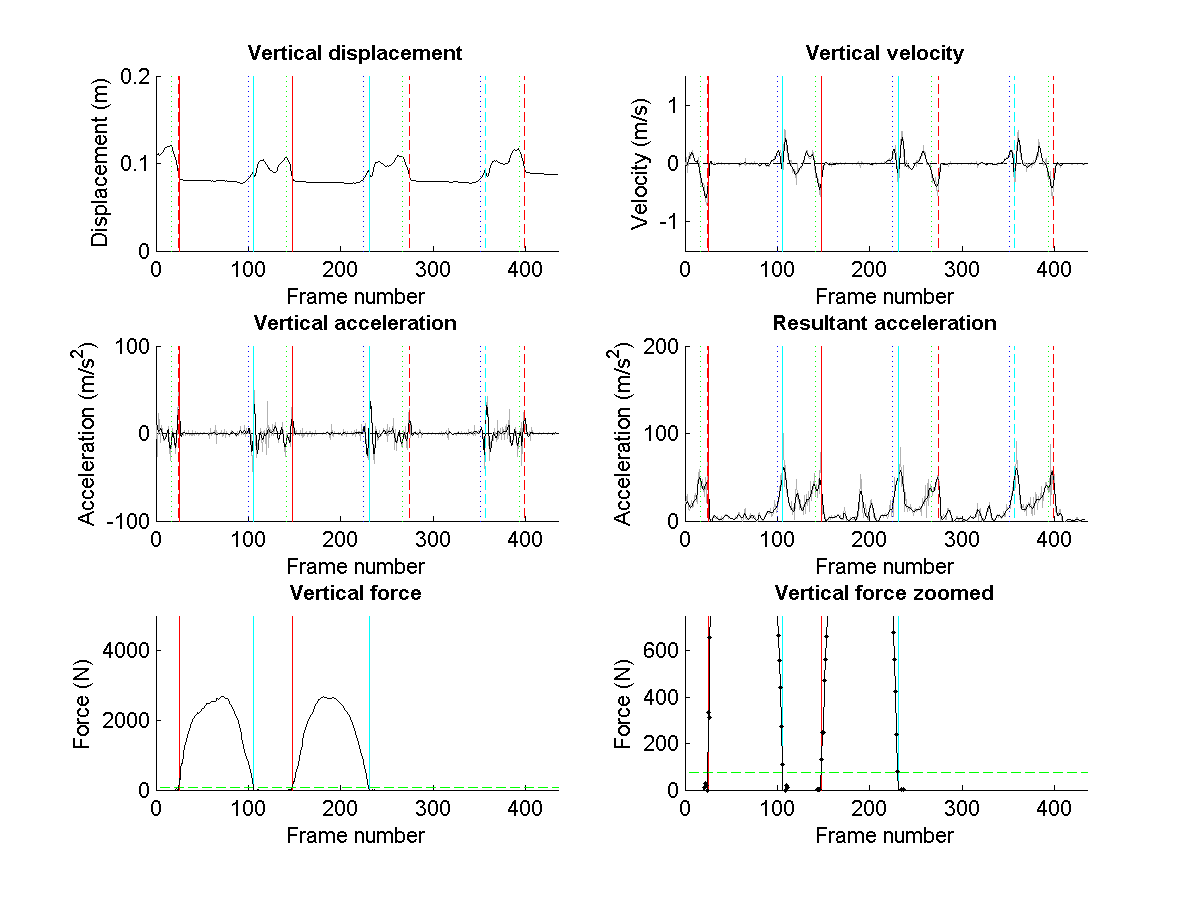

Supplement: Supplemental Information 3 [file peerj-03-783-s003.zip › Suppl figures/Event-based/Event_plot-RF_Horse9_circle_right_walk_08.png]

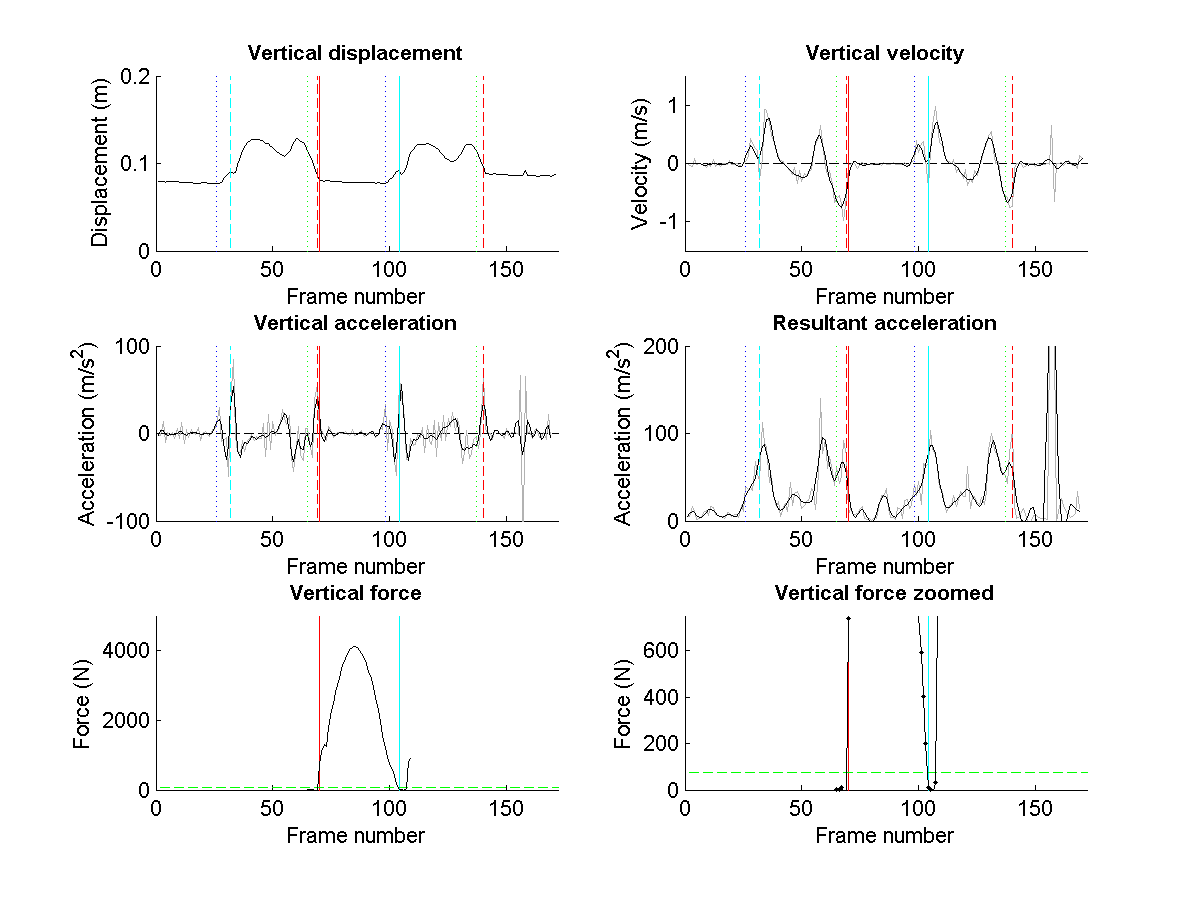

Supplement: Supplemental Information 3 [file peerj-03-783-s003.zip › Suppl figures/Event-based/Event_plot-RF_Horse9_trot_03.png]

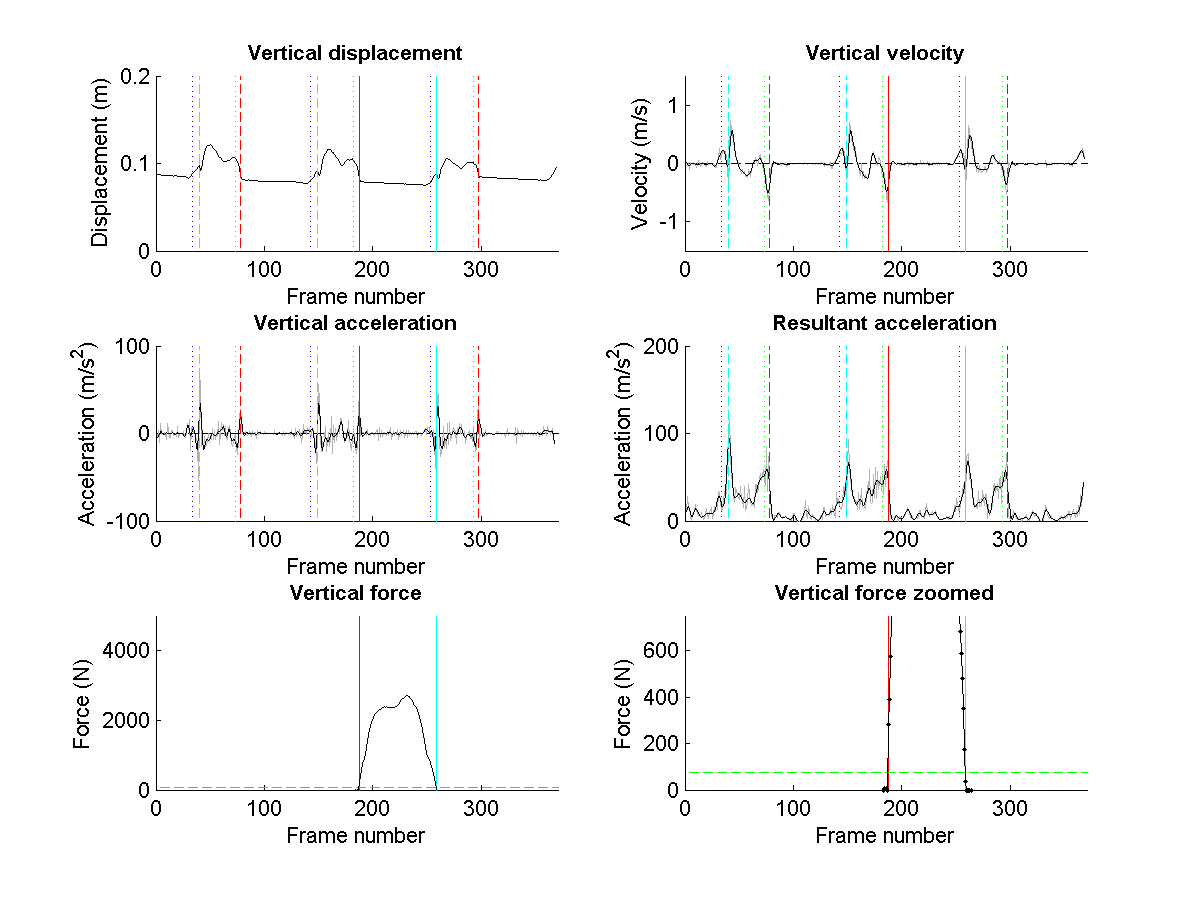

Supplement: Supplemental Information 3 [file peerj-03-783-s003.zip › Suppl figures/Event-based/Event_plot-RF_Horse9_walk_01.png]

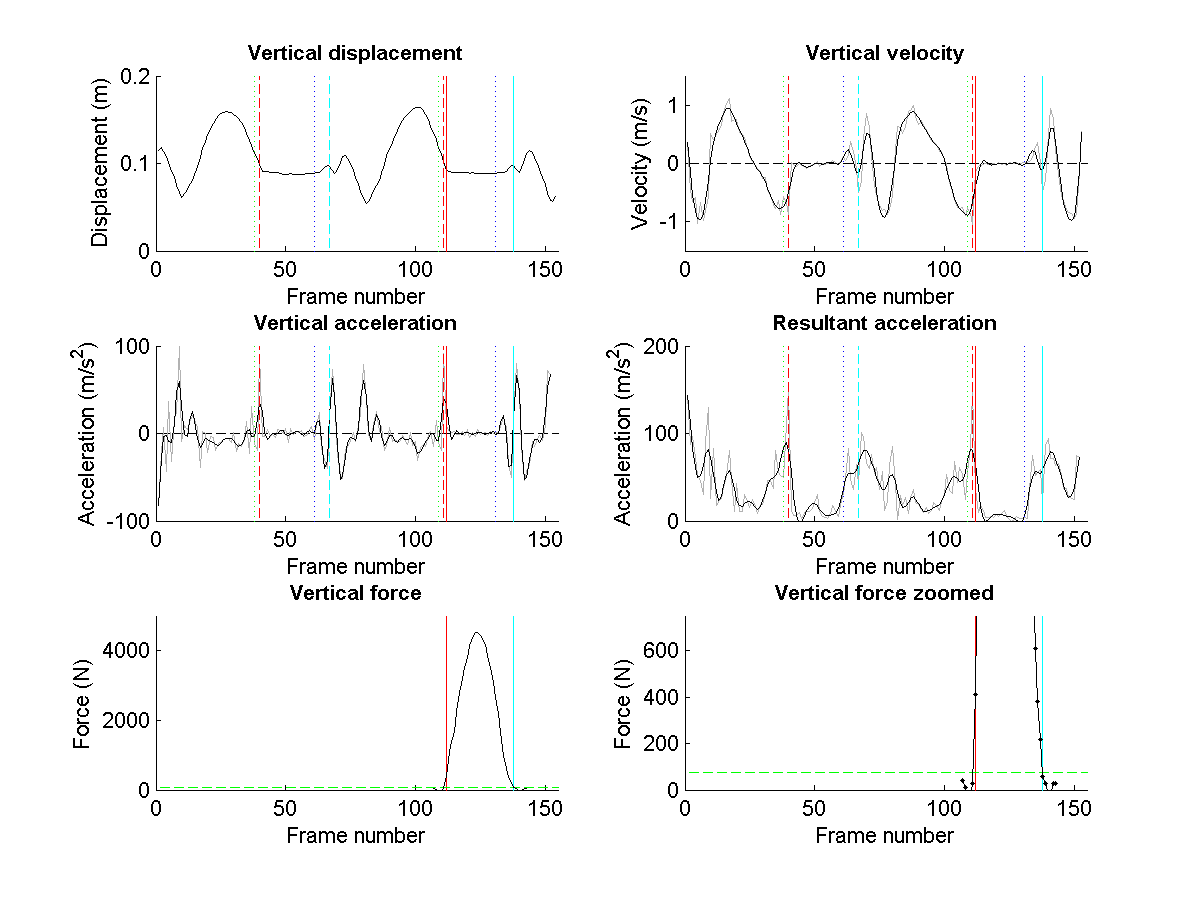

Supplement: Supplemental Information 3 [file peerj-03-783-s003.zip › Suppl figures/Event-based/Event_plot-RH_Horse11_trot_04.png]

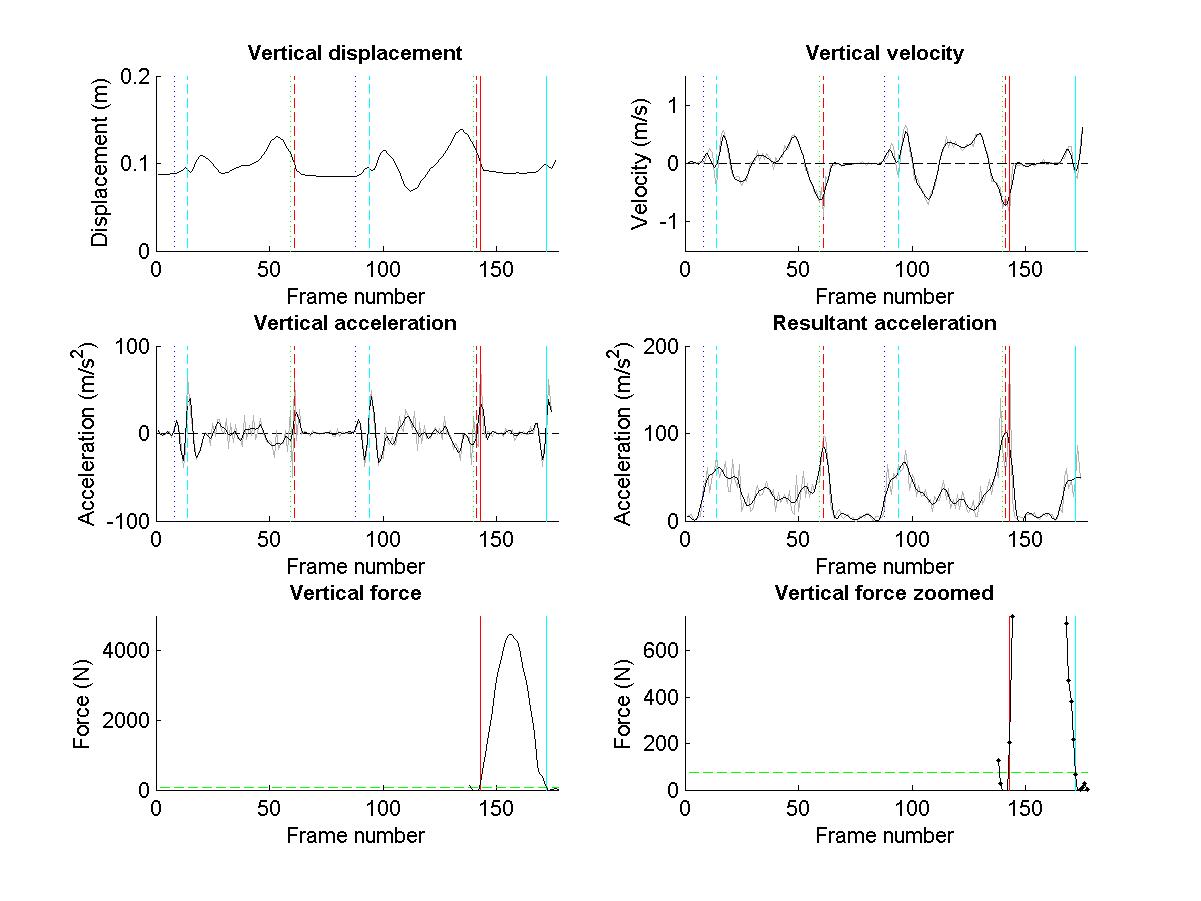

Supplement: Supplemental Information 3 [file peerj-03-783-s003.zip › Suppl figures/Event-based/Event_plot-RH_Horse11_trot_circle_left_06.png]

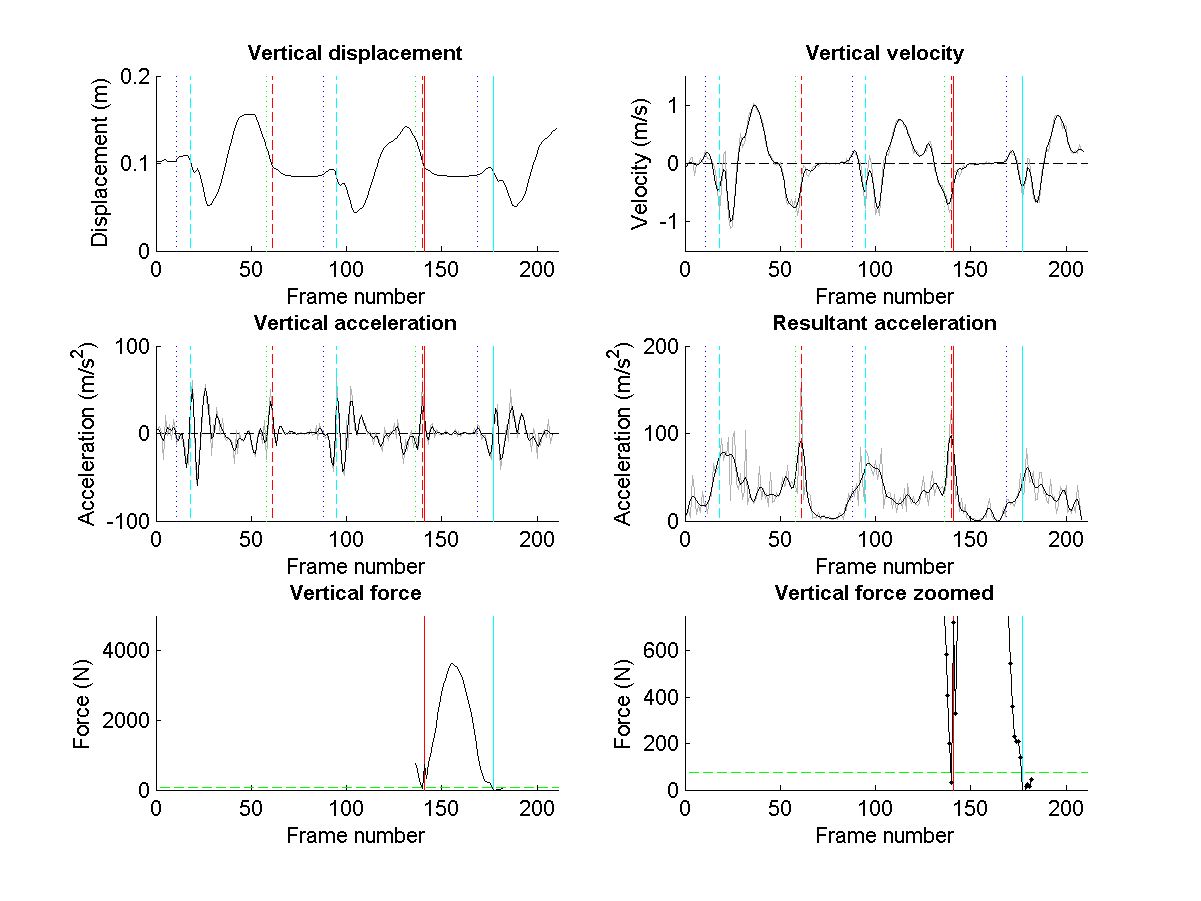

Supplement: Supplemental Information 3 [file peerj-03-783-s003.zip › Suppl figures/Event-based/Event_plot-RH_Horse11_trot_circle_right_04.png]

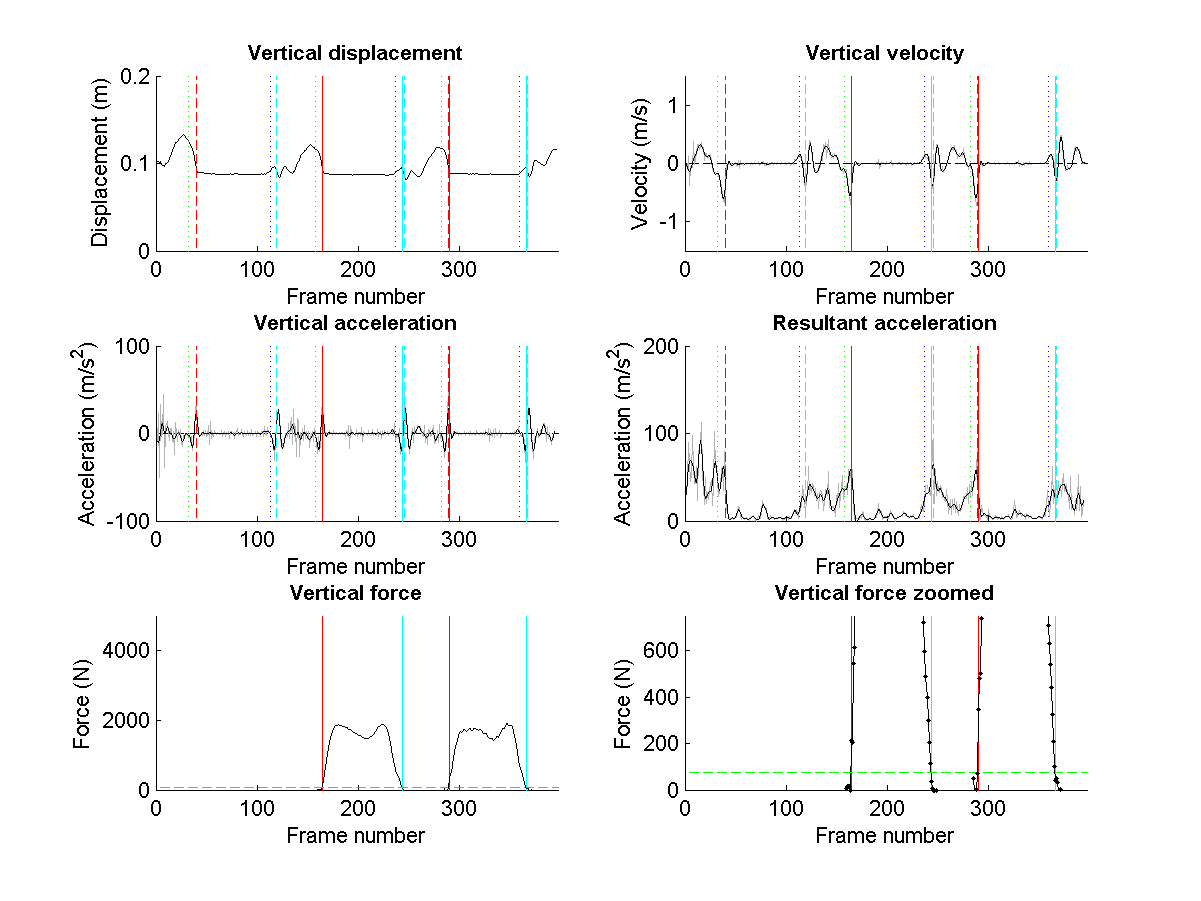

Supplement: Supplemental Information 3 [file peerj-03-783-s003.zip › Suppl figures/Event-based/Event_plot-RH_Horse11_walk_03.png]

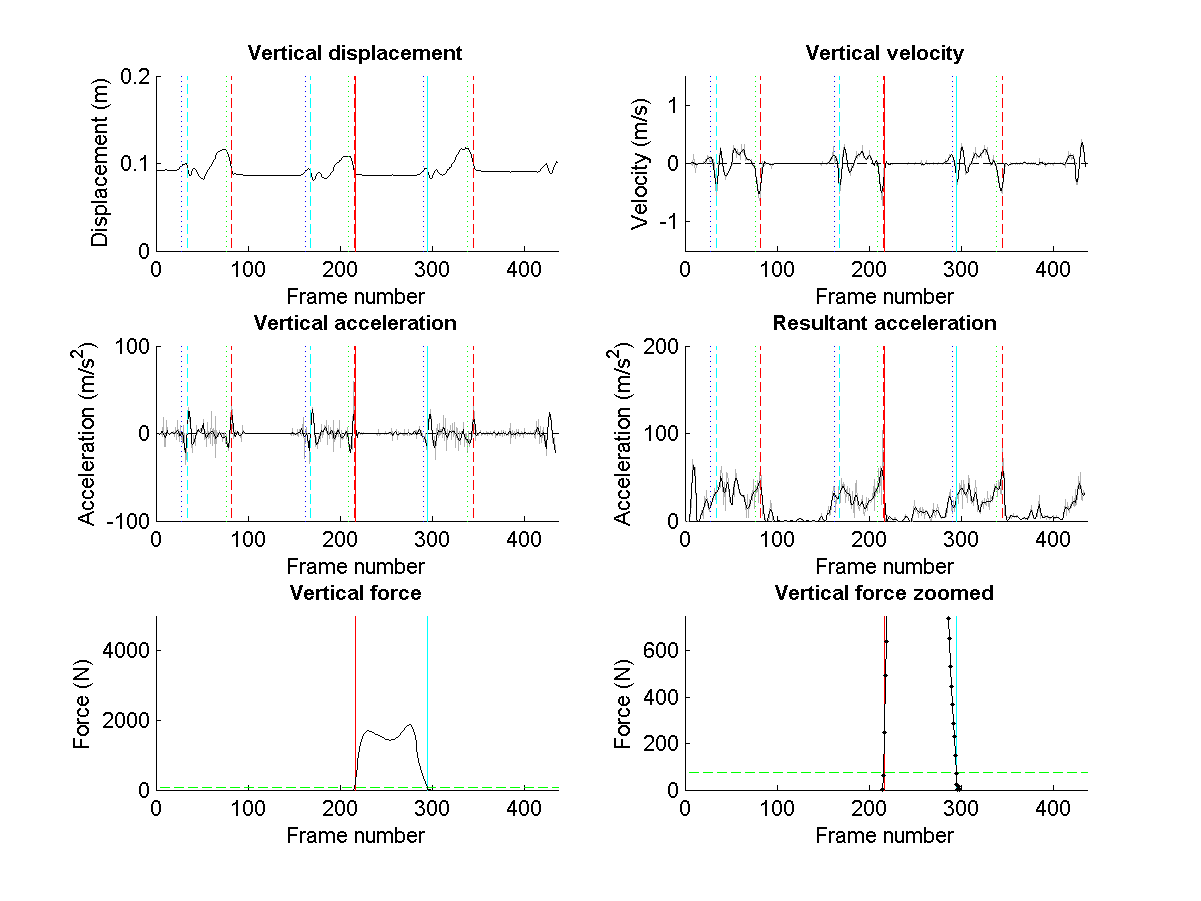

Supplement: Supplemental Information 3 [file peerj-03-783-s003.zip › Suppl figures/Event-based/Event_plot-RH_Horse11_walk_circle_left_06.png]

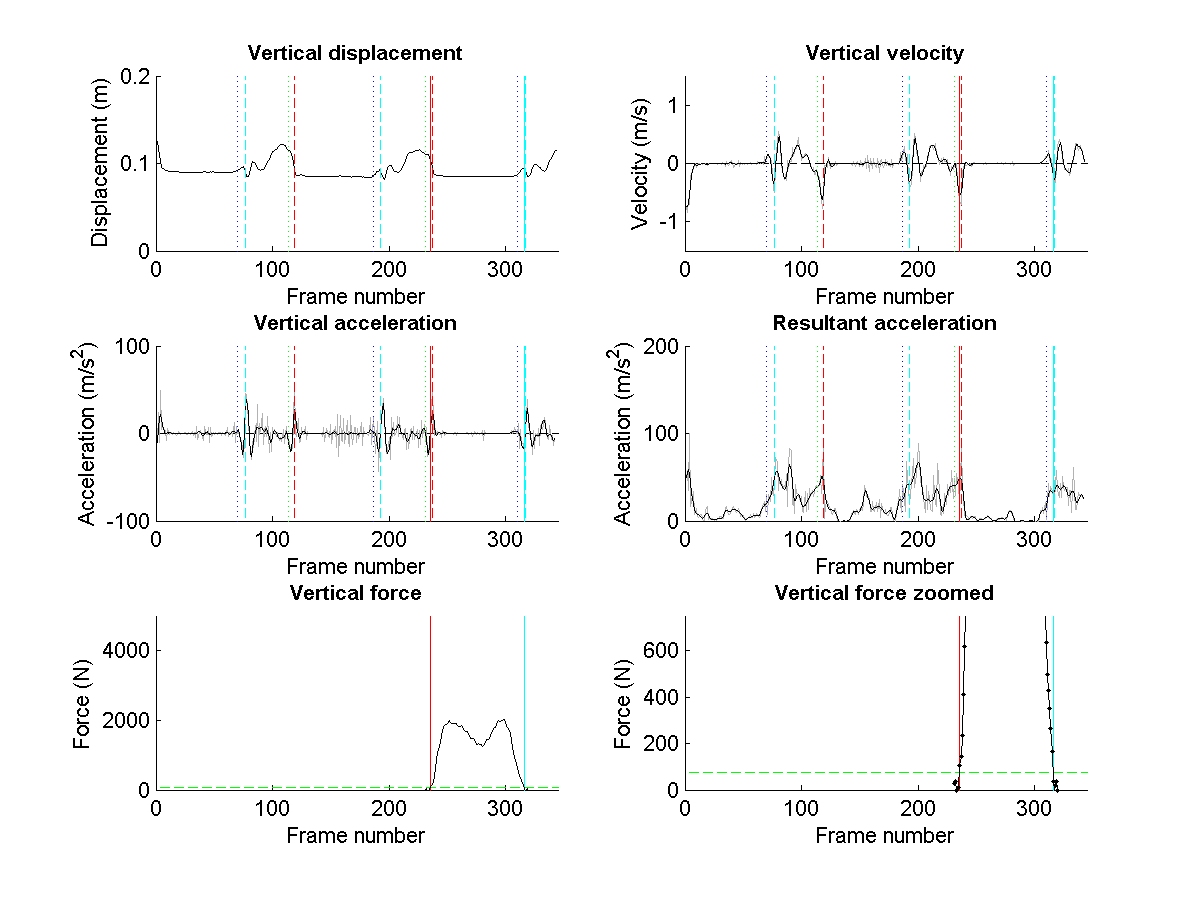

Supplement: Supplemental Information 3 [file peerj-03-783-s003.zip › Suppl figures/Event-based/Event_plot-RH_Horse11_walk_circle_right_06.png]

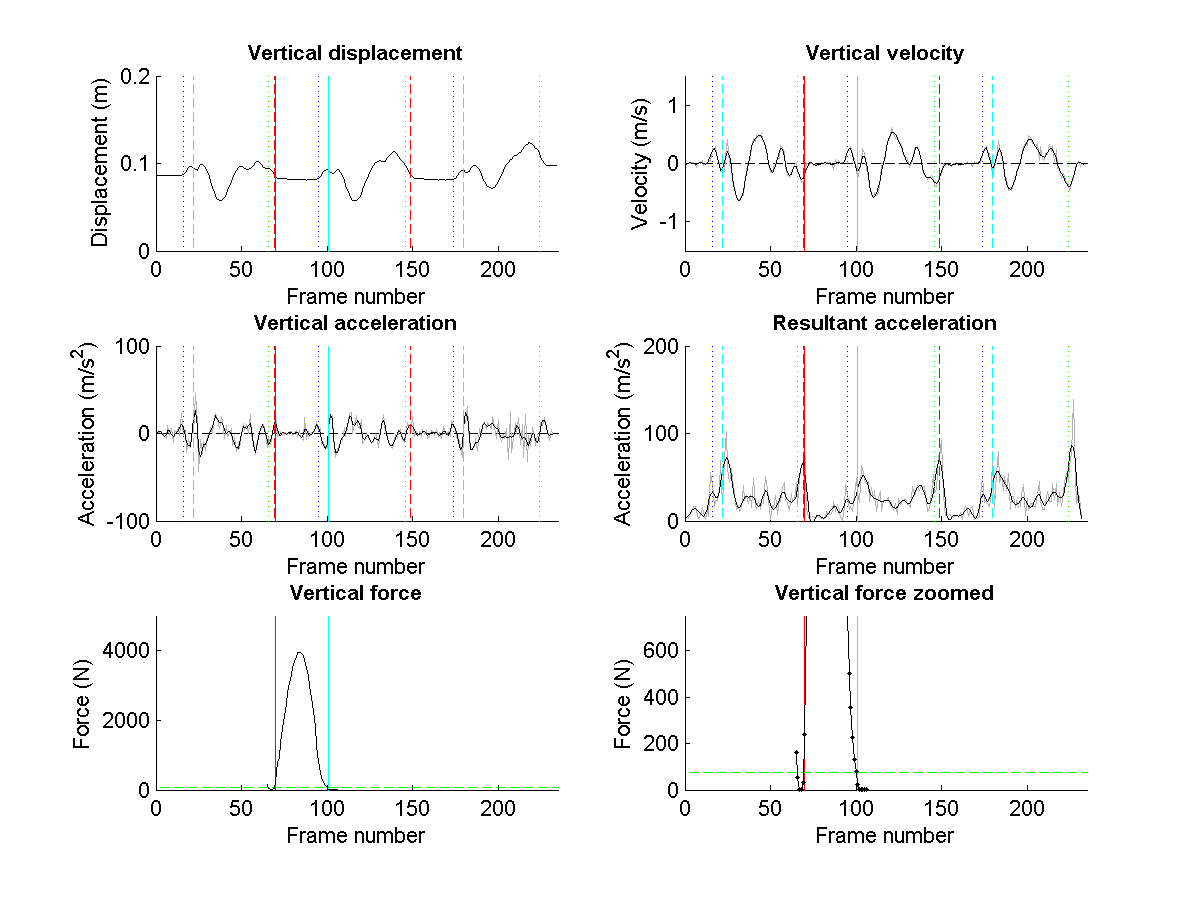

Supplement: Supplemental Information 3 [file peerj-03-783-s003.zip › Suppl figures/Event-based/Event_plot-RH_Horse4_circle_left_trot_07.png]

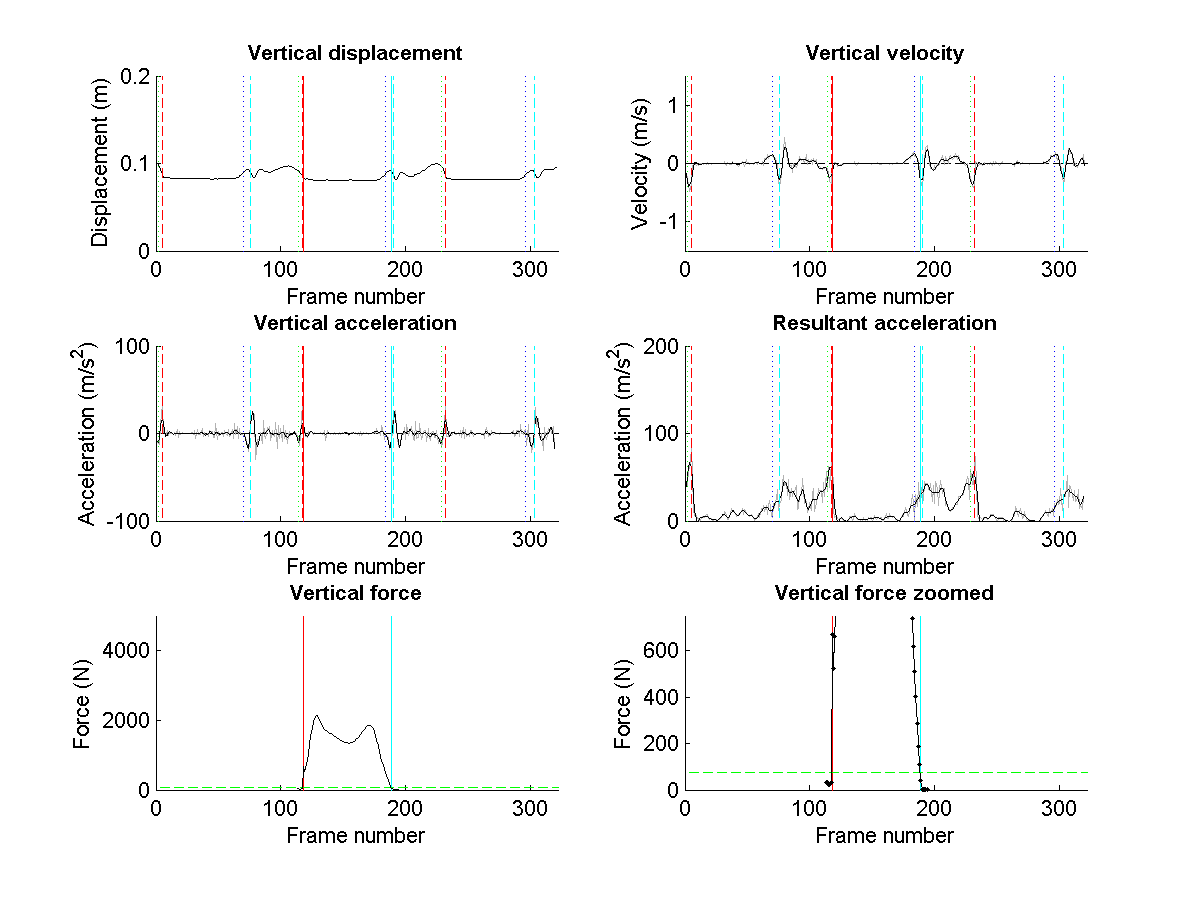

Supplement: Supplemental Information 3 [file peerj-03-783-s003.zip › Suppl figures/Event-based/Event_plot-RH_Horse4_circle_left_walk04.png]

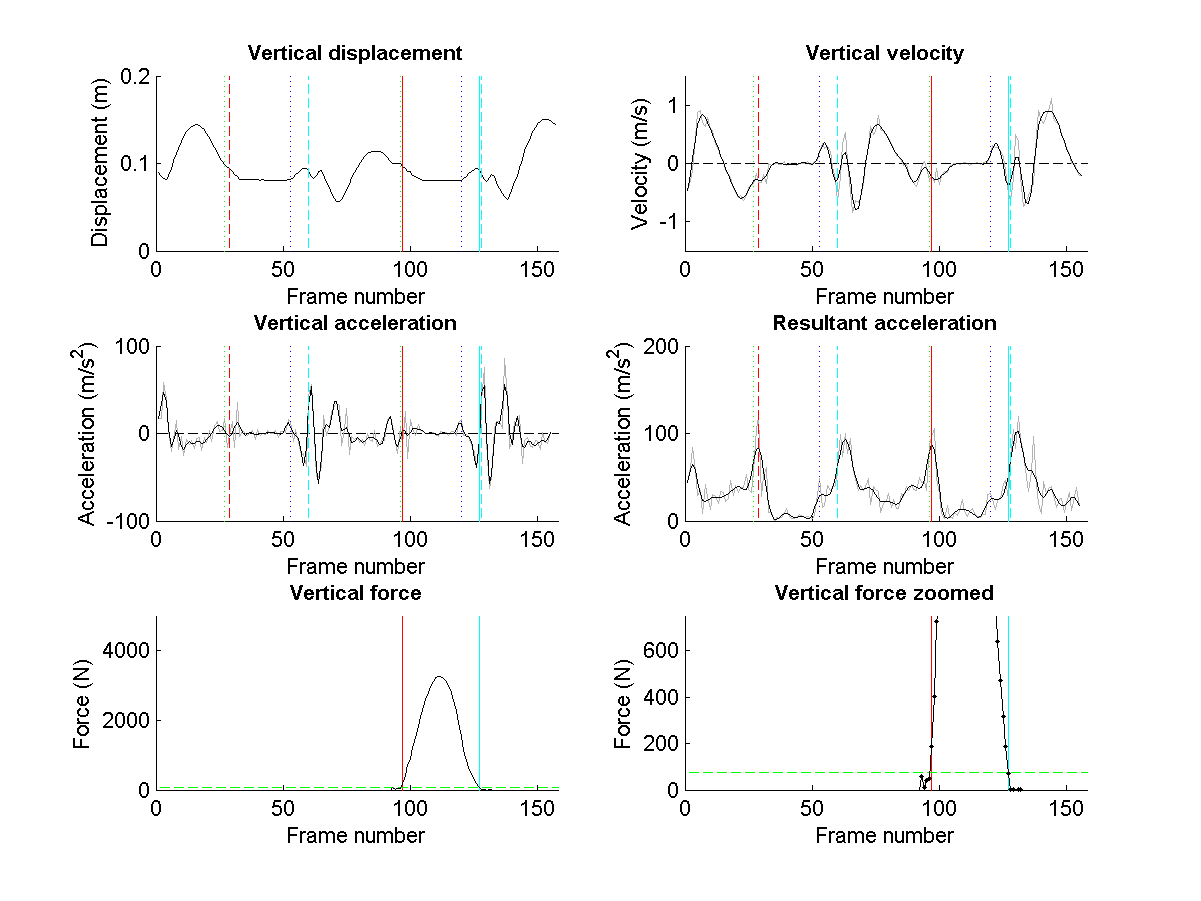

Supplement: Supplemental Information 3 [file peerj-03-783-s003.zip › Suppl figures/Event-based/Event_plot-RH_Horse4_circle_right_trot05.png]

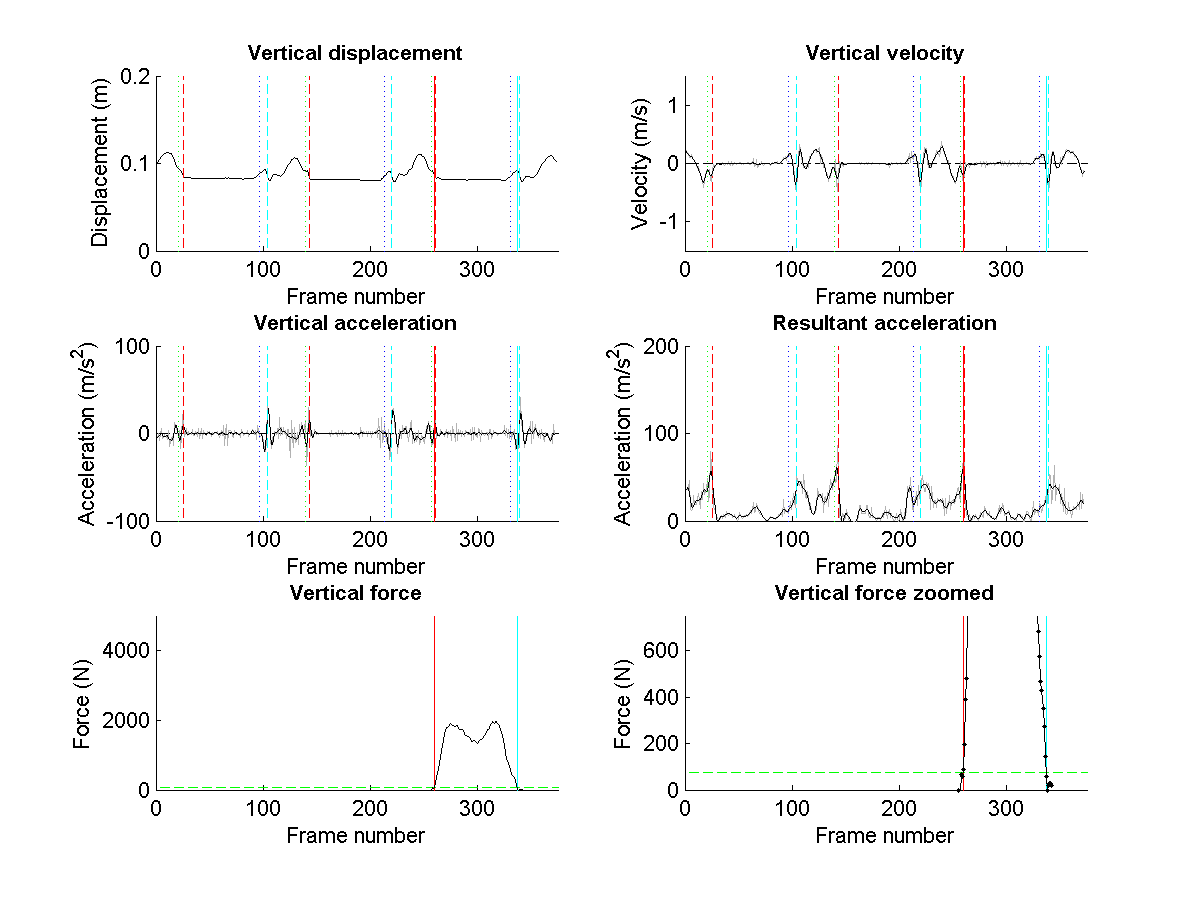

Supplement: Supplemental Information 3 [file peerj-03-783-s003.zip › Suppl figures/Event-based/Event_plot-RH_Horse4_circle_right_walk_01.png]

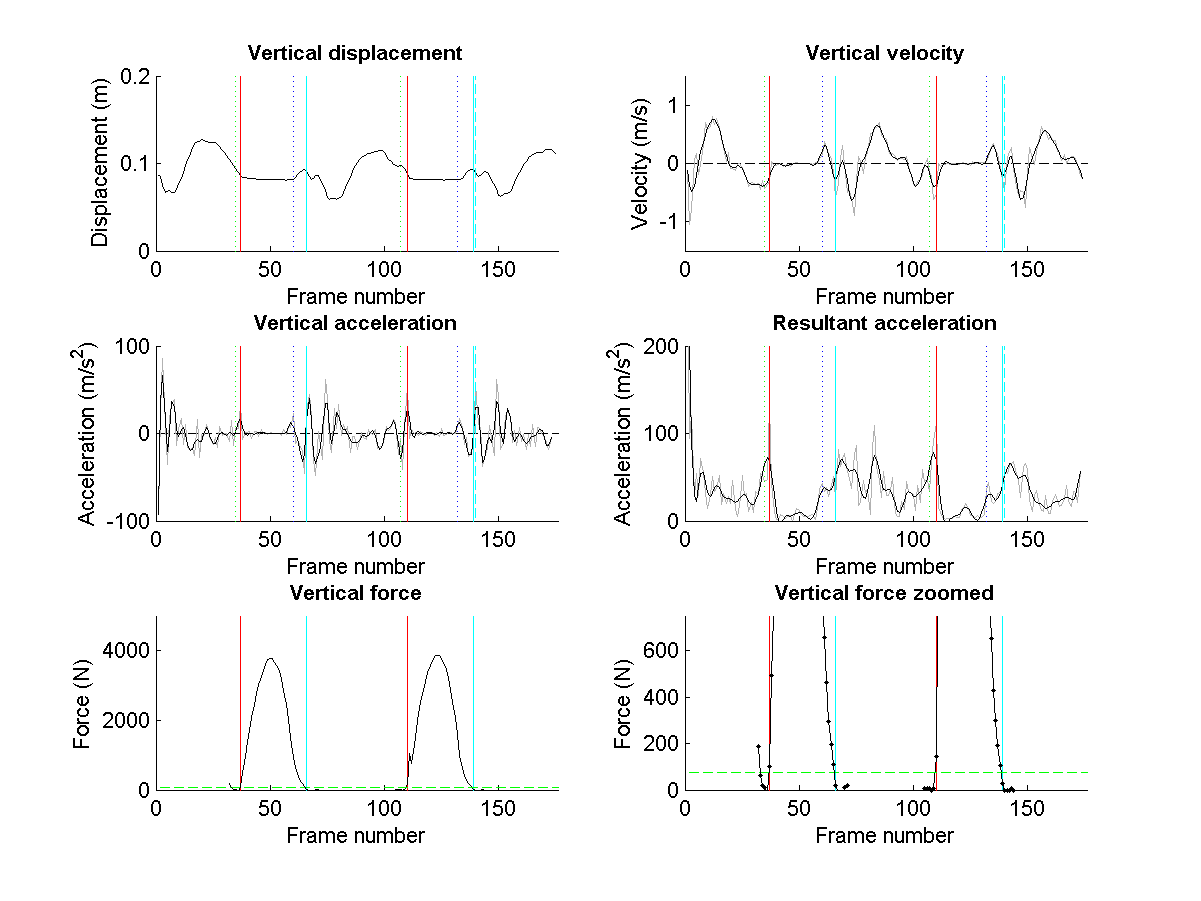

Supplement: Supplemental Information 3 [file peerj-03-783-s003.zip › Suppl figures/Event-based/Event_plot-RH_Horse4_trot_01.png]

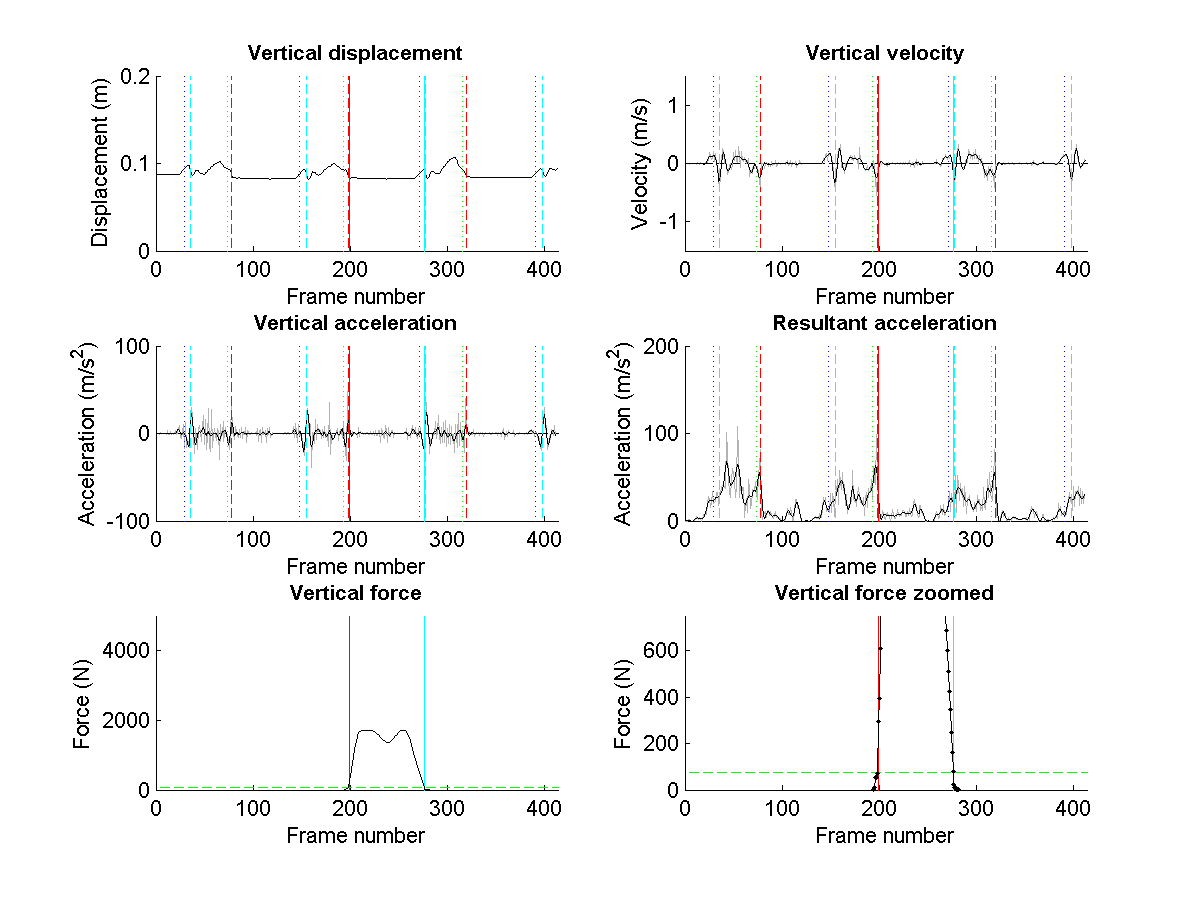

Supplement: Supplemental Information 3 [file peerj-03-783-s003.zip › Suppl figures/Event-based/Event_plot-RH_Horse4_walk_07.png]

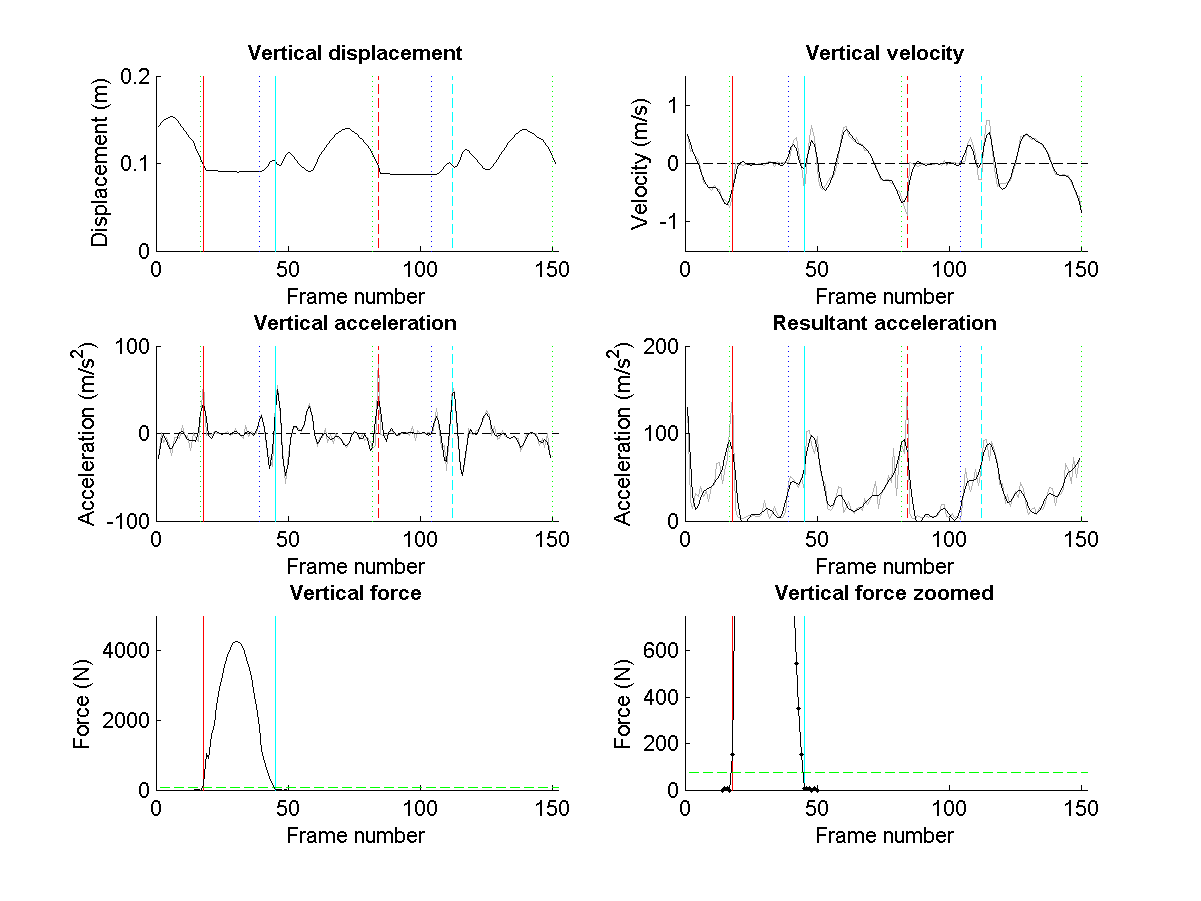

Supplement: Supplemental Information 3 [file peerj-03-783-s003.zip › Suppl figures/Event-based/Event_plot-RH_Horse6_trot_01.png]

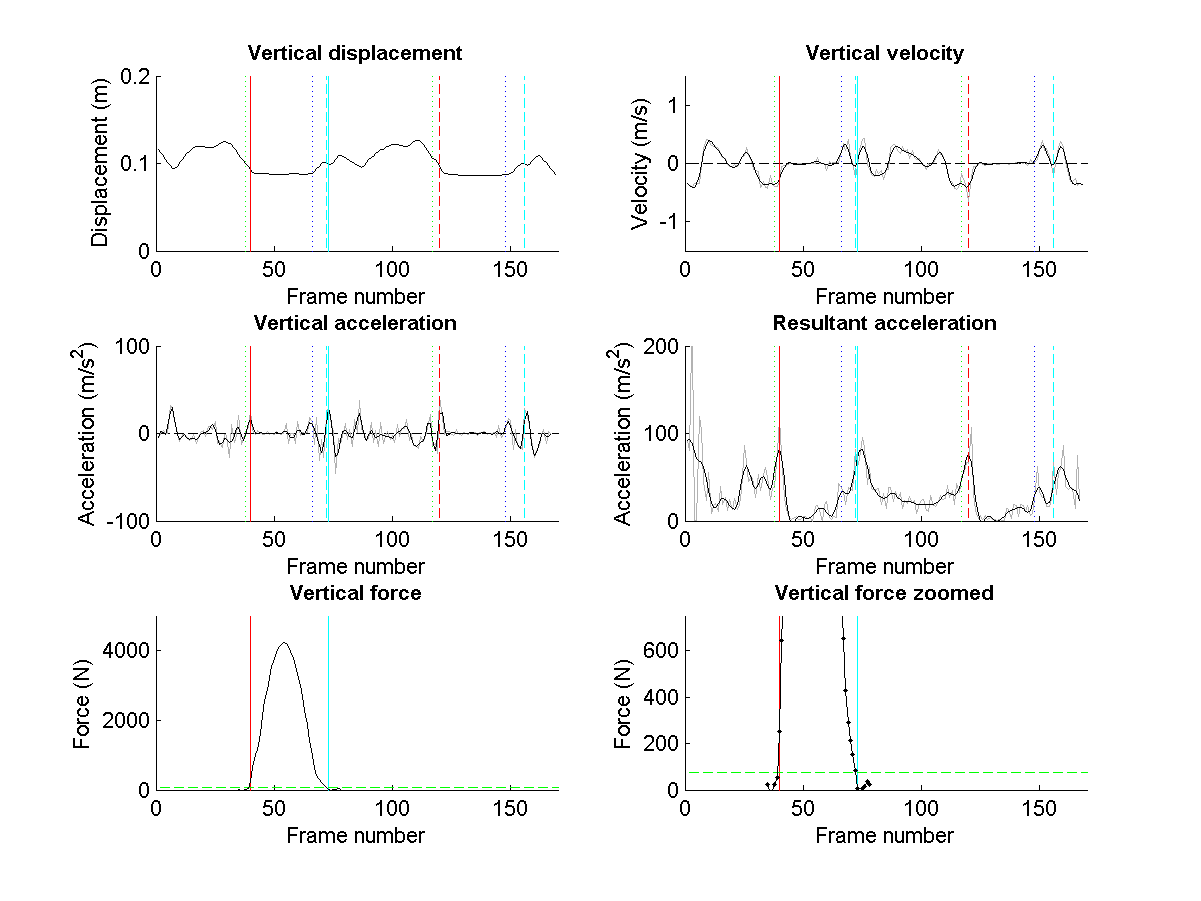

Supplement: Supplemental Information 3 [file peerj-03-783-s003.zip › Suppl figures/Event-based/Event_plot-RH_Horse6_trot_circle_left_04.png]

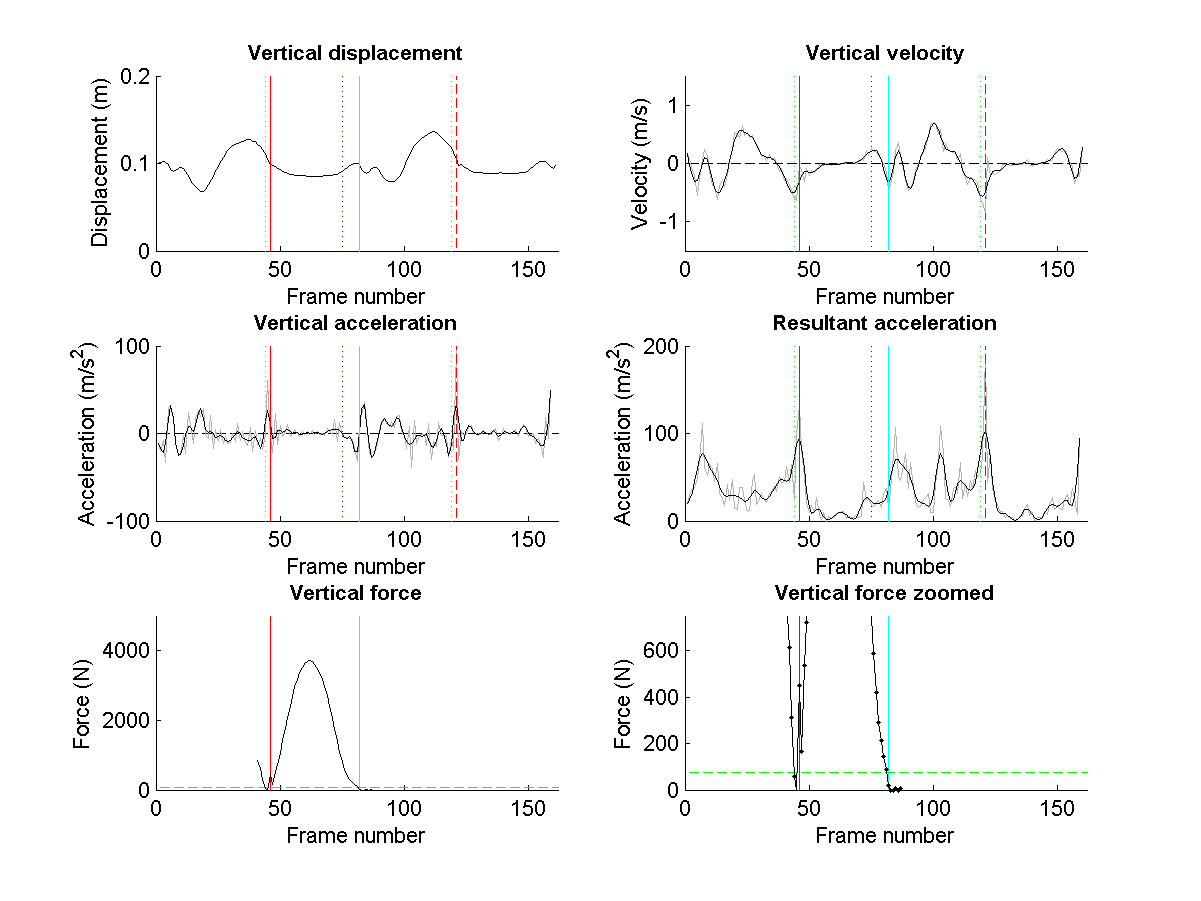

Supplement: Supplemental Information 3 [file peerj-03-783-s003.zip › Suppl figures/Event-based/Event_plot-RH_Horse6_trot_circle_right_02.png]

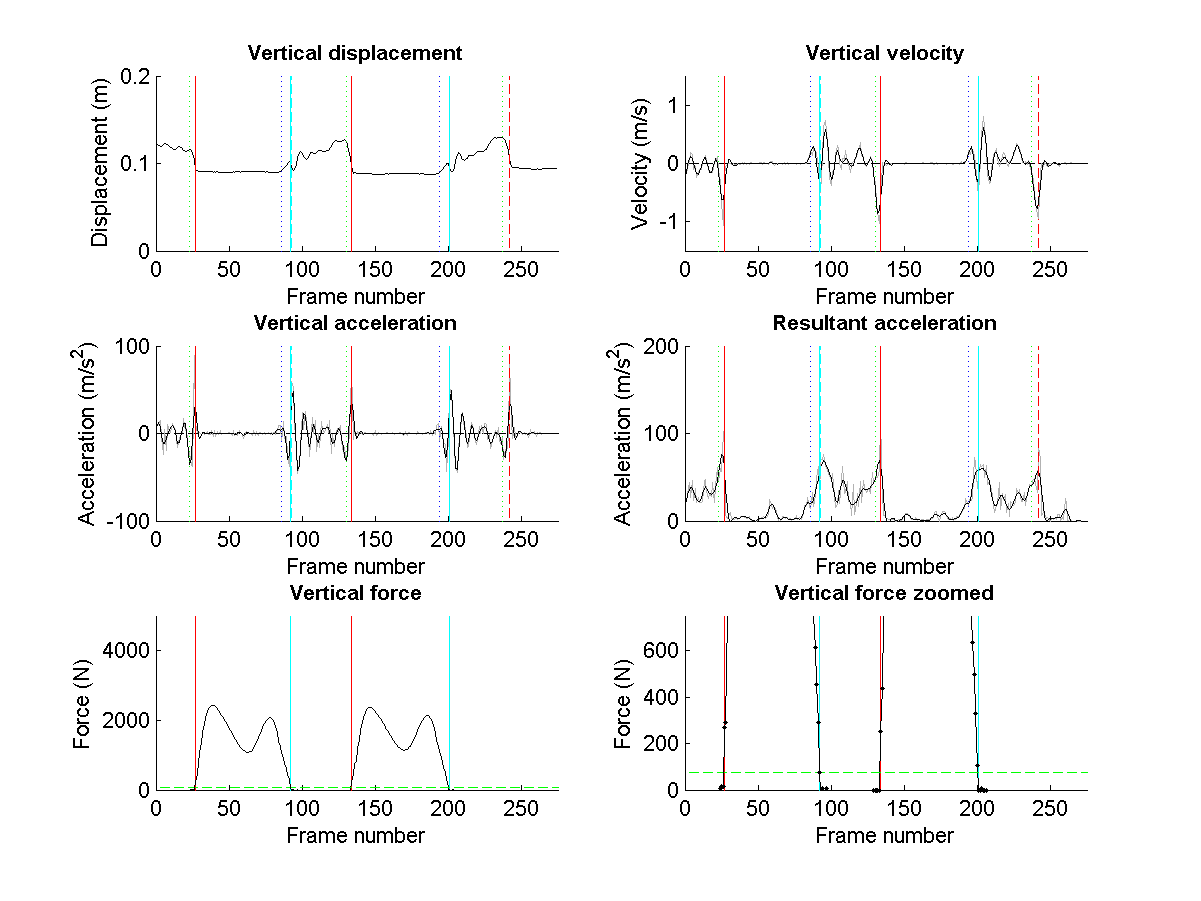

Supplement: Supplemental Information 3 [file peerj-03-783-s003.zip › Suppl figures/Event-based/Event_plot-RH_Horse6_walk_05.png]

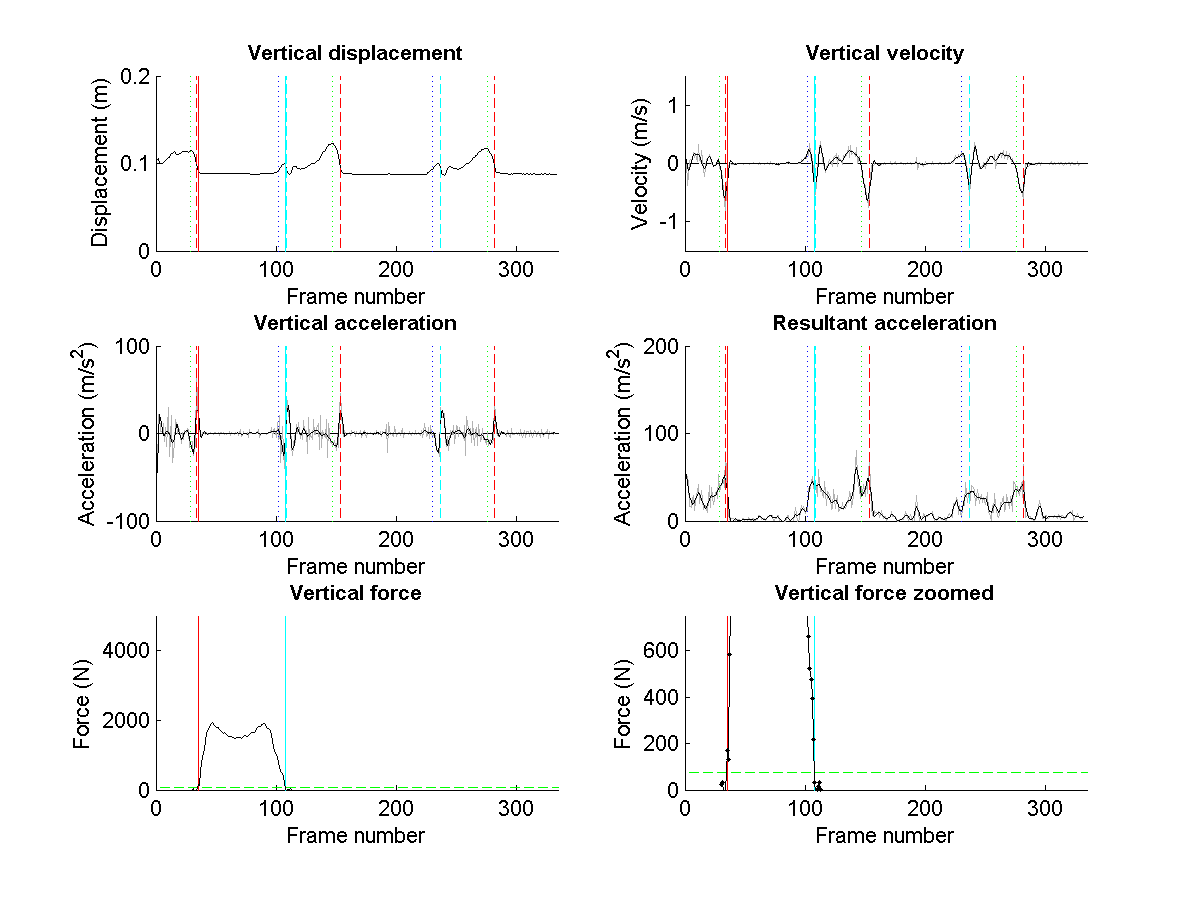

Supplement: Supplemental Information 3 [file peerj-03-783-s003.zip › Suppl figures/Event-based/Event_plot-RH_Horse6_walk_circle_left_05.png]

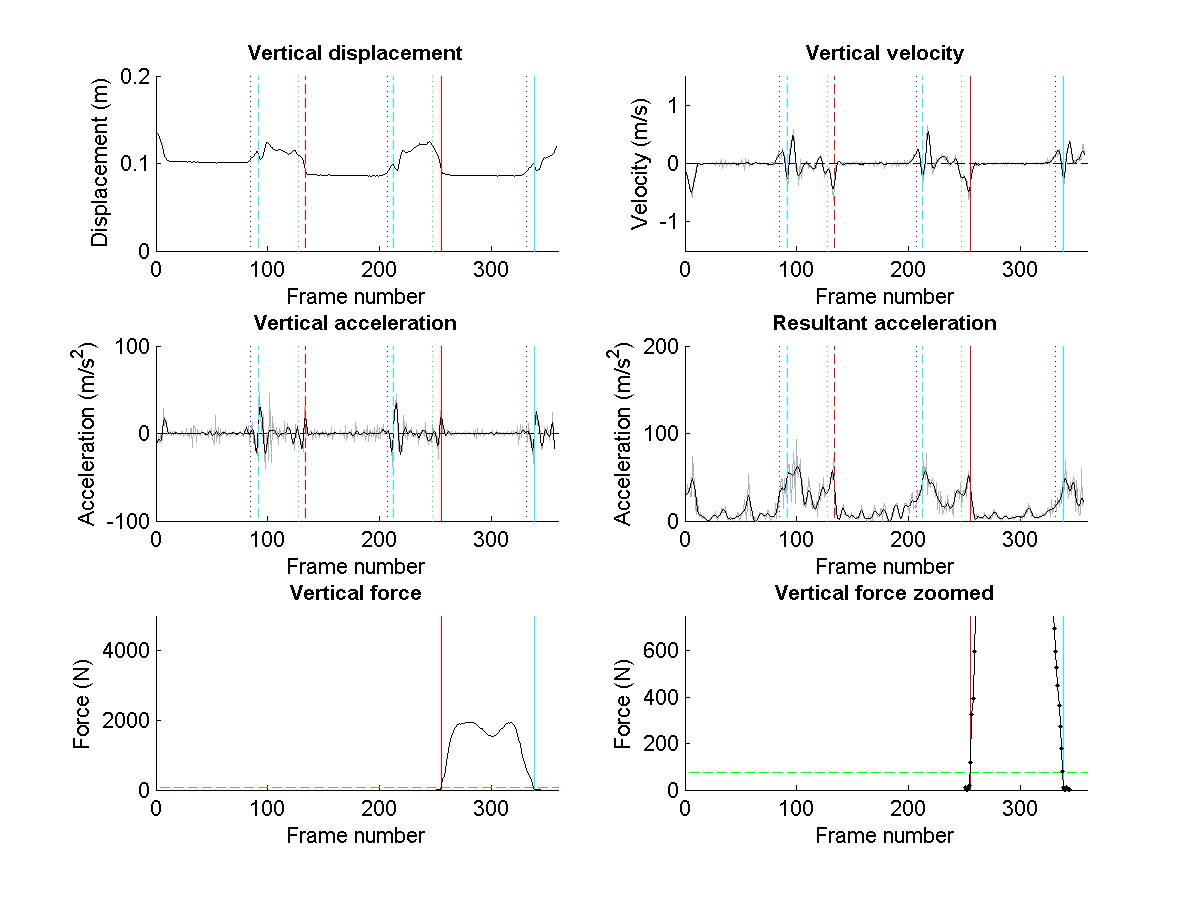

Supplement: Supplemental Information 3 [file peerj-03-783-s003.zip › Suppl figures/Event-based/Event_plot-RH_Horse6_walk_circle_right_14.png]

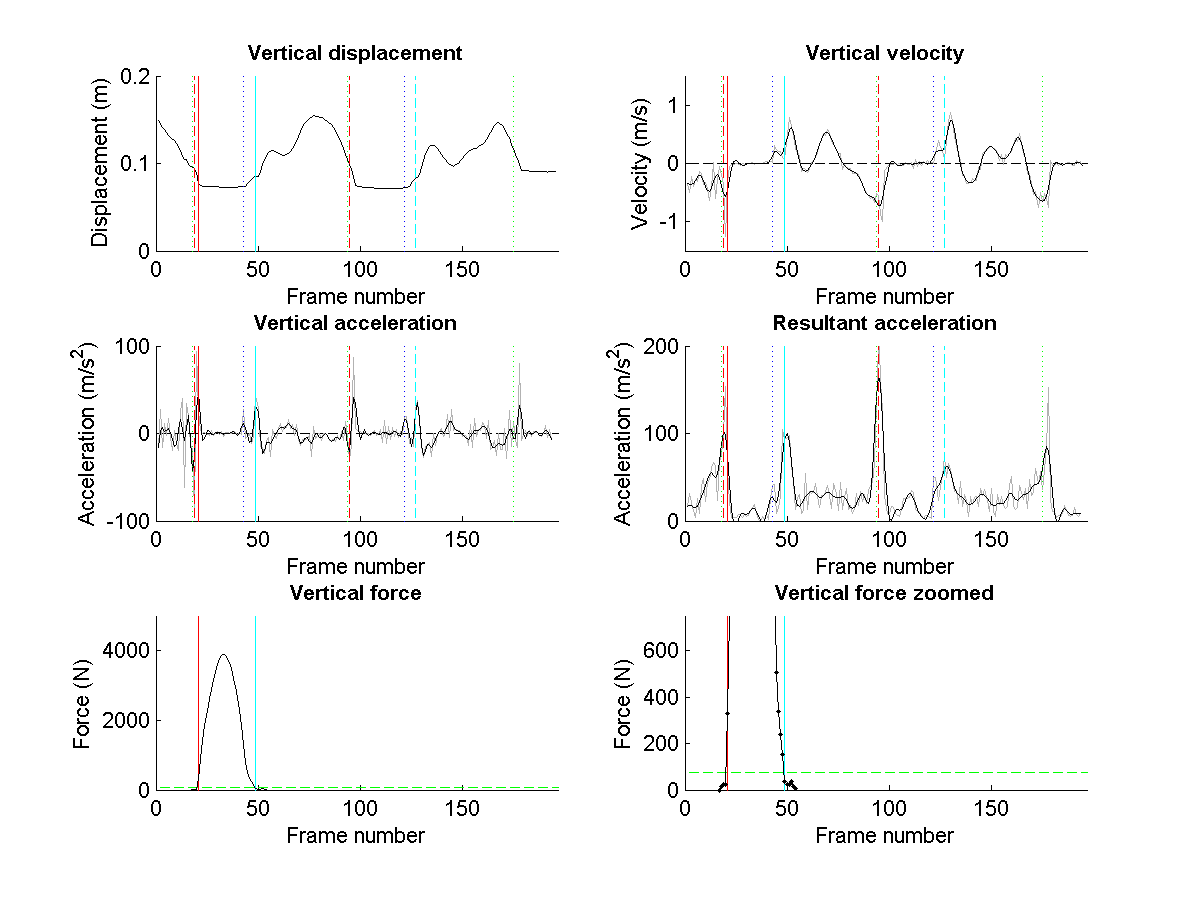

Supplement: Supplemental Information 3 [file peerj-03-783-s003.zip › Suppl figures/Event-based/Event_plot-RH_Horse9_circle_left_trot_11.png]

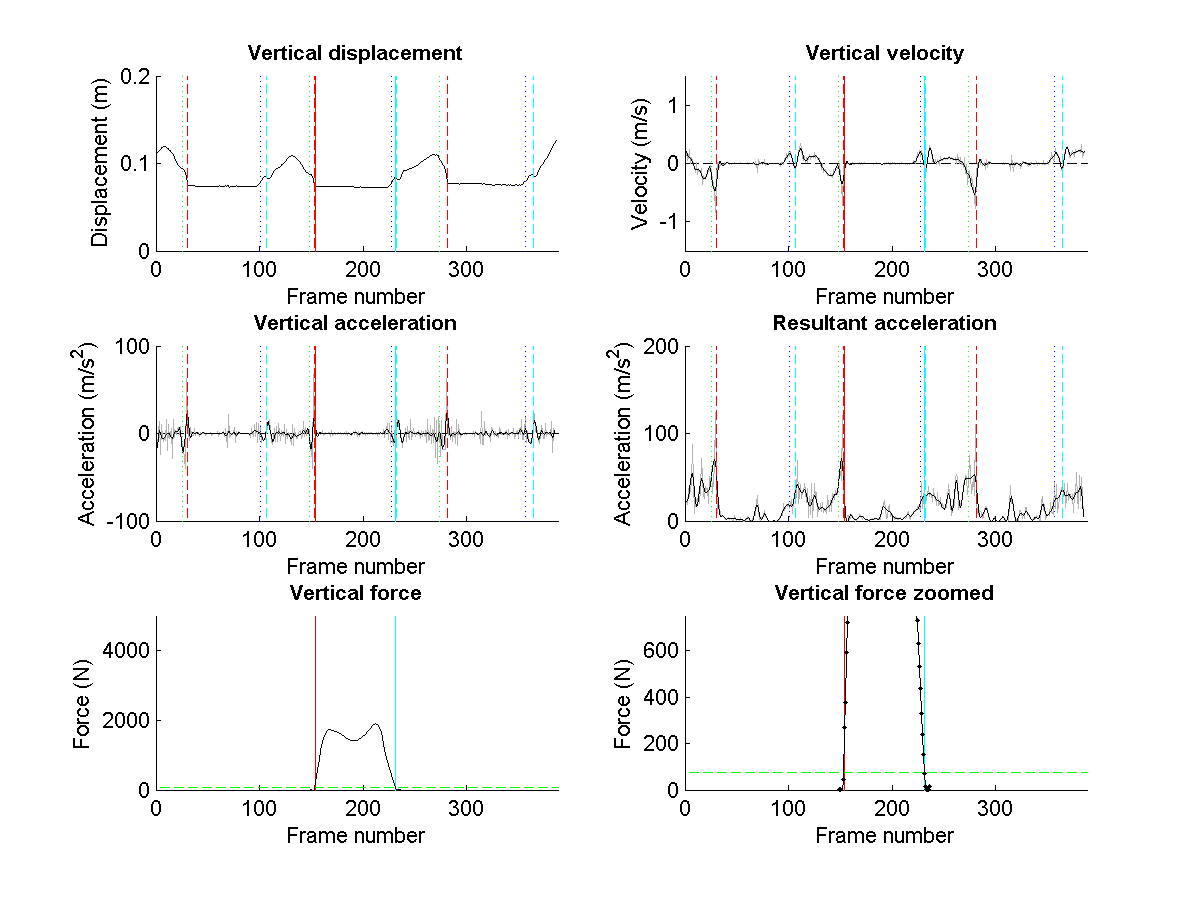

Supplement: Supplemental Information 3 [file peerj-03-783-s003.zip › Suppl figures/Event-based/Event_plot-RH_Horse9_circle_left_walk_11.png]

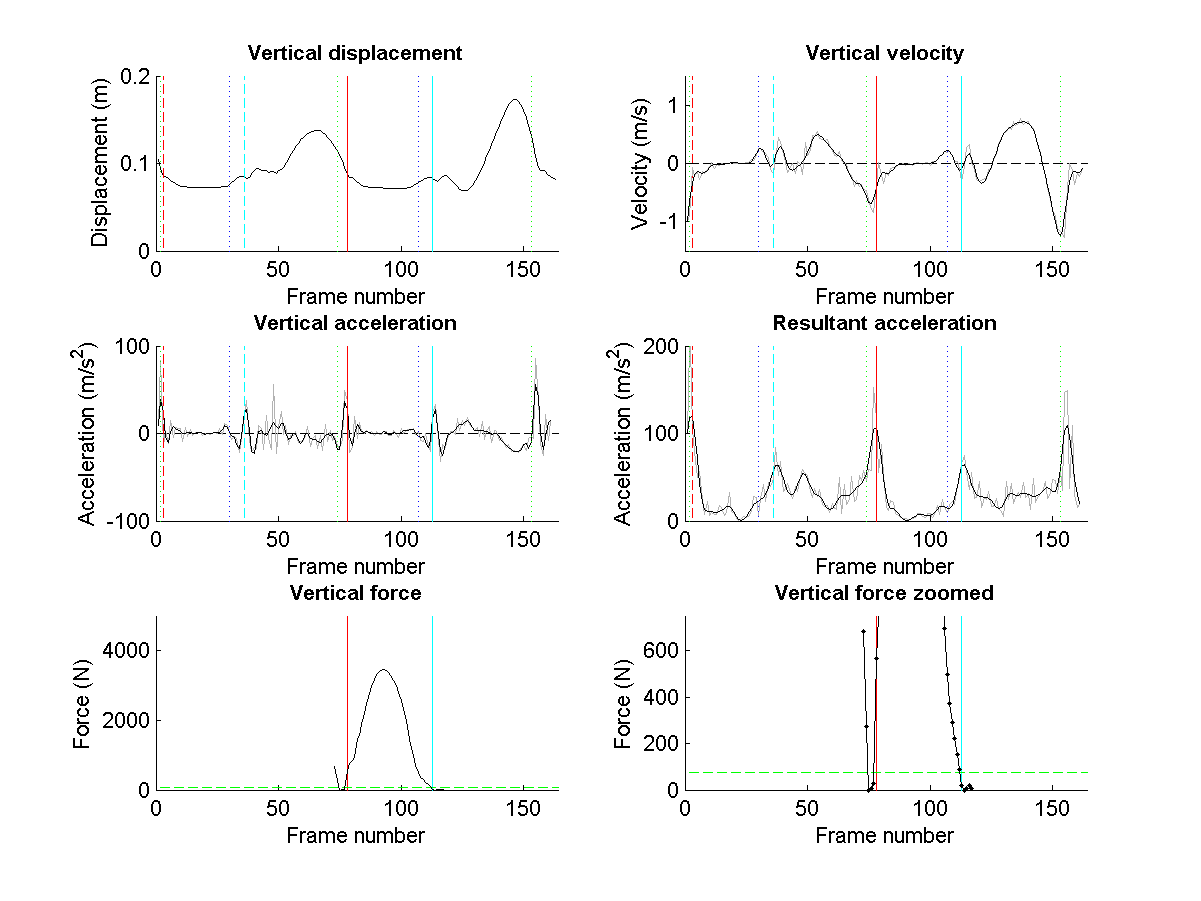

Supplement: Supplemental Information 3 [file peerj-03-783-s003.zip › Suppl figures/Event-based/Event_plot-RH_Horse9_circle_right_trot_02.png]

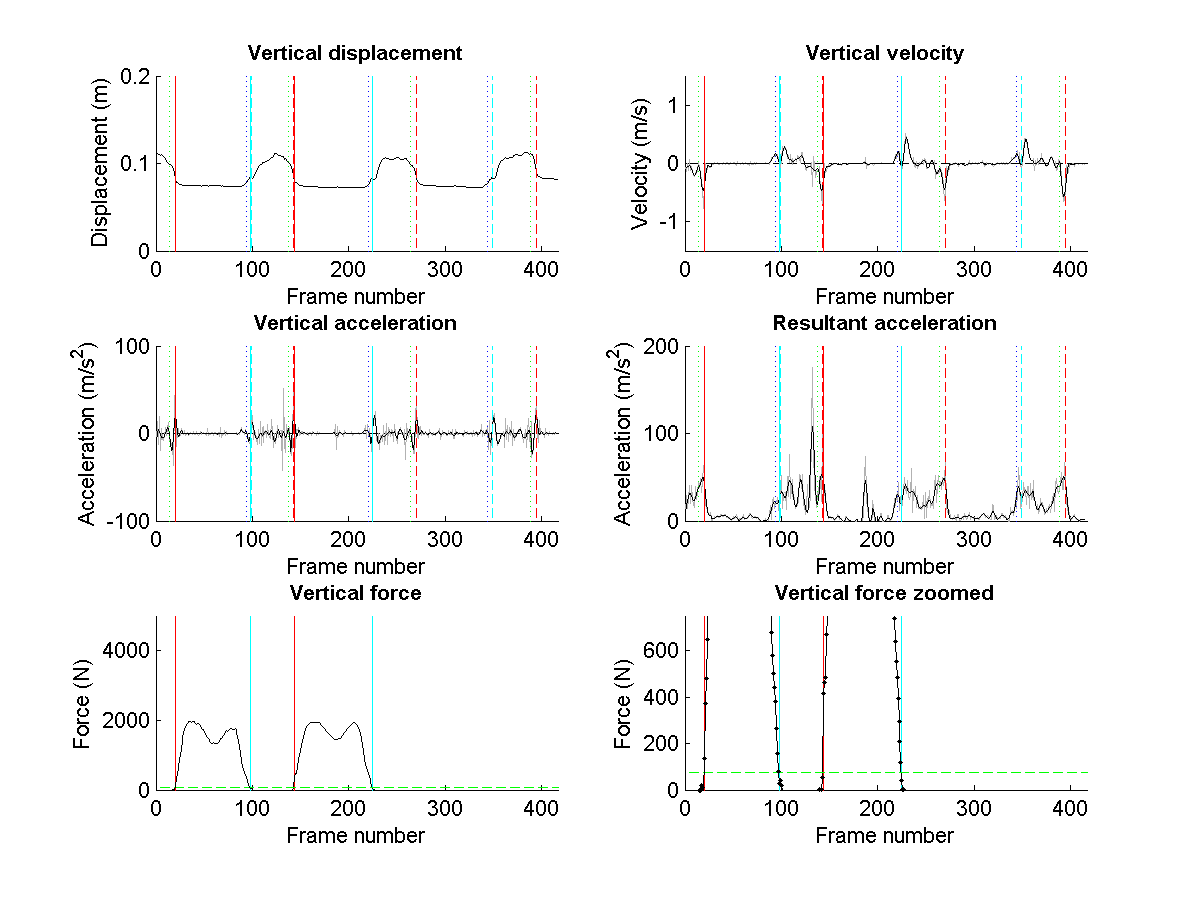

Supplement: Supplemental Information 3 [file peerj-03-783-s003.zip › Suppl figures/Event-based/Event_plot-RH_Horse9_circle_right_walk_08.png]

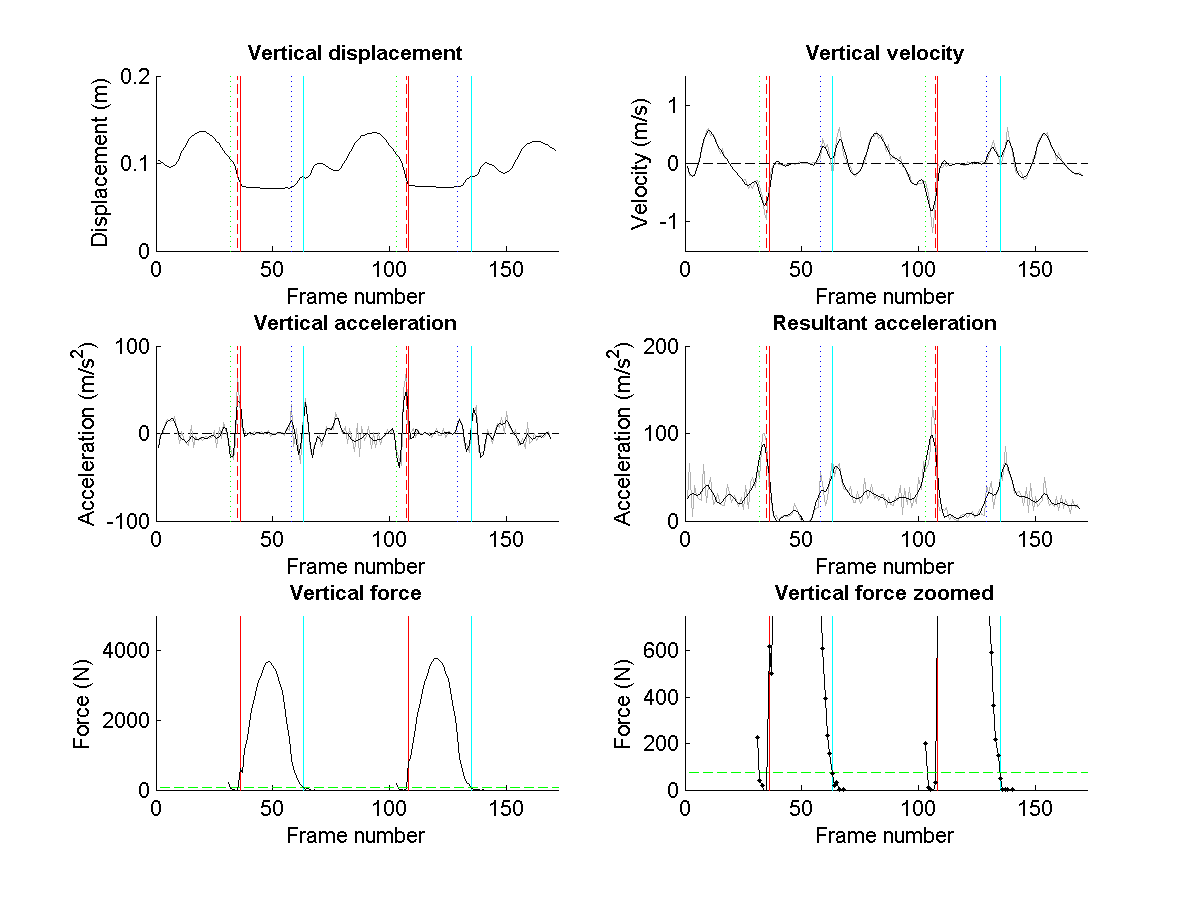

Supplement: Supplemental Information 3 [file peerj-03-783-s003.zip › Suppl figures/Event-based/Event_plot-RH_Horse9_trot_03.png]

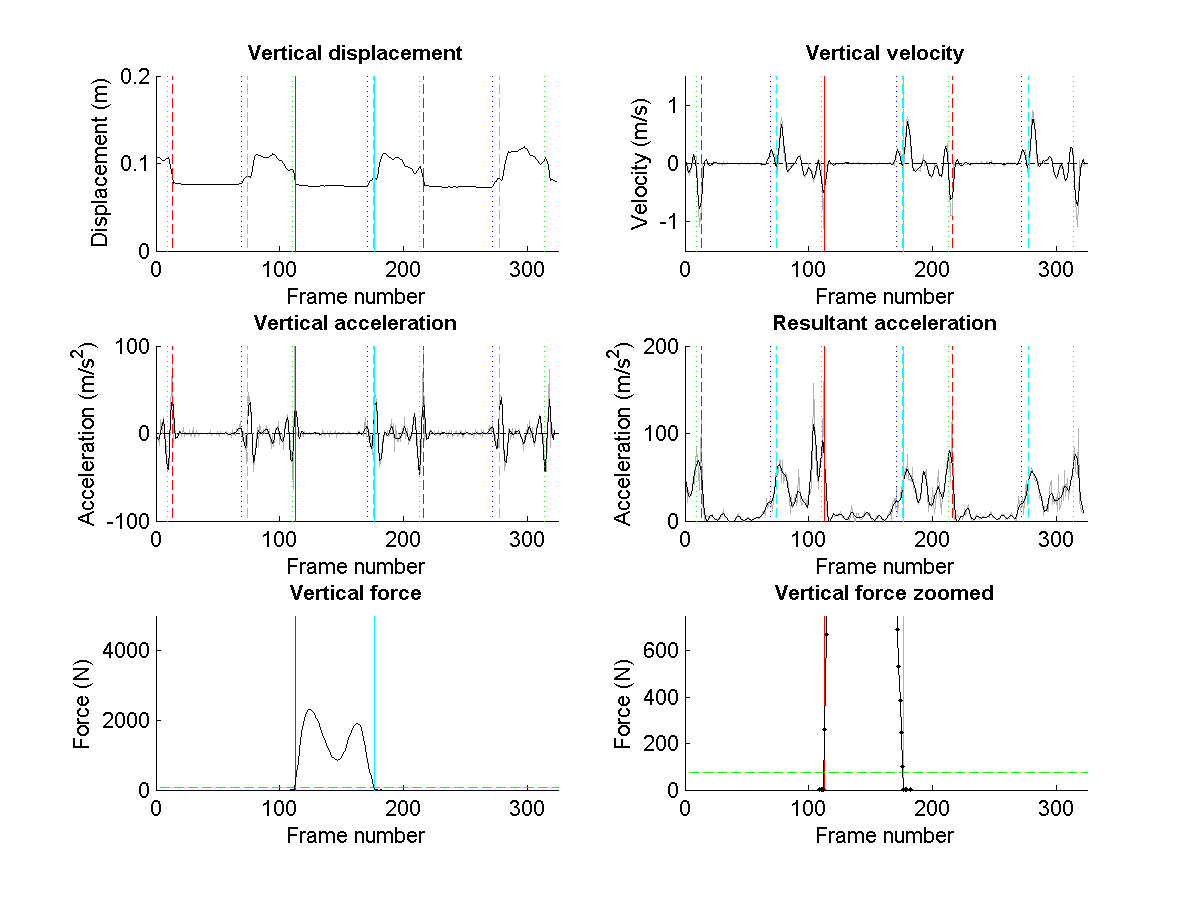

Supplement: Supplemental Information 3 [file peerj-03-783-s003.zip › Suppl figures/Event-based/Event_plot-RH_Horse9_walk_07.png]

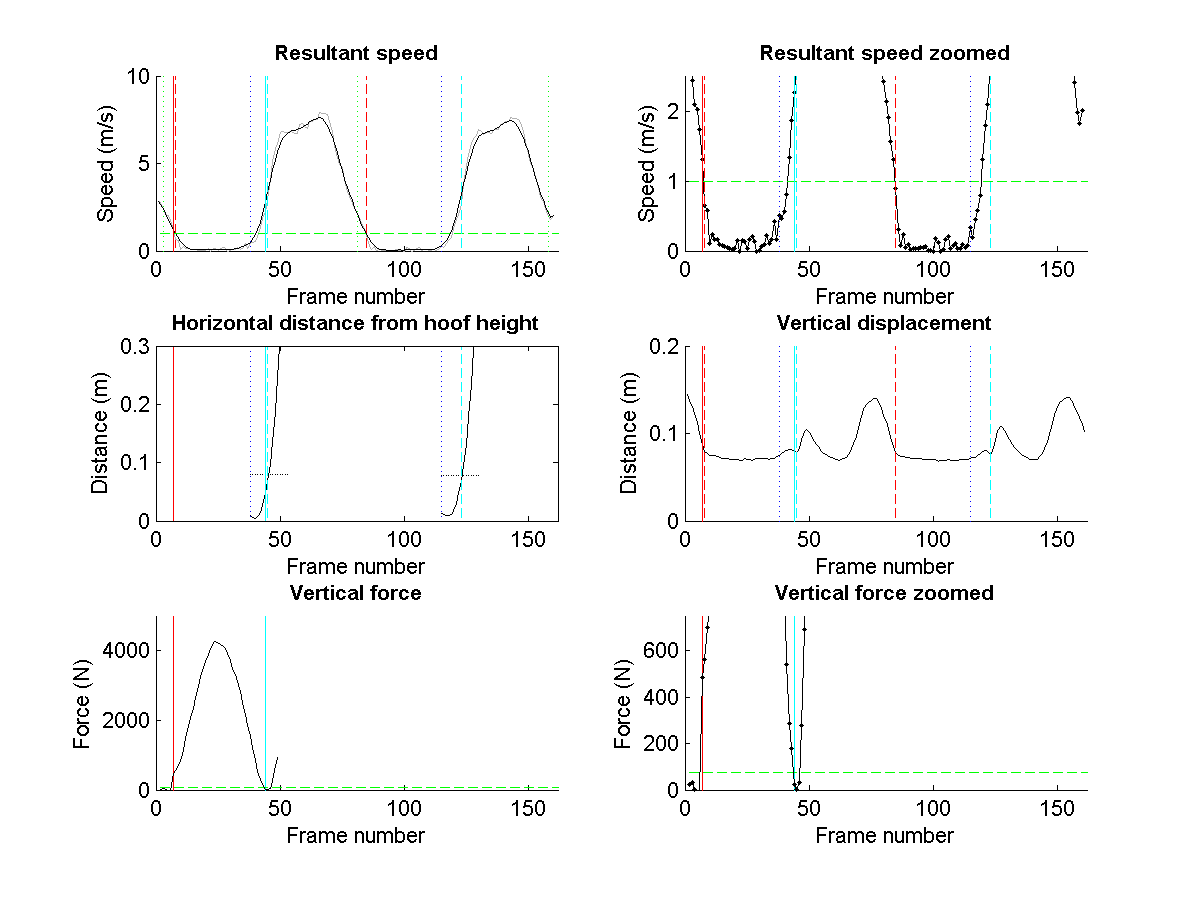

Supplement: Supplemental Information 3 [file peerj-03-783-s003.zip › Suppl figures/Threshold-based/Event_plot-LF_Horse10_circle_left_trot_03.png]

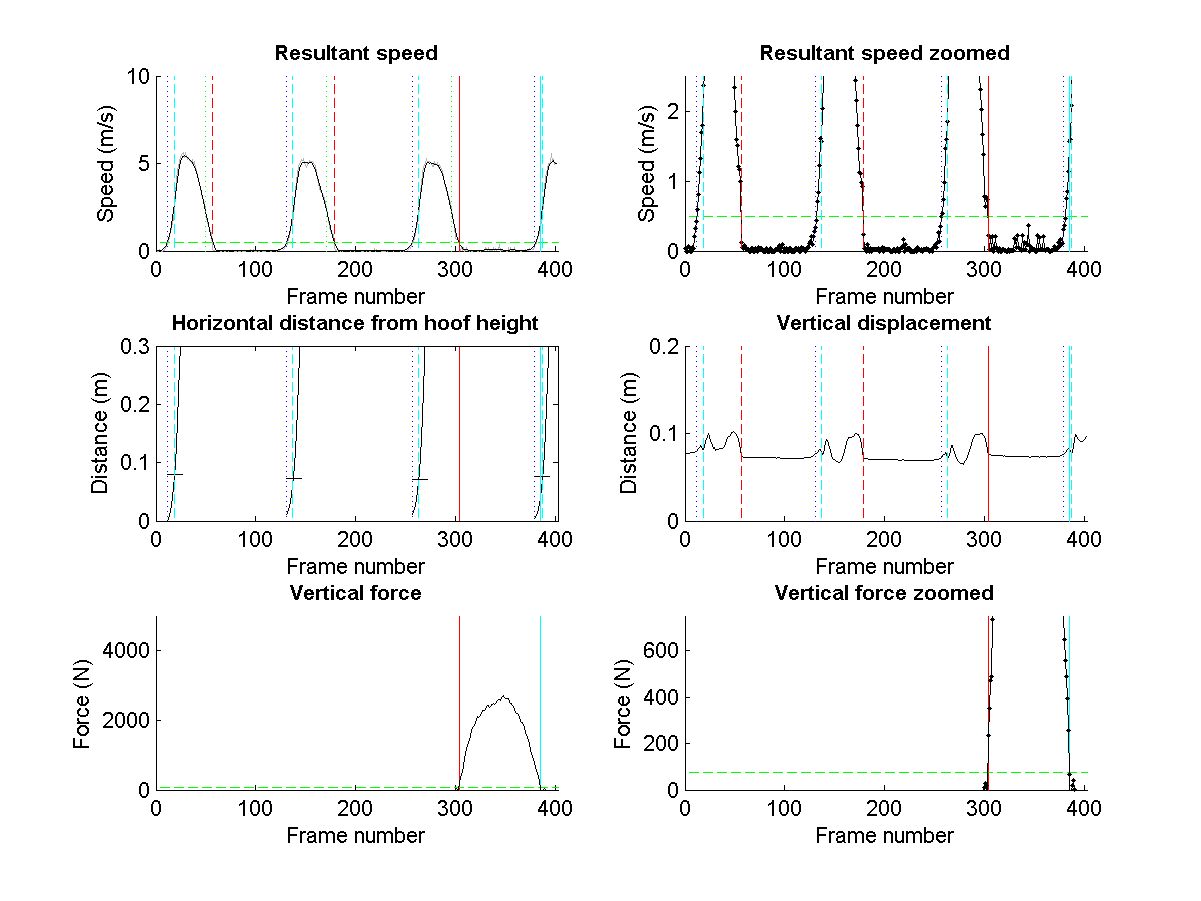

Supplement: Supplemental Information 3 [file peerj-03-783-s003.zip › Suppl figures/Threshold-based/Event_plot-LF_Horse10_circle_left_walk_02.png]

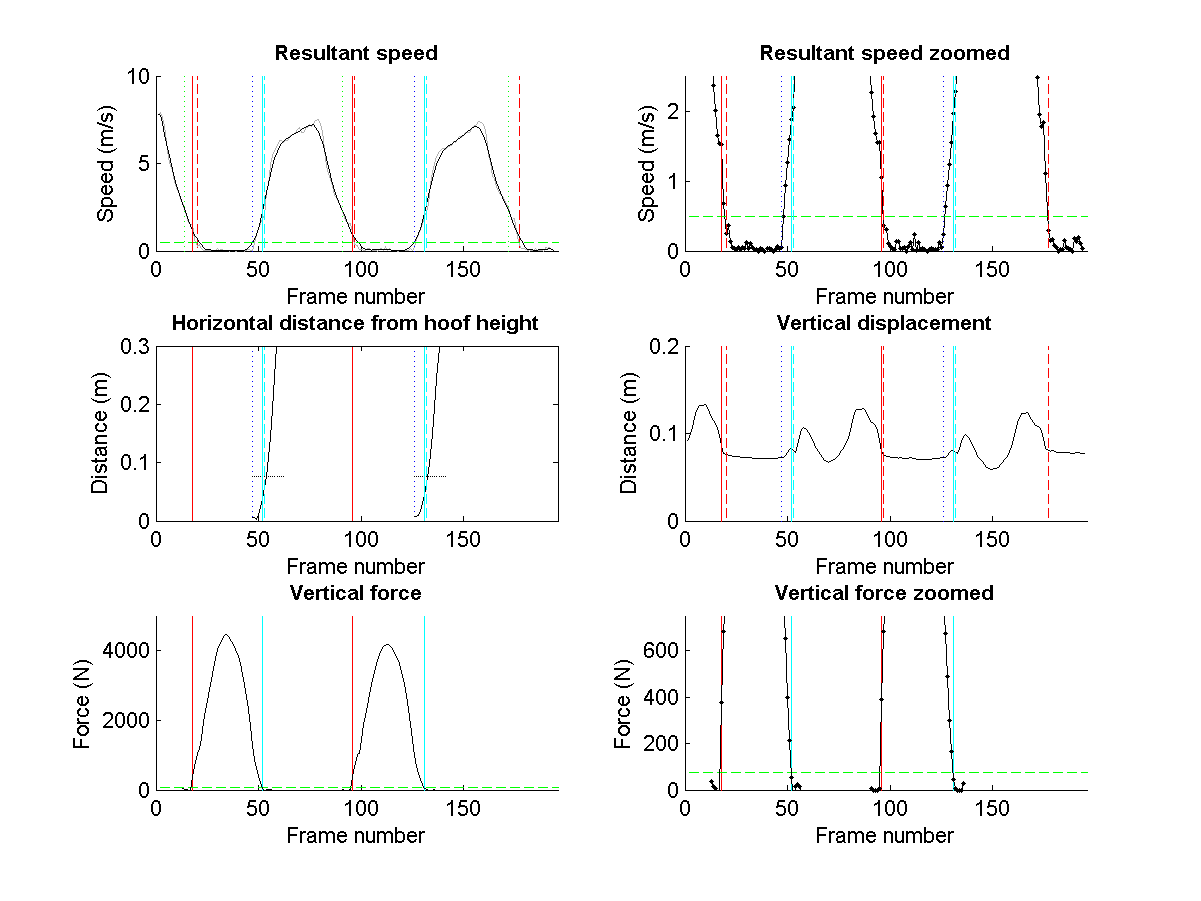

Supplement: Supplemental Information 3 [file peerj-03-783-s003.zip › Suppl figures/Threshold-based/Event_plot-LF_Horse10_circle_right_trot_03.png]

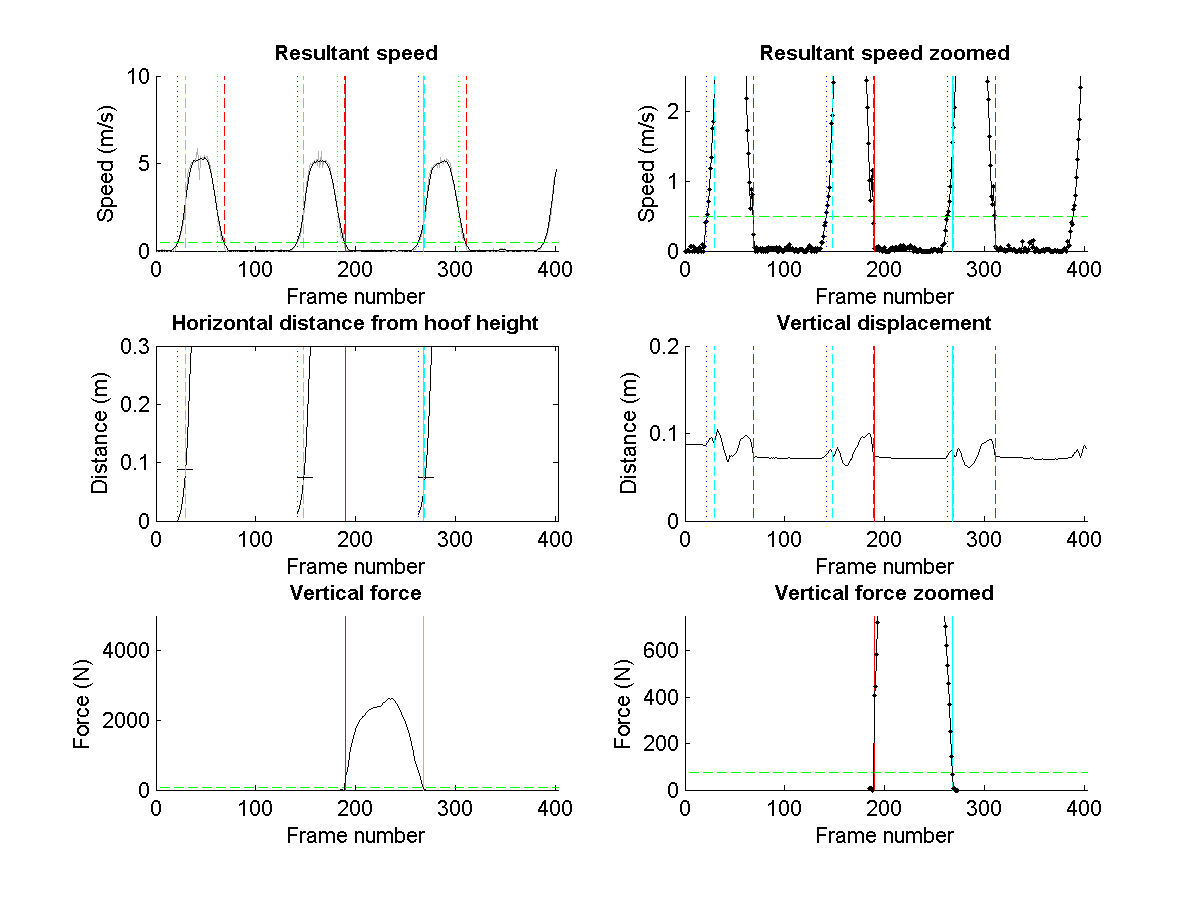

Supplement: Supplemental Information 3 [file peerj-03-783-s003.zip › Suppl figures/Threshold-based/Event_plot-LF_Horse10_circle_right_walk_08.png]
